# Probing the Mechanism of Action of Bis(phenolato) Amine (ONO Donor Set) Titanium(IV) Anticancer Agents

**DOI:** 10.1021/acs.jmedchem.3c01874

**Published:** 2024-02-08

**Authors:** Mustapha Musa, Mohammed Abid, Tracey D. Bradshaw, David J. Boocock, Clare Coveney, Stephen P. Argent, Simon Woodward

**Affiliations:** † GSK Carbon Neutral Laboratories for Sustainable Chemistry, University of Nottingham, Triumph Road, Nottingham NG7 2TU, U.K.; ‡ BDI, School of Pharmacy, University of Nottingham, University Park, Nottingham NG7 2RD, U.K.; § School of Science and Technology, Nottingham Trent University, Clifton, Nottingham NG11 8NS, U.K.; ∥ School of Chemistry, University of Nottingham, University Park, Nottingham NG7 2RD, U.K.; ⊥ Department of Chemistry, College of Science, University of Anbar, Anbarshire 31001, Iraq

## Abstract

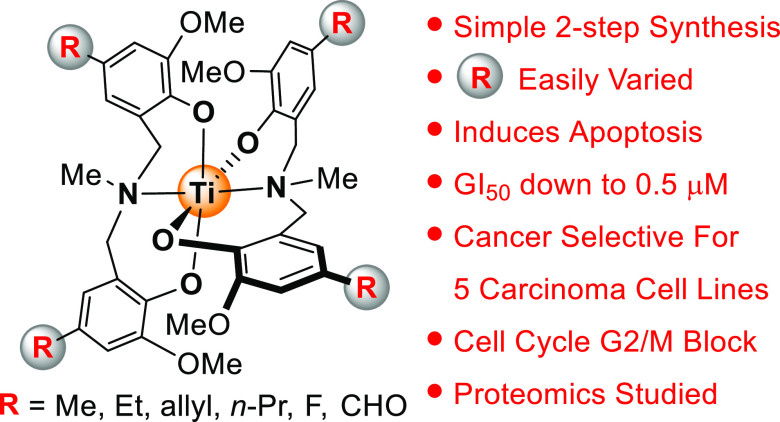

The need for anticancer therapies that overcome metallodrug resistance while minimizing adverse toxicities is targeted, herein, using titanium coordination complexes. Octahedral titanium(IV) *trans*,*mer*-[Ti{R^1^N(CH_2_–2-MeO-4-R^1^-C_6_H_2_)_2_}_2_] [R^1^ = Et, allyl, *n*-Pr, CHO, F, CH_2_(morpholino), the latter from the formyl derivative; R^2^ = Me, Et; not all combinations] are attained from Mannich reactions of commercial 2-methoxyphenols (27–74% overall yield, 2 steps). These crystalline (four X-ray structures) Ti(IV)-complexes are active against MCF-7, HCT-116, HT-29, PANC-1, and MDA-MB-468 cancer cell lines (GI_50_ = 0.5–38 μM). Their activity and cancer selectivity (vs nontumor MRC-5 cells) typically exceeds that of cisplatin (up to 16-fold). Proteomic analysis (in MCF-7) supported by other studies (G2/M cell cycle arrest, ROS generation, γH2AX production, caspase activation, annexin positivity, western blot, and kinase screens in MCF-7 and HCT-116) suggest apoptosis elicited by more than one mechanism of action. Comparison of these data to the modes of action proposed for salan Ti(IV) complexes is made.

## Introduction

**Figure 1:**
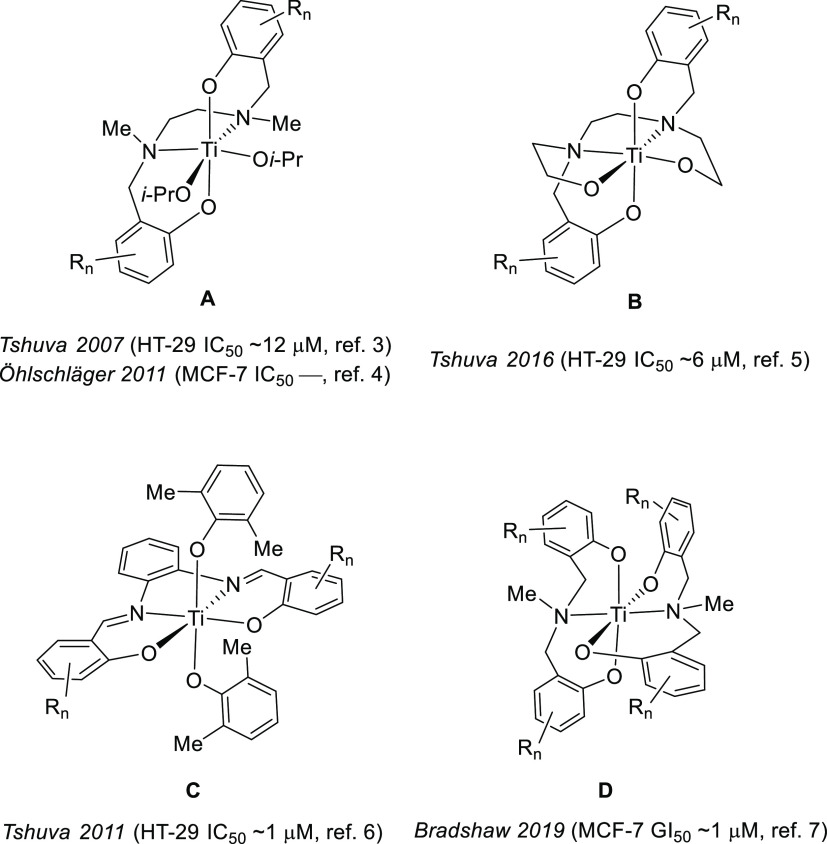
Contemporary titanium(IV)-based anticancer motifs **A–D**, with representative in vitro activity values against HT-29 and MCF-7 cell lines.^3−7^ Cisplatin shows activities of ∼20 and ∼8 μM, respectively, in the same two cell lines. Variation of the substituents (*R*_n_) is possible for each motif (for specific examples see refs (3–7)), but typically *R*_n_ are simply methyl, halogen, or alkoxy units.

Over the last two decades new ranges of phenolate-ligated titanium(IV) complexes have been defined^1,2^ in experimental anticancer studies (**A–D**, Figure 1).^3−7^ Titanium(IV) anticancer agents are of contemporary interest as, to the best of our knowledge, there is *no* reported example of any such species leading to development of acquired cancer cell line resistance,^1−7^ as is commonly observed for cisplatin.^8^ Indeed, salan Ti complexes have demonstrated activity against cisplatin-resistant A2780 ovarian cancer cells.^9^ This may indicate that titanium(IV) species can target conserved cellular processes that cannot be out-evolved. Contemporary phenolato, homoleptic, and heteroleptic titanium-based experimental anticancer agents now frequently deliver in vitro activities at low micromolar (μM) IC_50_ (or GI_50_) concentrations, frequently outperforming cisplatin and beckoning investigation of their mode(s) of action en route to clinical use.^1,2,10−12^ Unfortunately, historical cellular mechanistic investigations of titanium-based agents have been dogged by paradoxes rather than insight for >40 years, even for those species previously trialed in the clinic.^2^ The exact processes by which titanium cellular uptake occurs are still not fully defined. For the few anticancer titanium agents where cellular titanium burdens have been determined,^3,6,7^ only femtomol (10^–15^ mol) amounts of the metal per treated cancer cell have been found (see also the Supporting Information, Figure S62). Currently, only fluorescence imaging techniques (requiring tagged model compounds, sometimes of unknown relevance to the actual titanium drugs) have proved sensitive enough to allow the detection of such levels of titanium in treated cancer cells. The spatial resolution of these studies somewhat limits confidence in Ti-binding site assignment(s), but presently these suggest a biological target away from the cell nucleus.^13−16^ This is contrary to early reports,^17^ that proposed that the clinical candidate titanocene dichloride (η-C_5_H_5_)_2_TiCl_2_ accumulated mostly in the nucleus and/or chromatin. Contemporary data on substituted titanocenes are not in accord with that proposal.^18^ The site(s) of cancer cell Ti binding have been the subject of wide debate,^2^ and 2013 work by Tshuva et al. added the mitochondrion as a further potential target organelle for titanium agents.^19^ In 2020, accumulation of phenolate-ligated titanium agents within the endoplasmic reticulum (ER) of MCF-7 cells was proposed.^20^ The resultant ER stress, causing protein misfolding, was proposed as the preferred “mechanism of action” for agents of motif type **B** (Figure 1).^5,20^ The induced misfolded protein was suggested as the trigger for observed MCF-7 apoptosis brought about by such agents. As we recently (2019) identified the additional new motif **D** as an active titanium agent (Figure 1),^7^ we were intrigued to see if our species was related to class **B** in its mode(s) of action.

## Results and Discussion

**Scheme 1:**
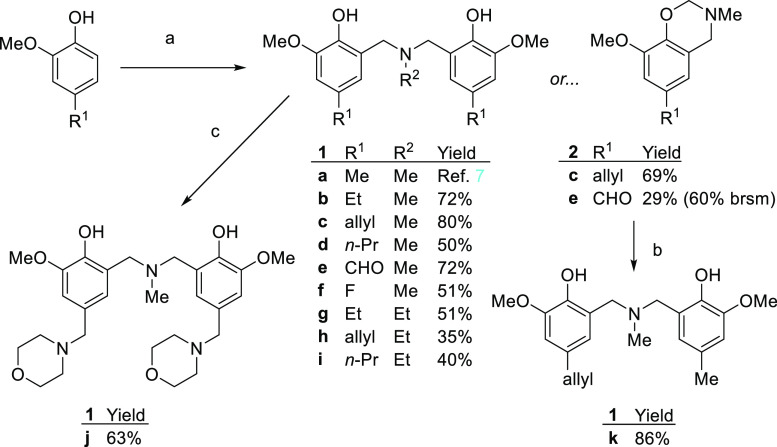
Preparation of Bis(phenolato) Amines (**1a**–**k**) Used in This Study Reagents and conditions: (a) parent 2-methoxyphenol (1 M in MeOH), 37% w/w aqueous formaldehyde in water (3 equiv), followed by 40% w/w methylamine in water (2 equiv) at room temperature for 36 h and then (if needed) at 65 °C for 4 h; (b) benzoxazine **2** (1 equiv) and substituted 2-methoxyphenol (1.2 equiv) mixed neat and heated to 100 °C for 4 h; (c) ligand **1e** (MeOH, 0.4 M), morpholine (2.2 equiv), picoline·borane (3 equiv), room temperature, 16 h. For preparation of **1a**, see ref (7).

One common issue for titanium(IV) complexes as potential therapeutics is establishing mild functional group tolerant methodologies that easily install points of derivatization (i.e., via *R*_n_, Figure 1) into their ligands. This is desirable for simplifying drug library design in structure–activity studies, and ultimately for optimization of drug delivery and related pharmacokinetic factors. For example, while our own motif **D** lead ligand **1a** (Scheme 1) used simple Mannich chemistry for its preparation, the forcing conditions (125–150 °C, acid solvent)^7^ used were incompatible with many useful functional groups for derivative formation. We now find that more concentrated solutions of 2-methoxyphenol derivatives (1 M, MeOH) give acceptable yields (35–88%) of symmetrical ligands **1a**–**i** typically at room temperature (followed, in some cases, by mild warming). These reactions proceed via the intermediate benzoxazines **2**, and, in favorable cases (**2c**, **2e**), these can be isolated at ambient temperature (with the mass balance being the derived ligands **1c**, **1e,** and starting phenol). As both alkenes and formyl groups are tolerated under these new conditions, low cost renewable eugenol (R^1^ = allyl) and vanillin (R^1^ = CHO) become attractive starting materials. The nonparticipation of the formyl substituent in the reaction of vanillin is ascribed to its conjugation to the phenol OH. Ambient temperature chemoselective Mannich reactions of vanillin are apparently very rare.^21^ The isolated benzoxazines **2** thus allow for the formation of mixed systems, as in the preparation of **1k**. Bis(formyl) **1e** readily undergoes reductive amination with amine/pinacol·borane mixtures. Representative morpholine is shown, providing mild diversification to exemplar **1j**. One slight complication is the tendency of **1j** to form borate complexes with the borane-derived byproducts. However, decomplexation is affected by a simple acid treatment (HCl, 12 M).

**Scheme 2:**
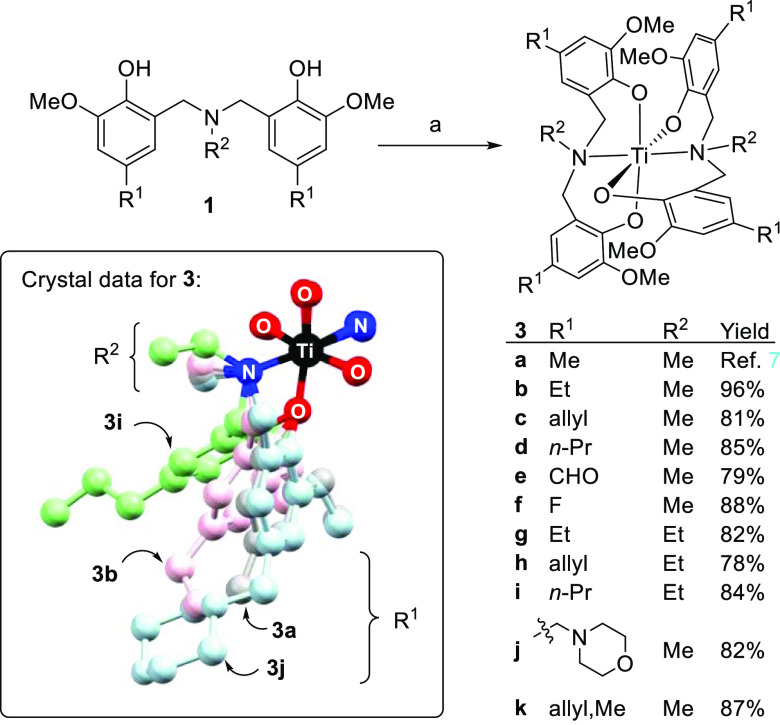
Preparation of Bis(phenolato) Amine Titanium(IV) Complexes (**3a**–**k**) Used in This Study Reagents, conditions, and notes: (a) ligand **1** (0.25 M in toluene), Ti(O*i*-Pr)_4_ (0.8 equiv) at room temperature for 4 h. For preparation of **3a**, see ref (7). (b) Comparative crystallographic data for **3b** (pink), **3i** (green), and **3j** (blue) vs the lead **3a** (gray) are shown overlapped (each with one Ti, O, and N atom coincident) in the box (see also Supporting Information for full data and discussion, including structure of **3c**). Only one of the four 2-methoxy aryl and two amine substituents are shown for clarity.

The derived bis(phenolato) amine titanium(IV) complexes **3** are easily prepared by the reaction of **1** with Ti(O*i*-Pr)_4_ (Scheme 2). All these are highly crystalline and easily purified (≥99% by CHN analysis) to levels appropriate for biological studies. Crystallographic studies show the compounds **3** are nearly isostructural in the solid-state (box within Scheme 2 and the Supporting Information, Figures S47–S58). All of structures **3a**–**c** and **3i**–**j** show Ti–O and Ti–N bond lengths in the range expected for Ti(IV) phenolate complexes, Ti–O: 1.858–1.909 Å and Ti–N: 2.244–2.269 Å (see the Supporting Information, Table S1 and associated CIF files). Complex **3i** shows the largest structural difference compared to the parent **3a**, which may be pertinent to its poorer biological activity (see later). Region R^2^ is poorly tolerant of steric factors; all attempts to complex ligands **1** bearing R^2^ = *i*-Pr to titanium(IV) failed. This distortion is reflected in the N–Ti–N bond angle, which rises from 173.2 to 179.1° in structure **3b** vs **3i**. The X-ray study of **3k** shows the presence of a water solvate hydrogen bonded to one of the morpholine nitrogens, consistent with its lower *C* Log *P* value (Table 1).

**Table 1:** Growth Inhibitory Activity of **3a**–**k** (Mean ± SD GI_50_ Values in μM, MTT Assay, 72 h) as a Function of Changing R^1^ (R^2^ = Me in all Cases) and Treated Cell Lines^a^

**3**	R^1^	*C* Log *P*(**1**)^b^	V(R^1^)^b^ (Å^3^)	σ_p_(R^1^)^b^	MCF-7	HCT-116	HT-29	PANC-1	MDA-MB-468	MRC-5
**a**	Me	3.0064	19.6	–0.17	**1.0±0.04**	3.4 ± 0.07				7.3 ± 0.04
**b**	Et	4.0644	38.9	–0.15	**1.3±0.2**	**0.5±0.1**	17.5 ± 0.4	**1.9±0.2**	**2.4±0.1**	9.3 ± 0.3
**c**	allyl	4.1544	53.5	–0.14	**2.4±0.2**	8.6 ± 0.3	7.5 ± 0.2	4.3 ± 0.1	**2.5±0.3**	8.2 ± 0.2
**d**	*n*-Pr	5.1224	56.2	–0.13	17.6 ± 0.3	7.4 ± 0.3	10.2 ± 0.2	16.7 ± 0.3	13.1 ± 0.3	17.7 ± 0.4
**e**	CHO	1.9286	27.7	+0.42	26.1 ± 0.1	48.4 ± 0.4	38.2 ± 0.2	20.2 ± 0.6	30.0 ± 0.4	52.1 ± 0.3
**f**	F	2.7377	10.3	+0.06	2.9 ± 0.3	5.7 ± 0.4	9.1 ± 06	**1.2±0.4**	3.8 ± 0.3	13.5 ± 0.4
**j**	CH_2_NR_2_^c^	1.5024	98.2	+0.01^c^	7.8 ± 0.4	13.2 ± 0.1	6.8 ± 0.4	14.8 ± 0.2	7.7 ± 0.5	18.5 ± 0.3
**k**	allyl, Me	3.5804	36.6^d^	–0.16^d^	7.9 ± 0.4	18.4 ± 0.5	25.0 ± 06	11.9 ± 0.5	7.4 ± 0.7	16.3 ± 0.6
	cisplatin	–2.19			7.6 ± 0.2	8.2 ± 0.4	16.0 ± 0.4	13.1 ± 0.5	4.9 ± 0.3	7.9 ± 0.6

a Data generated from ≥3 independent trials; *n* = 8 per experimental condition per trial.

b Ligand *C* Log *P* values from ChemDraw (ver. 20); R^1^ substituent volumes^22^ and Hammett parameters^23^ from literature sources.

c No Hammett parameter is available for CH_2_(morpholino), the value for CH_2_NMe_2_ is given.^23^

d Average of allyl and Me values.

Concentrations of titanium complexes **3b**–**k** that inhibited cell growth by 50% (GI_50_ values) in six cell lines were obtained from MTT studies and are shown in Tables 1 and 2, with comparison to literature^7^**3a** where possible.

**Table 2:** Growth Inhibitory Activity of **3h**–**j** (Mean ± SD GI_50_ Value in μM, MTT Assay, 72 h) as a Function of Changing R^1^ (R^2^ = Et in all Cases) and Treated Cell Lines^a^

**3**	R^1^	*C* Log *P*(**1**)^b^	V(R^1^)^b^ (Å^3^)	σ_p_(R^1^)^b^	MCF-7	HCT-116	HT-29	PANC-1	MDA-MB-468	MRC-5
**h**	Et	4.5934	38.9	–0.15	7.8 ± 0.4	13.2 ± 0.1	6.8 ± 0.4	14.8 ± 0.2	7.7 ± 0.5	18.5 ± 0.3
**i**	allyl	4.6834	53.5	–0.14	7.9 ± 0.4	18.4 ± 0.5	25.0 ± 06	11.9 ± 0.5	7.4 ± 0.7	16.3 ± 0.6
**j**	*n*-Pr	5.6514	56.2	–0.13	6.4 ± 0.2	15.4 ± 0.3	29.1 ± 0.2	11.4 ± 0.1	17.7 ± 0.3	25.3 ± 0.5
	cisplatin	–2.19			7.6 ± 0.2	8.2 ± 0.4	16.0 ± 0.4	13.1 ± 0.5	4.9 ± 0.3	7.9 ± 0.6

a Data generated from ≥3 independent trials; *n* = 8 per experimental condition per trial.

b Ligand *C* Log *P* values from ChemDraw (ver. 20); R^1^ substituent volumes^20^ and Hammett parameters^23^ from literature sources.

**Figure 2:**
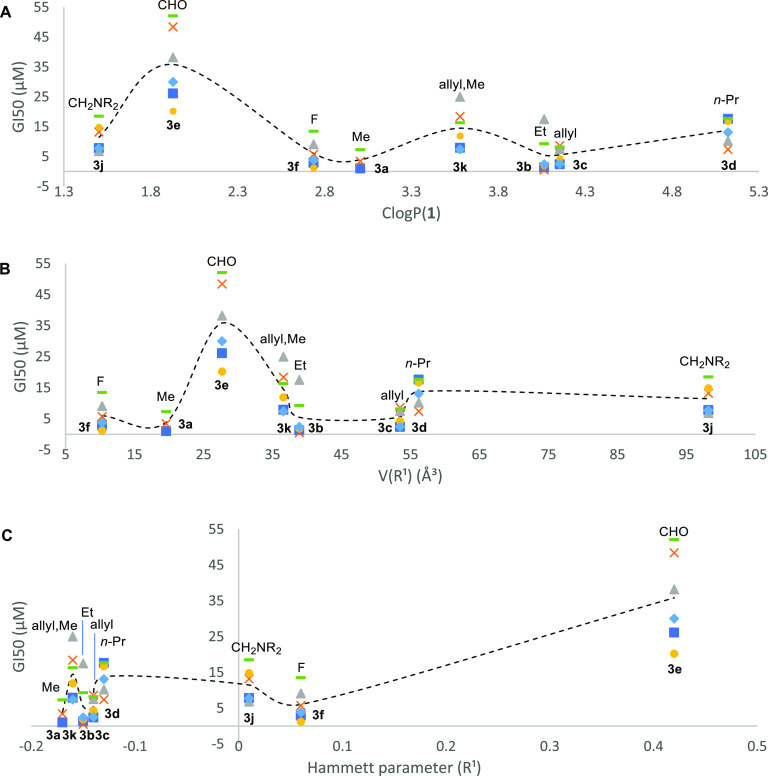
Analysis of the growth inhibitory activity (mean ± SD GI_50_ value in μM, MTT assay, 72 h) of complexes **3a**–**k** vs selected ligand features of **1**: (A) *C* Log *P*; (B) V(R^1^); and (C) σ_p_(R^1^). Key: (Royal blue) ■MCF-7, (mustard) ×HCT-116, (gray) ΔHT-29, (yellow) ●PANC-1, (pale blue) ◆MDA-MB-468, (green) —MRC-5, and (black) ---average GI_50_ value for all six cell lines studied.

Tables 1 and 2 indicate that greater activity compounds are attained when R^2^ = Me. Using the data of Table 1, Figure 2 plots three simple ligand features supporting the following conclusions: (i) ligand **1** polarities of 2.7–4.2 (*C* Log *P*) are typically associated with the highest activities, (ii) scope exists for a wide range of R^1^ volumes to be accommodated (without dramatically lowering overall activity), and (iii) mesomeric electron withdrawing groups at R^1^ lower overall anticancer activity. These three main ligand features cointeract providing the observed SAR. Additionally, while opportunities exist for maximizing activity for cancer cell lines (over representative noncancer MRC-5 cells), those factors are too complex to model accurately at present. Selectivity indices of 0.5–18.6 can be derived from the values of Table 1 (see also the Supporting Information, Table S4).

Based on Tables 1 and 2, new agents **3b**–**c** were selected for further scrutiny, focusing mainly on MCF-7 and HCT-116 cell lines. Cell counts and clonogenic assays of **3b**–**k** confirm the cytotoxic nature of antitumor activity detected in the initial MTT studies (see the Supporting Information, Figure S58).

**Figure 3:**
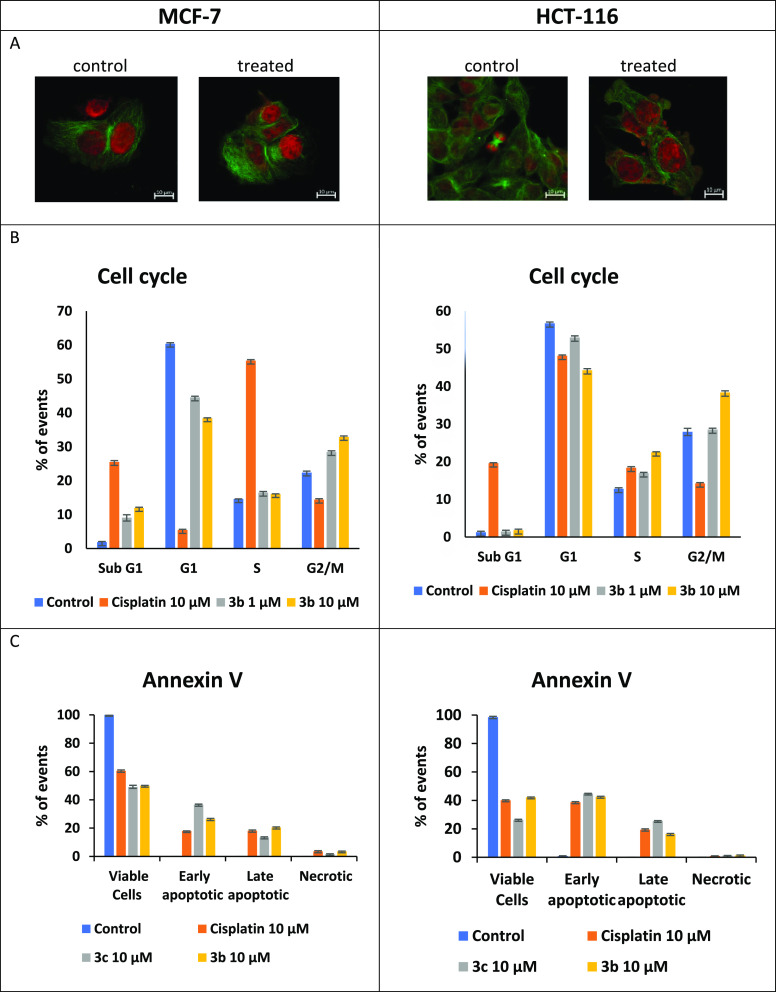
Studies of the effects of **3b** and/or **3c** on MCF-7 (left) or HCT-116 (right) cells vs untreated controls. (A) Confocal microscopy (**3c**, 10 μM, 24 h). Nuclear regions are shown in red (DRAQ5) and cell membrane regions in green (secondary antibody). The scale bars are 10 mm; for **3b** data see Supporting Information, Figure S72. (B) Cell cycle perturbation after treatment with **3b** and **3c** (10 μM, 72 h); for **3c** data see Supporting Information, Figures S68 and S69. (C) Annexin-V/PI apoptosis assay for **3b** and **3c** (10 μM, 72 h). Also, see Supporting Information, Figure S70.

**Figure 4:**
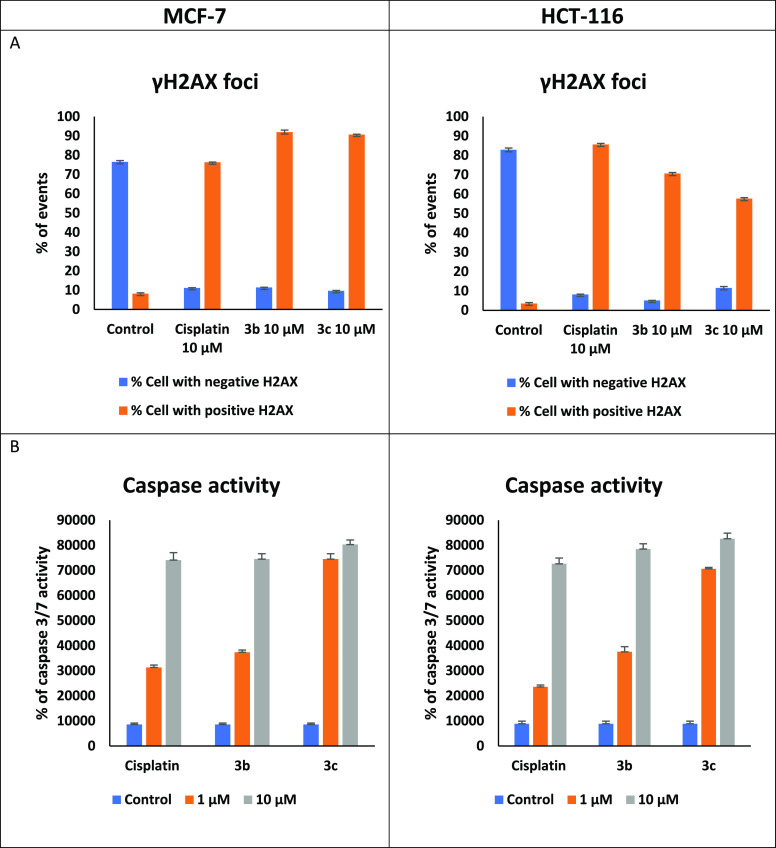
Studies of the effects of **3b** and/or **3c** on MCF-7 (left) or HCT-116 (right) cells vs untreated controls. (A) DNA double strand breaks were detected by γ-H2AX after treatment with **3b** and **3c** (10 μM, 72 h). (B) Dose-dependent elevation of caspase 3/7 activity induced by **3b** and **3c** (1 and 10 μM, 72 h).

Microscopy imaging and flow cytometry (cell cycle, annexin-V/PI) show that agents **3b**–**c** induce apoptosis in both MCF-7 and HCT-116 cells (Figure 3). Both chromatin condensation and membrane blebbing are imaged in **3b-** and **3c**-treated cells (Figure 3A and Supporting Information, Figure S72), which are characteristic morphological features of apoptosis.^24^ The fraction of cells in G2/M phases is significantly increased for both MCF-7 (1.4× control) and HCT-116 (1.8× control) cells for both **3b** and **3c** (Figure 3B, see also Supporting Information, Figures S68 and S69). Both complexes result in associated increases in the percentage of cells undergoing apoptosis (Figure 3C and Supporting Information, Figure S70), similar to that observed for cisplatin. Additional evidence that **3b**–**c** trigger apoptosis in response to catastrophic DNA damage is provided by γ-H2AX detection and caspase 3/7 activation (Figure 4 and Supporting Information). Both the presence of extensive DNA double strand breaks and enhanced caspase 3/7 activity are fully consistent with apoptosis triggered by DNA damage, causing arrest at the G2/M cell cycle check point, as has been observed in other phenolate-based titanium agents.^7,20^

The biological features observed in Figures 3 and 4 may correlate with signaling initiated by an extracellular ligand trigger. Evidence to support this thesis can be seen in UV–vis spectra (10 μM, ligand-to-titanium charge transfer band at 330 nm in cell growth medium) of **3c**. These show no change over 24 h, indicating that **3c** is stable in aqueous media for at least that period. After that time, slow hydrolysis of **3c** is detected by UV–vis spectroscopy, amounting to ca. 2% of **3c** per day. In the same culture medium containing MCF-7 cells, freshly prepared **3c** (10 μM initial concentration) is consumed at a much higher rate: [0.024(2) h^–1^], identical within experimental error to that of the growth of the treated MCF-7 cells [0.028(5) h^–1^] for the first 8 h (Supporting Information, see Figure S59). In the absence of **3c**, MCF-7 grows at a faster rate [0.043(4) h^–1^] with doubling times (16 h) that are identical, within experimental error, to literature values.^25^ MCF-7 cell driven consumption of **3c** starts immediately after the addition of **3c** and amounts to ca. 10 femtomol per cell at 8 h. After 8 h, the treated MCF-7 cell growth rate recovers partially [0.06(1) h^–1^], and the rate of **3c** depletion also increases [to 0.036(3) h^–1^]. From this time, populations of dead MCF-7 cells begin to emerge at [0.41(4) h^–1^]. The overall behavior is consistent with **3c** being the ultimate source of the growth inhibition that is recorded as MCF-7 G2/M arrest and which subsequently promotes apoptosis. The causative agent is clearly **3**, or a species derived from it. In that regard, we could detect the formation of free ligand **2c**, by LCMS, under aqueous conditions mirroring preparation of biological stock solutions of **3c** (200 μM, see Supporting Information, Figure S60). After 2 days, ≤4% ligand was detected, rising to 16% **2c** after 5 days. Similarly **3c** (200 μM in 4:1 DMSO–D_6_/D_2_O corresponding to the presence of 9.4 M water/4.7 × 10^4^ equivalents of D_2_O) remained completely intact for at least 2 days (see Supporting Information, Figure S61). At longer times, smooth formation of a new titanium species over 4 weeks is seen. The +ESI mass ion observed for the hydrolysis species is consistent with the formation of [LTi(OH)(OH_2_)]^+^ where L is the bis(phenolato) dianion of **2c** by hydrolytic cleavage of one of **2c** ligands. While the ability of complex **3**, or derived species, to bind to cells is demonstrated, we have not yet identified their localization in specific organelles.

To try to understand the G2/M block/apoptosis response elicited and elucidate possible molecular (cellular) targets for **3**, we undertook a proteomic analysis of the ca. 3100 proteins quantifiable by DIA(SWATH) LC-MSMS after MCF-7 cells are treated with **3c** (10 μM, 24 h) and lysed (see Supporting Information, Excel File “Proteomics”). In comparison to untreated MCF-7, those exposed to agent **3c** show significant (−3.49962 log_2_-fold change, ρ 0.0022) downregulation of protein CDK1 (the serine/threonine protein kinase critically involved in cell cycle regulation^26^). CDK1 is specifically responsible for enabling the G2/M phase transition; thus, onward cell division is impeded by its low availability. However, the concentration of CDK1’s archetypal p21 interaction protein, also associated with the normal inhibition of CDK1,^27^ was not affected (log_2_-fold changes +0.04 vs the control, Supporting Information, Excel File “Proteomics”). Similarly, all the other CDK proteins we could analyze in our study (2/5/6/7/9/11) were only modestly upregulated (log_2_-fold change +0.20 to +1.40, Supporting Information, Excel File “Proteomics”). CDK2 (which facilitates nuclear export) was the next most affected protein, consistent with DNA damage being present at the G2/M checkpoint.^28^ Selective CDK1 inhibition is rare^29^ being previously seen only for competitive ATP binding to that kinase. However, as the Wee1 and Cdc25 proteins (dictating the “hold” or “go” signals to CDK1) could not be analyzed in our protein set, definitive confirmation of CDK1 inhibition would need future work. Proteomic pathway analysis suggests a modified immune response as an alternative likely inhibition process. Immune response evasion is a hallmark of cancer, and the suppression of this innate ability by **3c** is also a viable possibility. Typically, growth factor signaling is required to initiate MCF-7’s entry into its division cycle. Interaction, at the cell surface, with receptor tyrosine kinase (RTK) sites initiates all subsequent events. The RTK-linked membrane bound ras-GDP signals to the mitogen-activated protein kinase (MAPK)/ extracellular signal-regulated kinase (ERK) cascade causing ERK translocation to the nucleus where multiple target interactions are possible. In line with this proposal, in **3c** treated MCF-7 cells, the Ras GTPase-activating protein is significantly downregulated (−2.00630 log_2_-fold change, Supporting Information, Excel File “Proteomics”), with a smaller, but notable effect on MAPK-1 (−0.29654 log_2_-fold change). We note that perturbation of MAPK signaling has been noted in previously studied titanium complexes.^18^

Three of the key downregulated proteins we identify (CDK1, PABPC, and NQ01) have equivalent genes that are implicated in anticancer activity in related RNA studies of Tshuva and co-workers.^20^ Our observation of CKD1 downregulation is in agreement with that earlier study, but their observation of PABPC upregulation (at 24 h) is different from our own findings, even though the cell line studied is identical (MCF-7) in both cases. In the study by Tshuva and co-workers,^20^ NQ01 regulation depends on the time course of exposure (upregulated between 15 and 24 h, then downregulated at 48 h). Our proteomic analyses imply very significant downregulation for this protein/gene at 24 h. As NQ01 is a superoxide scavenger, its downregulation is consistent with the reactive oxygen species (ROS) generation uptick we also observe (see later). To support our proteomic studies, we also investigated the qualitative behavior of the 23 proteins that we could map to the earlier RNA study^20^ (Table 3). The expression trend analysis within our proteomic study supports Tshuva’s implication of the involvement of the mitochondrial translation pathway. However, it did not completely support the suggested protein processing involvement in the ER pathway, even though MCF-7 was used in both studies. Overall, our data analysis indicates the possibility that perturbations of a rich and diverse pharmacology are viable, even for closely related titanium agents.

**Table 3:**
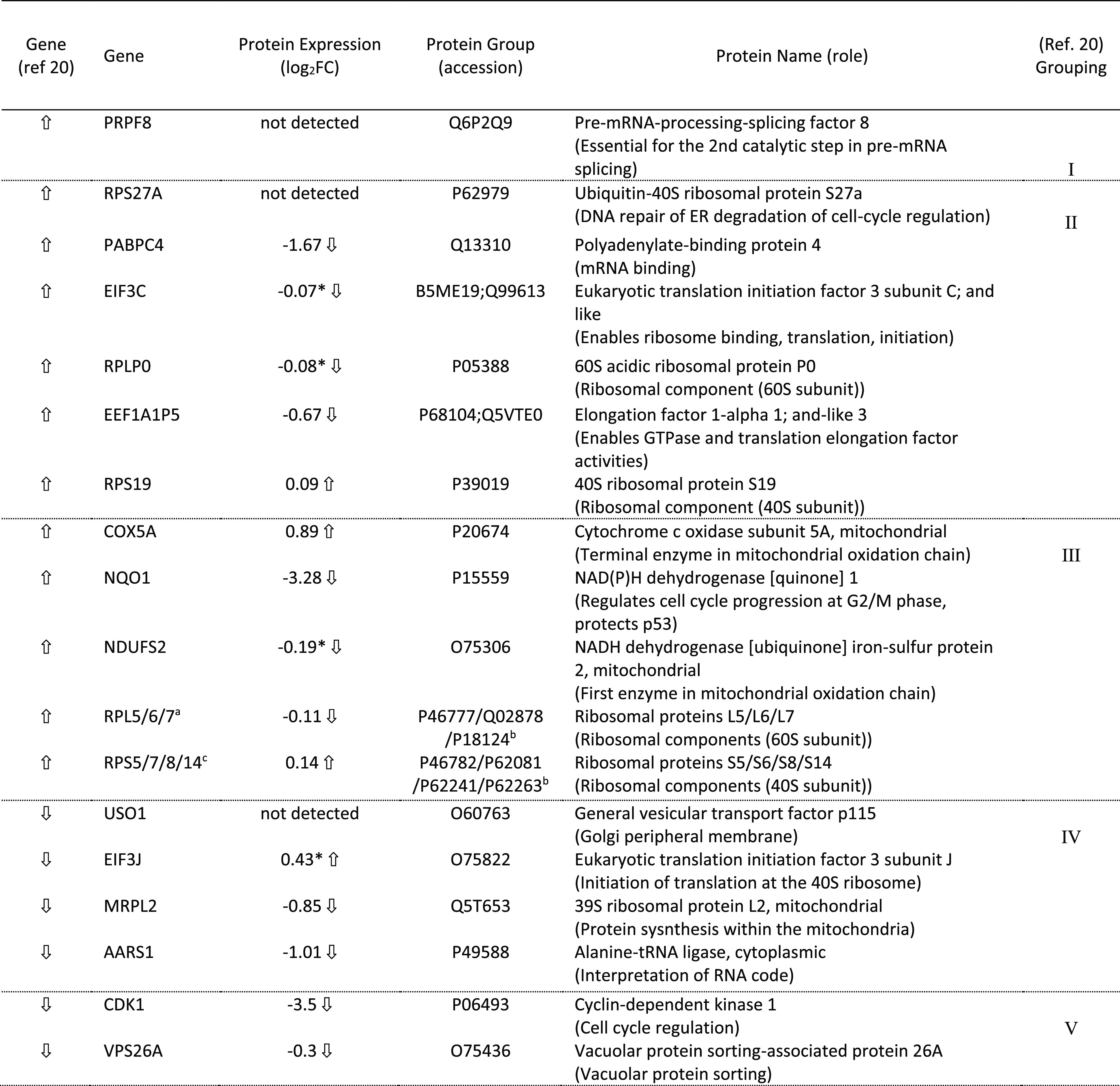
Comparison of the RNA Genomic Study of MCF-7 (Ref (20), Ti Salan Agent Type **B**,^5^ 54 μM) at 24 h Vs Our Proteomic Study Using MCF-7 and **3c** (10 μM, 24 h Exposure)^a^

a Similar and opposite trends in the two studies have been highlighted. *Non-significant fold change *p* > 0.05.

b Average of all gene results, log_2_ range 0.89 to 0.99.

c Average of all proteomic results, log_2_ range −0.13 to −0.09.

d Average of all gene results, log_2_ range 1.03 to 1.60.

e Average of all proteomic results, log_2_ range −0.04 to 0.23. See Supporting Information, Excel File “Proteomics”, for Table 3 primary data sources.

As we identified apoptosis as the mechanism of cell death, and Bcl-2 and Mcl-1 are key pro-survival proteins, we interrogated by western blot, changes in expression of these key cancer survival (antiapoptotic) proteins (Bcl-2 and Mcl-1) in MCF-7 and HCT 116 cells following exposure to **3b**/**3c** (Figure 6 and Supporting Information, Figures S74 and S75).^30^ Proteins Bcl-2^31,32^ and Mcl-1^33,34^ work together to counter apoptosis by limiting mitochondrial membrane release of cytochrome *c*;^35,36^ they are typically overproduced in cancer cells, and their expression is closely associated with poor prognosis and drug-resistance. Profoundly reduced expression of Bcl-2 and Mcl-1 in cells treated with **3b**/**3c** is in line with apoptosis induction and reduced immune response evasion indicated in the proteomic study. This pathway is also consistent with receptor-mediated (MAPK/ERK) apoptosis^37^ (see later).

**Figure 5:**
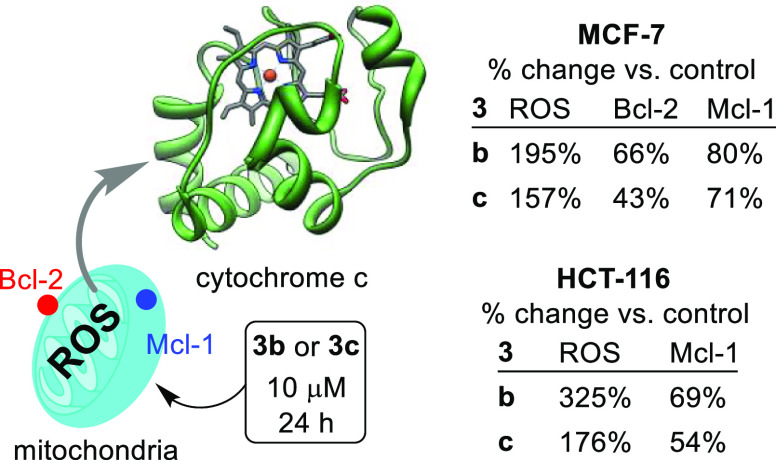
Change in species known to either promote ROS or apoptosis (downregulate proteins Bcl-2 and Mcl-1) through cytochrome c release from mitochondria vs controls; see the Supporting Information (Figures S74 and S75) for primary data. The structure of cytochrome *c* is from ref (41) and used with permission from Elsevier for reproduction, license number, 5531400991823; issue year, 2023.

**Figure 6:**
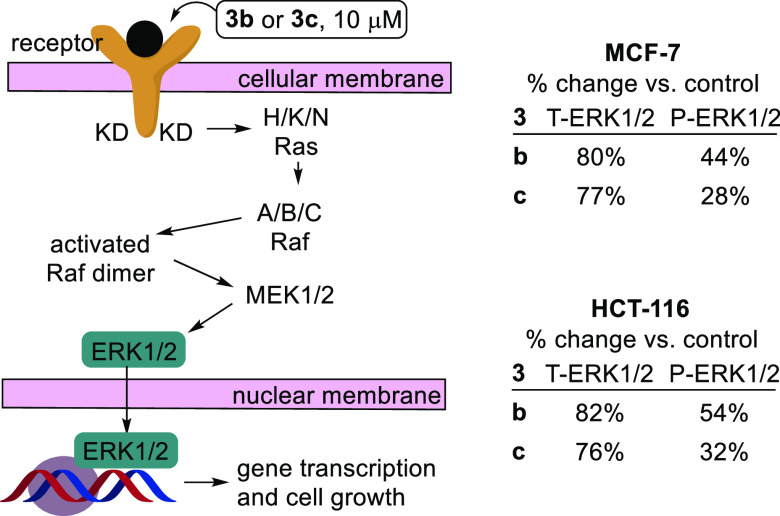
ERK1/2 as part of the MAPK cascade and its depletion in the presence of **3b**–**c**, as detected by western blot techniques. T-ERK1/2 and P-ERK1/2 are, respectively, the total ERK and phospho-ERK present.

In addition, because we detect DNA double strand breaks and metal complexes are known to damage DNA through ROS generation, we investigated whether **3b**/**3c-** induced MCF-7 and HCT 116 intracellular ROS formation.^38^ ROS are formed in mitochondria by electron chain reduction of O_2_ to form superoxide or by peroxisomes (through fatty acid oxidation) or by ER oxidation of proteins.^39^ Treated MCF-7 (**3c**, 10 μM, 24 h, Figure 6) shows nearly double the ROS of untreated controls. This is consistent with the NQ01 downregulation observed in our proteomic study. Excess ROS populations are known to promote cytochrome *c* release, through oxidation of mitochondrial pores, causing caspase activation and ultimately apoptosis.^40^ Consistently, ROS generation is noted in both HCT 116 and MCF-7 cells treated with **3b** as well as **3c** (Figure 5).

Complexes **3b**–**c** induce DNA damage. Poly(ADP-ribose) polymerase 1 (PARP1), an ADP-ribosylating enzyme becomes activated upon binding to DNA single strand and double strand breaks and is essential for initiating various forms of DNA repair.^42^ PARP is also a substrate for caspases—activated during apoptosis. Caspases cleave PARP during apoptosis and thus cleaved PARP emergence accompanied by downregulation of whole PARP has become a marker for apoptosis.^43^ MCF-7 cells treated with **3b** or **3c** (10 μM, 24 h) show, through western blots, highly reduced PARP populations (13–15% of control). Similar behavior is seen for the same two complexes in the HCT-116 cell line (12–13% of control). The reduced PARP (and associated increased cleaved PARP) are clear markers for DNA damage-induced apoptosis (see the Supporting Information, Figure S73).

The heterogeneous nuclear ribonucleoprotein M is strongly downregulated (−4.15369 log_2_-fold change, ρ 0.0005) in our proteomic study. The latter nuclear proteins sequester and transport RNA out of the nucleus and are closely associated with cell cycle regulation at the G2/M DNA checkpoint. Heterogeneous nuclear ribonucleoproteins regulate the surface receptor glycoprotein CD44^44^ that acts via a range of signaling kinases, but especially Ras-MAPK pathways, terminating in gene transcription at the nucleus. We thus used western blot techniques to quantify the marker ERK1/2 protein of the Ras-MAPK cascade. ERK1/2 cascade activation is typically initiated by membrane receptors such as RTKs, G protein-coupled receptors (GPCRs), and ion channel receptors, for example.^45^ These receptors transmit signals by recruiting adaptor proteins (e.g., Grb2) and exchange factors (e.g., son of sevenless, SOS), which in turn activate Ras. The active, GTP-bound Ras then delivers the signal by activating the Raf-1, B-Raf, and A-Raf (Rafs) protein kinases within the MAPK cascade.^45,46^ Ras/Raf/MEK/ERK1/2 signaling is triggered via a small GTPase-mediated activation of activated tyrosine receptors and cytoplasmic kinase signaling cascades.^47^ The key point of activation is the transmission of a signal from tyrosine kinase receptors, including the epidermal growth factor receptor, which then recruit SOS via intracellular Shc and Grb2 domains, catalyzing the conversion of inactive Ras/guanosine diphosphate to an active Ras/guanosine triphosphate complex.^47,48^ This ERK1/2 cascade is also a major signaling system that regulates not only many activated cellular activities, most notably proliferation, differentiation, and survival, but also apoptosis and stress response.^45^ The effect of compounds **3b**–**c** on ERK1/2 within the MAPK cascade is shown schematically in Figure 6 (see also the Supporting Information, Figures S74 and S75).

The reduction of ERK1/2 induced by **3b**–**c** is opposite to the increase seen in our earlier studies of the action of chiral titanocenes.^7^ However, in that case paraptotic cell death is induced by acceleration of cellular processes (paraptosis) vs the inhibition (apoptosis) seen here. This behavior is exemplary of the increasing repertoire of compounds that mediate ERK activation leading to apoptosis. While activation of ERK1/2 typically promotes cell proliferation, some compounds induce ERK activation while exerting antiproliferative effects. It is acknowledged that the mechanisms underlying ERK1/2-mediated cell death are still to be fully defined.^30,38^

## Conclusions

To conclude, we describe the synthesis of a series of octahedral titanium(IV) complexes whose anticancer activity and putative molecular targets have been interrogated. Potent antitumor activity was demonstrated against cancer cell lines derived from breast, colorectal, and pancreatic carcinomas. Anticancer activity and cancer selectivity superior to those of cisplatin are indicated. Treatment of carcinoma cells with titanium complexes **3b**–**c** perturbs intracellular signaling cascades that generate intracellular ROS and arrest the cell cycle at G2/M phases, evoking DNA double strand break damage, as indicated by γ-H2AX foci, leading to downregulation of antiapoptotic survival proteins BCl-2 and Mcl-1, ultimately triggering apoptotic cell death. SWATH proteomics, subsequent MAP kinase arrays, and western blot identified putative protein targets pertinent to cell cycle regulation and tumorigenesis, indicating mechanisms of antitumor activity that involve MAPK signal disruption. Indeed, oncogenic mutations (e.g., *KRAS* and *BRAF*) within the MAPK network are involved in pathogenesis of a significant number of human tumors. These observations are consistent with Shpilt et al. (2023)^49^ and Pesch et al. (2016)^50^ whose Ti complexes possess a non-DNA mechanism of action, evoking G2/M cell cycle arrest, causing ER stress, ROS, mitochondrial disruption, and apoptosis. Allison et al. recently (2021)^51^ demonstrated selective inhibition of multiple kinases by metal (Zn and Cu) complexes in human carcinoma cell lines. Overall, a picture emerges that titanium(IV) anticancer agents evoke responses in multiple, highly conserved, cellular processes that significantly limits the evolution of resistant cancer cell types. Thus, research into Ti(IV) complexes for treatment of intractable malignancies is worthy of continued development, to progress full elucidation of molecular/cellular mechanisms, preclinical biodistribution, pharmacokinetics, tolerability, and efficacy studies, and eventual clinical evaluation.

## Experimental Section

### Chemical Synthesis

Reactions were carried out under appropriate conditions using commercial reagents of ≥98% purity. Solvents were dried (4A molecular sieves) when appropriate. TLC analyses were performed on foil-backed plates coated with Merck silica gel 60 F_254_. Ultraviolet light and basic aqueous potassium permanganate were used to visualize the plates. Liquid chromatography was performed using forced flow (flash column) techniques with the solvent systems indicated. The stationary phase used was silica gel 60 (220–240 mesh) supplied by Fluorochem. Fourier-transform infrared spectra were recorded on a Bruker Alpha Platinum spectrometer. Nuclear magnetic resonance spectra were recorded on Bruker AV(III)400 (400.1 MHz), Bruker AV400 (400.1 MHz), Bruker Ascend 400 (400.1 MHz), or Bruker Ascend 500 (500.1 MHz) spectrometers at ambient temperature (unless otherwise stated). Chemical shifts are quoted in parts per million (ppm). Coupling constants (*J*) are quoted in hertz. Couplings are written using the following abbreviations: br (broad), s (singlet), d (doublet), t (triplet), q (quartet), m (multiplet), and app (apparent). Carbon NMR multiplicities and connectivities were assigned using DEPT and the relevant 2D NMR experiments. Mass spectrometry was performed using a VG Micromass AutoSpec spectrometer (EI) or Bruker MicroTOF (ESI) instrument as noted. Theoretical HRMS molecular weights were taken from the spectrometer output file, and for HRMS analyses, deviations from expected values (σ) are given in ppm. Melting points were measured on a Gallenkamp melting point apparatus and are uncorrected. Liquid chromatography–mass spectrometry (LCMS) analysis was performed by using an Agilent 1260 Infinity HPLC with a 6120 Quadrupole mass spectrometer with a multimode source. Chromatography conditions: XBridge C18, 3.5 μm, 2.1 mm × 30 mm column. Mobile phase A: 0.1% ammonia in water; mobile phase B: acetonitrile. Flow rate: 0.8 mL/min in a gradient of 5–95% mobile phase B over 3.5 min, with UV detection at 210–400 nm, reported at 254 nm. Column temperature was 40 °C. Data on X-ray diffraction were gathered via the University of Nottingham, X-ray Crystallography Service. Appropriate single crystals were selected and submerged in an inert oil. After that, the crystal was fixed to a glass capillary and fastened to a goniometer head. Data were collected on a Bruker X8 Apex or an Agilent Supernova diffractometer using graphite-monochromated Mo–K_α_ radiation (λ = 0.71073 Å) using 1.0° ϕ-rotation frames. The crystal was cooled to 100 K by an Oxford Cryostream low temperature device.

Additional compounds and procedures for this publication are described in the Supporting Information.

#### General Procedure A: Direct Synthesis of Amine Bis(phenolate) Ligands (**1**) or Benzoxazines (**2**)

Typical reactions were conducted on gram scales. The phenols (1 equiv) were dissolved with stirring (10 min) in methanol (ca. 1.6 mL per mmol of phenol used). To the resulting solution, 37% w/w aqueous formaldehyde in water (3 equiv) was added followed by 40% w/w methylamine in water (2 equiv). The resulting mixture was stirred at room temperature for up to 36 h (maximizes yield of benzoxazines **2**) and at 65 °C for 4 h (maximizes yield of ligands **1**). The mixture was then concentrated by the evaporation of the solvent under reduced pressure. The crude product was purified using column chromatography (SiO_2_, EtOAc/cyclohexane 2:1), and the resulting material crystallized from saturated ambient temperature Et_2_O/pentane (1:4) solutions upon cooling to 4 °C. Compound **1a** was available from a literature procedure.^7^

#### 6,6′-((Methylazanediyl)bis(methylene))bis(4-ethyl-2-methoxyphenol) (**1b**)

Colorless solid in 72% yield. mp 46–47 °C; ^1^H NMR (400.1 MHz, CDCl_3_): δ_H_ 6.63 (d, *J* = 1.9 Hz, 2H), 6.54 (d, *J* = 1.9 Hz, 2H), 3.86 (s, 6H), 3.69 (s, 4H), 2.55 (q, *J* = 7.6 Hz, 4H), 2.20 (s, 3H), 1.20 (t, *J* = 7.6 Hz, 6H), the broad phenol OH signals at ca. 8.3 ppm not easily observed due to exchange; ^13^C NMR (101.0 MHz, CDCl_3_): δ_C_ 146.8, 143.5, 134.6, 122.1, 120.7, 109.9, 58.3, 55.7, 40.7, 28.3, 15.7; IR (ATR): 3400, 2970, 1721, 1637, 1494, 1366, 1291, 1194, 1020, 989, 877, 792, 663, 552, 441 cm^–1^, HRMS (ESI): calcd for [M + H]^+^ C_21_H_29_NO_4_, 360.2175; found, 360.2182 (|σ| = 1.9 ppm); Anal. Calcd (%) for C_21_H_29_NO_4_: C, 70.12; H, 8.13; N, 3.90. Found: C, 70.16; H, 8.11; N, 3.91.

#### 6,6′-((Methylazanediyl)bis(methylene))bis(4-allyl-2-methoxyphenol) (**1c**)

Colorless solid in 80% yield. mp 69–70 °C; ^1^H NMR (500.1 MHz, CDCl_3_): δ_H_ 6.61 (d, *J* = 2.0 Hz, 2H), 6.53 (d, *J* = 2.0 Hz, 2H), 5.94 (ddt, *J* = 16.8, 10.0, 6.6 Hz, 2H), 5.09–5.05 (m, 2H) overlapped by 5.06–5.03 (m, 2H), 3.85 (s, 6H), 3.68 (s, 4H), 3.29 (br, d, *J* = 6.6 Hz plus unresolved ^4^*J* coupling, 4H), 2.19 (s, 3H), the broad phenol OH signals at ca. 8.3 ppm not easily observed due to exchange; ^13^C NMR (126.0 MHz, CDCl_3_): δ_c_ 147.2, 144.2, 138.0, 130.6, 122.5, 121.9, 115.5, 110.9, 58.6, 55.9, 41.1, 39.8; IR (ATR): 3396, 2975, 1721, 1637, 1494, 1366, 1291, 1194, 1020, 989, 877, 792, 663, 552, 441 cm^–1^; HRMS (ESI): calcd for [M + H]^+^ C_23_H_29_NO_4_, 384.2174; found, 384.2185 (|σ| = 2.8 ppm); Anal. Calcd (%) for C_23_H_29_NO_4_: C, 74.04; H, 7.66; N, 3.65. Found: C, 74.06; H, 7.66, N, 3.72.

#### 6-Allyl-3-methyl-3,4-dihydro-2*H*-benzo[*e*][1,3]oxazine (**2c**)

Colorless solid in 69% yield. mp 46–47 °C; ^1^H NMR (400.1 MHz, CDCl_3_): δ_H_ 6.56 (d, *J* = 1.9 Hz, 1H), 6.40 (d, *J* = 1.9 Hz, 1H), 5.98 (ddt, *J* = 16.8, 10.0, 6.7 Hz, 1H), 5.11–5.06 (m, 1H) overlapped by 5.07–5.03 (m, 1H), 4.85 (s, 2H), 3.92 (s, 2H), 3.86 (s, 3H), 3.29 (br, d, *J* = 6.7 Hz plus unresolved ^4^*J* coupling, 2H), 2.61 (s, 3H); ^13^C NMR (100.6 MHz, CDCl_3_): δ_c_ 147.6, 141.4, 137.7, 131.8, 120.2, 119.1, 115.8, 109.9, 82.3, 55.9, 51.9, 40.1, 39.9; IR (ATR): 3073, 2972, 2892, 1737, 1637, 1590, 1495, 1349, 1274, 1146, 1096, 993, 921, 840, 738, 688 cm^–1^; HRMS (ESI): calcd for [M + H]^+^ C_13_H_17_NO_2_, 220.1332; found, 220.1356 (|σ| = 10.9 ppm); Anal. Calcd (%) for C_13_H_17_NO_2_: C, 68.14; H, 6.71; N, 3.61. Found: C, 68.16; H, 6.71, N, 3.44. These agree with the only partially previously reported data for (**2c**).^21^

#### General Procedure B: Synthesis of Intermediate Amine Bis(phenolate) Ligands (**1**) from Benzoxazines (**2**)

Benzoxazine (**2**) (1 equiv) and the required phenol (1.2 equiv) were mixed neat and heated at 100 °C for 4 h. Thus, **1c** was also prepared in 80% yield from **2c** and eugenol.

#### General Procedure C: Synthesis of Bis(amine) Tetrakis(phenolate) Titanium(IV) Complexes (**3**)

The bis(phenol) ligand (**1**) (1 equiv) was dissolved with stirring (3 min) in toluene (ca. 4 mL per mmol of bisphenol). To the resulting solution, titanium(IV) isopropoxide (0.6 equiv) was added dropwise, and the mixture left to stir (4 h) at RT under a nitrogen atmosphere. The solvent was then removed by trap–trap distillation (ca. 1–2 mbar, RT) to afford compound (**3**) as an orange solid. The product was crystallized by liquid–liquid diffusion using suitable solvents upon cooling to 4 °C. Compound **3a** was available from a literature procedure.^7^ All biologically tested **3** were >99% pure by CHN analysis.

#### Complex (**3b**)

Orange rhomboidal crystals in 96% yield. mp 140–141 °C; ^1^H NMR (400.1 MHz, CDCl_3_): δ_H_ 6.62 (d, *J* = 2.0 Hz, 2H), 6.55 (d, *J* = 2.0 Hz, 2H), 6.48 (d, *J* = 2.0 Hz, 2H), 6.44 (d, *J* = 2.0 Hz, 2H), 4.91 (d, *J* = 12.8 Hz, 2H), 4.75 (d, *J* = 12.8 Hz, 2H), 3.42 (s, 6H), 3.38 (d, *J* = 12.8 Hz, 2H), 3.31 (d, *J* = 12.8 Hz, 2H), 3.28 (s, 6H), 2.55 (q, *J* = 7.8 Hz, 4H), 2.51 (s, 6H), 2.49 (q, *J* = 7.8 Hz, 4H), 1.23 (t, *J* = 7.6 Hz, 6H), 1.18 (t, *J* = 7.6 Hz, 6H); ^13^C NMR (100.6 MHz, CDCl_3_). δ_C_ 150.9, 150.6, 146.2, 133.6, 133.4, 124.5, 123.6, 120.1, 119.9, 112.0, 111.4, 64.6, 64.3, 55.8, 55.6, 43.8, 28.4, 28.3, 15.9, 15.8; IR (ATR): 2809, 1670, 1545, 1471, 1391, 1256, 1175, 1088, 972, 856, 711, 570, 482 cm^–1^; HRMS (ESI): calcd for [M + H]^+^ C_42_H_54_N_2_O_8_Ti, 763.3432; found, 763.3439 (|σ| = 0.9 ppm); Anal. Calcd (%) for C_42_H_54_N_2_O_8_Ti: C, 66.14, H, 7.14; N, 3.67. Found: C, 66.09; H, 7.16; N, 3.59. This compound could be recrystallized from diethyl ether/pentane 1:4.

#### Complex (**3c**)

Orange rhomboidal crystals in 85% yield. mp 129–130 °C; ^1^H NMR (500.1 MHz, CDCl_3_): δ_H_ δ 6.63 (d, J = 2.0 Hz, 2H), 6.56 (d, J = 2.0 Hz, 2H), 6.48 (d, J = 2.0 Hz, 2H), 6.45 (d, J = 2.0 Hz, 2H), 5.98 (ddt, J = 16.8, 10.0, 6.6 Hz, 2H), 5.94 (ddt, J = 16.8, 10.0, 6.6 Hz, 2H), 5.12–5.01 (m, 8H), 4.90 (d, J = 12.7 Hz, 2H), 4.76 (d, J = 12.7 Hz, 2H), 3.45 (s, 6H), 3.42 (d, J = 12.7 Hz, 2H), 3.34–3.31 (m, 6H) overlapped by 3.32 (s, 6H), 3.29–3.23 (m, 4H), 2.53 (s, 6H); ^13^C NMR (126.0 MHz, CDCl_3_): δ_C_ 151.1, 151.0, 146.4, 138.2, 138.2, 129.4, 129.1, 124.6, 123.7, 120.8, 120.8, 114.9, 114.9, 112.4, 112.0, 64.5, 64.3, 55.7, 55.6, 43.8, 39.9, 39.8, 13.9; IR (ATR): 2998, 2348, 2249, 2181, 2089, 1988, 1738, 1638, 1578, 1482, 1381, 1228, 1147, 988, 834, 763, 680, 583, 486 cm^–1^; HRMS (ESI): calcd for [M + H]^+^ C_46_H_54_N_2_O_8_Ti, 811.3432; found, 811.3449 (|σ| = 2.0 ppm); Anal. Calcd (%) for C_46_H_54_N_2_O_8_Ti: C, 68.14; H, 6.71; N, 3.61. Found: C, 68.16; H, 6.71; N, 3.44. This compound could be recrystallized from diethyl ether/pentane 1:4.

All other details and primary data for compounds of types **1**–**3** are fully described in the Supporting Information.

### Cancer Biology

Full details of all biological studies are given in the Supporting Information. Exemplary annexin-V and determination of γ-H2AX assays are given below.

#### Annexin-V Assay

Cells were seeded in 10 cm diameter Petri dishes with 10 mL of complete medium at a density of 4 × 10^5^ cells The cells were incubated for 24 h to allow cell attachment. Following treatment (72 h; 10 μM) with a test compound, the cells were trypsinized with 300 μL of 1× trypsin–EDTA and pooled in a total of 1 mL of complete growth medium. Afterward, the cells were resuspended in 2 mL of cold medium and decanted into labeled FACS tubes and kept on ice to allow recovery from any damage caused by trypsin. Cells were centrifuged at 1200 rpm (Beckman Coulter Allegro centrifuge) for 5 min at 4 °C. The supernatant was discarded, and the pellet broken up by gently flicking the tube. Cold PBS (2 mL) was added, and the cells were centrifuged as before. The supernatant was discarded, and the pellet broken up by gently flicking the tube. Thereafter, 100 μL of 1× annexin binding buffer and 5 μL of annexin V FITC was added to each tube. The tubes were briefly vortexed and kept at room temperature for 15 min in the dark. Annexin binding buffer (400 μL; 1×) and 10 μL of 50 μg/mL PI solution were added to each tube, which was vortexed and kept for 10 min at room temperature in the dark prior to analysis on the flow cytometer. Samples were analyzed within 1 h of preparation to avoid sample deterioration using a FC500 Beckman Coulter flow cytometer, and 20,000 events were evaluated for each sample. The results obtained were analyzed using WEASEL software.^7,52^

#### Determination of γ-H2AX Foci Perturbation

For γ-H2AX detection of DNA double strand breaks with concurrent cell cycle analysis, cells were seeded in cell culture dishes at densities of 3–5 × 10^5^ cells/dish in 10 mL of medium. Following 72 h treatment, the cells were harvested and pelleted by centrifugation, resuspended and washed (2×) in PBS, pelleted again by centrifugation and fixed in 500 μL of 1% methanol-free formaldehyde in PBS (5 min; room temperature). The cells were permeabilized by adding 500 μL of 0.4% Triton-X-100 in PBS. FBS (1% in PBS; 1 mL) was then added to cells with gentle mixing before incubation at room temperature for 30 min. The cell suspensions were centrifuged and supernatants aspirated. Primary antibody (1° Ab, p-Histone γ-H2AX) solution (200 μL, 1:3333 in 1% FBS) was added to each tube and the samples incubated (1.5 h). PBS (1 mL) was added, and the samples were centrifuged and supernatants discarded. Secondary antibody (2° Ab, Alexa Flour 488 goat secondary antimouse) was introduced (200 μL, 1:750 in 1% FBS) and the samples incubated for 1 h at room temperature before addition of 1 mL of PBS. Samples were centrifuged and supernatant again discarded. The cells were resuspended in PBS containing 300 μL of 50 μg mL^–1^ PI and 0.1 mg mL^–1^ RNaseA. Analyses of cells (20,000 events per experimental sample) by flow cytometry followed a 10 min incubation.^18^

## Supporting Information Available

Complete experimental data for compounds (**1**–**3**), X-ray crystallographic studies, Hirshfeld surface analysis, antiproliferation kinetic rate study, hydrolysis studies of complexes **3,** estimates of cellular Ti-uptake, MTT assay, cell counting assay, clonogenic assay, cell cycle assay, annexin V assay, determination of γ-H2AX foci perturbation, caspase-3/7 activity assay, confocal microscopy, relative protein phosphorylation, western blot, and detection of reactive oxygen species (PDF)
Proteomic analysis (XLSX)
Kinetics of cell growth in presence of agents (XLSX)
Video showing growth of HCT-116 in the presence of **3c** (MP4)
Video showing growth of HCT-116 in the absence of **3c** (MP4)
Video showing growth of MCF-7 in the presence of **3c** (MP4)
Video showing growth of MCF-7 in the absence of **3c** (MP4)
URLs for accessing cif files (PDF)
GI_50_ values for agents (CSV)

## Abbreviations

Bcl-1: B-cell lymphoma 2
Cdc: cell division control protein
CDK: cyclin-dependent kinase
*C* Log *P*: calculated partition coefficient
DIA: data-independent acquisition
DMSO: dimethyl sulfoxide
ER: endoplasmic reticulum
FITC: fluorescein isothiocyanate
G1: gap 1 cell cycle phase
G2: gap2 cell cycle phase
GI_50_: concentration that inhibits cell growth by 50%
GPCR: G protein-coupled receptor
h: hour
γ-H2AX: phosphorylated (gamma) histone H2AX
Mcl-1: myeloid cell leukemia-1
MTT: (3-(4,5-dimethylthiazolyl-2)-2,5-diphenyltetrazolium bromide)
PABPC: poly(A) binding protein cytoplasmic 1
PARP-1: poly(ADP-ribose) polymerase-1
PI: propidium iodide
M: mitotic cell cycle phase
NQ01: NAD(P)H dehydrogenase (quinone 1)
RAS: rat sarcoma
RTK: receptor tyrosine kinase
S: DNA synthesis cell cycle phase
SD: standard deviation
SOS: son of sevenless
SWATH: sequential window acquisition of all theoretical fragment ion spectra
UV–vis: ultraviolet–visible
