# Supplementary material for: Probing the Mechanism of Action of Bis(phenolato) Amine (ONO Donor Set) Titanium(IV) Anticancer Agents
Source: J Med Chem. 2024 Feb 8;67(4):2732–44. doi: 10.1021/acs.jmedchem.3c01874 (PMC10895680; doi:10.1021/acs.jmedchem.3c01874)
Supplement: Supplementary file 1 — jm3c01874_si_001.pdf [file jm3c01874_si_001.pdf]

Probing the Mechanism of Action of *bis*-Phenolato Amine (ONO Donor Set) Titanium(IV) Anti-Cancer Agents

Mustapha Musa,[1,2] Mohammed Abid,[1,2,3] Tracey D. Bradshaw,[2]* David J. Boocock,[4] Clare Coveney,[4] Stephen P. Argent,[5] Simon Woodward[1]

[1] *GSK Carbon Neutral Laboratories for Sustainable Chemistry, University of Nottingham, Triumph Road, Nottingham, NG7 2TU, United Kingdom.*
[2] *Biodiscovery Institute, University of Nottingham, University park, NG7 2RD, United Kingdom.*
[3] *Department of Chemistry, College of Science, University of Anbar, Western side of Ramadi City, Anbarshire, Iraq.*
[4] *John van Geest Cancer Research Centre, Nottingham Trent University, College Drive, Nottingham NG11 8NS, United Kingdom.*
[5] *School of Chemistry, University Park, Nottingham NG7 2RD, United Kingdom.*
*corresponding author tracey.bradshaw@nottingham.ac.uk

■ Supporting Information

Contents *Page*

1. Complete experimental data for compounds (**1-3**) S2
1.1 ^{1}H and ^{13}C NMR spectra for compounds (**1-3**) [Figures S1-S46] S8
2. X-ray crystallographic studies [Figures S47-S54, Table S1] S55
*'Cif Files for (**3b**) (**3c**), (**3i**), (**3k**)' (CIF)*
2.1 Hirshfeld Surface Analysis [Figures S55-S58] S59
3. Biology methods S61
3.1 Cell culture S61
3.2 Anti-proliferation kinetic rate study (cellular Ti-uptake) [Figure S59, Table S2] S61
'Kinetics-Cell-Growth-in-presence-of-2' (Excel)
3.3 Hydrolysis studies of complexes **3** [Figure S60-S61] S63
3.4 Literature studies allowing estimates of cellular Ti-uptake [Figure S62, Table S3] S65
3.5 MTT assay [Figure S63, Table S4] S66
3.6 Visualization of cellular growth and inhibition [Figure S64] S70
'Movies S1-S4' (.mp4)
3.7 Cell counting assay [Figure S65] S70
3.8 Clonogenic assay [Figures S66-S67] S73
3.9 Cell cycle assay [Figures S68-S69] S77
3.10 Annexin V assay [Figure S70] S79
3.11 Determination of γ-H2AX foci perturbation [Figure S71] S80
3.12 Caspase-3/7 activity assay S80
3.13 Confocal microscopy [Figure S72] S80
3.14 Relative protein phosphorylation [Figure S73] S81
3.15 Western blot [Figure S74-S75] S85
3.16 Proteomics S88
'Proteomics' (Excel)
3.17 Detection of reactive oxygen species [Figure S76] S83

■ 1. Complete experimental data for compounds (1-3)

Compounds were prepared by General Procedures A-C as outlined in the main paper. Compounds (**1a**) and (**3a**) were prepared by the procedure of ref 7 cited in the main paper, affording (**1a**) and (**3a**) in 62% and 88% yields respectively and with the expected data.[1]

*6,6'-((Methylazanediyl)bis(methylene))bis(4-ethyl-2-methoxyphenol) (**1b**).* Synthesized according to General Procedure A, using 4-ethyl-2-methoxyphenol (2.00 g, 13.1 mmol), 37% w/w aqueous formaldehyde in water (3.0 mL, 39 mmol) 40% w/w methylamine in water (2.3 mL, 26 mmol) and MeOH (10 mL) as the solvent to afford a colorless solid in 72% yield (1.71 g, 4.76 mmol). Data in main paper.

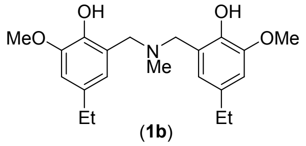

(**1b**)

*6,6'-((Methylazanediyl)bis(methylene))bis(4-allyl-2-methoxyphenol (**1c**)*. Synthesized according to General Procedure B, eugenol (328 mg, 2.00 mmol) and benzoxazine (375 mg, 1.71 mmol) to afford a colorless solid in 80% yield (300 mg, 1.36 mmol). Compound (**1c**) could also be synthesized by General Procedure A in similar yield. Data in main paper.

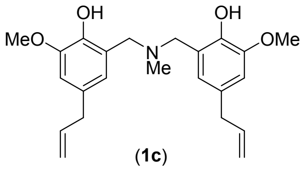

(**1c**)

*6,6'-((Methylazanediyl)bis(methylene))bis(2-methoxy-4-n-propylphenol) (**1d**).* Synthesized according to General Procedure A, using 2-methoxy-4-*n*-propylphenol (1.00 g, 6.00 mmol), 37% w/w aqueous formaldehyde in water (1.4 mL, 18 mmol) 40% w/w methylamine in water (1.1 mL, 12 mmol) and MeOH (10 mL) as the solvent to afford a colorless solid in 50% yield (0.58 g, 1.49 mmol). M.p. 40–41 °C; ^{1}H NMR (400.1 MHz, $CDCl_3$): δ_H 6.61 (d, *J* = 1.9 Hz, 2H), 6.52 (d, *J* = 1.9 Hz, 2H), 3.85 (s, 6H), 3.68 (s, 4H), 2.48 (t, *J* = 7.1 Hz, 4H), 2.19 (s, 3H), 1.76 – 1.63 (m, 4H), 0.92 (t, *J* = 7.3 Hz, 6H), phenol OH signals not observed due to exchange; ^{13}C NMR (100.6 MHz, $CDCl_3$): δ_c 147.0, 143.8, 133.4, 122.3, 121.6, 110.8, 58.7, 56.0, 41.0, 37.9, 25.0, 14.9; ν_{max} (ATR): 3371, 2950, 2848, 1605, 1497, 1418, 1365, 1236, 1210, 1194, 1121, 1086, 986, 955, 922, 875, 833, 787, 757, 592, 566, 553 cm^{-1}; HRMS (ESI) Calcd for $[M+H]^+$ $C_{23}H_{33}NO_4$ 388.2488, found 388.2496 (|σ| = 2.0 ppm); Anal Calcd (%) for $C_{23}H_{33}NO_4$: C, 71.29; H, 8.59; N, 3.61; found C, 71.32; H, 8.60; N, 3.61.

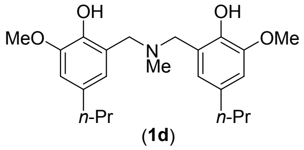

(**1d**)

*5,5'-((Methylazanediyl)bis(methylene))bis(4-hydroxy-3-methoxybenzaldehyde) (**1e**).* Synthesized according to General Procedure A, using vanillin (2.00 g, 13.1 mmol) and 37% w/w aqueous formaldehyde in water (3.0 mL, 39 mmol) 40% w/w methylamine in water (2.3 mL, 26 mmol) and MeOH (10 mL) as the solvent to afford orange powder 72% yield (1.70 g, 4.73 mmol). M.p. 42–43 °C; ^{1}H NMR (500.1 MHz, $CDCl_3$): δ_H 9.80 (s, 2H), 7.35 (d, *J* = 1.8 Hz, 2H), 7.27 (d, *J* = 1.8 Hz, 2H), 3.94 (s, 6H), 3.84 (s, 4H), 2.25 (s, 3H), phenol OH signals not observed due to exchange; ^{13}C NMR (126.0 MHz, $CDCl_3$): δ_C 191.0, 152.5, 148.2, 128.9, 127.3, 122.3, 109.6, 58.5, 56.4, 41.1; ν_{max} (ATR): 3367, 2975, 1721, 1637, 1494, 1366, 1291, 1194, 1020, 989, 877, 792, 663, 552, 441 cm^{-1}; HRMS (ESI) Calcd for $[M+H]^+$ $C_{19}H_{21}NO_6$ 360.1447, found 360.1459 (|σ| = 3.3 ppm). This intermediate ligand could only be brought to ca. 96% purity (based on CHN analyses). Subsequent reactivity to anti-cancer agent (**3e**) is unaffected and that complex is attained analytically pure using (**1d**) of this quality.

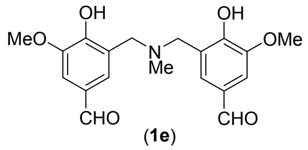

(**1e**)

*6,6'-((Methylazanediyl)bis(methylene))bis(4-fluoro-2-methoxyphenol) (**1f**).* Synthesized according to General Procedure A, using 4-flouro-2-methoxyphenol (200 mg, 1.40 mmol) and 37% w/w aqueous formaldehyde in water (313 µL, 4.20 mmol) 40% w/w methylamine in water (242 µL, 2.80 mmol) in methanol (5 mL) as the solvent to afford a colorless solid in 51% yield (121 mg, 0.35 mmol). M.p. 62–63 °C; ^{1}H NMR (400.1 MHz, $CDCl_3$): δ_H ca. 8.3 (vbr, 2H), 6.56 (dd, J = 2.0, J_{HF} = 8.8 Hz, 2H), 6.44 (dd, J = 2.0, J_{HF} = 8.8 Hz, 2H), 3.85 (s, 6H), 3.68 (s, 4H), 2.20 (s, 3H), phenol OH signals highly broadened due to exchange; ^{13}C NMR (100.6 MHz, $CDCl_3$): δ_C 157.5 (d, J_{CF} = 237.3 Hz), 148.0 (d, J_{CF} = 10.0 Hz), 142.3 (d, J_{CF} = 2.9 Hz), 123.0 (d, J_{CF} = 8.4 Hz), 107.8 (d, J_{CF} = 22.8 Hz), 99.5 (d, J_{CF} = 27.2 Hz), 57.8 (d, J_{CF} = 1.9 Hz), 56.5, 41.3; ^{19}F NMR (376.5 MHz, $CDCl_3$): δ_F –123.0; ν_{max} (ATR): 3410, 3056, 2900, 2861, 1462, 1433, 1200, 1189, 1120, 1030, 1000, 872, 766, 720, 552, 441 cm^{-1}; HRMS (ESI) Calcd for $[M+H]^+$ $C_{17}H_{19}F_2NO_4$ 340.1360, found 340.1365 (|σ| = 1.4 ppm). We could not attain satisfactory elemental analyses on (**1f**); HF generation on combustion apparently causes analysis issues. Samples of ligand (**1f**) were pure/homogeneous by ^{1}H, ^{13}C and ^{19}F NMR spectroscopy. Subsequent reactivity to anti-cancer agent (**3f**) is unaffected and that complex is attained analytically pure using (**1f**) of this quality.

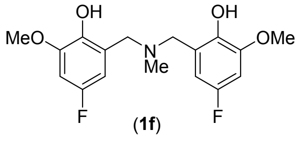

(**1f**)

*6,6'-((Ethylazanediyl)bis(methylene))bis(4-ethyl-2-methoxyphenol) (**1g**).* Synthesized according to General Procedure A using 2-methoxy-4-ethylphenol (1.00 g, 6.57 mmol) and 37% w/w aqueous formaldehyde in water (1.5 mL, 20 mmol) 70% w/w ethylamine in water (1.25 mL, 13.1 mmol) and methanol (10 mL) as the solvent to afford a colorless solid in 51% yield (630 mg, 1.68 mmol). M.p. 43–44 °C; ^{1}H NMR (400.1 MHz, $CDCl_3$): δ_H 6.60 (d, J = 1.9 Hz, 2H), 6.44 (d, J = 1.9 Hz, 2H), 4.96 (s, 2H), 4.01 (s, 4H), 3.89 (s, 6H), 2.86 (q, J = 7.2 Hz, 4H), 2.63 (t, J = 7.2 Hz, 2H), 2.53 (t, J = 7.2 Hz, 3H), 1.21 (q, J = 7.4 Hz, 6H), phenol OH signals not observed due to exchange; ^{13}C NMR (100.6 MHz, $CDCl_3$): δ_C 147.4, 141.4, 135.9, 120.3, 118.1, 109.2, 55.8, 49.8, 45.4, 28.6, 15.9, 13.4; ν_{max} (ATR): 3341, 3118, 2806, 1605, 1460, 1418, 1365, 1297, 1194, 1156, 1086, 966, 955, 875, 841, 787, 592, 576 cm^{-1}; HRMS (ESI) Calcd for $[M+H]^+$ $C_{22}H_{31}NO_4$ 374.2331, found 374.2334 (|σ| = 0.8 ppm). This ligand could only be brought to ca. 95% purity (based on CHN analyses). Subsequent reactivity to anti-cancer agent (**3g**) is unaffected and that complex is attained analytically pure using (**1g**) of this quality.

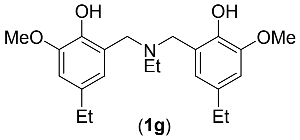

(**1g**)

*6,6'-((Ethylazanediyl)bis(methylene))bis(4-allyl-2-methoxyphenol) (**1h**).* Synthesized according to General Procedure A, using eugenol (1.00 g, 6.09 mmol) and 37% w/w aqueous formaldehyde in water (1.4 mL, 18 mmol) 70% w/w ethylamine in water (1.15 mL, 12.0 mmol) and methanol (10 mL) as the solvent to afford a colorless solid in 35% yield (429 mg, 1.08 mmol). M.p. 43–44 °C; ^{1}H NMR (400.1 MHz, $CDCl_3$): δ_H 6.60 (br, d, J = 1.9 Hz, 2H), 6.54 (br, d, J = 1.9 Hz, 2H), 5.98 (ddt, J = 16.8, 10.0, 6.7 Hz, 2H), 5.10 – 5.04 (m, 2H), overlapped by 5.06 – 5.03 (m, 2H), 3.84 (s, 6H), 3.72 (s, 4H), 3.28 (br, d, J = 6.7, Hz, 4H), 2.60 (q, J = 7.1 Hz, 2H), 1.14 (t, J = 7.1 Hz, 3H), phenol OH signals not observed due to exchange; ^{13}C NMR (100.6 MHz, $CDCl_3$): δ_H 147.6, 144.6, 138.3, 130.9, 122.3 br, 122.0, 115.9, 111.2, 56.3, 54.6, 47.1, 40.3, 11.2; ν_{max} (ATR): 3396, 2974, 1722, 1637, 1494, 1366, 1291, 1194, 1020, 989, 877, 792, 663, 552, 441 cm^{-1}; HRMS (ESI) Calcd for $[M+H]^+$ $C_{24}H_{31}NO_4$ 398.2331, found 398.2338 (|σ| = 1.7 ppm). This intermediate ligand could only be brought to ca. 95% purity (based on CHN analyses). Subsequent reactivity to anti-cancer agent (**3h**) is unaffected and that complex is attained analytically pure using (**1h**) of this quality.

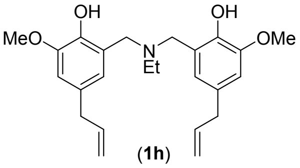

(**1h**)

*6,6'-((Ethylazanediyl)bis(methylene))bis(2-methoxy-4-n-propylphenol) (**1i**).* Synthesized according to General Procedure A, using 2-methoxy-4-propylphenol (1.00 g, 6.02 mmol)

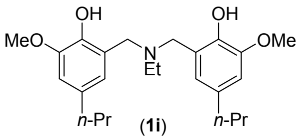

(**1i**)

and 37% w/w aqueous formaldehyde in water (1.40 mL, 18 mmol) 70% w/w ethylamine in water (1.15 mL, 12.0 mmol) and methanol (10 mL) as the solvent to afford a colorless solid in 60% yield (730 mg, 1.82 mmol). M.p. 42–47 °C; ^{1}H NMR (400.1 MHz, CDCl$_3$): δ_H 6.64 (d, *J* = 1.8 Hz, 2H), 6.41 (d, *J* = 1.8 Hz, 2H), 4.96 (app. s, 2H), 4.00 (s, 4H), 3.87 (s, 6H), 2.85 (q, *J* = 7.2 Hz, 2H), 2.54 (t, *J* = 6.8 Hz, 4H), 1.68 – 1.57 (m, 4H), 1.19 (t, *J* = 7.2 Hz, 3H), 0.96 (t, *J* = 7.3 Hz, 6H),), phenol OH signals not observed due to exchange; ^{13}C NMR (100.6 MHz, CDCl$_3$): δ_C 147.8, 141.8, 134.8, 120.7, 119.2, 110.1, 82.5, 50.2, 45.9, 38.3, 25.1, 14.3, 13.8; ν_{max} (ATR): 3333, 3000, 2913, 2848, 1605, 1497, 1365, 1236, 1136, 1086, 992 922, 862, 777, 592, cm^{-1} HRMS (ESI) Calcd for [M+H]$^+$ $C_{24}H_{35}NO_4$ 402.2644, found 402.2652 (|σ| = 1.9 ppm). This intermediate ligand could only be brought to ca. 95% purity (based on CHN analyses), unreacted starting phenol removal is problematic in this case. Subsequent reactivity to anti-cancer agent (**3i**) is unaffected and that complex is attained analytically pure using (**1i**) of this quality.

*6,6'-((Methylazanediyl)bis(methylene))bis(2-methoxy-4 (morpholinomethyl)phenol) (**1j**).* To a solution containing *bis*(phenolate) ligand (**1e**) (200 mg, 0.56 mmol) and morpholine (158 µL, 1.83 mmol) in MeOH (2 mL), picoline borane (266 mg, 2.49 mmol) was added and the resultant mixture stirred overnight at room temperature. The reaction was worked up by evaporating the solvent *in vacuo*. The crude product was dissolved in dioxane (2 mL) and treated with concentrated hydrochloric acid (12 M, 0.5 mL) followed by stirring at ambient temperature (1 h). The resulting mixture was washed with saturated $NaHCO_3$ (5 mL), the organic layer dried over anhydrous Na_2SO_4 and the solvent removed to afford pink powder. Chromatography (SiO_2, EtOAc/pentane 2:1 with 1 mL of 10% ammonia in methanol per 10 mL of total eluent) afforded a colorless solid in 63% yield (168.0 mg, 0.35 mmol) M.p. 80–82 °C; ^{1}H NMR (400.1 MHz, CDCl$_3$): δ_H 6.73 (br s, 2H), 6.44 (br s, 2H), 3.86 (s, 4H), 3.82 (s, 6H), 3.73 (br, s, 8H), 3.69 (s, 4H), 3.34 (s, 2H), 2.48 (br, s, 8H), 2.20 (s, 3H) phenol OH signals ca. 3.3 due to internal morpholino protonation; ^{13}C NMR (100.6 MHz, CDCl$_3$): δ_C 149.8, 142.4, 128.7, 119.1, 116.6, 112.5, 67.1, 63.4, 60.3, 58.6, 56.1, 53.8, 50.4, 44.0, the spectrum complicated by to internal morpholino protonation; ν_{max} (ATR): 3332, 2932, 2804, 1588, 1497, 1366, 1264, 1114, 1055, 911, 864, 769, 663, 552, 441 cm^{-1}; HRMS (ESI) Calcd for [M+H]$^+$ $C_{27}H_{39}N_3O_6$ 502.2918, found 502.2924 (|σ| = 1.1 ppm); Anal Calcd (%) for $C_{27}H_{39}N_3O_6$. C, 64.65; H, 7.84; N, 8.38. found C, 64.70; H, 7.84; N, 8.39. Subsequent exchange behaviour of intermediate ligand (**1j**) does not affect reactivity to anti-cancer agent (**3j**) which is attained analytically pure.

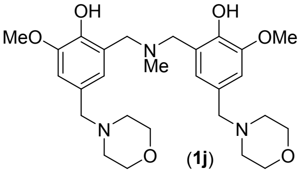

(**1j**)

*4-Allyl-2-(((2-hydroxy-3-methoxy-5-methylbenzyl)(methyl)amino)methyl)-6-methoxyphenol (**1k**).* Synthesised according to General Procedure B, using 2-methoxy-4-methylphenol (302 mg, 2.19 mmol) and benzoxazine (**2c**) (400 mg 1.82 mmol) to afford a colourless solid in 53% yield (345 mg, 0.97 mmol). M.p. 30–31 °C; ^{1}H NMR (500.1 MHz, CDCl$_3$): δ_H 6.61 (d, *J* = 2.0 Hz, 1H), 6.605 (d, *J* = 2.0 Hz, 1H), 6.53 – 6.51 (m, 2H), 5.91 (ddt, *J* = 16.8, 10.0, 6.7 Hz, 1H), 5.09 – 5.04 (m, 1H) overlapped by 5.05 – 5.03 (m, 1H), 3.85 (s, 3H), 3.84 (s, 3H), 3.69 (s, 2H), 3.67 (s, 2H) 3.29 (d, *J* = 6.7 Hz plus unresolved ^{4}J coupling, 2H), 2.26 (s, 3H), 2.19 (br, s, 3H), the broad phenol OH signals at ca. 8.4 ppm were not easily observed due to exchange; ^{13}C NMR (126.0 MHz, CDCl$_3$): δ_C 147.3, 147.0, 144.3, 143.5, 138.0, 130.5, 128.3, 122.5, 122.4, 122.3, 122.2, 121.8, 121.7, 115.5, 111.4, 110.9, 58.7, 56.0, 40.9, 40.0, 21.1; ν_{max} (ATR): 3394, 3009, 2979, 2806, 1234, 1118, 1083, 986, 954, 903, 833, 724, 688, 559, 465 cm^{-1}; HRMS (ESI) Calcd for [M+H]$^+$ $C_{21}H_{27}NO_4$ 358.2013, found 358.2030 (|σ| = 4.7 ppm); Anal Calcd (%) for $C_{21}H_{27}NO_4$ C, 70.56; H, 7.61; N, 3.92; found C, 72.62; H, 7.63, N, 3.89.

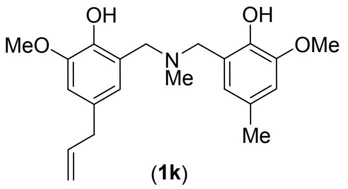

(**1k**)

8-Methoxy-3-methyl-3,4-dihydro-2H-benzo[e][1,3]oxazine-6-carbaldehyde (**2e**). Synthesized according to General Procedure A, using vanillin (2.00 g, 13.1 mmol), 37% w/w aqueous formaldehyde in water (2.9 mL, 39 mmol), 40% w/w methylamine in water (2.3 mL, 26 mmol) and MeOH (10 mL) as the solvent to afford a colorless solid in 29% yield (0.80 g, 3.86 mmol). The mass balance of the reaction is mostly recovered vanillin; allowing for this give yields of (**2e**) of 55-65% (brsm). M.p. 38–39 °C; ^{1}H NMR (500.1 MHz, $CDCl_3$): δ_H 9.81 (s, 1H), 7.30 (d, *J* = 1.8 Hz, 1H), 7.15 (d, *J* = 1.8 Hz plus unresolved ^{4}J coupling, 1H), 4.99 (s, 2H), 4.04 (s, 2H), 3.95 (s, 3H), 2.64 (s, 3H); ^{13}C NMR (126.0 MHz, $CDCl_3$): δ_C 190.7, 148.7, 148.3, 129.0, 124.7, 107.8, 85.0, 55.9, 51.3, 39.7, we could not detect the *ipso*-1-OC carbon (δ_C calc 156 ppm) in our maximum viable accumulation times; ν_{max} (ATR): 3023, 2972, 2892, 1737, 1637, 1590, 1495, 1349, 1274, 1146, 1096, 993, 921, 840, 738, 688 cm^{-1}; HRMS (ESI) Calcd for $[M+H]^+$ $C_{11}H_{13}NO_3$ 208.0968, found 208.0974 ($|\sigma|$ = 2.8 ppm). The compound was used promptly, as attained, due to its reactive formyl group.

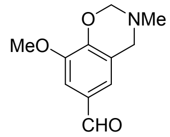

(**2e**)

Bis((2,2'-((methylimino-N)bis(methylene))bis(4-ethyl-6-methoxyphenolato-O)))titanium(IV) (**3b**). Synthesized according to General Procedure C, using ligand (**1b**) (200 mg, 0.50 mmol), titanium(IV) isopropoxide (94 mg, 0.30 mmol) and toluene (2 mL) as a solvent to afford orange rhomboidal crystals in 96% yield (190 mg, 0.24 mmol). Data in main paper.

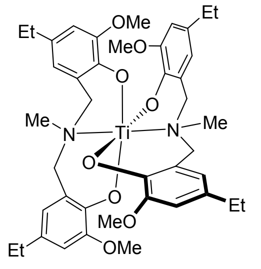

(**3b**)

Bis((2,2'-((methylimino-N)bis(methylene))bis(4-allyl-6-methoxyphenolato-O)))titanium(IV) (**3c**). Synthesized according to General Procedure C, using ligand (**1c**) (120 mg, 0.31 mmol), titanium(IV) isopropoxide (51 mg, 0.18 mmol) and toluene (2 mL) as a solvent to afford orange rhomboidal crystals in 81% yield (102 mg, 0.13 mmol). Data in main paper.

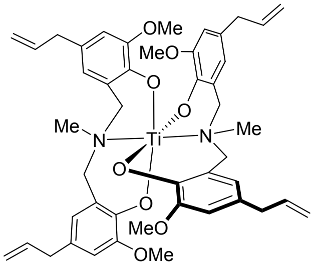

(**3c**)

Bis((2,2'-((methylimino-N)bis(methylene))bis(4-propyl-6-methoxyphenolato-O)))titanium(IV) (**3d**). Synthesized according to General Procedure C, using ligand (**1d**) (200 mg, 0.52 mmol), titanium(IV) isopropoxide (88 mg, 0.31 mmol) and toluene (2 mL) as a solvent to afford orange monoclinic crystals in 85% yield (180 mg, 0.22 mmol). M.p. 100–101 °C; ^{1}H NMR (400.1 MHz, $CDCl_3$): δ_H 6.59 (d, *J* = 1.9 Hz, 2H), 6.53 (d, *J* = 1.9 Hz, 2H), 6.45 (d, *J* = 1.9 Hz, 2H), 6.42 (d, *J* = 1.9 Hz, 2H), 4.92 (d, *J* = 12.7 Hz, 2H), 4.75 (d, *J* = 12.7 Hz, 2H), 3.42 (s, 6H), 3.37 (d, *J* = 12.7 Hz, 2H), 3.30 (d, *J* = 12.7 Hz, 2H), 3.28 (s, 6H), 2.49 (s, 6H) overlapped by 2.51 – 2.41 (m, 6H), 1.60 – 1.49 (m, 10H), 0.93 (t, *J* = 7.3 Hz, 6H), 0.90 (t, *J* = 7.3 Hz, 6H); ^{13}C NMR (100.6 MHz, $CDCl_3$): δ_c 151.3, 151.1, 146.5 (br 2C), 132.4, 132.2, 124.8, 123.8, 121.1, 121.0, 112.9, 112.4, 64.9, 64.7, 56.1, 56.0, 44.0, 38.0, 37.9, 25.1, 25.0, 14.0, 13.95; ν_{max} (ATR): 2900, 1530, 1482, 1388, 1224, 1150, 1092, 979, 837, 730, 550, 481 cm^{-1}; HRMS (ESI) Calcd for $[M+H]^+$ $C_{46}H_{62}N_2O_8Ti$ is

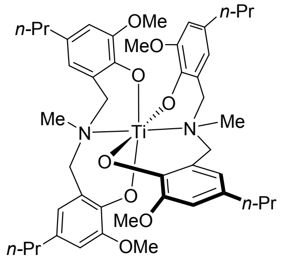

(**3d**)

819.4068, found 819.4064 (|σ| = 0.4 ppm); Anal Calcd (%) for $C_{46}H_{62}N_2O_8Ti$: C, 67.47, H, 7.63; N, 3.42; found C, 67.44; H, 7.65; N, 3.37. This compound could be recrystallized from (diethyl ether/pentane 1:4).

Bis((2,2′-((methylimino-N)bis(methylene))bis(4-formyl-6-methoxyphenolato-O)))titanium(IV) (**3e**).

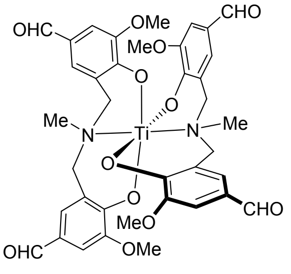

(**3e**)

Synthesized according to General Procedure C, using ligand (**1e**) (100 mg, 0.28 mmol), titanium(IV) isopropoxide (45 mg, 0.16 mmol) and toluene (2 mL) as a solvent to afford orange fluffy needles in 79% yield (85 mg, 0.11 mmol). M.p. 126 –127 °C; ^{1}H NMR (400.1 MHz, $CDCl_3$) δ_H 9.84 (s, 2H), 9.78 (s, 2H), 7.44 (d, *J* = 1.8 Hz, 2H), 7.36 (d, *J* = 1.8 Hz, 2H), 7.25 (d, *J* = 1.8 Hz, 2H), 7.22 (d, *J* = 1.8 Hz, 2H), 4.89 (d, *J* = 12.7 Hz, 2H), 4.80 (d, *J* = 12.7 Hz, 2H), 3.66 (d, *J* = 12.7 Hz, 2H), 3.62 (s, 6H), 3.59 (d, *J* = 12.7 Hz, 2H), 3.38 (s, 6H), 2.59 (s, 6H); ^{13}C NMR (100.6 MHz, $CDCl_3$): δ_C 190.5, 190.4, 158.0, 157.4, 147.5, 147.5, 128.6, 128.3, 126.0, 124.3, 123.8, 110.4, 110.3, 63.9, 55.7, 55.3, 44.2, 30.9; ν_{max} (ATR): 2909, 1710, 1637, 1579, 1484, 1384, 1244, 1152, 1097, 989, 827, 720, 580, 487 cm^{-1}; HRMS (ESI) Calcd for $[M+H]^+$ $C_{38}H_{38}N_2O_{12}Ti$ is 763.1977 found 763.1982 (|σ| = 0.6 ppm); Anal Calcd (%) for $C_{38}H_{38}N_2O_{12}Ti$; C, 59.85; H, 5.02; N, 3.67; found C, 56.81; H, 5.04, N, 3.69. This compound could be recrystallized from dioxane/pentane (1:4).

Bis((2,2′-((methylimino-N)bis(methylene))bis(4-fluoro-6-methoxyphenolato-O)))titanium(IV) ***(3f)***.

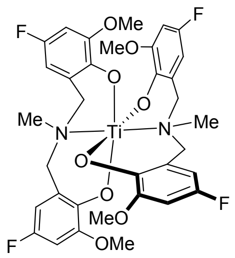

(**3f**)

Synthesized according to General Procedure C, using ligand (**1f**) (100 mg, 0.29 mmol), titanium(IV) isopropoxide (34 mg, 0.12 mmol) and toluene (2 mL) as a solvent to afford orange rhomboidal crystals in 88% yield (92.0 mg, 0.13 mmol). M.p. 152–153 °C; ^{1}H NMR (400.1 MHz, $CDCl_3$): δ_H 6.52 (dd, *J* = 2.9, J_{HF} = 8.6, Hz, 2H), 6.44 (dd, *J* = 2.9, J_{HF} = 8.6, Hz, 2H), 6.41 (dd, *J* = 2.9, J_{HF} = 8.6, Hz, 2H), 6.37 (dd, *J* = 2.9, J_{HF} = 8.6, Hz, 2H), 4.75 (d, *J* = 13.0 Hz, 2H), 4.70 (d, *J* = 13.0 Hz, 2H), 3.46 (s, 6H), 3.44 (d, *J* = 13.0 Hz, 2H), 3.38 (s, 6H), 3.30 (d, *J* = 13.0 Hz, 2H) 2.56 (s, 6H); ^{13}C NMR (100.6 MHz, $CDCl_3$): δ_C 155.9 (d, J_{CF} = 236.8 Hz), 155.1 (d, J_{CF} = 236.5 Hz), 148.55 (d, J_{CF} = 22.7 Hz), 148.5 (d, J_{CF} = 22.7 Hz), 147.4 (d, J_{CF} = 10.4 Hz), 147.35 (d, J_{CF} = 10.4 Hz), 124.2 (d, J_{CF} = 9.4 Hz), 123.2 (d, J_{CF} = 9.3 Hz), 106.4, 106.1, 99.4, 99.2, 64.3, 64.1, 55.7, 55.6, 44.4, 29.9, J_{CF} coupling vs. diastereotopic carbons were assigned by comparison with known $^{n}J_{CF}$ values; ^{19}F NMR (376.5 MHz, $CDCl_3$): δ_F –126.09; ν_{max} (ATR): 2909, 1637, 1579, 1484, 1384, 1244, 1152, 1097, 989, 827, 720, 580, 487 cm^{-1}; HRMS (ESI) Calcd for $[M+H]^+$ $C_{34}H_{34}F_4N_2O_8Ti$ 723.1804 found 723.1810 (|σ| = 0.8 ppm); Anal Calcd (%) for $C_{34}H_{34}F_4N_2O_8Ti$: C, 56.52; H, 4.74; N, 3.88; found C, 56.56; H, 4.71; N, 3.92. This compound could be recrystallised from (diethyl ether/pentane 1:4).

Bis((2,2′-((ethylimino-N)bis(methylene))bis(4-ethyl-6-methoxyphenolato-O)))titanium(IV) ***(3g)***.

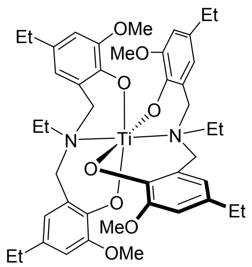

(**3g**)

Synthesized according to General Procedure C, using ligand (**1g**) (200 mg, 0.54 mmol), titanium(IV) isopropoxide (88 mg, 0.31 mmol) and toluene (2 mL) as a solvent to afford orange rhomboidal crystals in 82% yield (172 mg, 0.22 mmol). M.p. 118–119 °C; ^{1}H NMR (400.1 MHz, $CDCl_3$): δ_H 6.66 (d, *J* = 2.0 Hz, 2H), 6.58 (d, *J* = 2.0 Hz, 2H), 6.50 (d, *J* = 2.0 Hz, 2H), 6.49 (d, *J* = 2.0 Hz, 2H), 4.72 (d, *J* = 13.0 Hz, 2H), 4.63 (d, *J* = 13.3 Hz, 2H), 3.70 (d, *J* = 13.0 Hz, 2H), 3.64 (d, *J* = 13.3 Hz, 2H), 3.50 (s, 6H), 3.30 (s, 6H), 3.11 (dq, *J* = 14.3, 7.1 Hz, 2H), 3.01 (dq, *J* = 14.3, 7.1 Hz, 2H), 2.56 (q, *J* = 7.5, Hz, 4H), 2.50 (q, *J* = 7.5, Hz, 4H), 1.22 (t, *J* = 7.5 Hz, 6H), 1.16 (t, *J* = 7.5 Hz, 6H), 0.99 (t, *J* = 7.1 Hz, 6H); ^{13}C NMR (100.6 MHz, $CDCl_3$): δ_C 151.8, 151.6, 146.5, 146.4, 133.9, 133.8, 124.6, 123.9, 120.4, 120.35, 112.6, 112.3, 57.9, 57.8, 56.3, 56.2, 44.3, 28.7, 28.65, 16.3, 16.2, 5.8; ν_{max} (ATR): 2925, 2806, 1605, 1460, 1365, 1297, 1121, 1086, 986, 922, 841,

740, 592, 553 cm^{-1}; HRMS (ESI) Calcd for $[M+H]^+$ $C_{44}H_{58}N_2O_8Ti$ 791.3745, found 791.3750 (|σ| = 0.6 ppm); Anal Calcd (%) for $C_{44}H_{58}N_2O_8Ti$; C, 66.83; H, 7.39; N, 3.54; found C, 66.81; H, 7.42, N, 3.57. This compound could be recrystallised from dioxane/pentane 1:4.

*Bis((2,2'-((ethylimino-N)bis(methylene))bis(4-allyl-6-methoxyphenolato-O)))titanium(IV) (**3h**).* Synthesized according to General Procedure C, using ligand (**1h**) (214 mg, 0.54 mmol), titanium(IV) isopropoxide (88 mg, 0.31 mmol) and toluene (2 mL) as a solvent to afford orange rhomboidal crystals in 78% yield (176 mg, 0.21 mmol). M.p. 130–141 °C; ^1H NMR (400.1 MHz, $CDCl_3$): δ_H 6.64 (d, *J* = 2.0 Hz, 2H), 6.56 (d, *J* = 2.0 Hz, 2H), 6.48 (d, *J* = 2.0 Hz, 2H), 6.47 (d, *J* = 2.0 Hz, 2H), 5.96 (ddt, *J* = 16.8, 10.1, 6.6 Hz, 4H), 5.91 (ddt, *J* = 16.8, 10.1, 6.6 Hz, 4H), 5.09 – 5.07 (m, 8H), 4.68 (d, *J* = 13.0 Hz, 2H), 4.63 (d, *J* = 13.0 Hz, 2H), 3.73 (d, *J* = 13.0 Hz, 2H), 3.64 (d, *J* = 13.0 Hz, 2H), 3.50 (s, 6H), 3.30 (s, 6H) overlapped by 3.30 (d, *J* = 6.6, Hz, 4H), 3.24 (t, *J* = 6.6 Hz plus unresolved ^4J coupling, 4H), 3.11 (dq, *J* = 14.3, 7.0 Hz, 2H), 3.00 (dq, *J* = 14.3, 7.0 Hz, 2H), 0.98 (t, *J* = 7.1 Hz, 6H); ^{13}C NMR (100.6 MHz, $CDCl_3$): δ_C 152.0, 151.8, 146.6, 146.5, 138.6, 138.5, 129.4, 129.35, 124.6, 124.0, 121.2, 121.1, 115.15, 115.1, 112.9, 112.8, 57.9, 57.7, 56.2, 56.0, 44.3, 40.2, 40.0, 5.8; ν_{max} (ATR): 2910, 1647, 1579, 1484, 1384, 1244, 1152, 1097, 989, 827, 720, 580, 480 cm^{-1}; HRMS (ESI) Calcd for $[M+H]^+$ $C_{48}H_{58}N_2O_8Ti$ is 839.3673 found 839.3669 (|σ| = 0.4 ppm); Anal Calcd (%) for $C_{48}H_{58}N_2O_8Ti$: C, 68.73, H, 6.97; N, 3.34; found C, 68.82; H, 6.91; N, 3.36. This compound could be recrystallized from (dioxane/pentane 1:4).

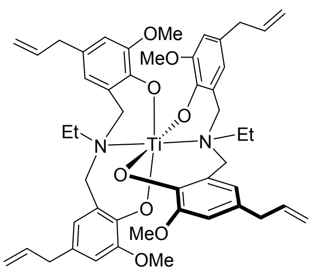

(**3h**)

*Bis((2,2'-((ethylimino-N)bis(methylene))bis(4-n-propyl-6-methoxyphenolato-O)))titanium(IV) (**3i**).* Synthesized according to General Procedure C, using ligand (**1i**) (210 mg, 0.52 mmol), titanium(IV) isopropoxide (88 mg, 0.31 mmol) and toluene (2 mL) as a solvent to afford orange monoclinic crystals in 84% yield (190 mg, 0.22 mmol). M.p. 138–139 °C; ^1H NMR (400.1 MHz, $CDCl_3$): δ_H 6.63 (d, *J* = 2.0 Hz, 2H), 6.56 (d, *J* = 2.0 Hz, 2H), 6.48 (d, *J* = 2.0 Hz, 2H), 6.47 (d, *J* = 2.0 Hz, 2H), 4.74 (d, *J* = 13.0 Hz, 2H), 4.63 (d, *J* = 13.0 Hz, 2H), 3.67 (d, *J* = 13.0 Hz, 2H), 3.63 (d, *J* = 13.0 Hz, 2H), 3.50 (s, 6H), 3.29 (s, 6H), 3.10 (dq, *J* = 14.3, 7.1 Hz, 2H), 2.98 (dq, *J* = 14.3, 7.1 Hz, 2H), 2.55 – 2.39 (m, 4H), 1.66 – 1.49 (m, 4H), 0.97 (t, *J* = 7.1 Hz, 6H), 0.93 (t, *J* = 7.1 Hz, 6H), 0.89 (t, *J* = 7.1 Hz, 6H); ^{13}C NMR (100.6 MHz, $CDCl_3$): δ_C 151.9, 151.7, 146.4, 146.35, 132.2 (2C), 124.4, 123.8, 121.15, 121.1, 113.0, 112.9, 57.9, 57.7, 56.4, 56.1, 44.1, 38.0, 37.9, 25.2, 25.1, 13.95, 13.9, 5.6; ; ν_{max} (ATR): 2929, 1637, 1579, 1484, 1384, 1244, 1152, 1097, 989, 827, 720, 580, 487 cm^{-1}; HRMS (ESI) Calcd for $[M+H]^+$ $C_{48}H_{66}N_2O_8Ti$ is 847.4371, found 847.4381 (|σ| = 1.1 ppm); Anal Calcd (%) for $C_{48}H_{66}N_2O_8Ti$: C, 68.07, H, 7.86; N, 3.31; found C, 68.14; H, 7.92; N, 3.35. This compound could be recrystallized from (diethyl ether/pentane 1:4).

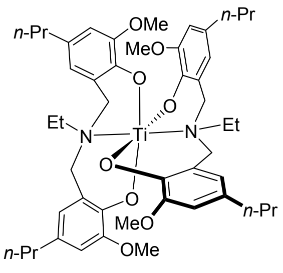

(**3i**)

*Bis((2,2'-((methylimino-N)bis(methylene))bis(4-morpholinomethyl-6-methoxyphenolato-O)))titanium(IV) (**3j**).* Synthesized according to General Procedure C, using ligand (**1j**) (117 mg, 0.23 mmol), titanium(IV) isopropoxide (35 mg, 0.12 mmol) and toluene (1 mL) as a solvent to afford orange orthorhombic crystals in 82% yield (100 mg, 0.096 mmol). M.p. 157–158 °C; ^1H NMR (400.1 MHz, $CDCl_3$): δ_H 6.74 (d, *J* = 1.9 Hz, 2H), 6.67 (d, *J* = 1.9 Hz, 2H), 6.63 (d, *J* = 1.9 Hz, 2H), 6.60 (d, *J* = 1.9 Hz, 2H), 4.84 (d, *J* = 12.7 Hz, 2H), 4.78 (d, *J* = 12.7 Hz, 2H), 3.73 – 3.66 (m, 16H), 3.49 (s, 6H), 3.46 – 3.26 (m, 12H) overlapped by 3.32 (s, 6H), 2.48 (s, 6H), 2.46 – 2.36 (m, 16H); ^{13}C NMR (100.6 MHz, $CDCl_3$): δ_C 152.15, 152.1, 146.6 (2C), 127.4, 127.2, 124.5, 123.8, 122.2, 122.15, 113.2, 113.1, 64.6, 64.5, 63.75, 63.7, 56.1, 55.8, 53.75, 53.7, 14.0, not all of the

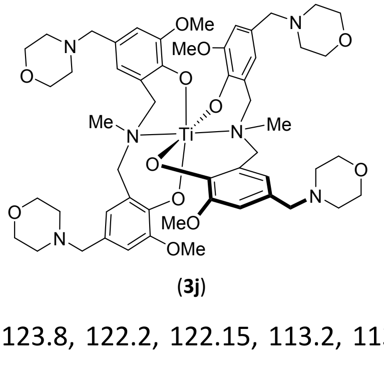

(**3j**)

diastereotopic aliphatic carbons are resolved; ν_{max} (ATR): 2909, 1637, 1579, 1484, 1384, 1244, 1152, 1097, 989, 827, 720, 580, 487 cm^{-1}; HRMS (ESI) Calcd for $[M+2H]^+$ $C_{54}H_{76}N_6O_{12}Ti$ is 1048.4923, found 1048.4927 (|σ| = 6.5 ppm); Anal Calcd (%) for $C_{54}H_{74}N_6O_{12}Ti$: C, 61.94, H, 7.12; N, 8.03; found C, 61.90; H, 7.19; N, 8.09. This compound could be recrystallized, as a powder, from (diethyl ether/pentane 1:4); or by diffusion of pentane into undried CH_2Cl_2, which provided a water/CH_2Cl_2 solvate (see Section 2).

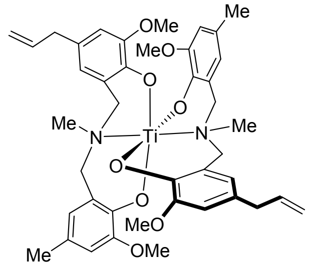

(**3k**)

*Bis((2,2'-((methylimino-N)bis(methylene))bis(4-allyl-4-methyl-6,6-dimethoxyphenolato-O)))titanium(IV) (**3k**).* Synthesized according to General Procedure C, using ligand (**1k**) (500 mg, 1.40 mmol), titanium(IV) isopropoxide (221 mg, 0.78 mmol) and toluene (2 mL) as a solvent to afford orange powder in 87% yield (460 mg, 0.61 mmol). M.p. 105–106 °C; ^1H NMR (500.1 MHz, $CDCl_3$): δ_C 6.60 (d, *J* = 1.9 Hz, 1H), 6.59 (d, *J* ~ 1.9 Hz, 1H), 6.53 (d, *J* ~ 1.9 Hz, 1H), 6.52 (d, *J* ~ 1.9 Hz, 1H), 6.46 (d, *J* ~ 1.9 Hz, 1H), 6.59 (d, *J* ~ 1.9 Hz, 1H), 6.42 (d, *J* ~ 1.9 Hz, 1H), 6.41 (d, *J* ~ 1.9 Hz, 1H), 5.96 (ddt, *J* = 16.7, 9.9, 6.6 Hz, 1H), 5.92 (ddt, *J* = 16.7, 9.9, 6.6 Hz, 1H), 5.10 – 4.98 (m, 4H), 4.90 – 4.70 (m, 4H), 3.44 – 3.41 (m, 6H), 3.41 – 3.35 (m, 2H), 3.31 – 3.28 (m, 6H) overlapped by 3.29 – 3.23 (m, 6H), 2.53 – 2.49 (m, 6H), 2.27 (s, 3H), 2.21 (s, 3H); ^{13}C NMR (126.0 MHz, $CDCl_3$): δ_C 151.4 – 151.1 (m), 150.7 – 150.5 (m), 146.6 (br), 146.4 (br), 138.4, 129.6 – 129.0 (m), 128.3, 127.4 – 126.9 (m), 125.3, 124.8 – 124.6 (m), 123.8, 123.7, 121.4, 121.3, 121.0, 120.9, 115.05, 115.0, 113.05, 113.0, 112.7 – 112.5 (m), 112.3 – 112.1, 64.65, 64.6, 64.45, 64.4, 55.8, 55.75,44.1 – 43.8 (m), 40.1, 39.9, 21.1, 21.0; the four methoxy signals 64.65 – 64.4 ppm show evidence of shoulders point to the presence of isomeric complex (*iso*-**3k**) of the same constitution, as do the multiple aryl environments; ν_{max} (ATR): 2909, 1637, 1579, 1484, 1384, 1244, 1152, 1097, 989, 827, 720, 580, 487 cm^{-1}; HRMS (ESI) Calcd for $[M+H]^+$ $C_{42}H_{50}N_2O_8Ti$ is 759.3119 found 759.3140 (|σ| = 2.7 ppm); Anal Calcd (%) for $C_{42}H_{50}N_2O_8Ti$, C, 66.49; H, 6.64; N, 3.69, found C, 66.53; H, 6.68, N, 3.71. This compound couldn't be recrystallized into a recognizable habit from diethyl ether/pentane but was attained as an analytically pure powder.

■ 1.1 ^1H and ^{13}C NMR spectra for compounds (1-3)

Presented on pages 9-52.

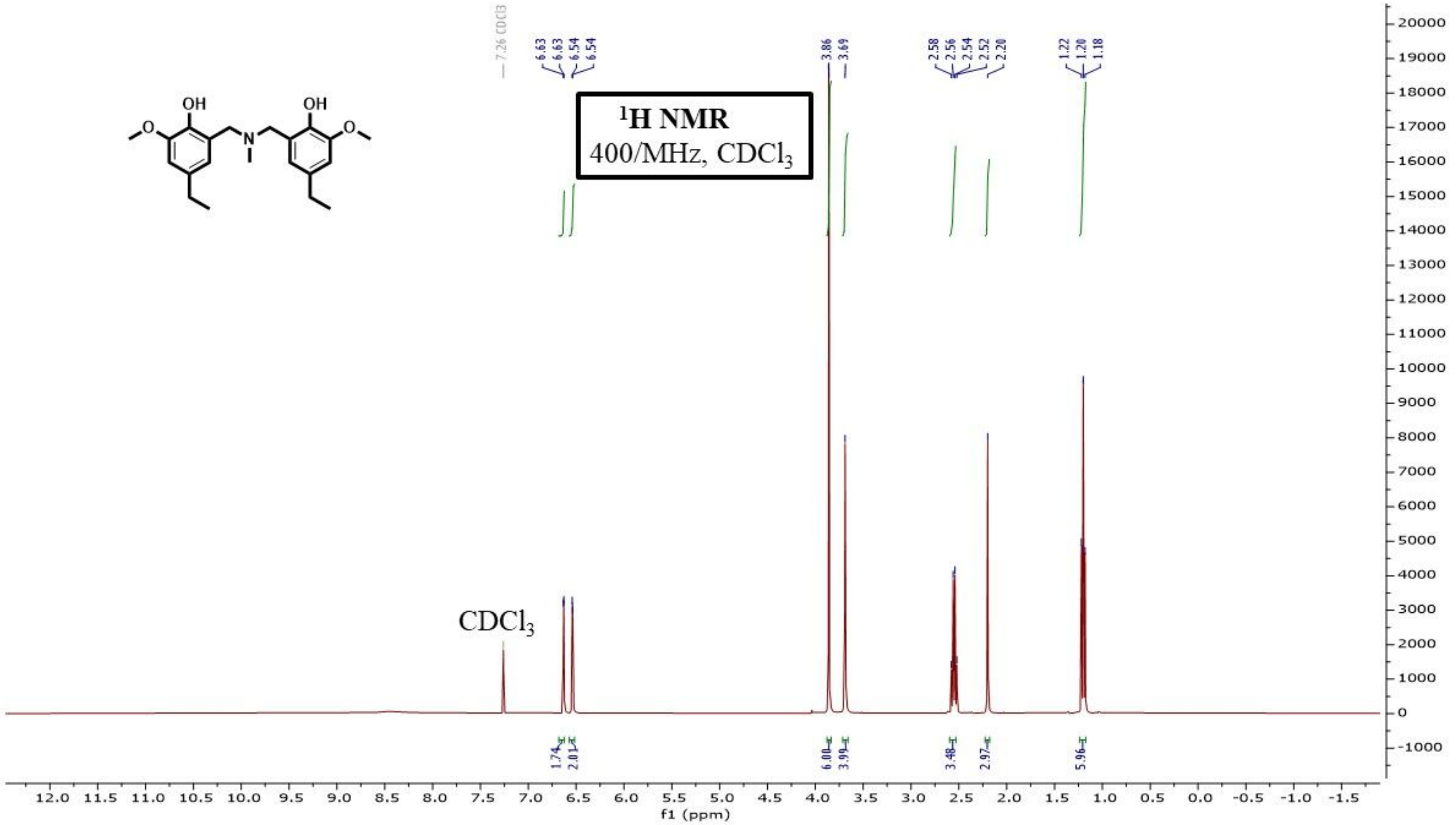

Figure S1. Representative ^{1}H NMR spectrum of intermediate ligand 6,6'-((methylazanediyl)*bis*(methylene))*bis*(4-ethyl-2-methoxyphenol) (**1b**).

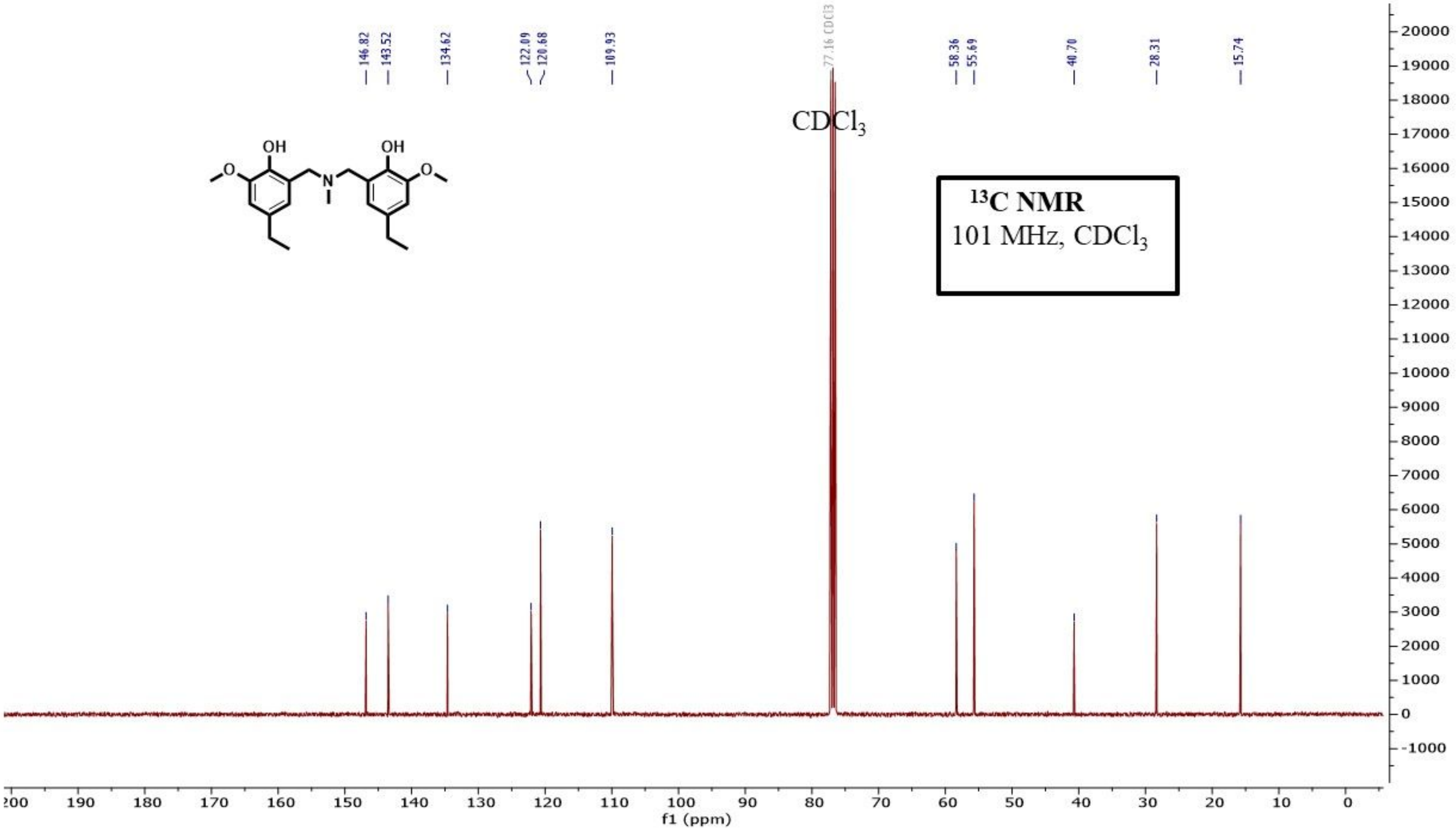

Figure S2. Representative ^{13}C NMR spectrum of intermediate ligand 6,6'-((methylazanediyl)*bis*(methylene))*bis*(4-ethyl-2-methoxyphenol) (**1b**).

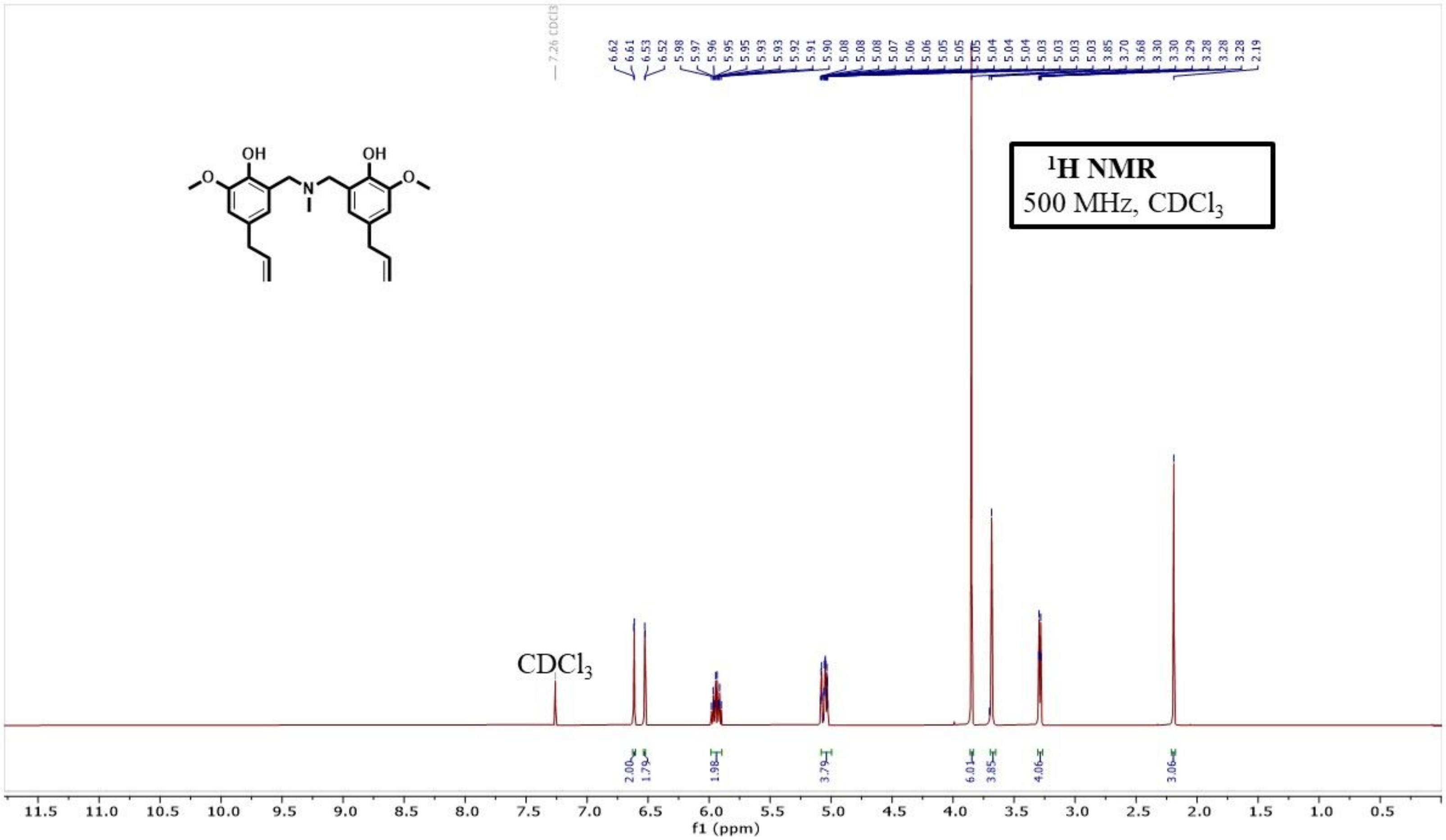

Figure S3. Representative ^{1}H NMR spectrum of intermediate ligand 6,6'-((methylazanediyl)*bis*(methylene))*bis*(4-allyl-2-methoxyphenol (**1c**).

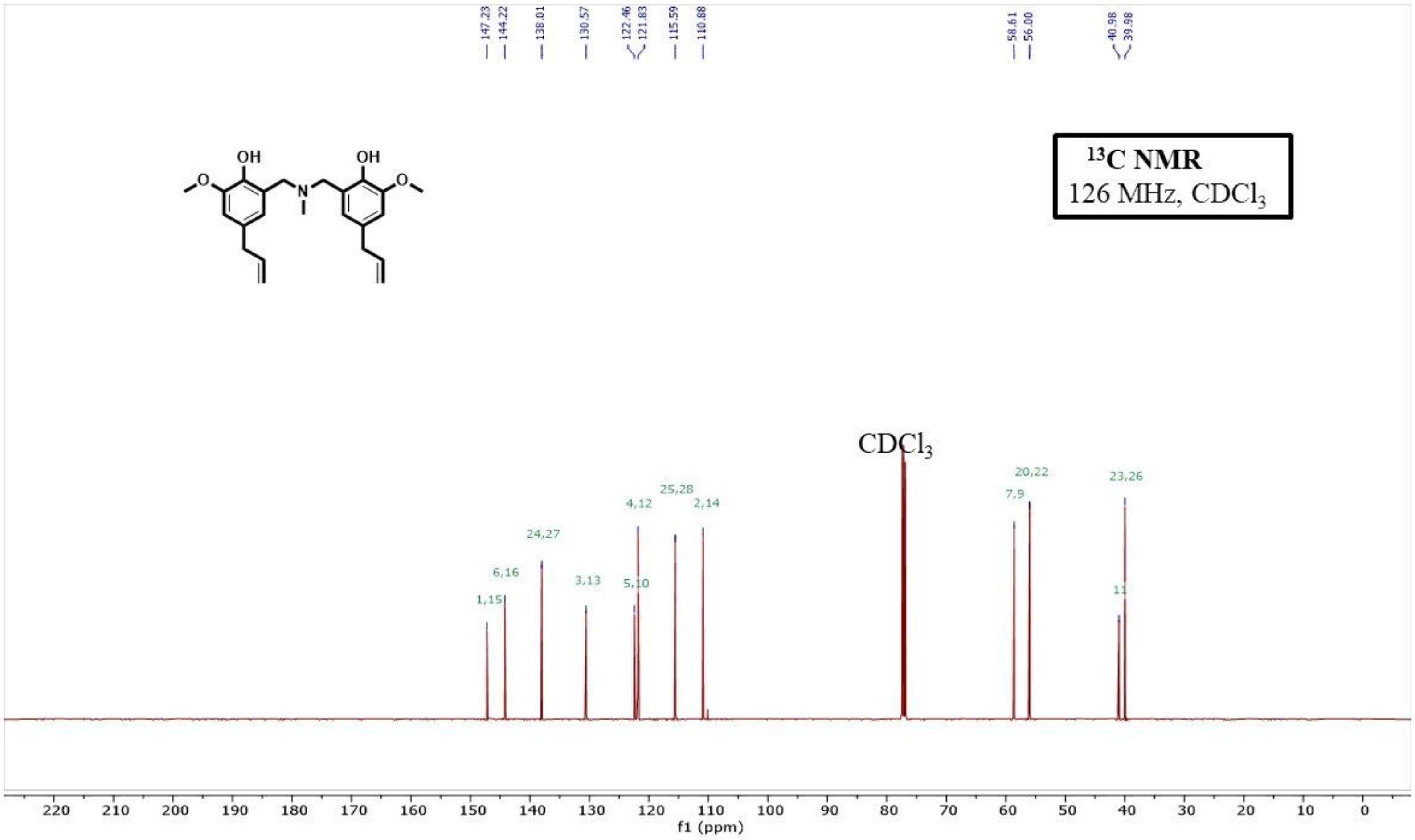

Figure S4. Representative ^{13}C NMR spectrum of intermediate ligand 6,6'-((methylazanediyl)*bis*(methylene))*bis*(4-allyl-2-methoxyphenol (**1c**).

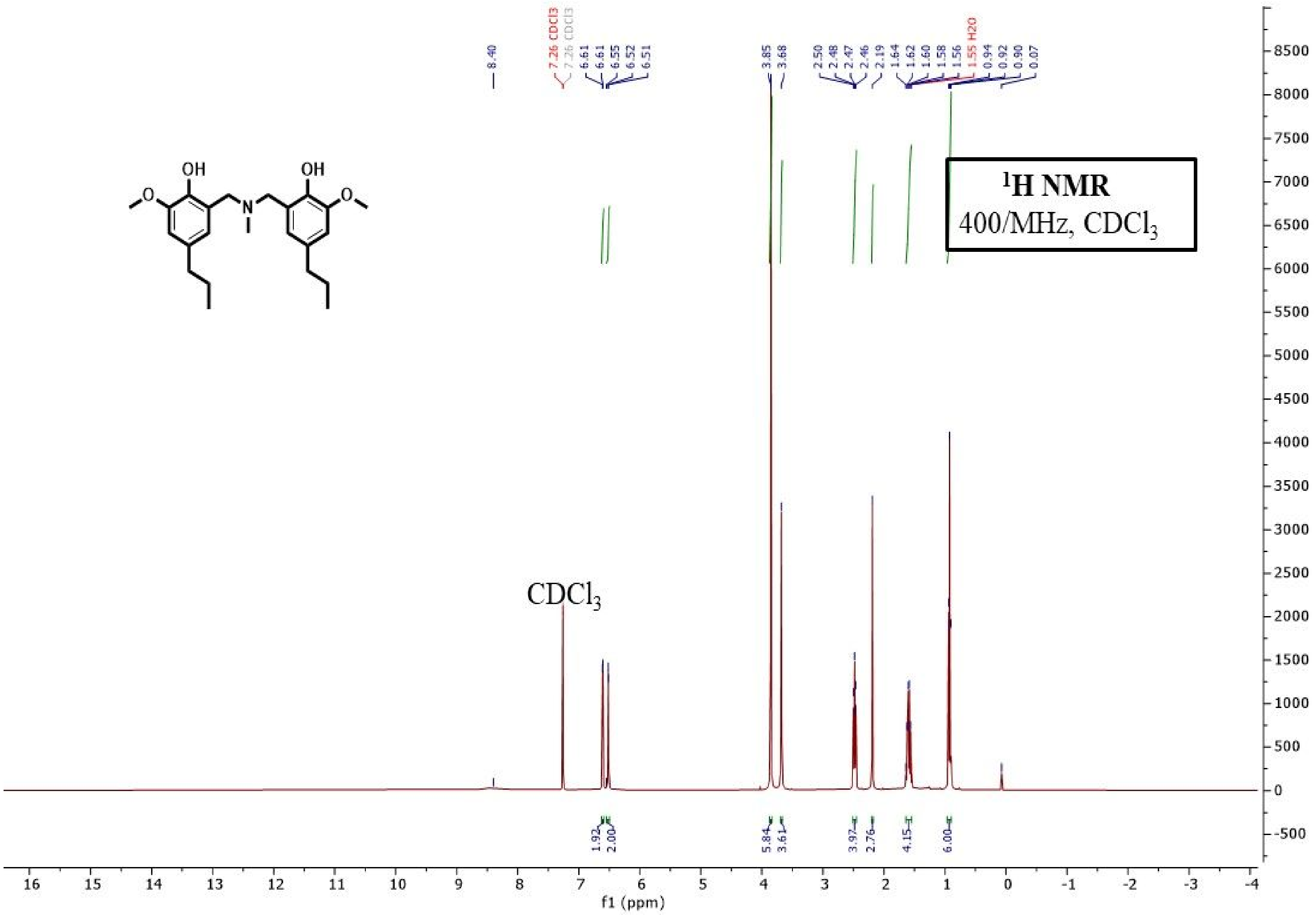

Figure S5. Representative ^{1}H NMR spectrum of intermediate ligand 6,6'-((methylazanediyl)*bis*(methylene))*bis*(2-methoxy-4-propylphenol) (**1d**).

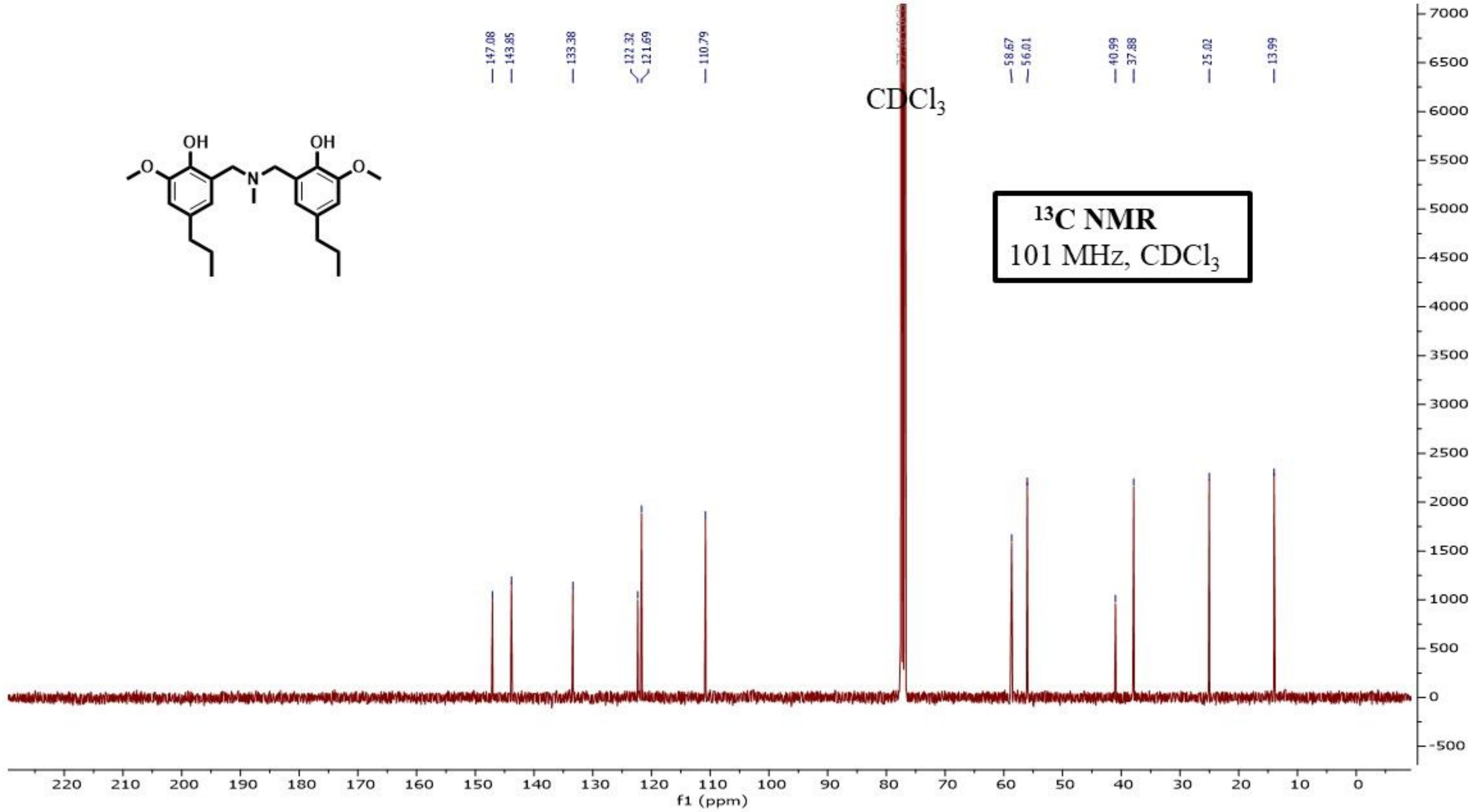

Figure S6. Representative ^{13}C NMR spectrum of intermediate ligand 6,6'-((methylazanediyl)*bis*(methylene))*bis*(2-methoxy-4-propylphenol) (**1d**).

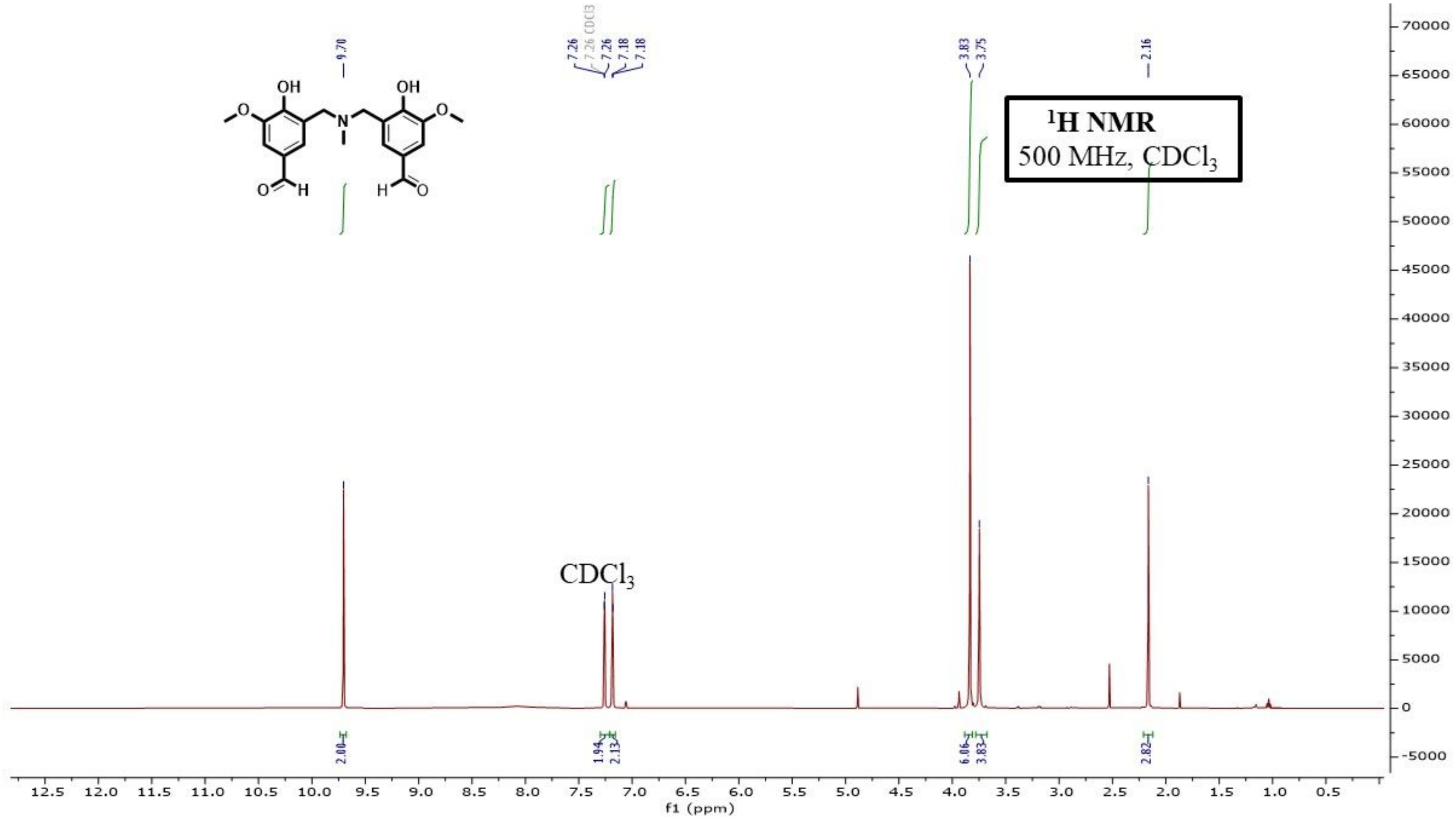

Figure S7. Representative ^{1}H NMR spectrum of intermediate ligand 5,5'-((methylazanediyl)*bis*(methylene))*bis*(4-hydroxy-3-methoxybenzaldehyde) (**1e**).

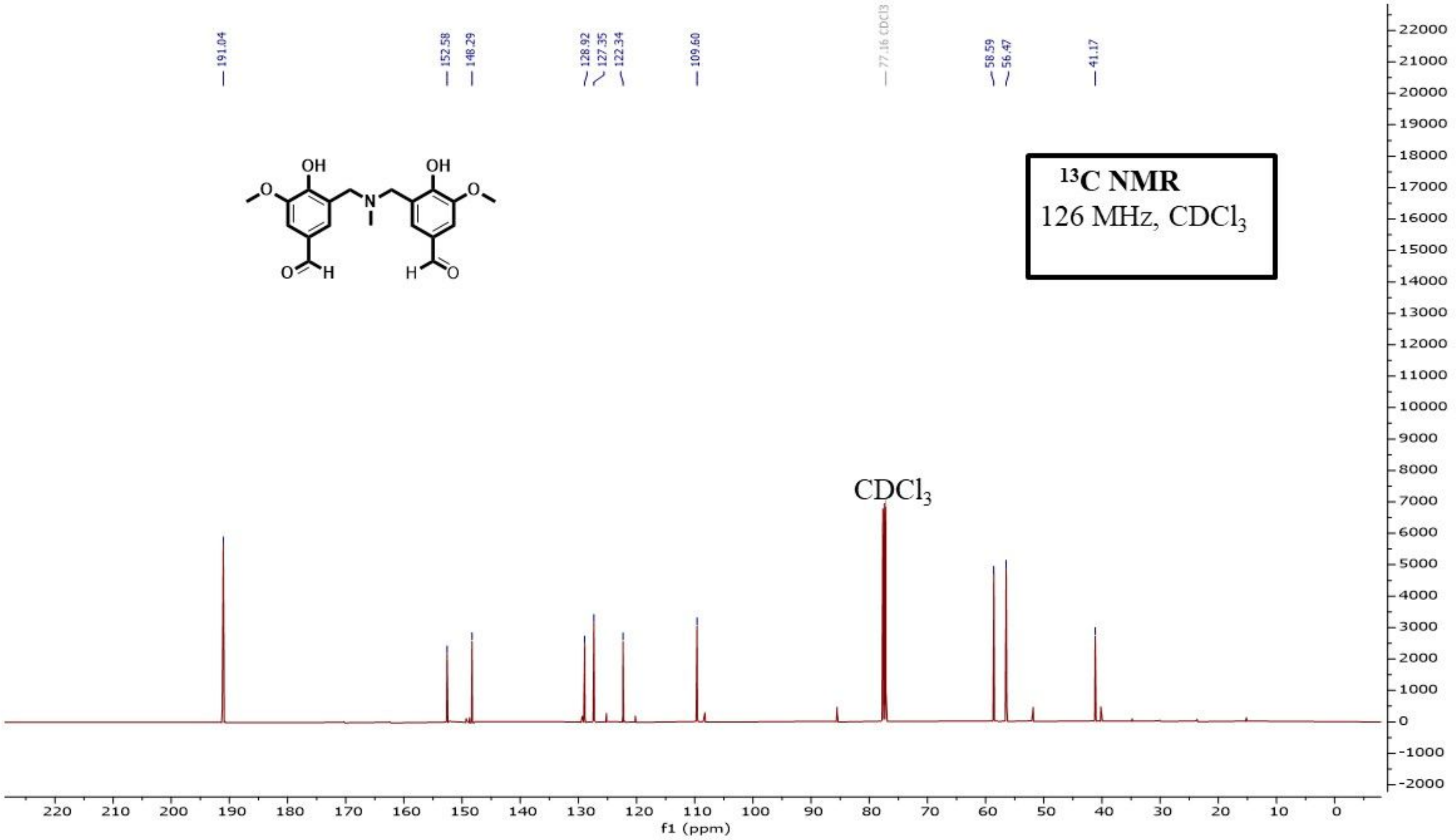

Figure S8. Representative ^{13}C NMR spectrum of intermediate ligand 5,5'-((methylazanediyl)*bis*(methylene))*bis*(4-hydroxy-3-methoxybenzaldehyde) (**1e**)

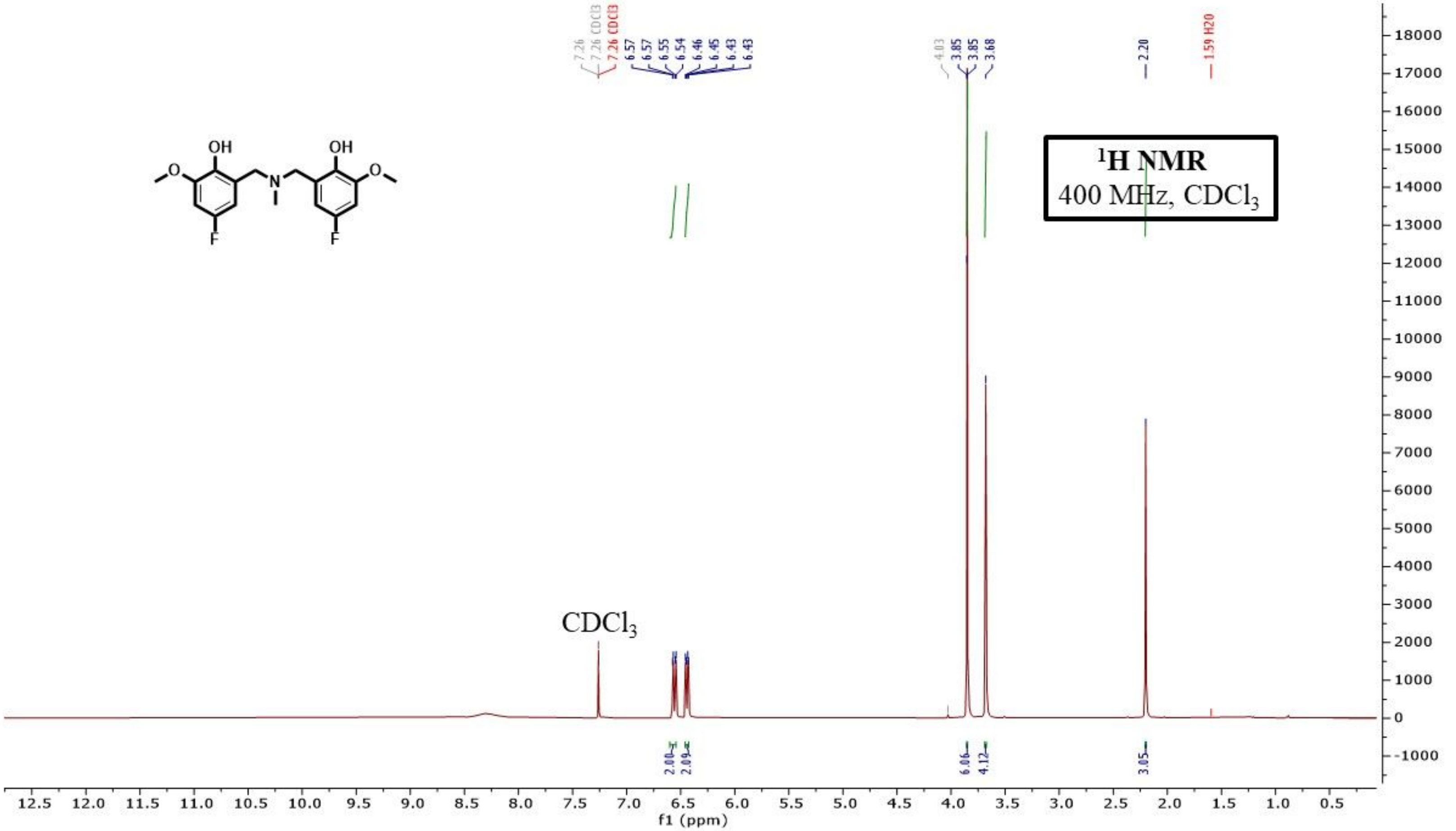

Figure S9. Representative ^{1}H NMR spectrum of intermediate ligand 6,6'-((methylazanediyl)*bis*(methylene))*bis*(4-fluoro-2-methoxyphenol) (**1f**).

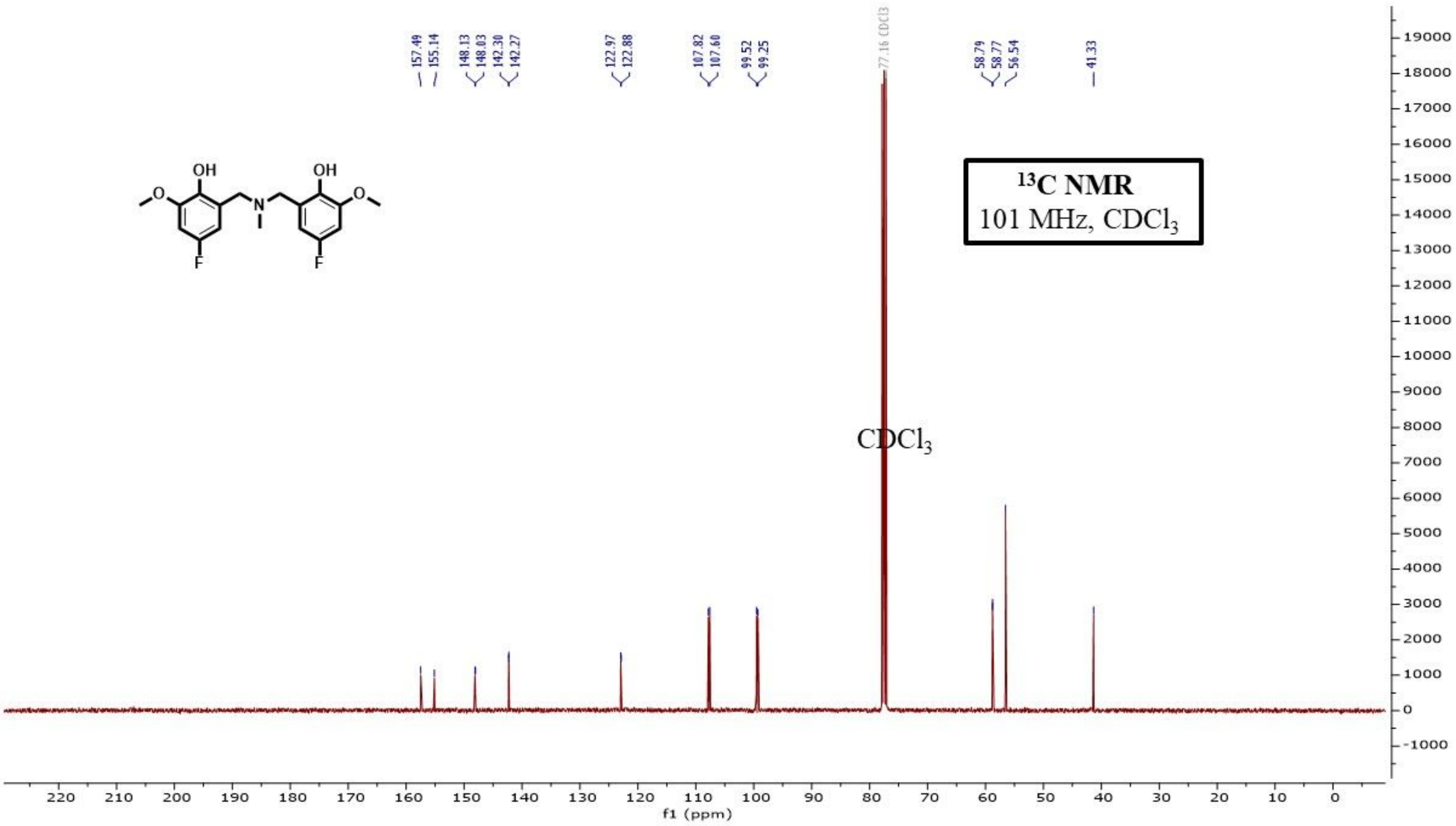

Figure S10. Representative ^{13}C NMR spectrum of intermediate ligand 6,6'-((methylazanediyl)*bis*(methylene))*bis*(4-fluoro-2-methoxyphenol) (**1f**).

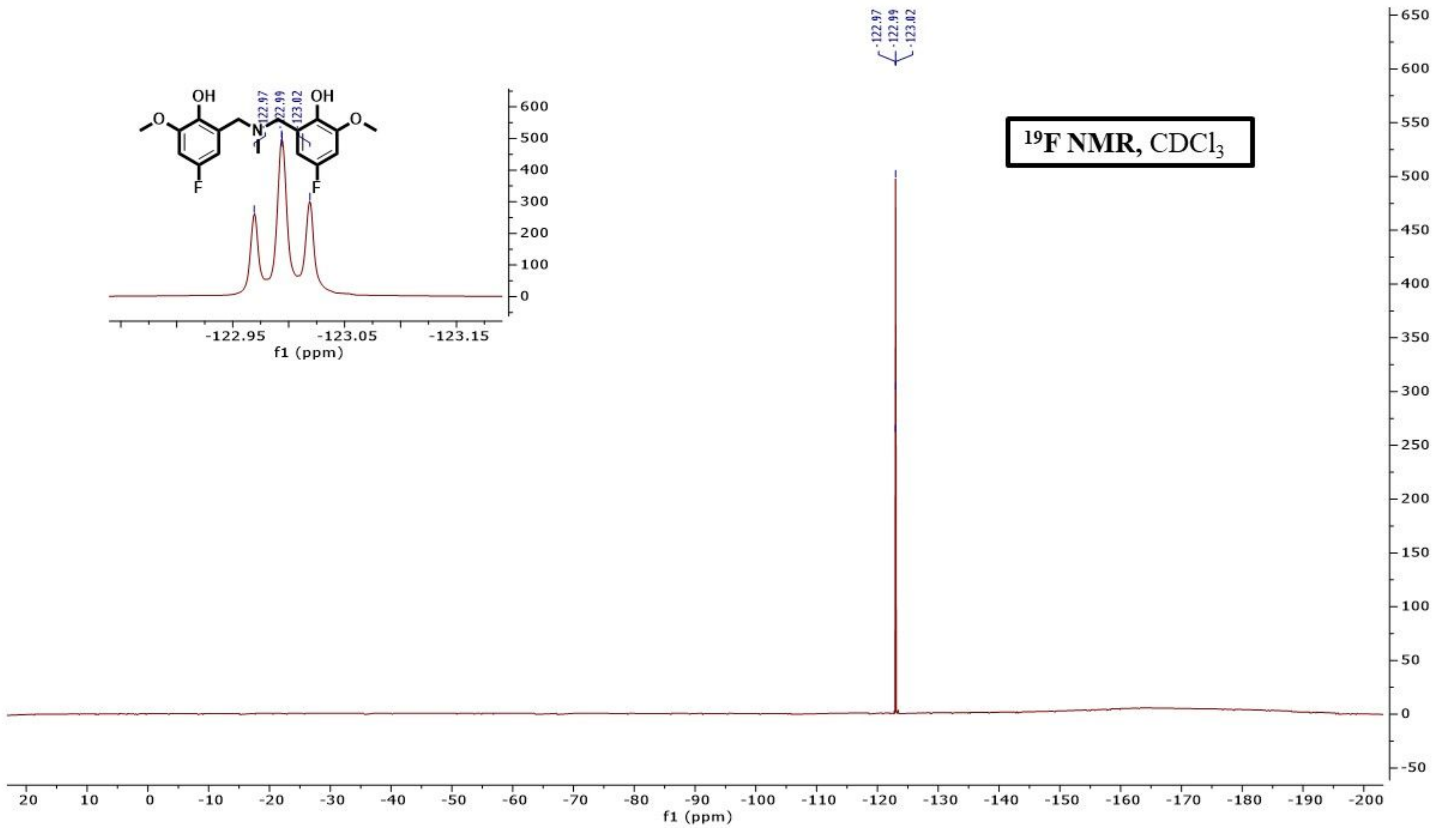

Figure S11. Representative ^{19}F spectrum of intermediate ligand 6,6'-((methylazanediyl)*bis*(methylene))*bis*(4-fluoro-2-methoxyphenol) (**1f**); ^{1}H coupled spectrum in insert.

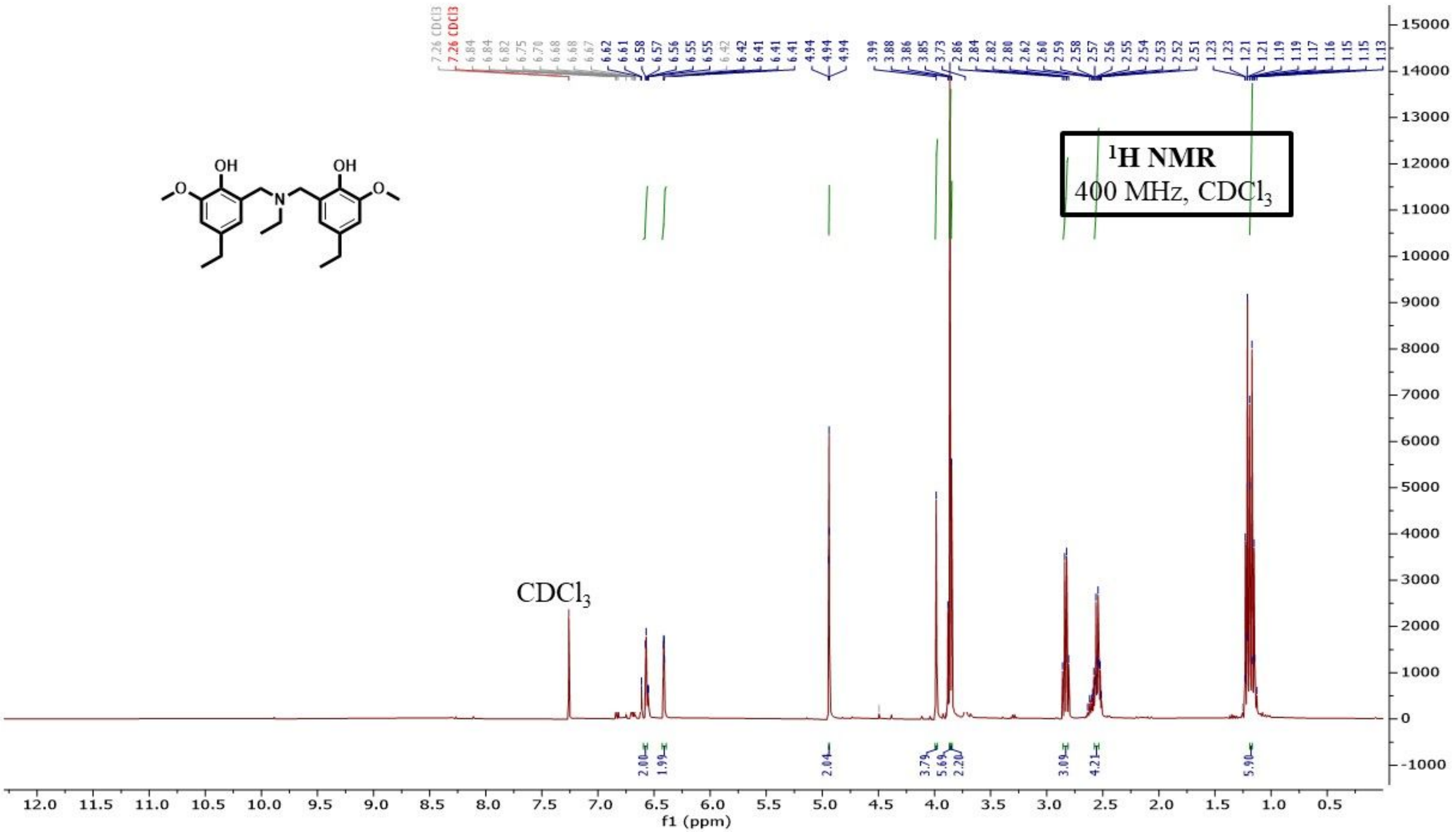

Figure S12. Representative ^{1}H NMR spectrum of intermediate ligand 6,6'-((ethylazanediyl)*bis*(methylene))*bis*(4-ethyl-2-methoxyphenol) (**1g**).

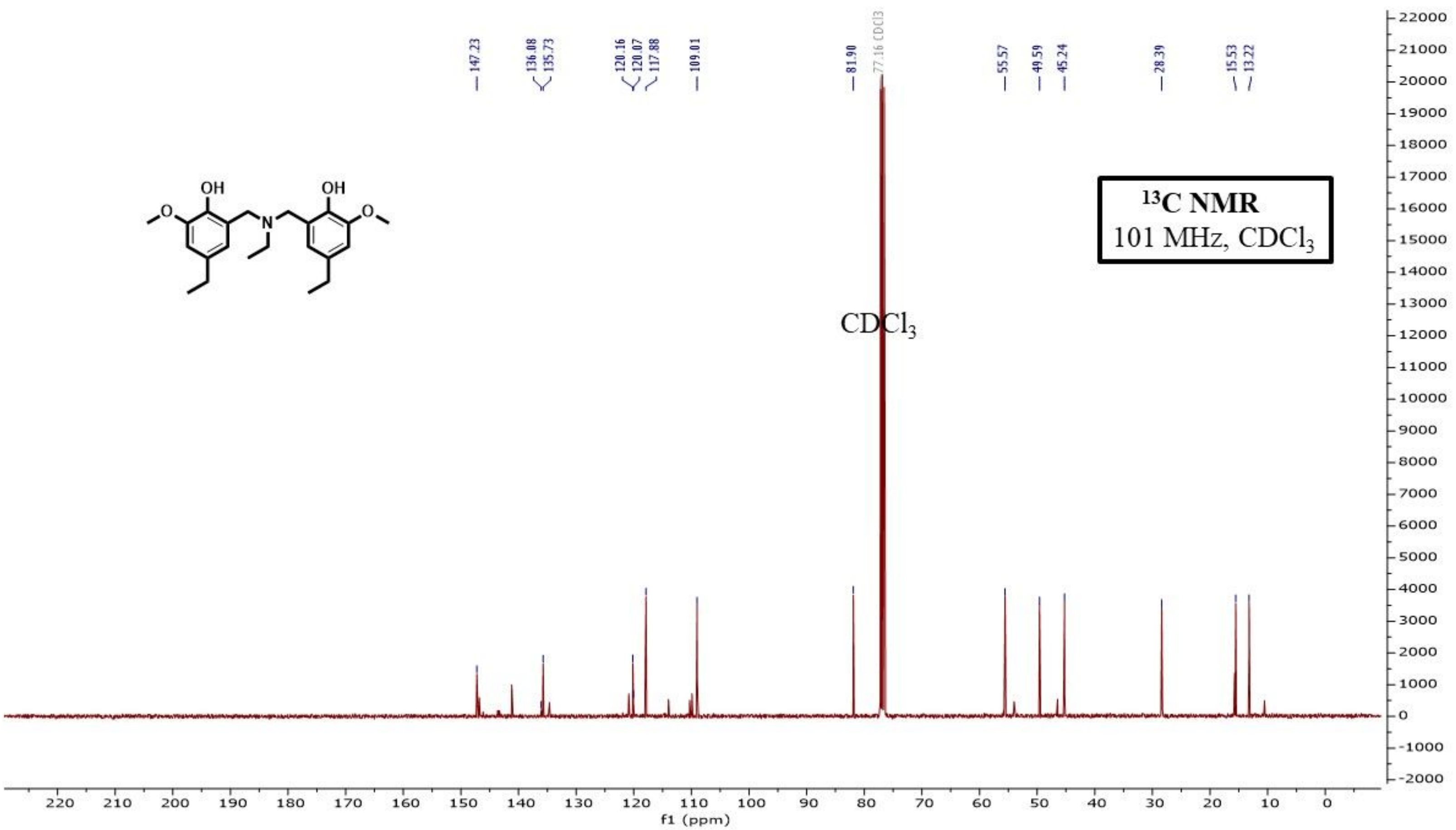

Figure S13. Representative ^{13}C NMR spectrum of intermediate ligand 6,6'-((ethylazanediyl)*bis*(methylene))*bis*(4-ethyl-2-methoxyphenol) (**1g**).

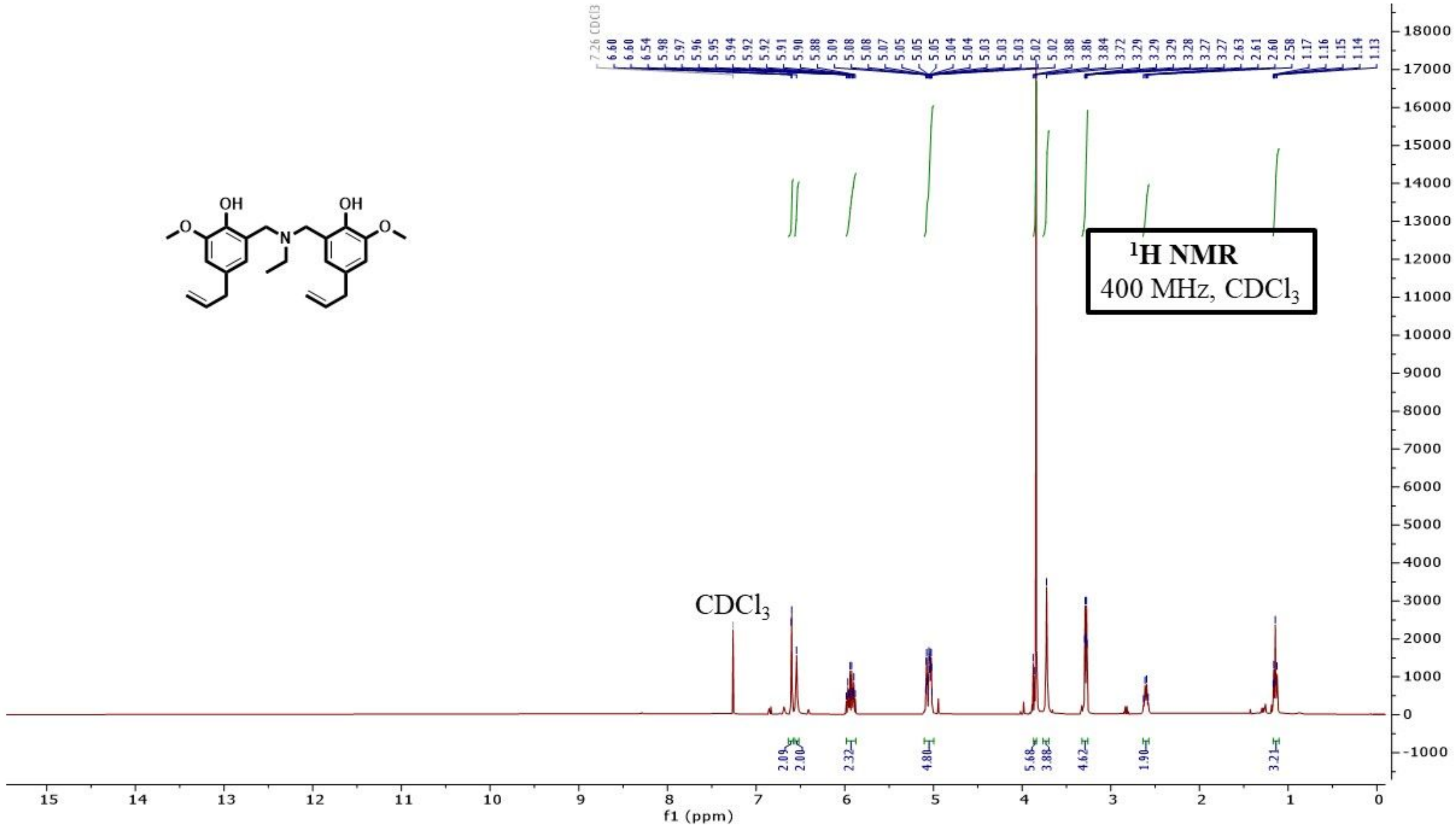

Figure S14. Representative ^{1}H NMR spectrum of intermediate ligand 6,6'-((ethylazanediyl)*bis*(methylene))*bis*(4-allyl-2-methoxyphenol) (**1h**).

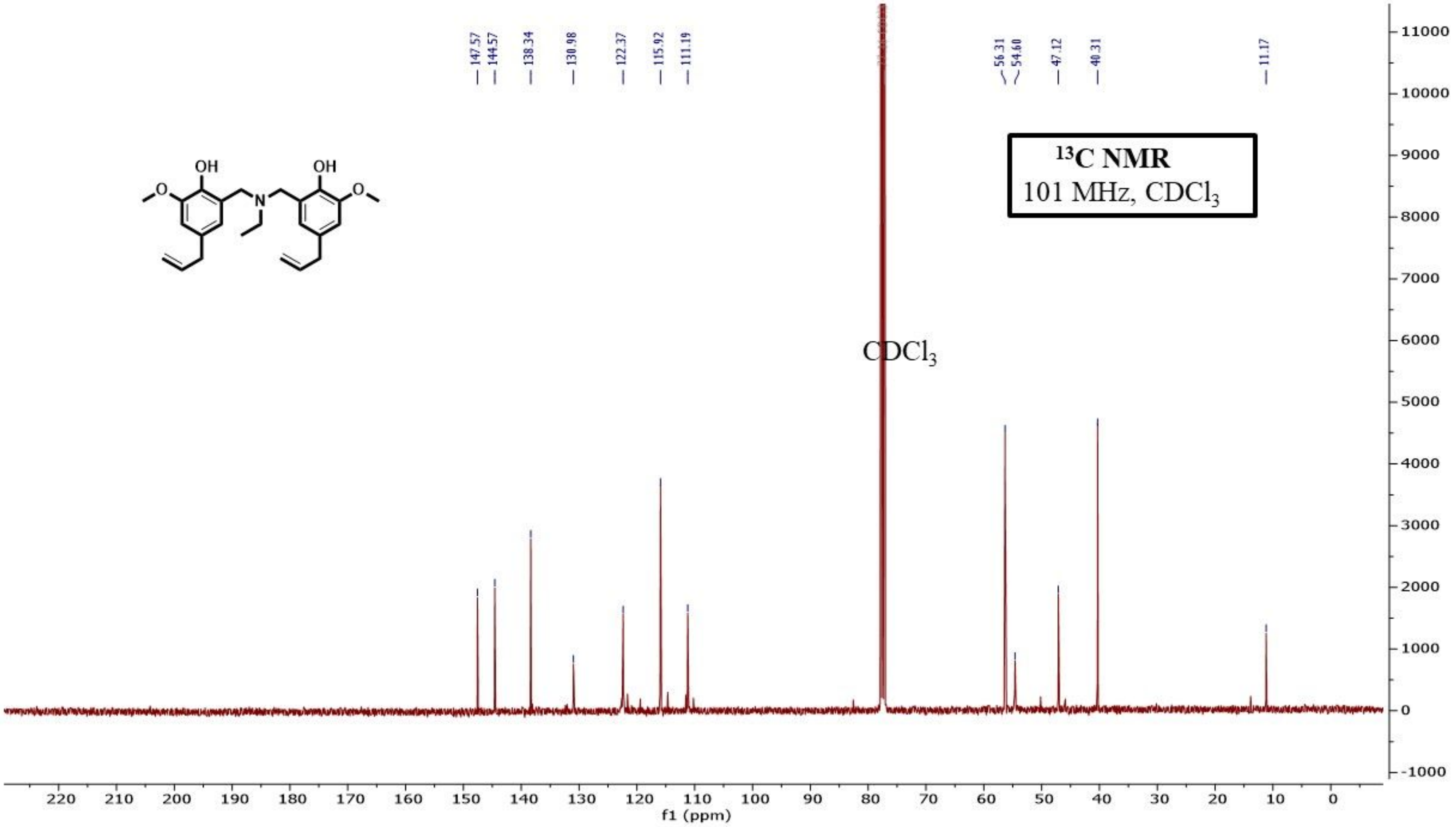

Figure S15. Representative ^{13}C NMR spectrum of intermediate ligand 6,6'-((ethylazanediyl)*bis*(methylene))*bis*(4-allyl-2-methoxyphenol) (**1h**).

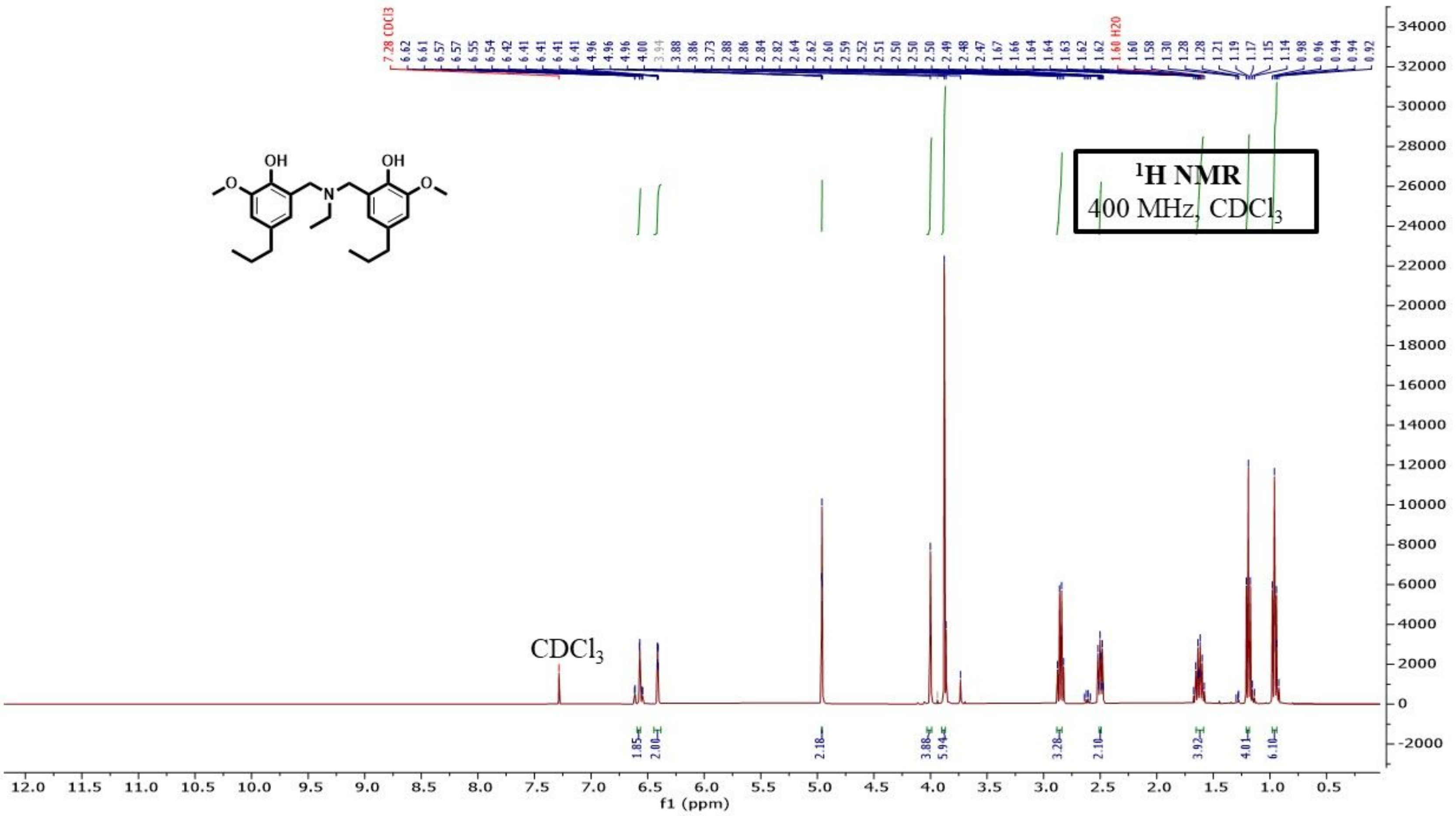

Figure S16. Representative ^{1}H NMR spectrum of intermediate ligand 6,6'-((ethylazanediyl)*bis*(methylene))*bis*(2-methoxy-4-propylphenol) (**1i**).

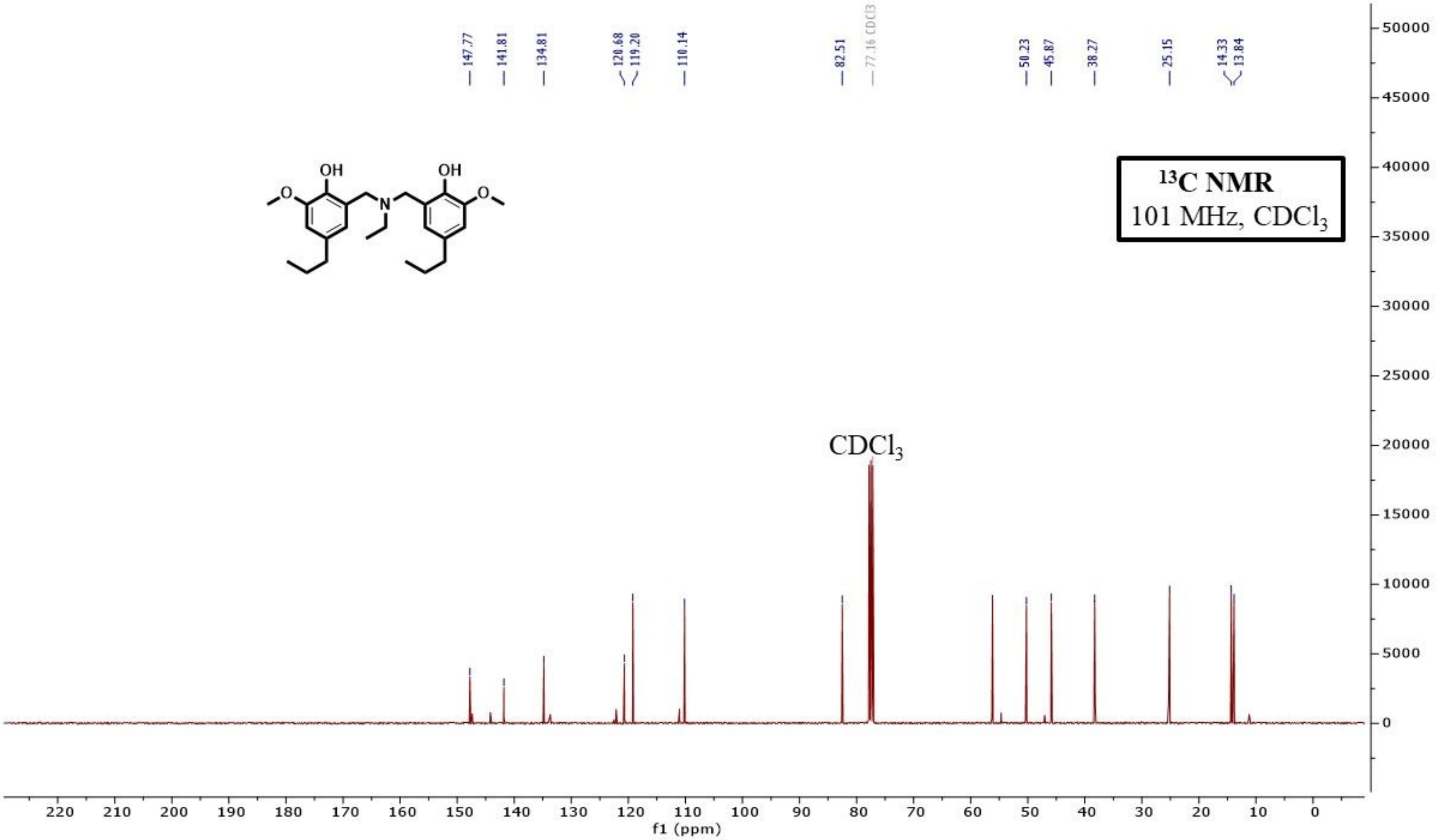

Figure S17. Representative ^{13}C NMR spectrum of intermediate ligand 6,6'-((ethylazanediyl)*bis*(methylene))*bis*(2-methoxy-4-propylphenol) (**1i**).

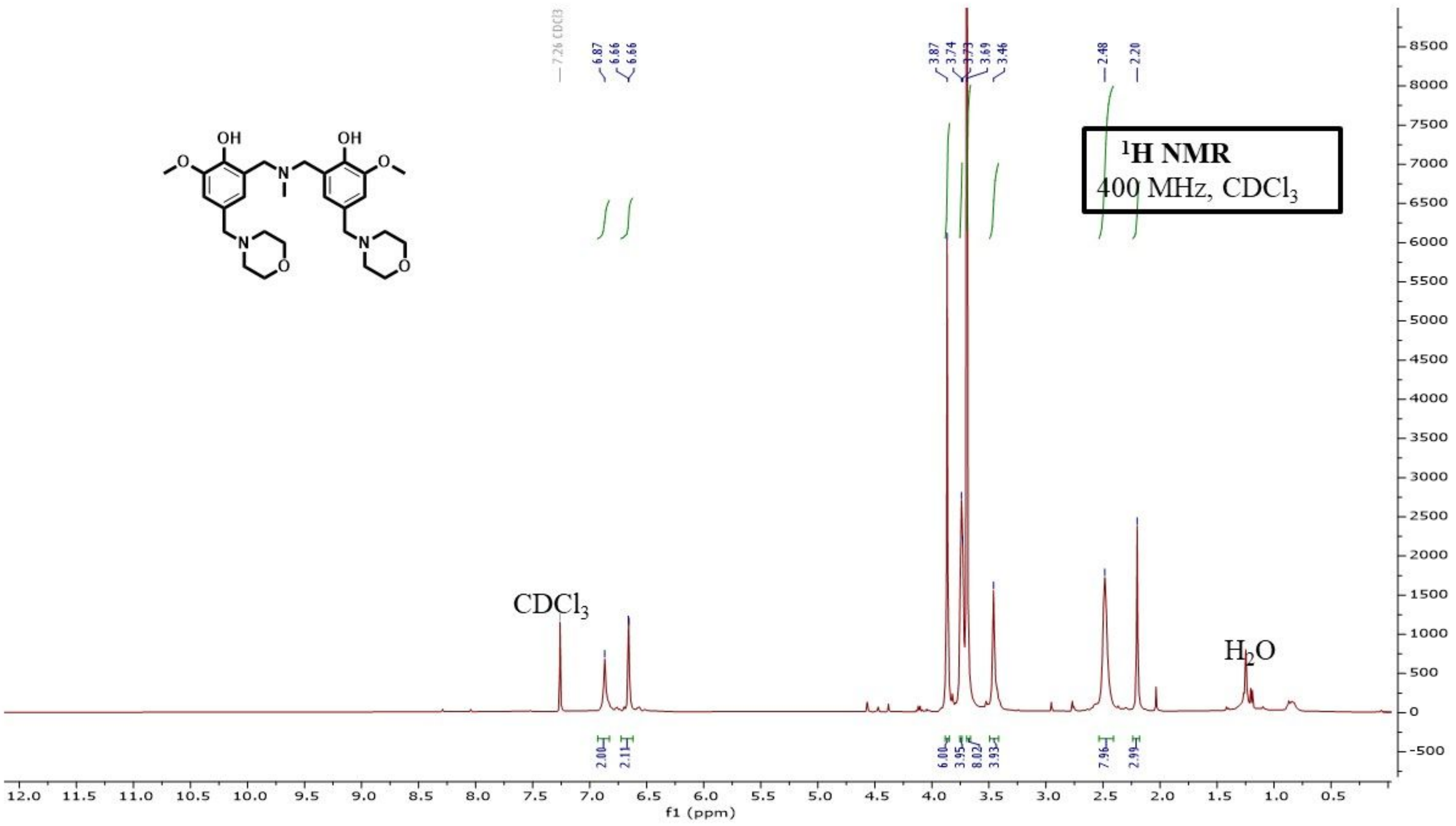

Figure S18. Representative ^{1}H NMR spectrum of intermediate ligand6,6'-((methylazanediyl)*bis*(methylene))*bis*(2-methoxy-4-(morpholinomethyl)phenol) (**1j**).

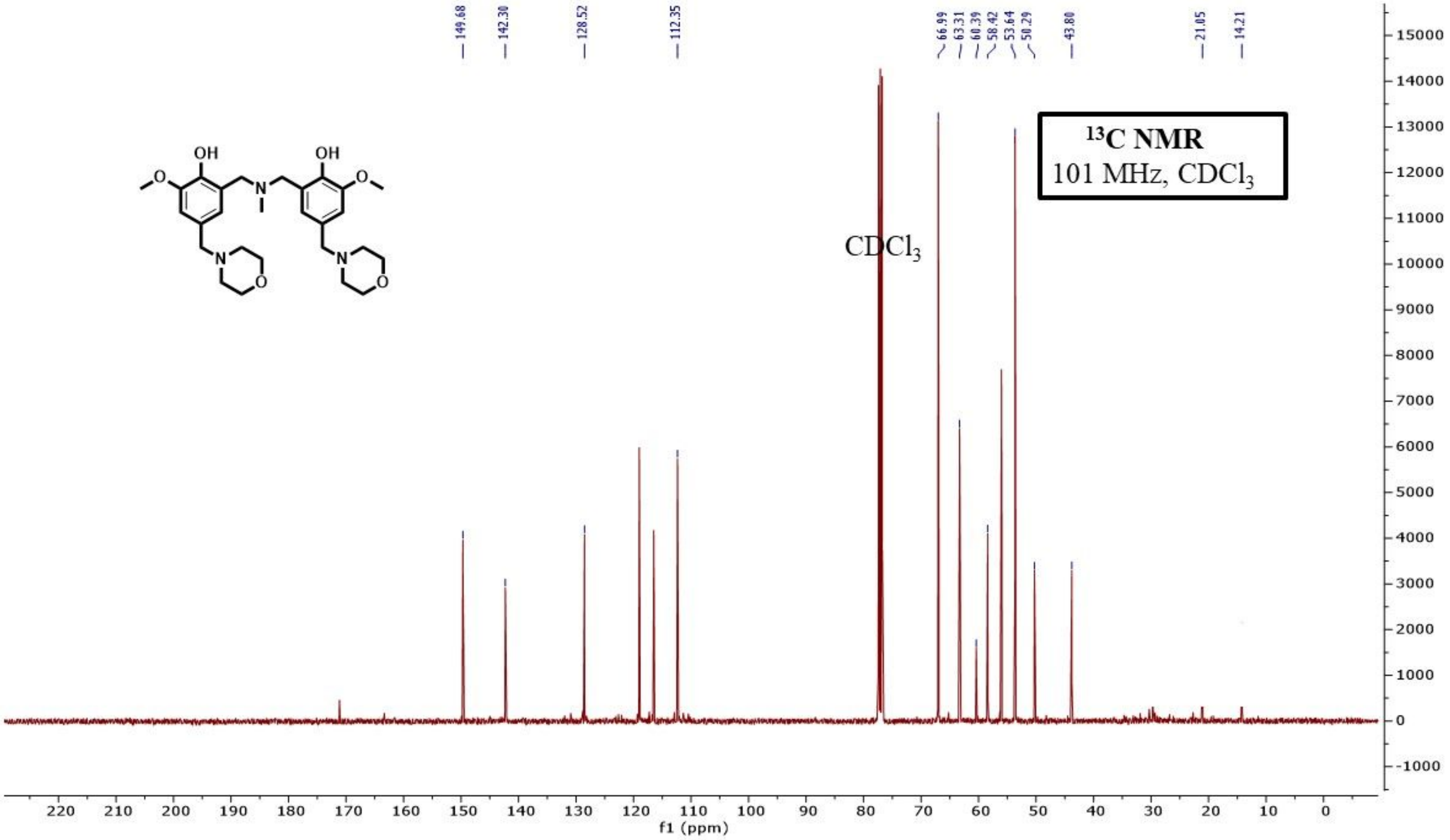

Figure S19. Representative ^{13}C NMR spectrum of intermediate ligand 6,6'-((methylazanediyl)*bis*(methylene))*bis*(2-methoxy-4-(morpholinomethyl)phenol) (**1j**).

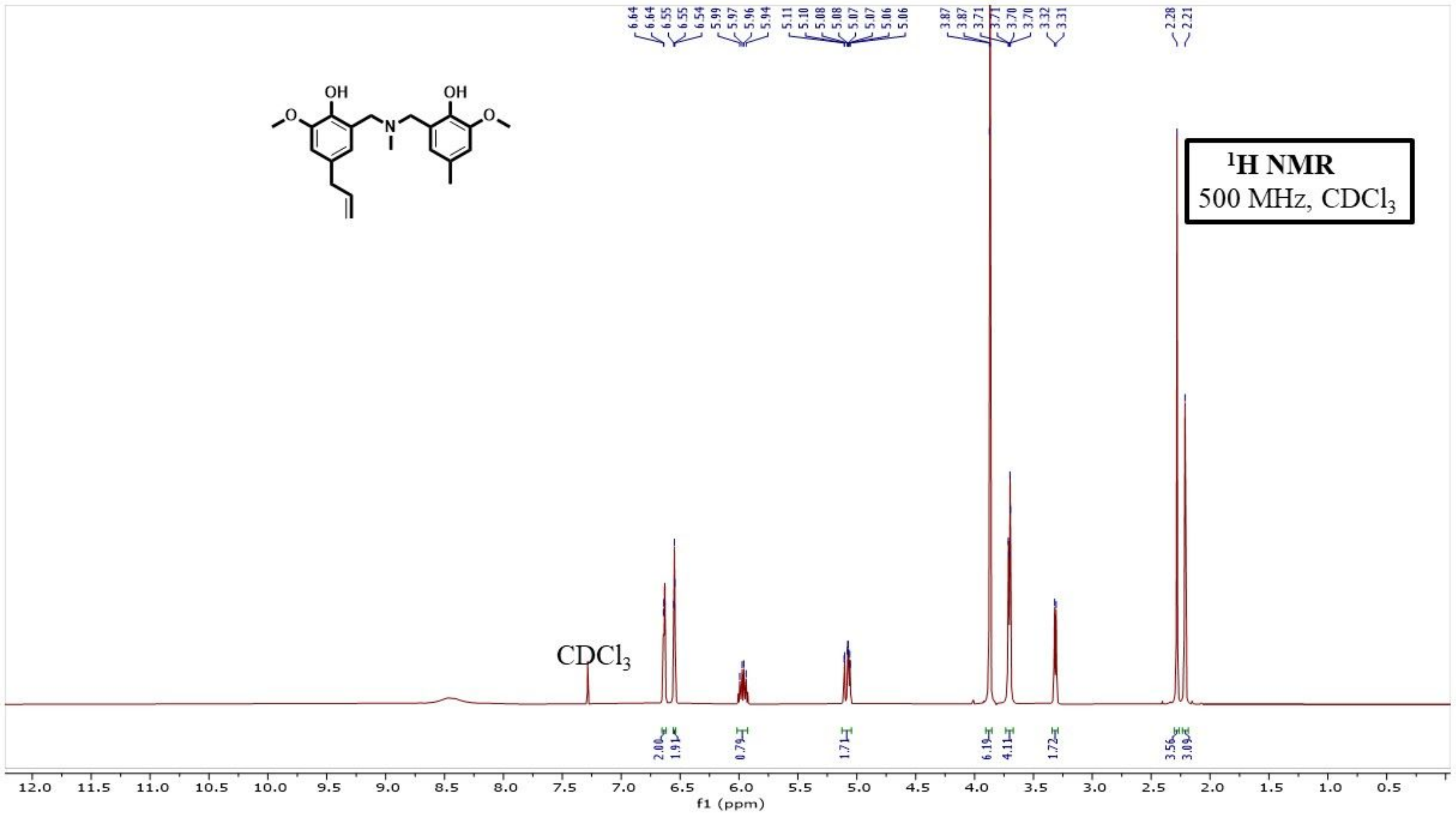

Figure S20. Representative ¹H NMR spectrum of intermediate ligand 4-allyl-2-(((2-hydroxy-3-methoxy-5-methylbenzyl)(methyl)amino)methyl)-6-methoxyphenol (**1k**).

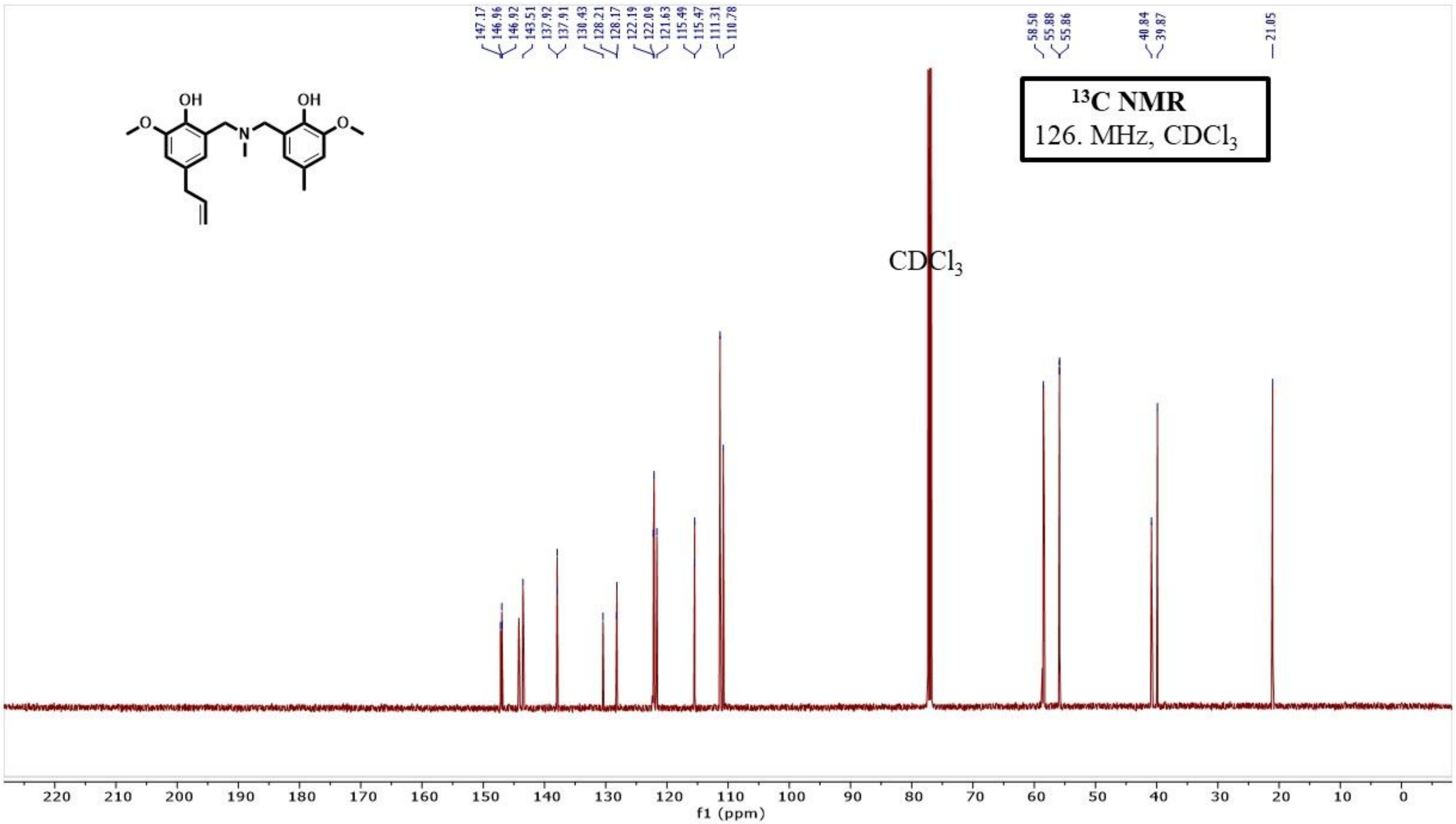

Figure S21. Representative ^{13}C NMR spectrum of intermediate ligand 4-allyl-2-(((2-hydroxy-3-methoxy-5-methylbenzyl)(methyl)amino)methyl)-6-methoxyphenol (**1k**).

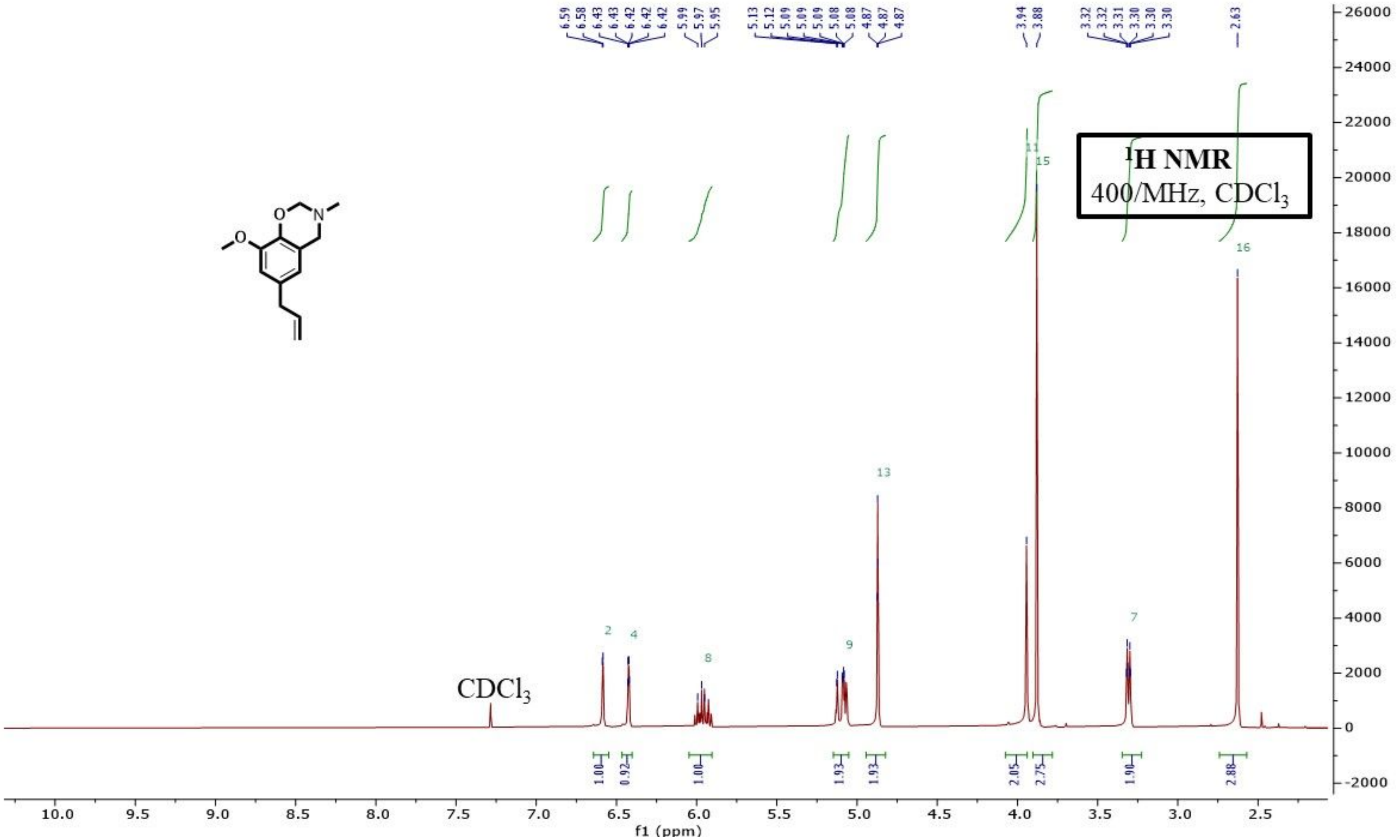

Figure S22. Representative ^{1}H NMR spectrum of intermediate 6-allyl-3-methyl-3,4-dihydro-*2H*-benzo[e][1,3]oxazine (**2c**).

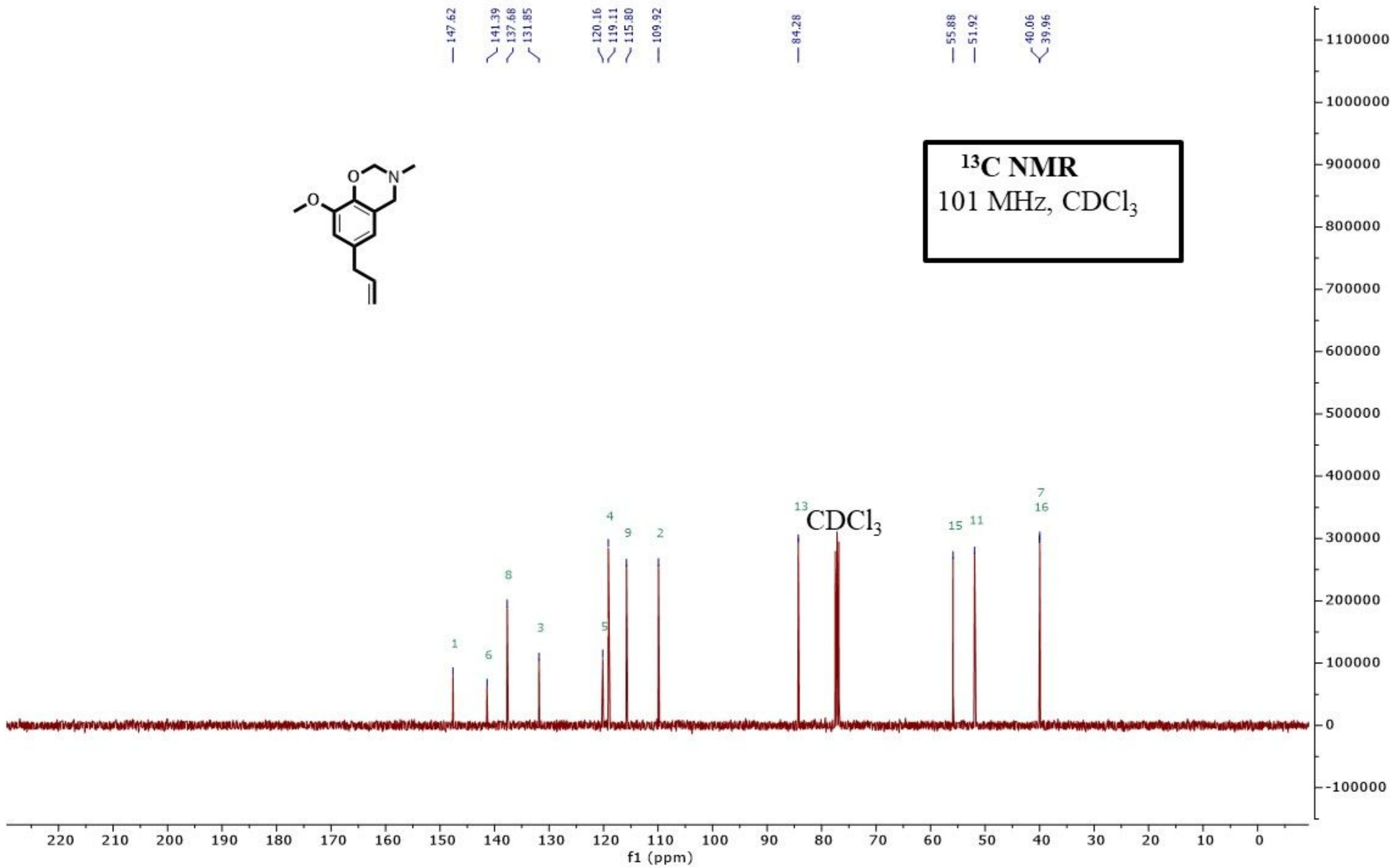

Figure S23. Representative ^{13}C NMR spectrum of intermediate 6-allyl-3-methyl-3,4-dihydro-*2H*-benzo[*e*][1,3]oxazine (**2c**).

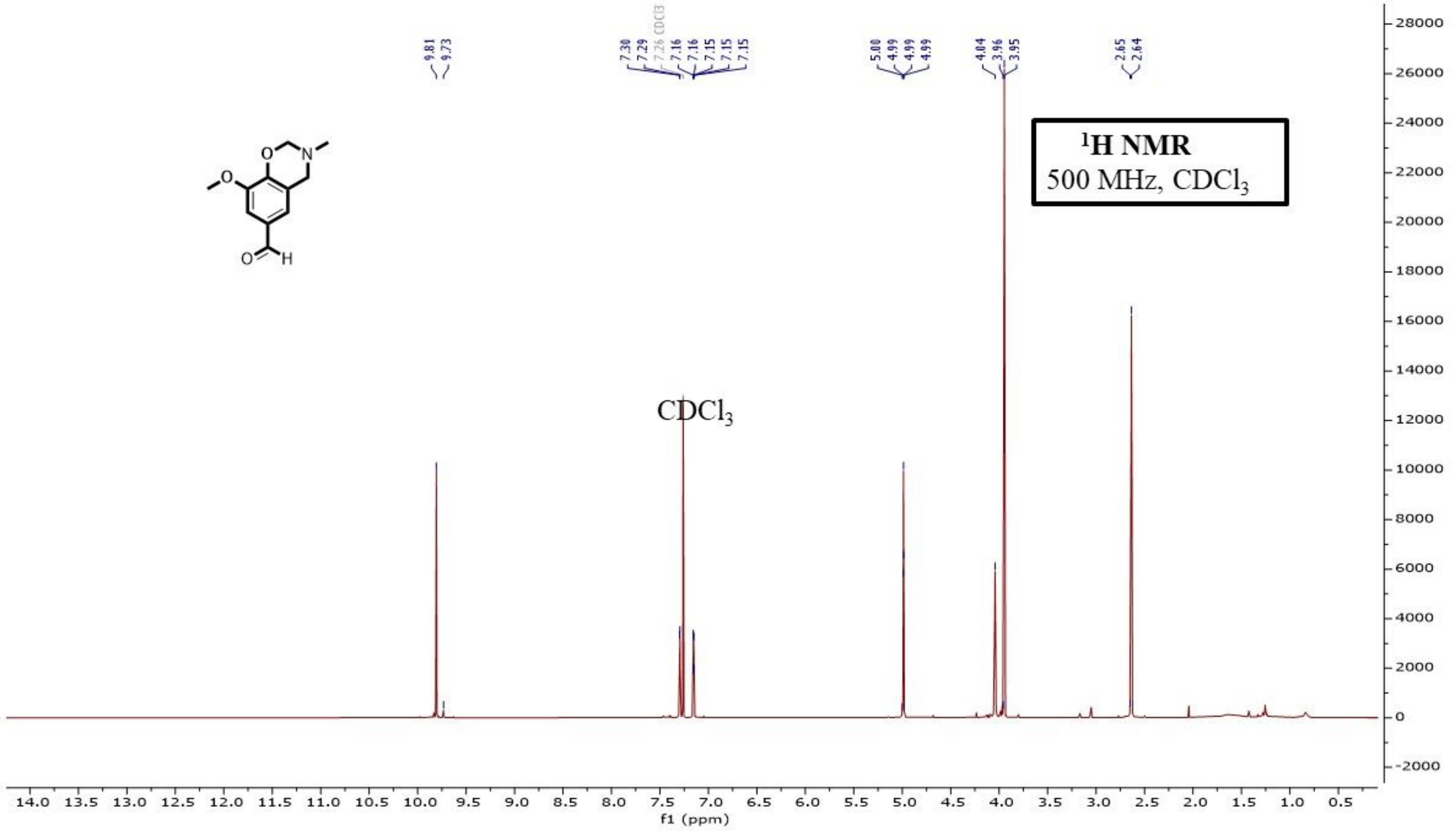

Figure S24. Representative ^{1}H NMR spectrum of intermediate methoxy-3-methyl-3,4-dihydro-*2H*-benzo[*e*][1,3]oxazine-6-carbaldehyde (**2e**).

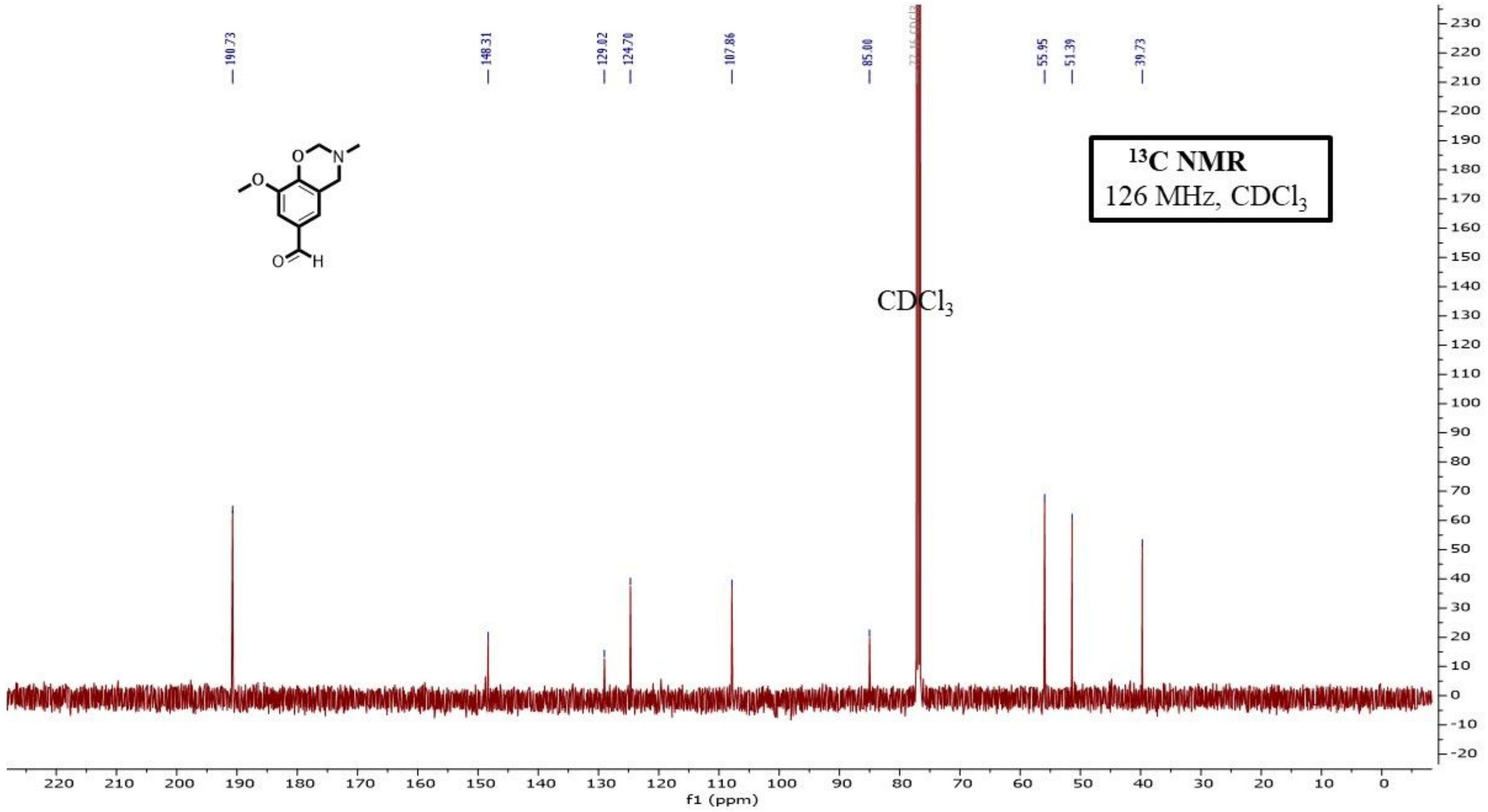

Figure S25. Representative ^{13}C NMR spectrum of intermediate methoxy-3-methyl-3,4-dihydro-*2H*-benzo[*e*][1,3]oxazine-6-carbaldehyde (**2e**).

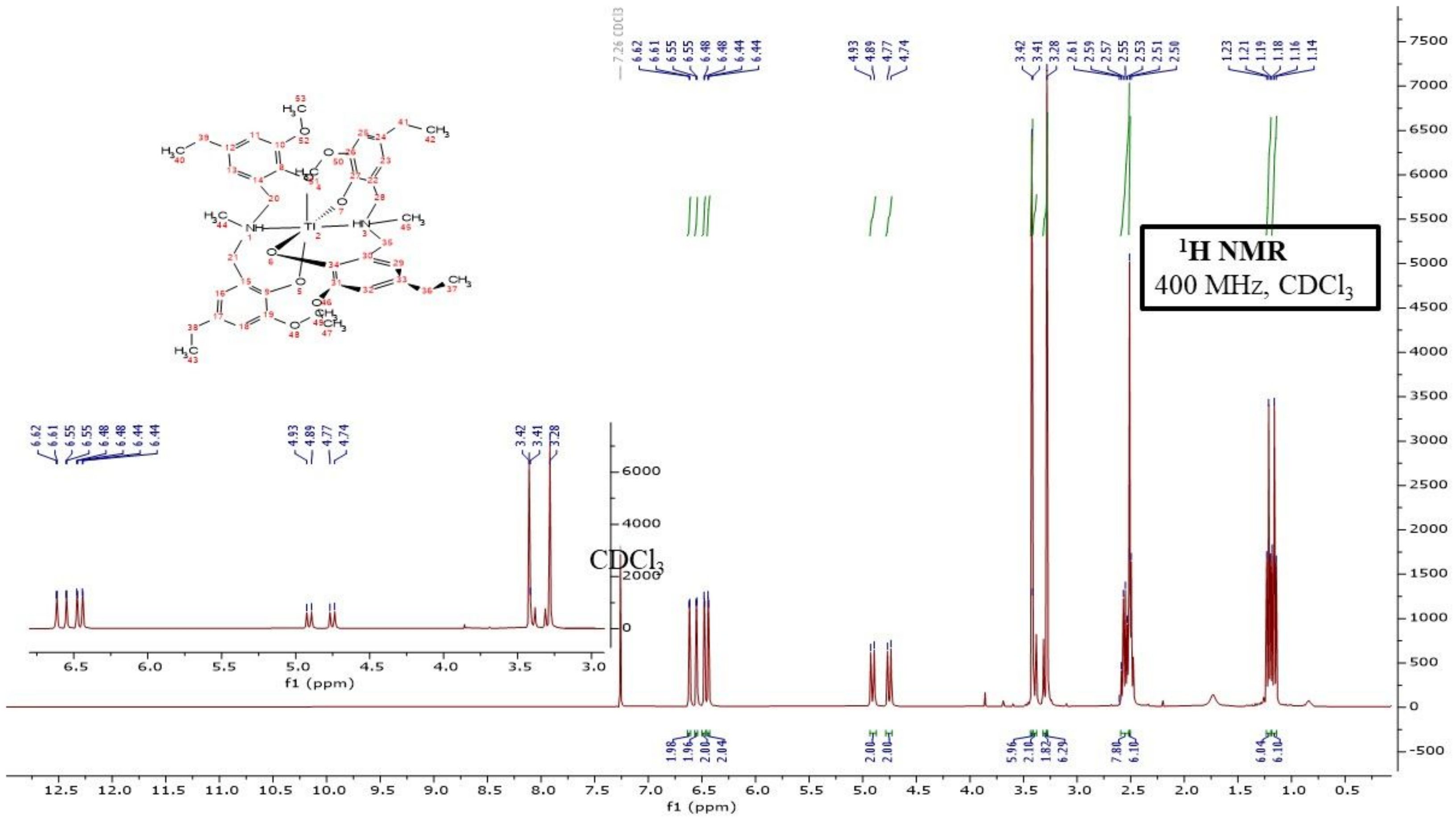

Figure S26. Representative ^{1}H NMR spectrum of final complex *bis*((2,2’-((methylimino-*N*)*bis*(methylene))*bis*(4-ethyl-6-methoxyphenolato-*O*)))titanium(IV) (**3b**); expansion inserted.

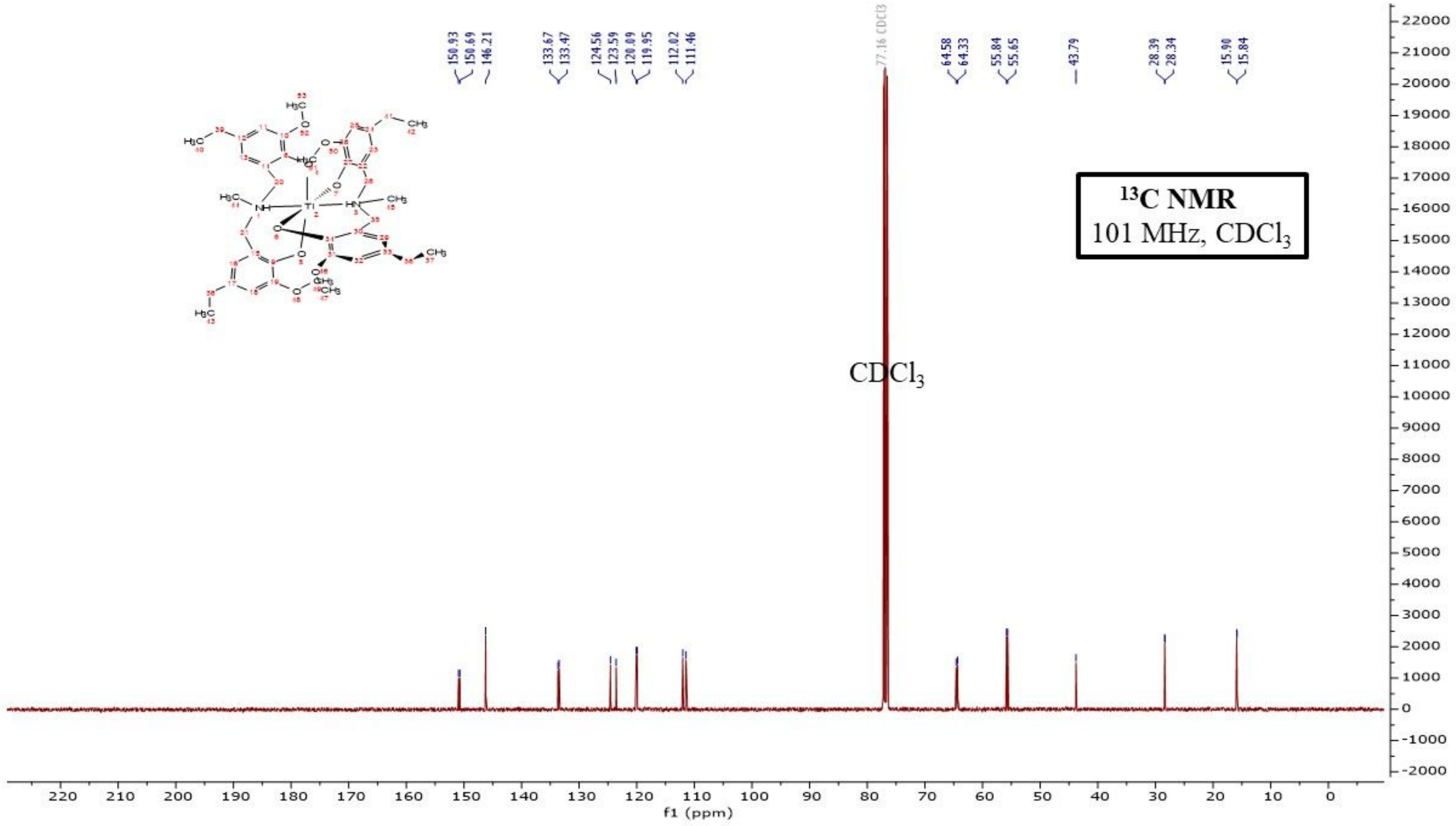

Figure S27. Representative ^{13}C NMR spectrum of final complex *bis*((2,2'-((methylimino-*N*)*bis*(methylene))*bis*(4-ethyl-6-methoxyphenolato-*O*)))titanium(IV) (**3b**).

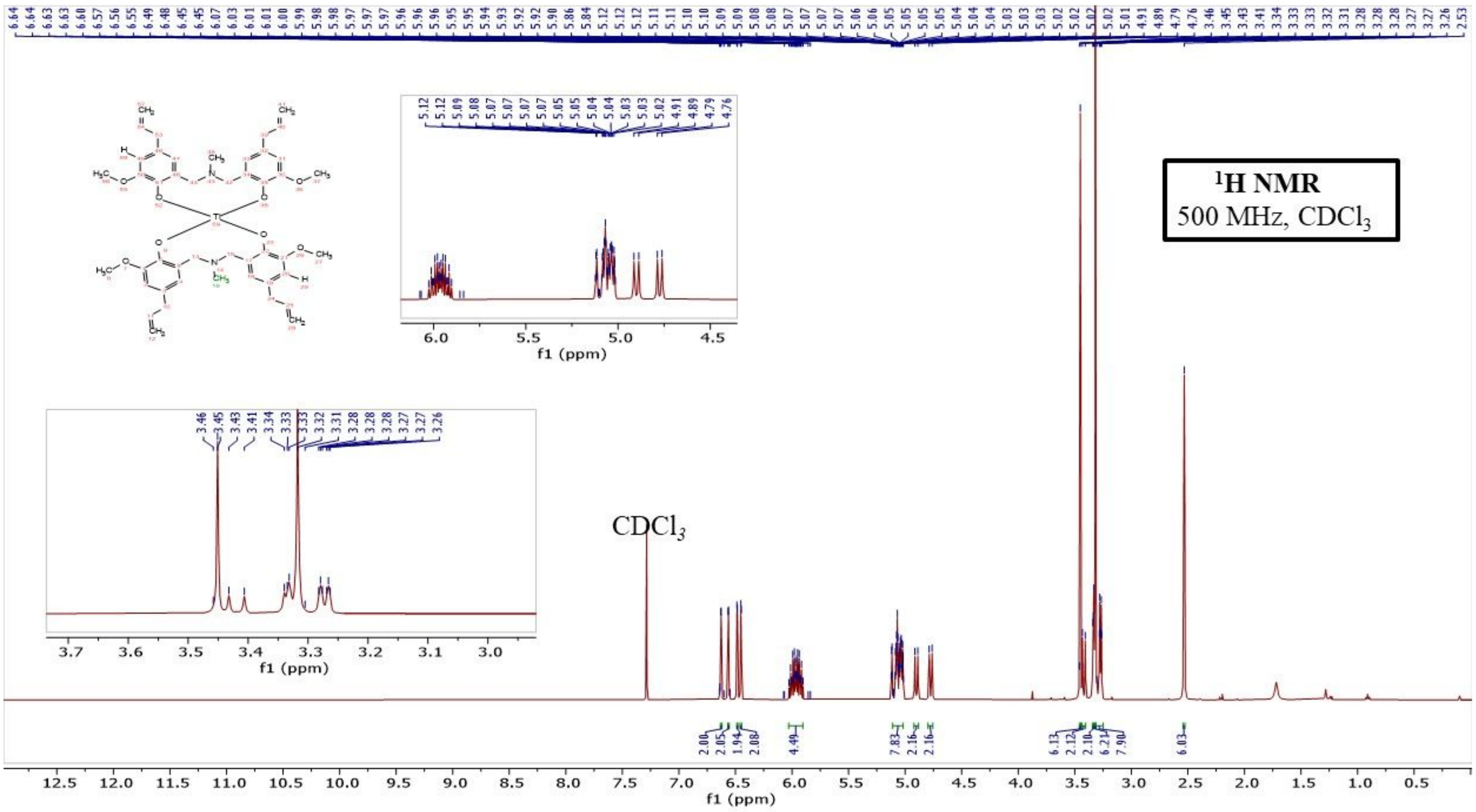

Figure S28. Representative ^{1}H NMR spectrum of final complex *bis*((2,2'-((methylimino-*N*)*bis*(methylene))*bis*(4-allyl-6-methoxyphenolato-*O*)))titanium(IV) (**3c**); expansions inserted.

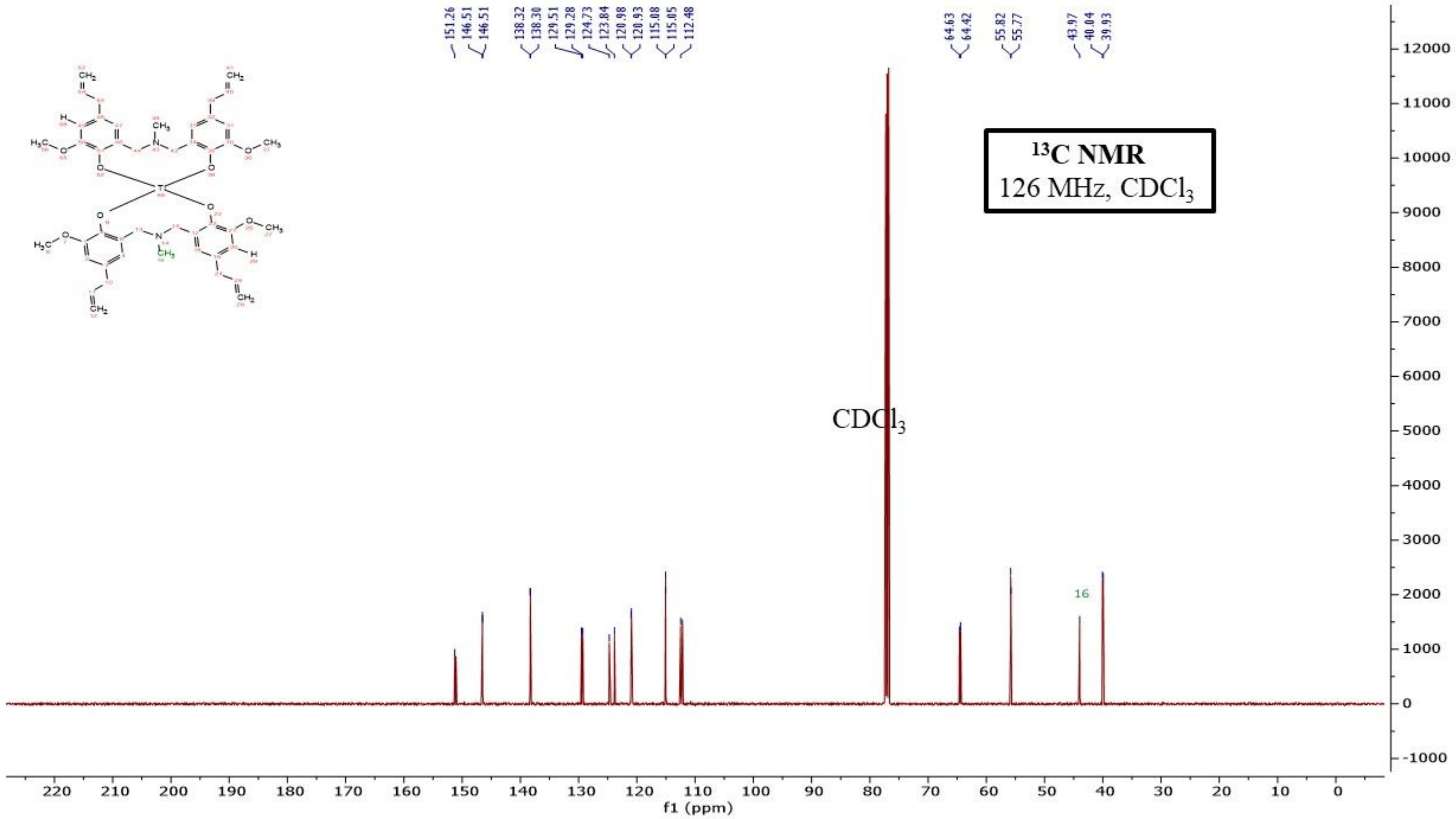

Figure S29. Representative ^{13}C NMR spectrum of final complex *bis*((2,2'-((methylimino-*N*)*bis*(methylene))*bis*(4-allyl-6-methoxyphenolato-*O*)))titanium(IV) (**3c**).

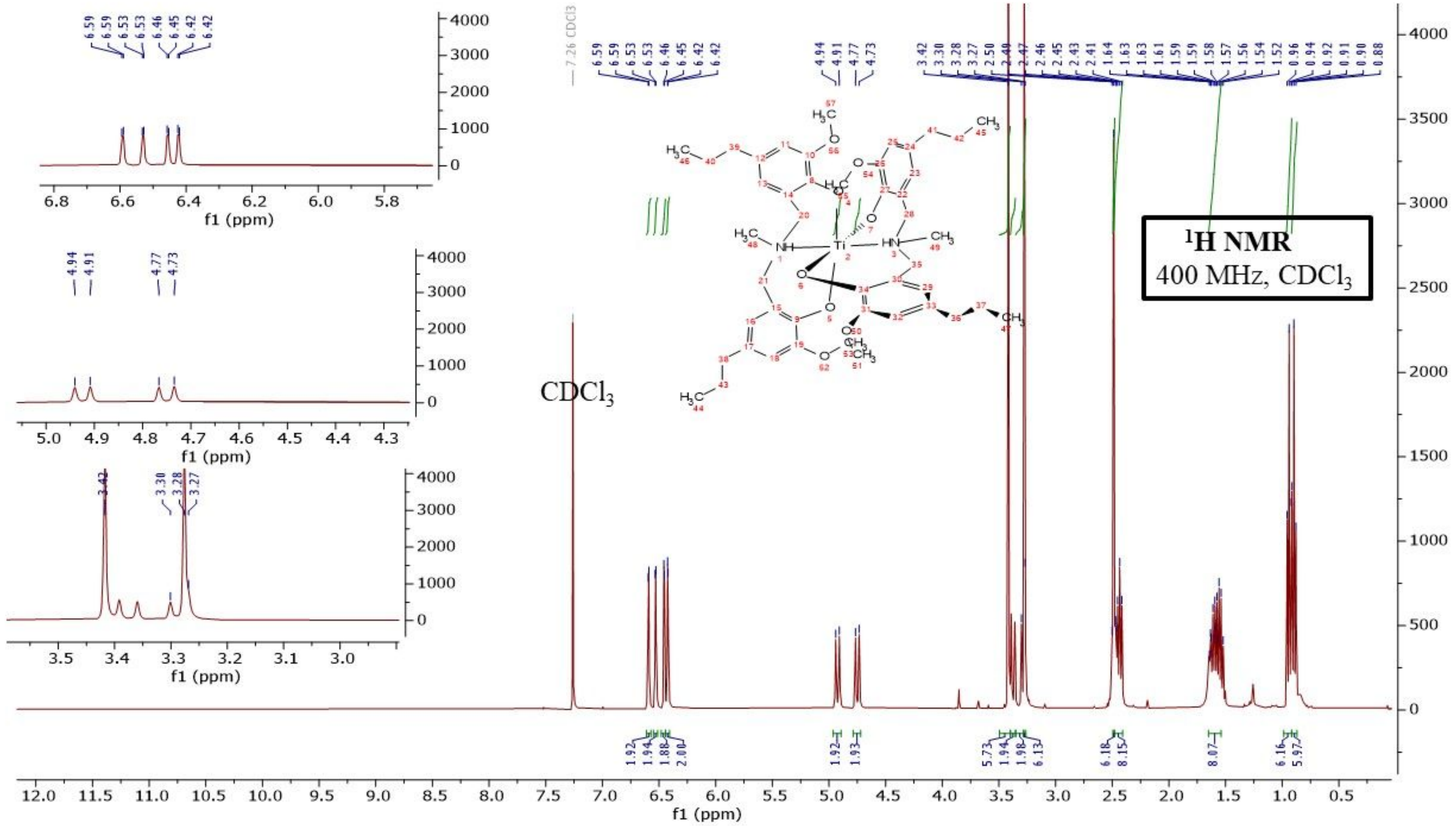

Figure S30. *Representative* ^{1}H NMR spectrum of final complex *bis*((2,2'-((methylimino-*N*)*bis*(methylene))*bis*(4-*n*-propyl-6-methoxyphenolato-*O*)))titanium(IV) (**3d**); expansions inserted.

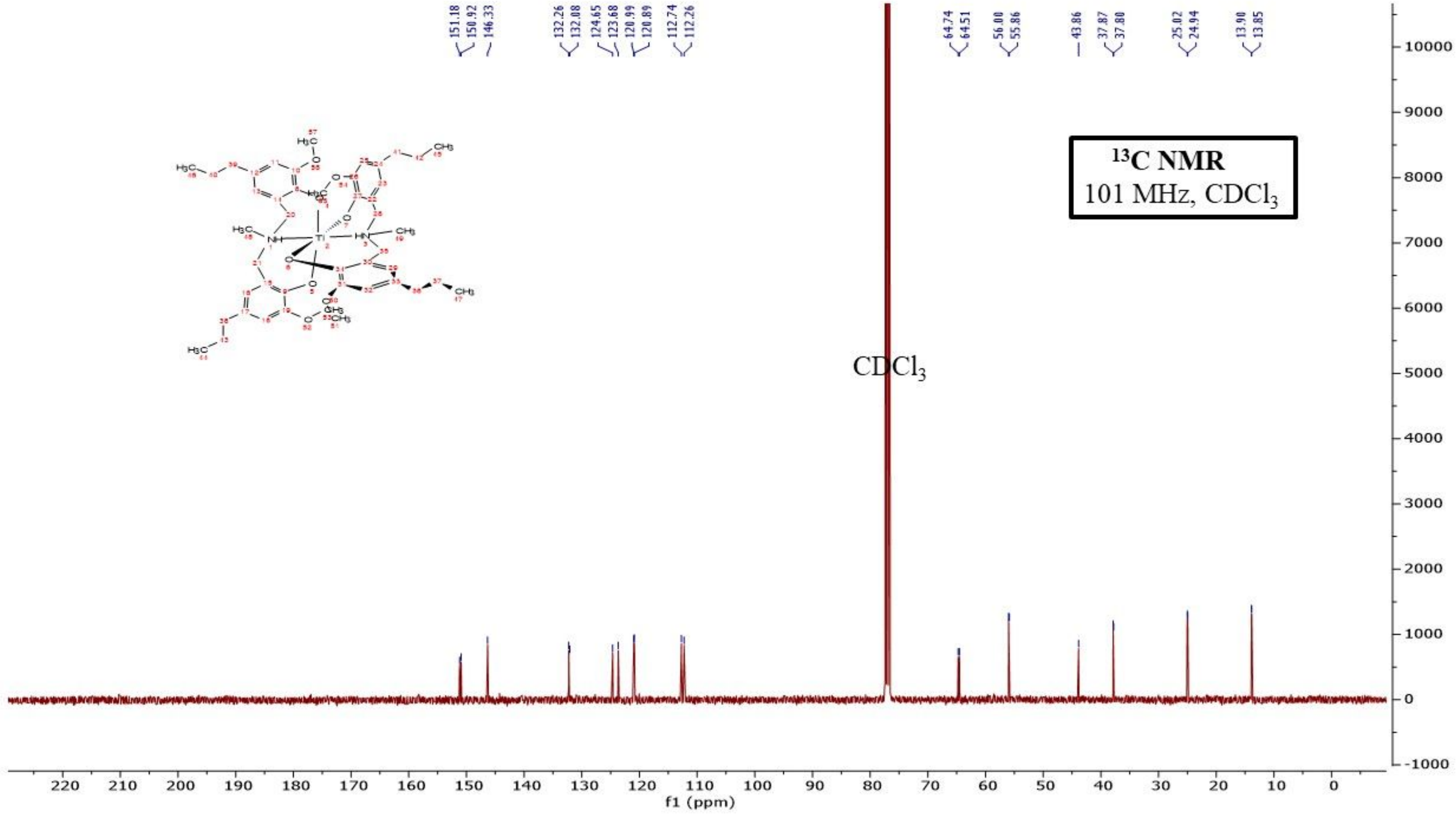

Figure S31. Representative ^{13}C NMR spectrum of final complex *bis*((2,2'-((methylimino-*N*)*bis(*methylene))*bis*(4-propyl-6-methoxyphenolato-*O*)))titanium(IV) (**3d**).

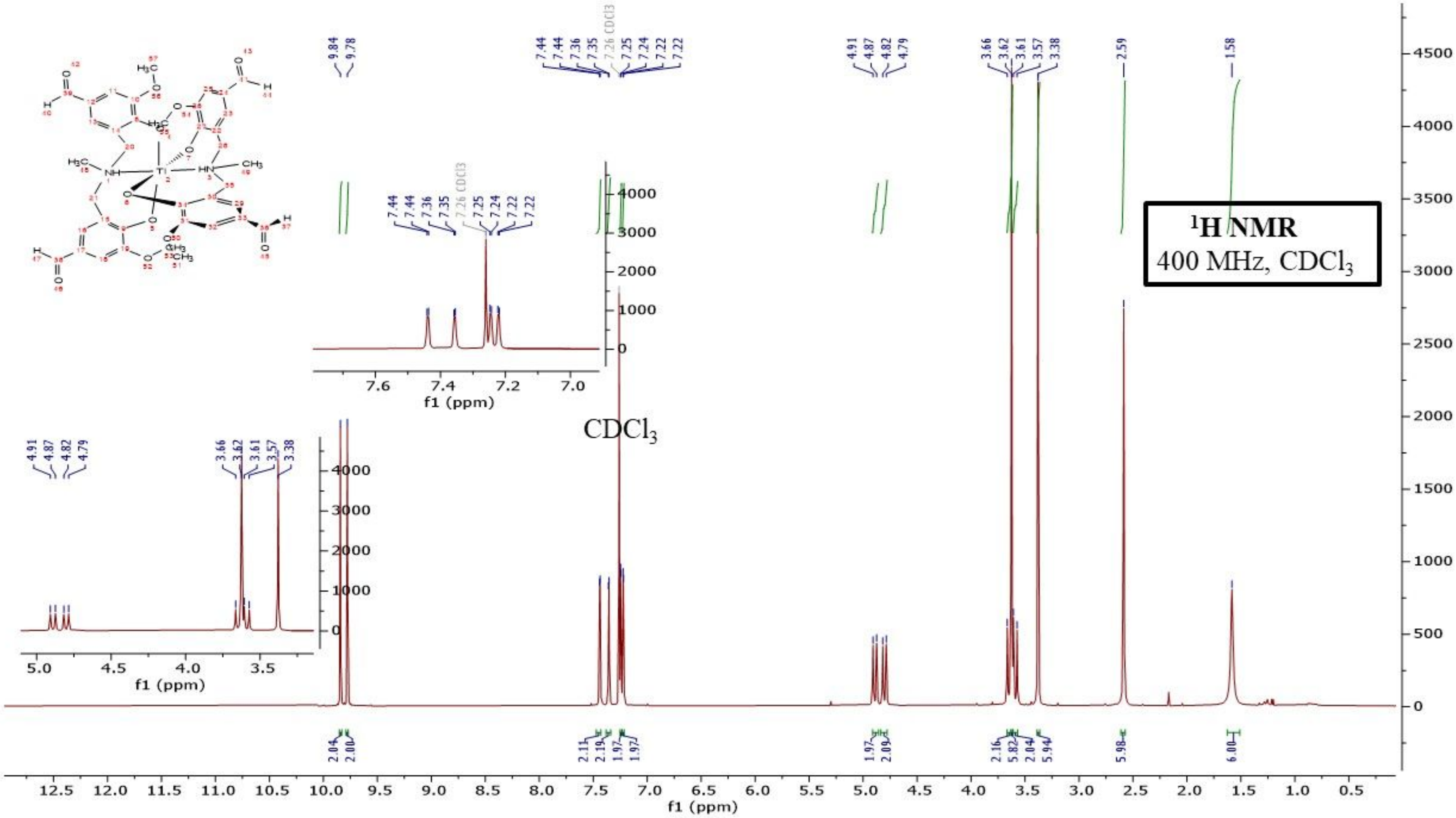

Figure S32. Representative ^{1}H NMR spectrum of final complex *bis*((2,2’-((methylimino-*N*)*bis*(methylene))*bis*(4-formyl-6-methoxyphenolato-*O*)))titanium(IV) (**3e**); expansions inserted.

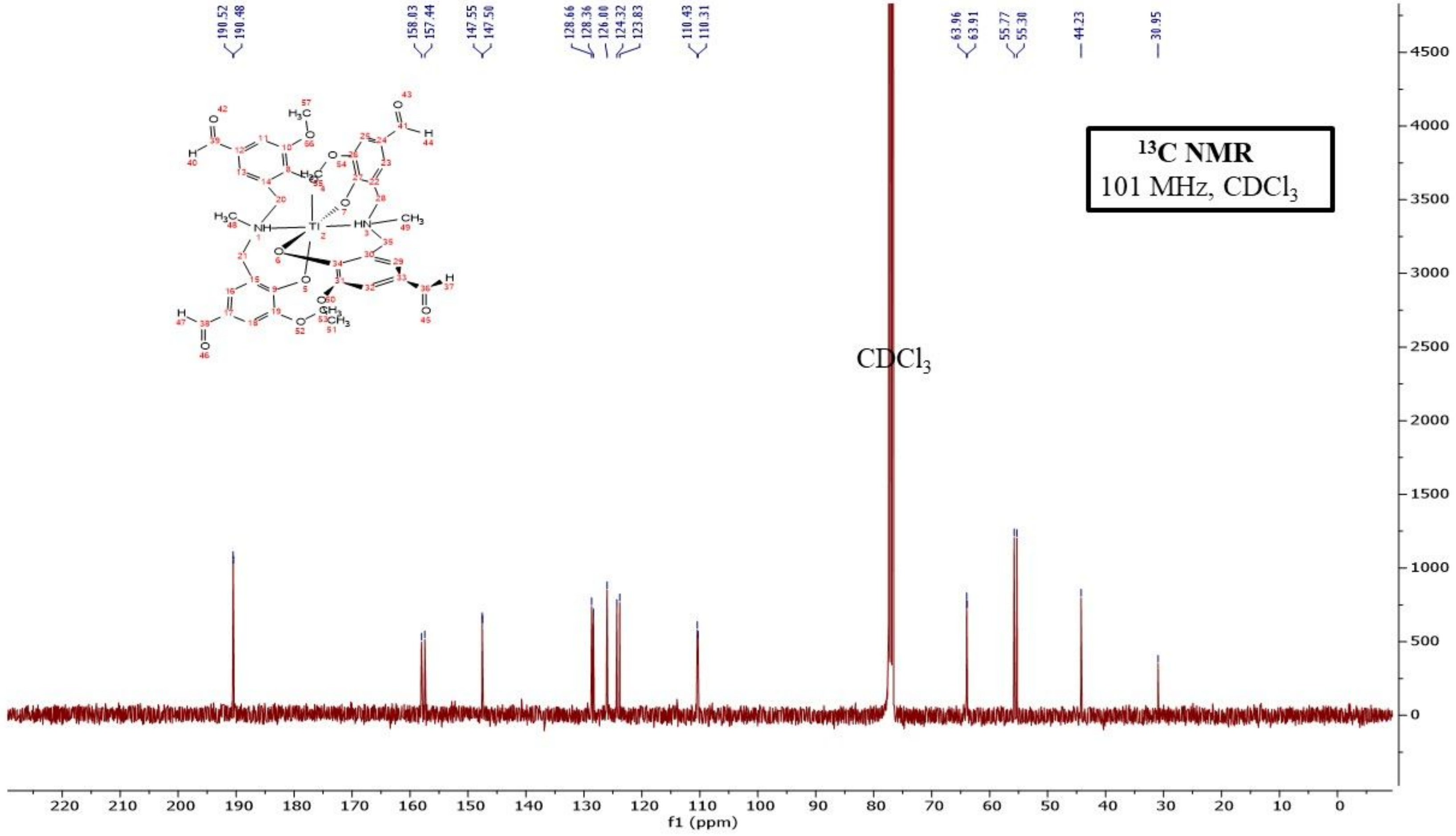

Figure S33. Representative ^{13}C NMR spectrum of final complex *bis*((2,2'-((methylimino-*N*)*bis*(methylene))*bis*(4-formyl-6-methoxyphenolato-*O*)))titanium(IV) (**3e**).

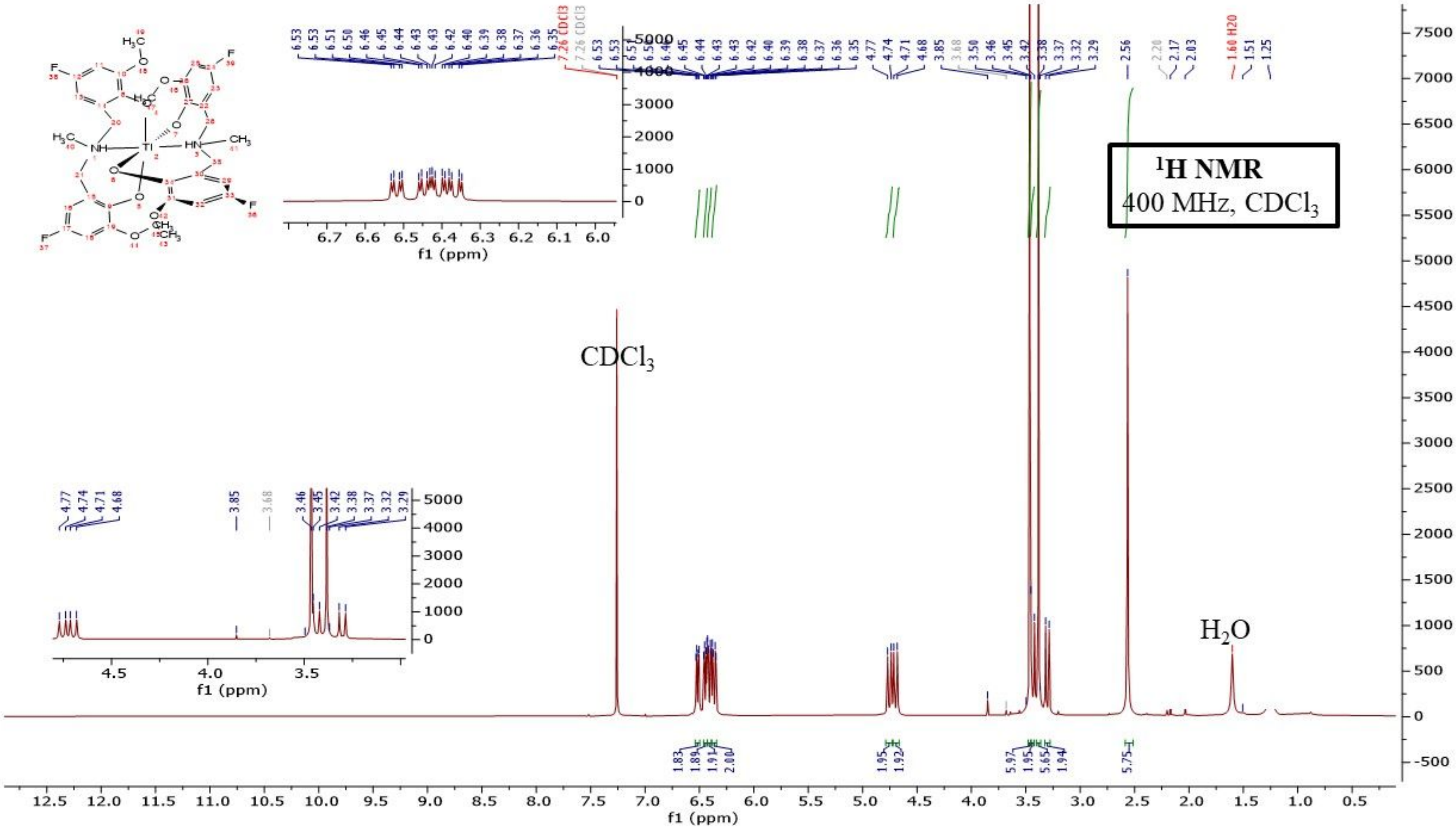

Figure S34. Representative ^{1}H NMR spectrum of final complex *bis*((2,2'-((methylimino-*N*)*bis*(methylene))*bis*(4-fluoro-6-methoxyphenolato-*O*)))titanium(IV) (**3f**); expansions inserted.

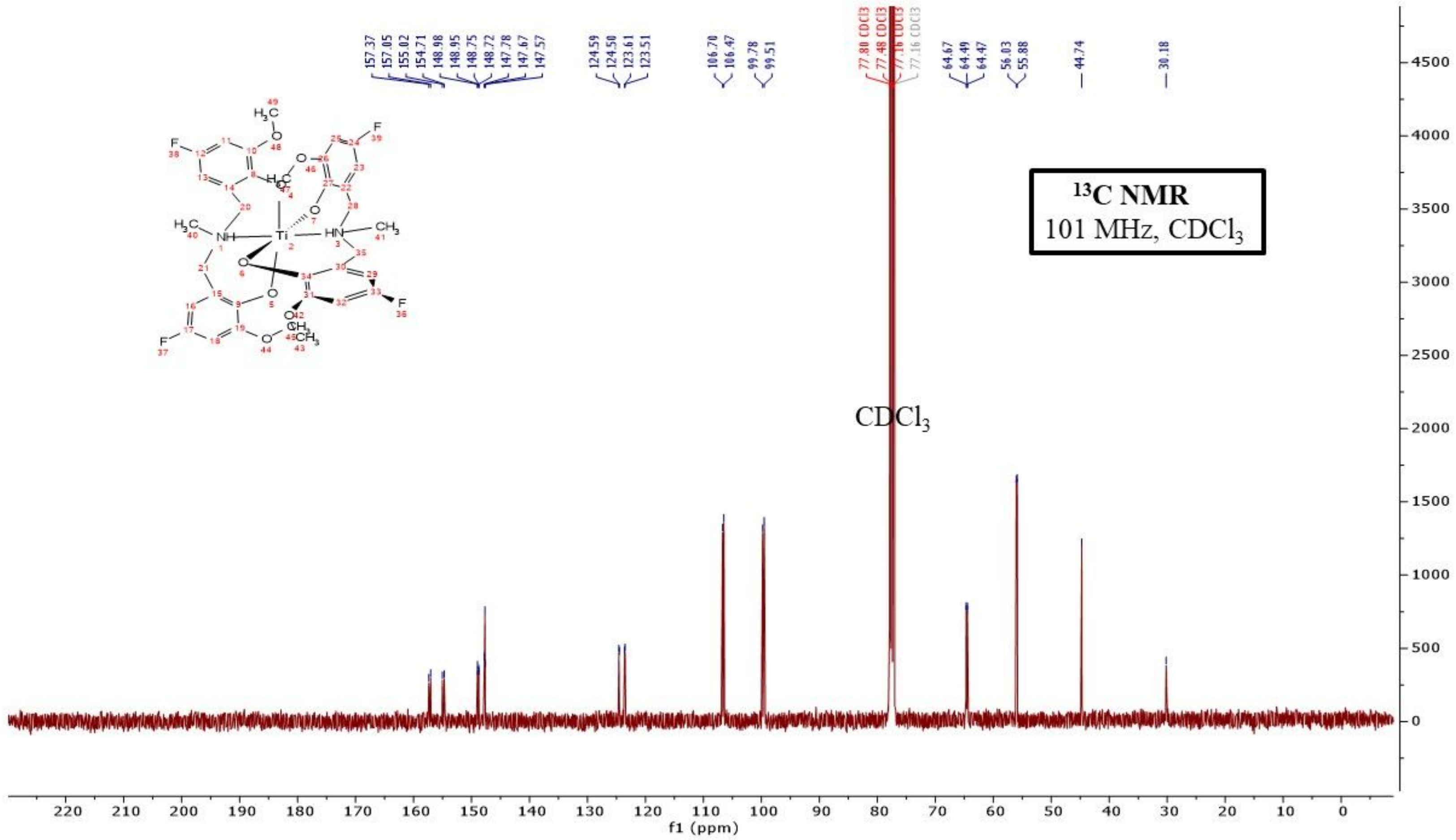

Figure S35. Representative ^{13}C NMR spectrum of final complex *bis*((2,2'-((methylimino-*N*)*bis*(methylene))*bis*(4-fluoro-6-methoxyphenolato-*O*)))titanium(IV) (**3f**).

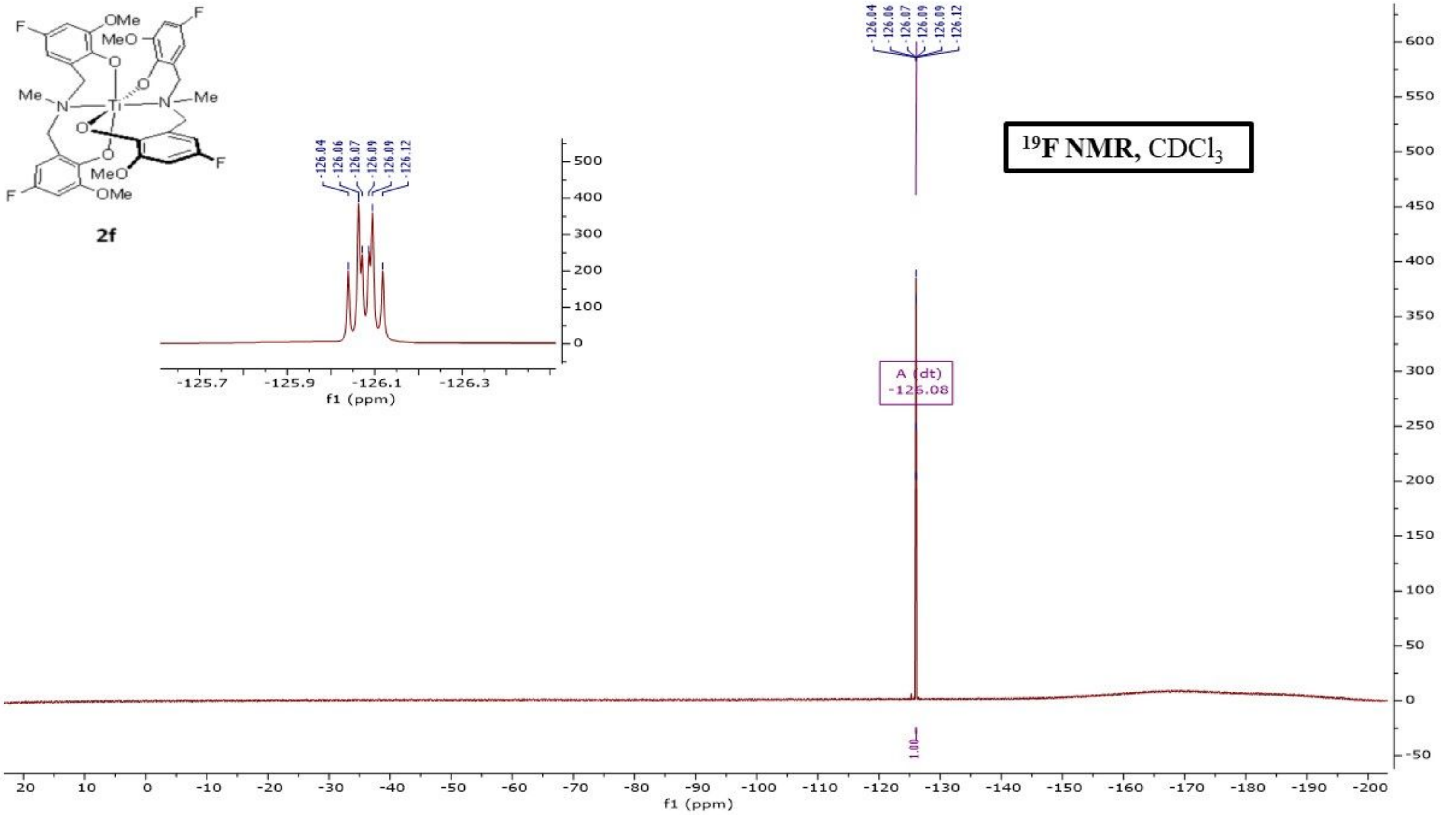

Figure S36. Representative ^{19}F NMR spectrum of final complex *bis*((2,2'-((methylimino-*N*)*bis*(methylene))*bis*(4-fluoro-6-methoxyphenolato-*O*)))titanium(IV) (**3f**); coupled spectrum inserted.

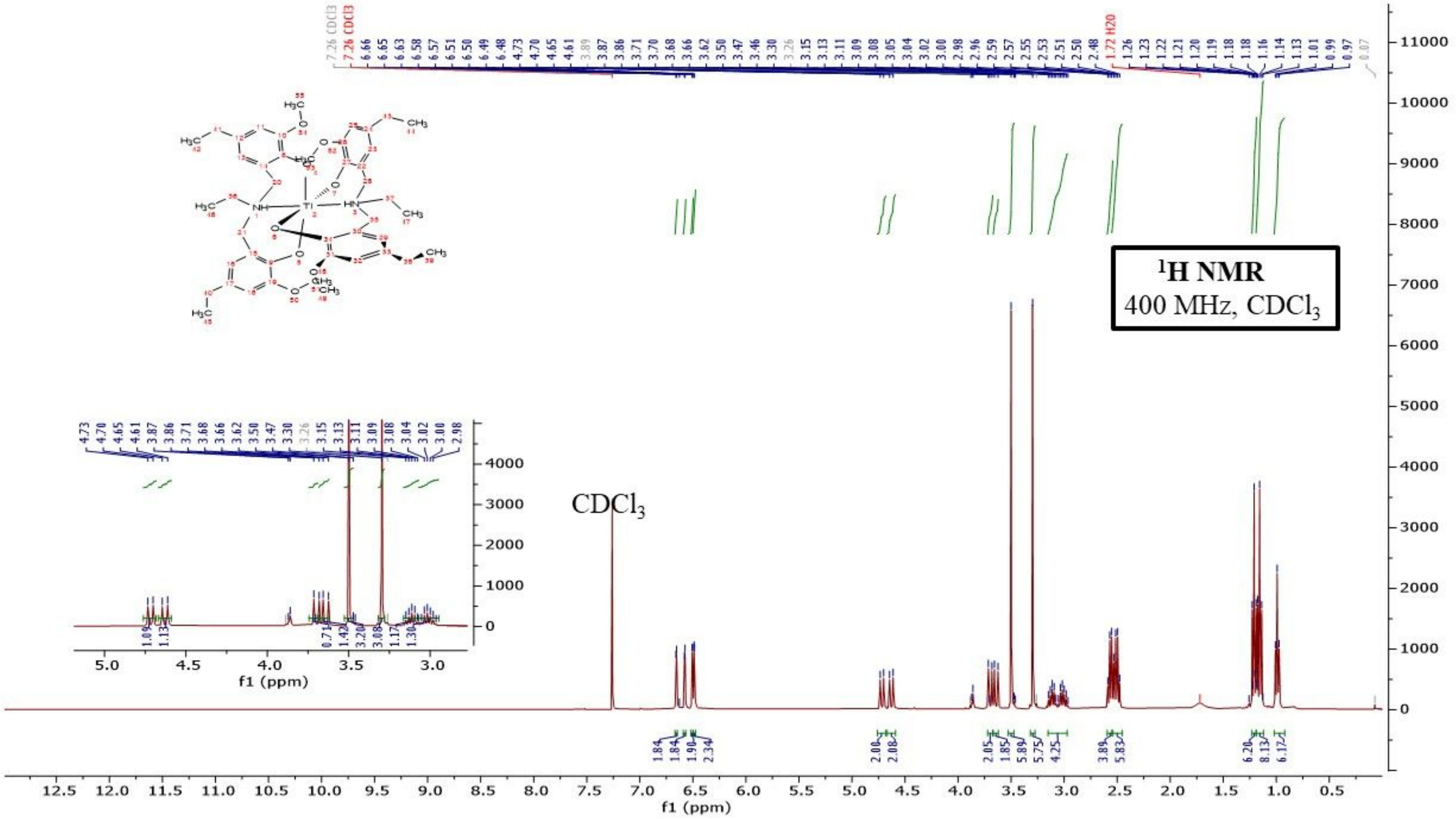

Figure S37. Representative ^{1}H NMR spectrum of final complex *bis*((2,2'-((ethylimino-*N*)*bis*(methylene))*bis*(4-ethyl-6-methoxyphenolato-*O*)))titanium(IV) (**3g**); expansions inserted.

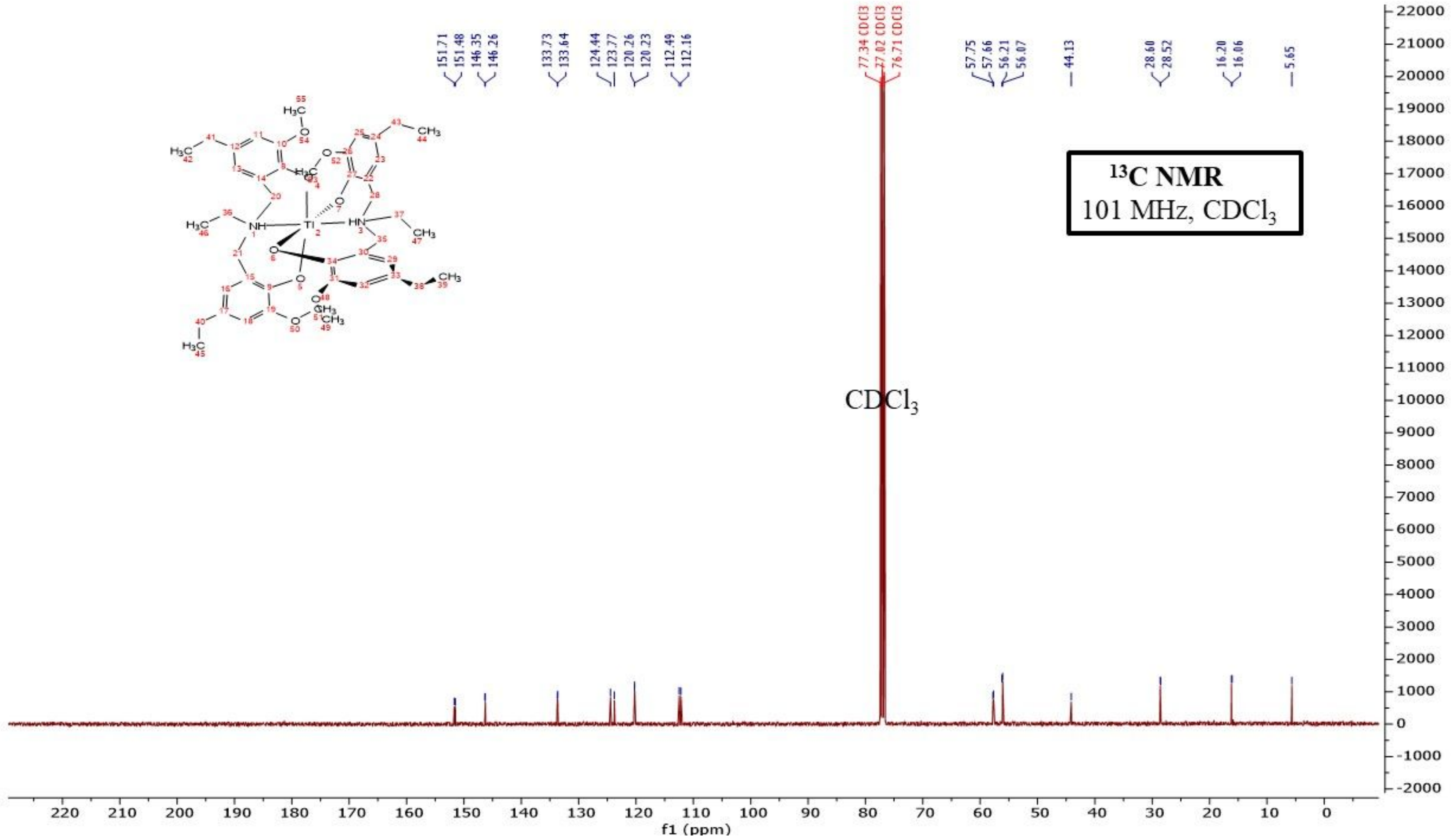

Figure S38. Representative ^{13}C NMR spectrum of final complex *bis*((2,2'-((ethylimino-*N*)*bis*(methylene))*bis*(4-ethyl-6-methoxyphenolato-*O*)))titanium(IV) (**3g**).

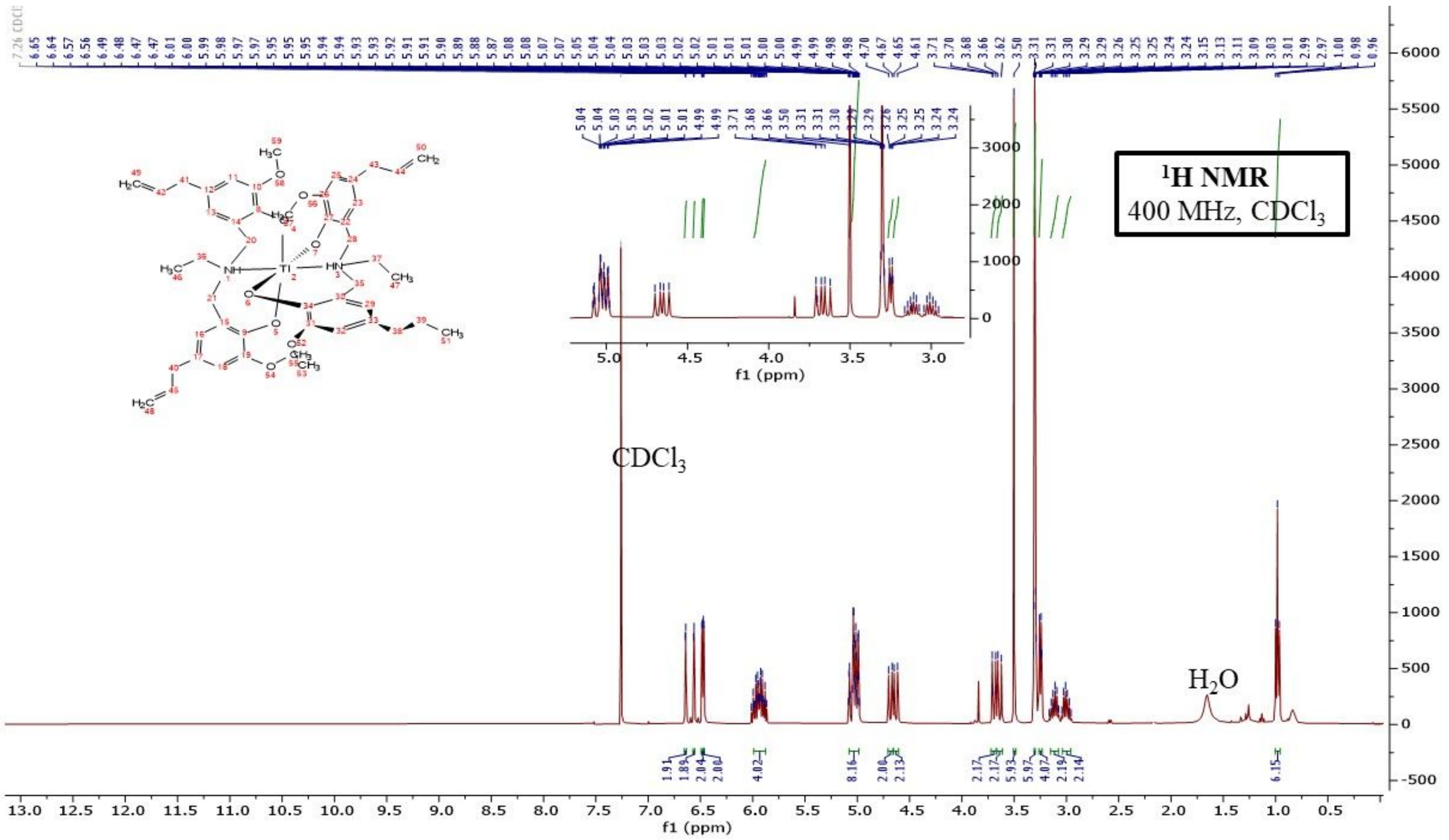

Figure S39. Representative ^{1}H NMR spectrum of final complex *bis*((2,2'-((ethylimino-*N*)*bis*(methylene))*bis*(4-allyl-6-methoxyphenolato-*O*)))titanium(IV) (**3h**); expansion inserted.

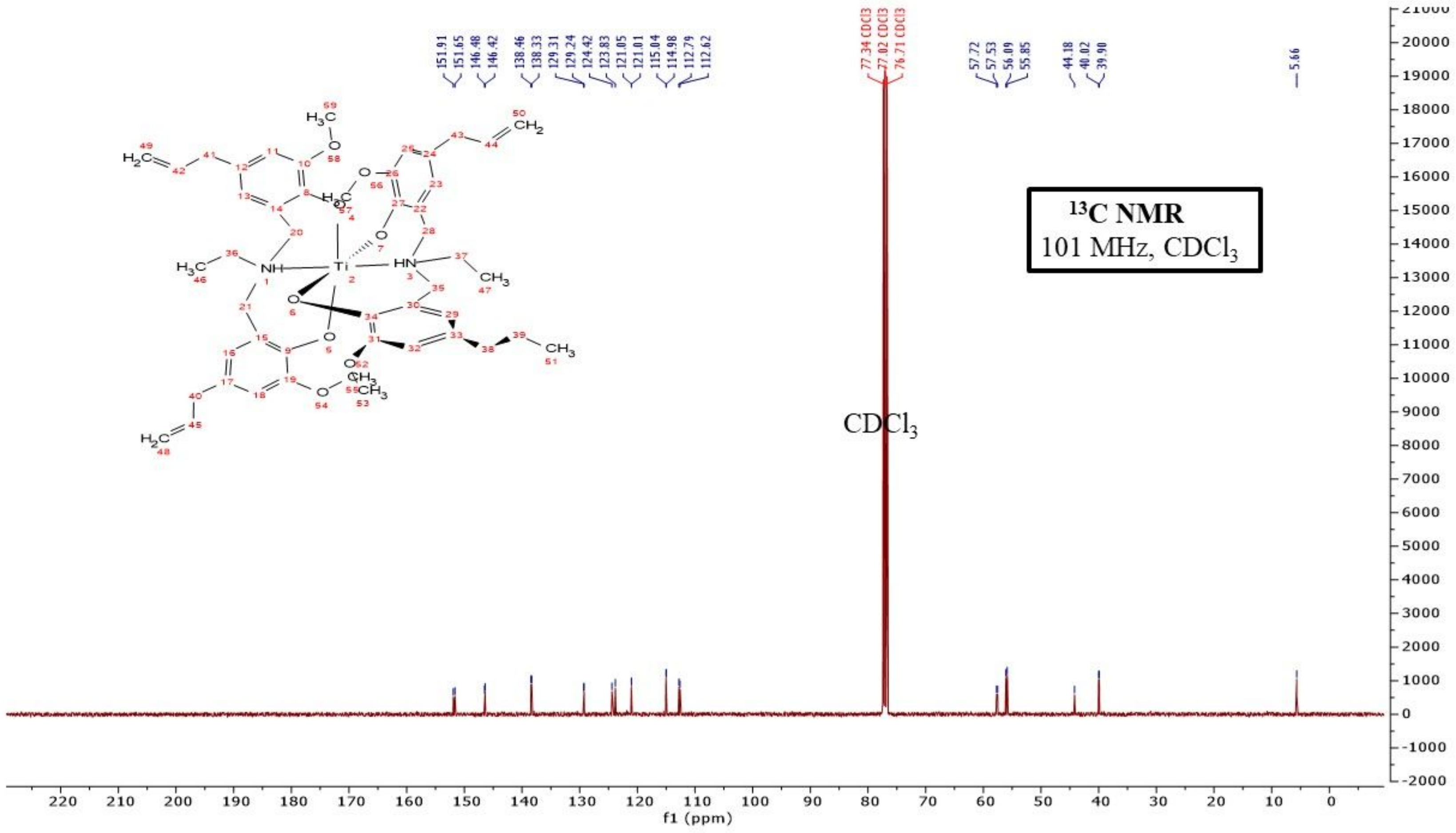

Figure S40. Representative ^{13}C NMR spectrum of final complex *bis*((2,2'-((ethylimino-*N*)*bis*(methylene))*bis*(4-allyl-6-methoxyphenolato-*O*)))titanium(IV) (**3h**).

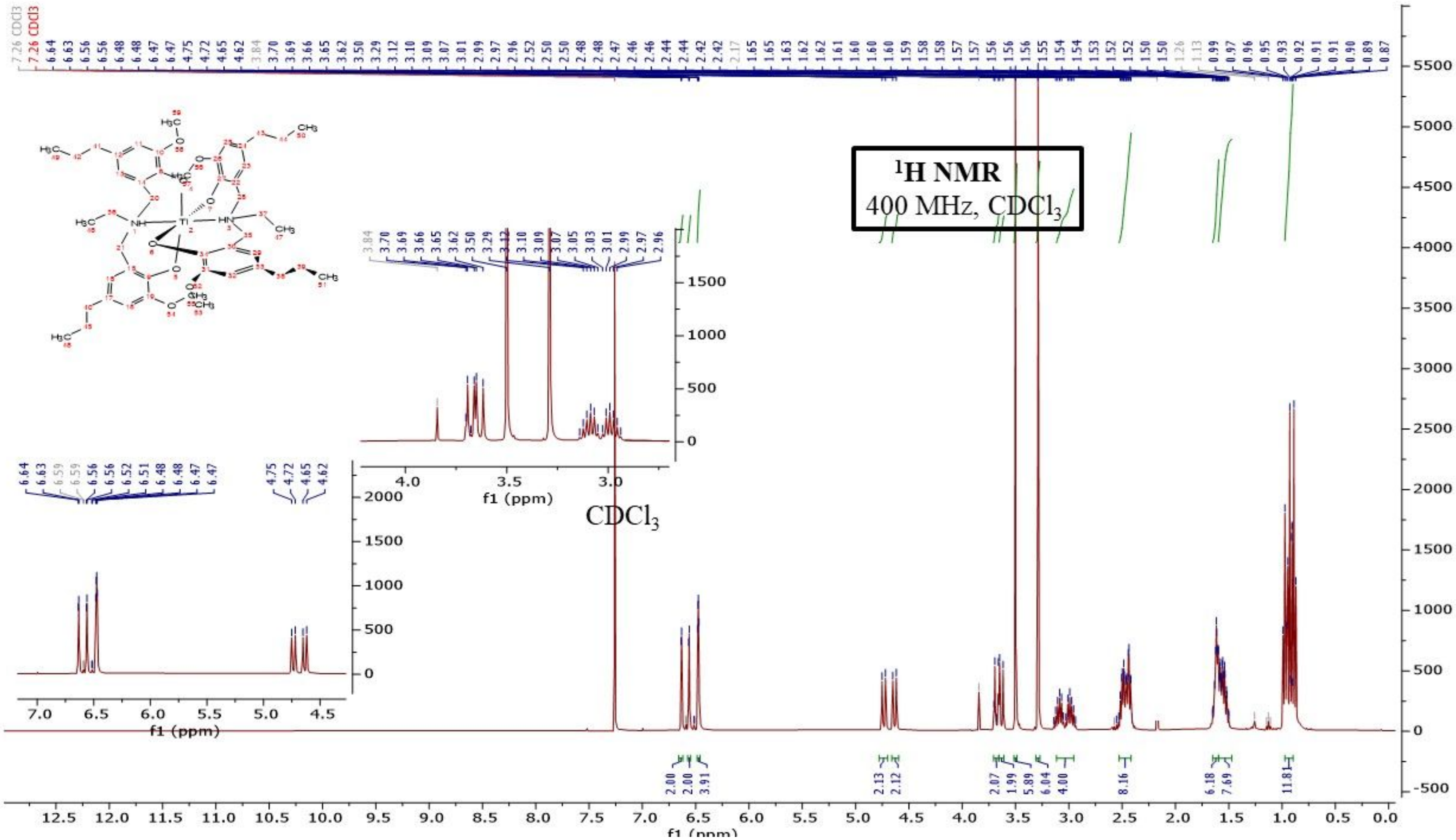

Figure S41. Representative ^{1}H NMR spectrum of final complex *bis*((2,2'-((ethylimino-*N*)*bis*(methylene))*bis*(4-*n*-propyl-6-methoxyphenolato-*O*)))titanium(IV) (**3i**); expansions inserted.

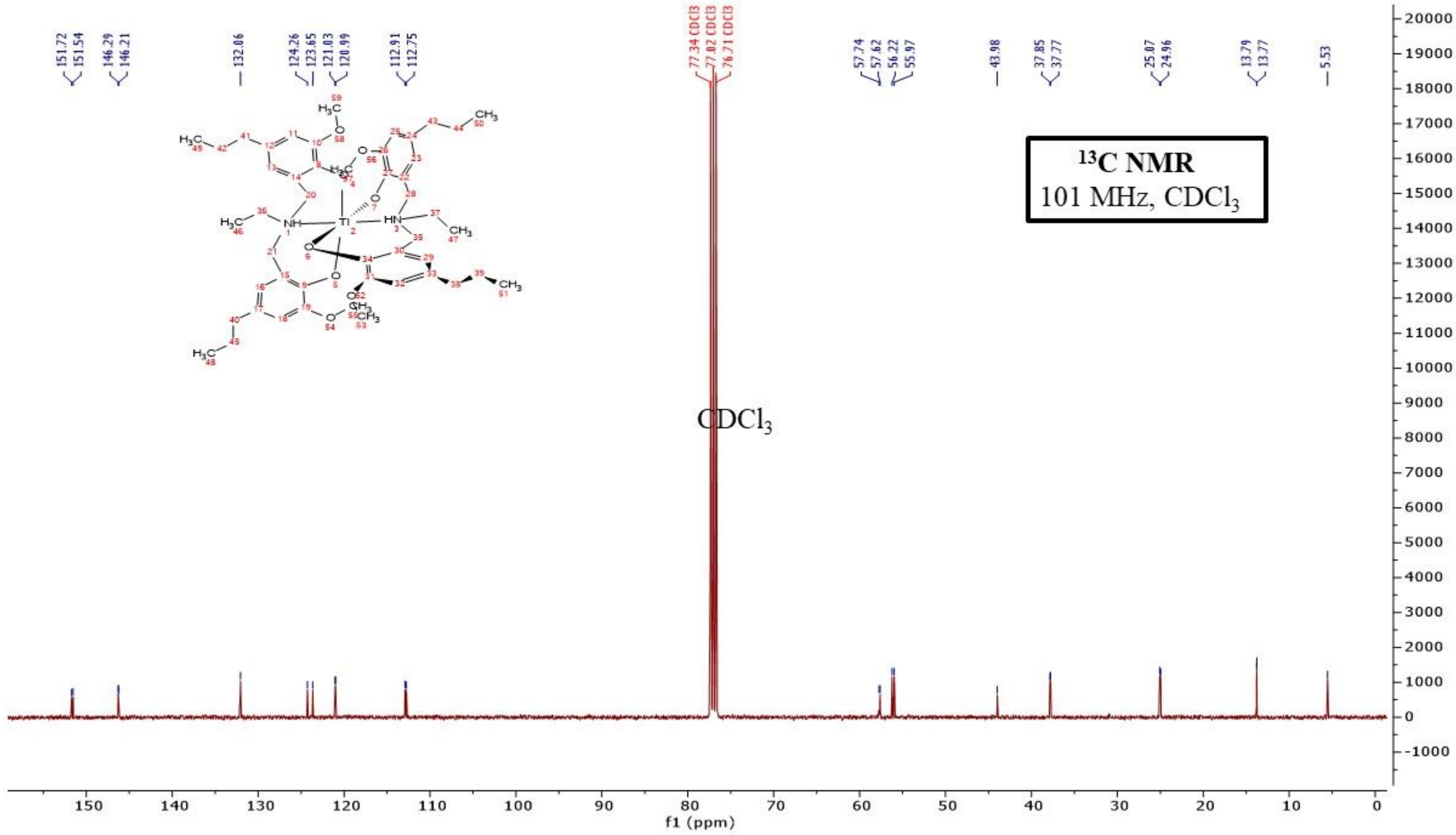

Figure S42. Representative ^{13}C NMR spectrum of final complex *bis*((2,2’-((ethylimino-*N*)*bis*(methylene))*bis*(4-*n*-propyl-6-methoxyphenolato-*O*)))titanium(IV) (**3i**).

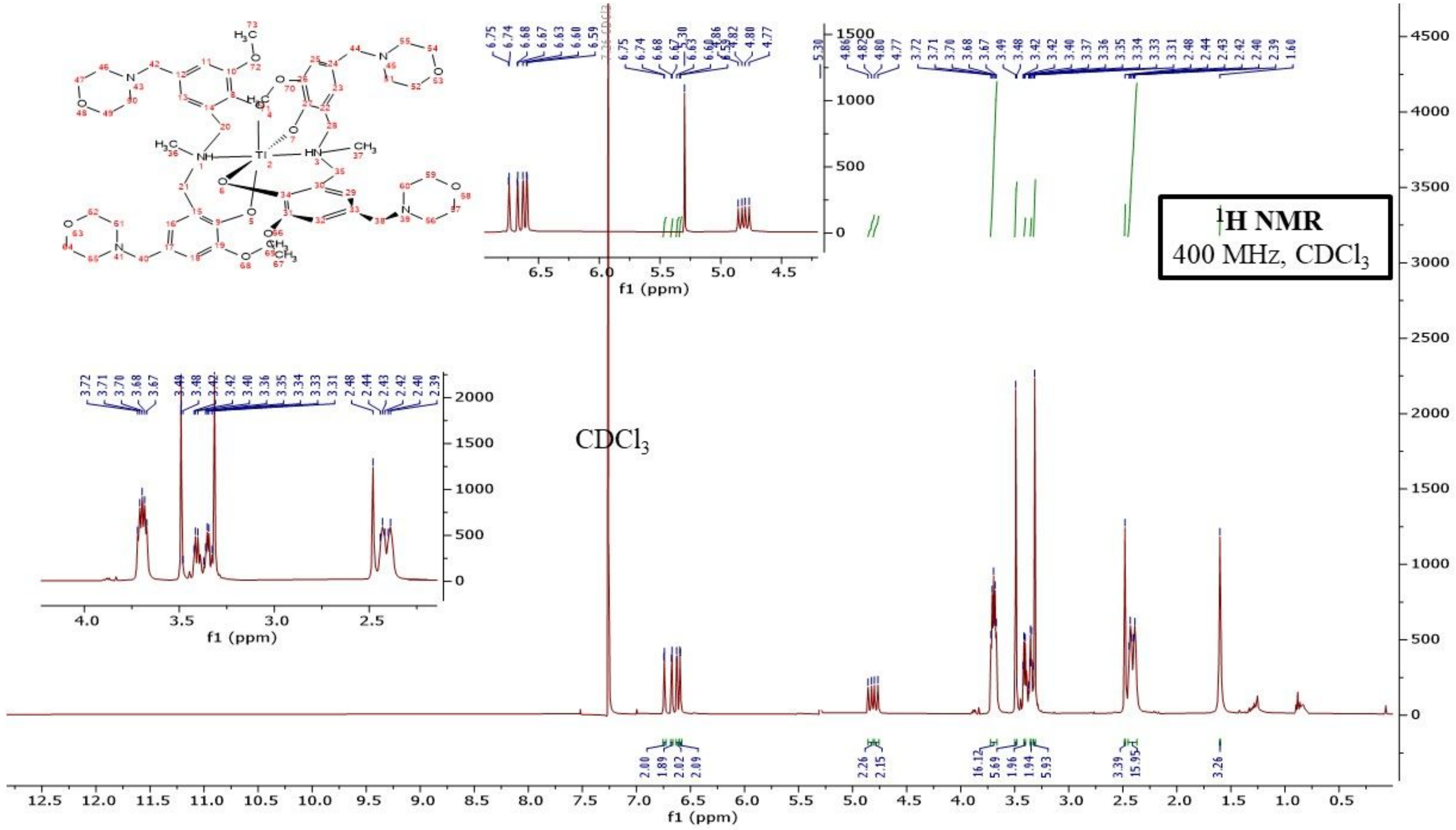

Figure S43. Representative ^{1}H NMR spectrum of final complex *bis*((2,2'-((methylimino-*N*)*bis*(methylene))*bis*(4-morpholinomethyl-6-methoxyphenolato-*O*)))titanium(IV) (**3j**); expansions inserted.

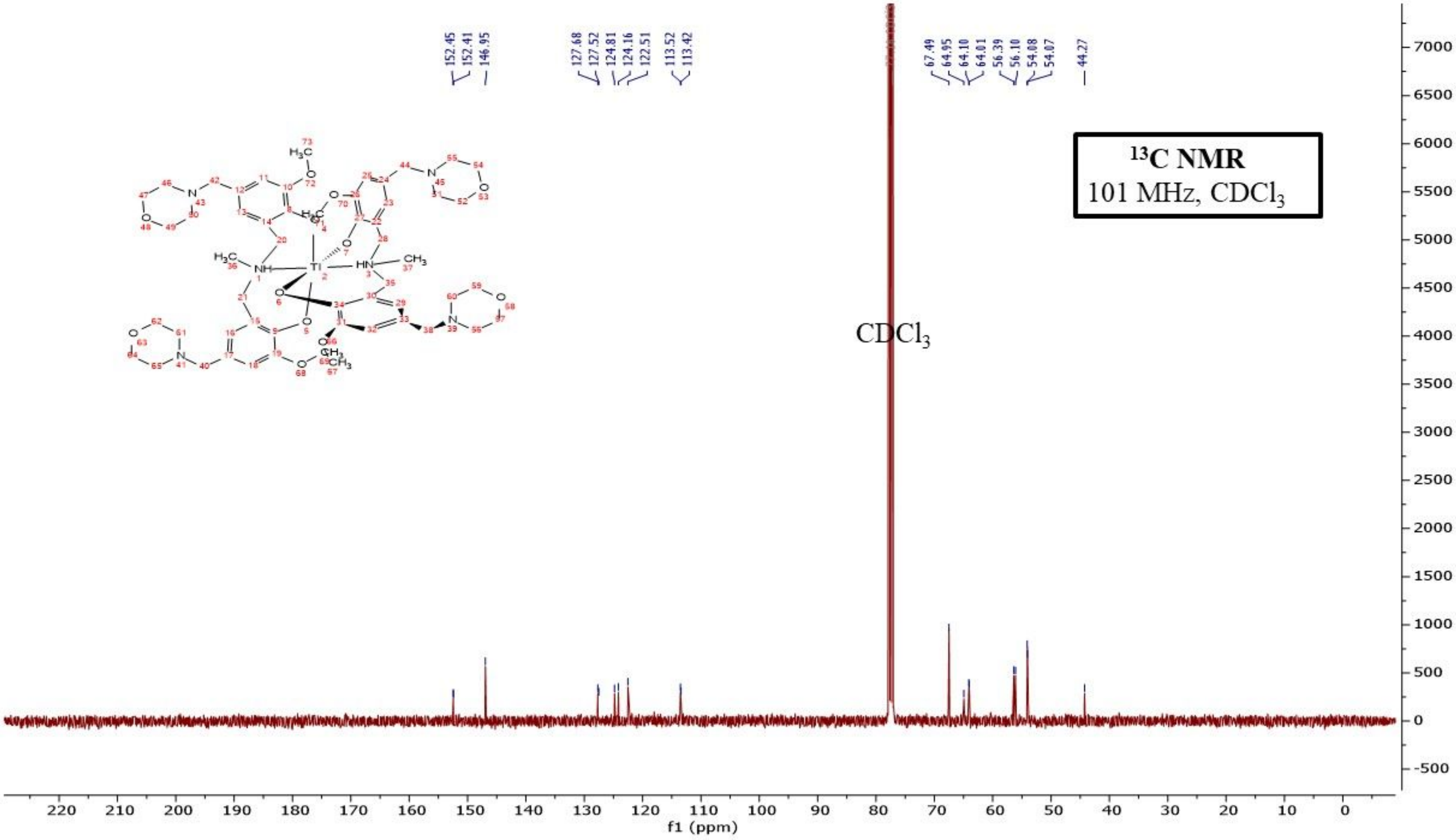

Figure S44. Representative ^{13}C NMR spectrum of final complex *bis*((2,2'-((methylimino-*N*)*bis*(methylene))*bis*(4-morpholinomethyl-6-methoxyphenolato-*O*)))titanium(IV) (**3j**).

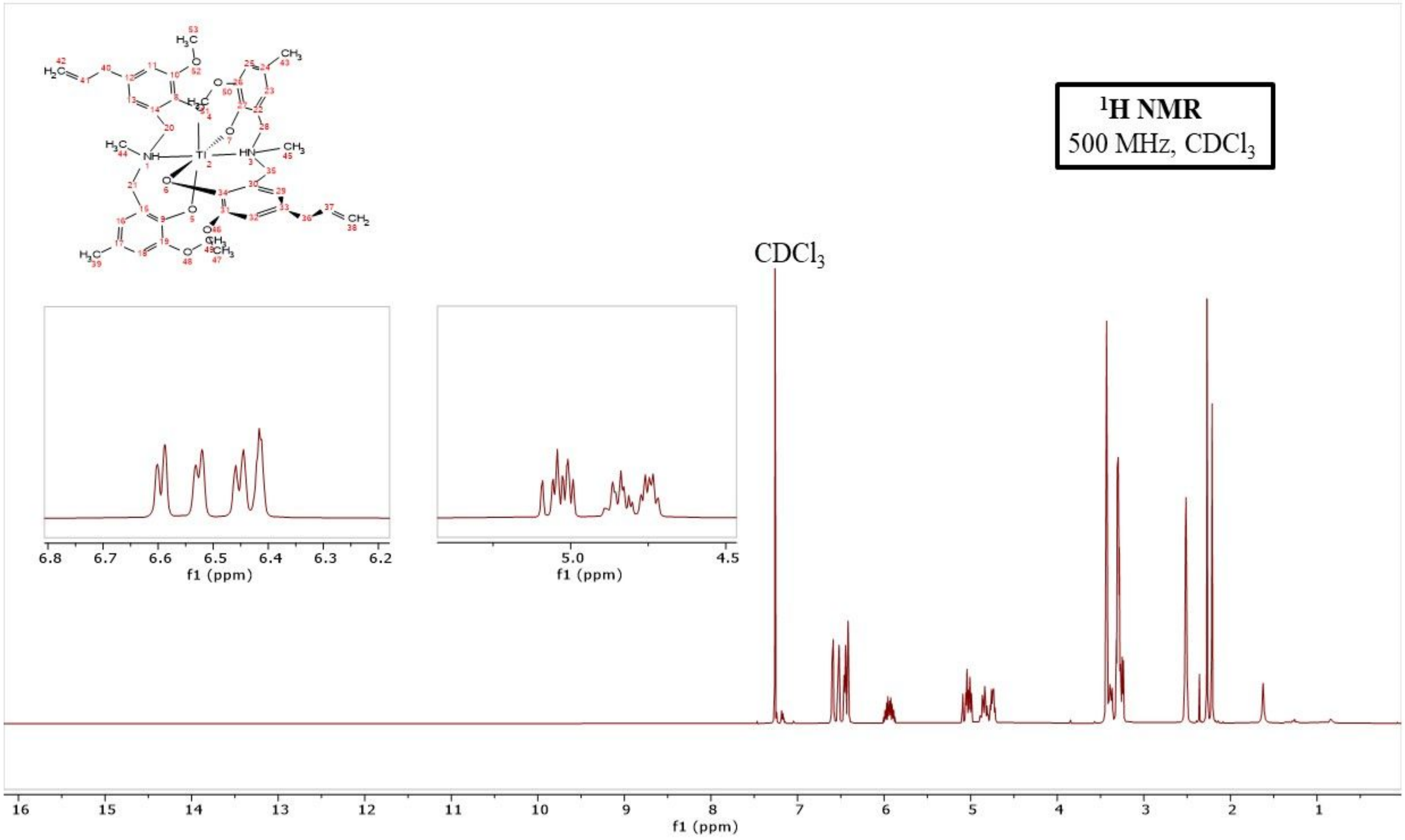

Figure S45. Representative ^{1}H NMR spectrum of final complex *bis*((2,2'-((methylimino-*N*)*bis*(methylene))*bis*(4-allyl-4-methyl-6,6-dimethoxyphenolato-*O*)))titanium(IV) (**3k**); expansions inserted.

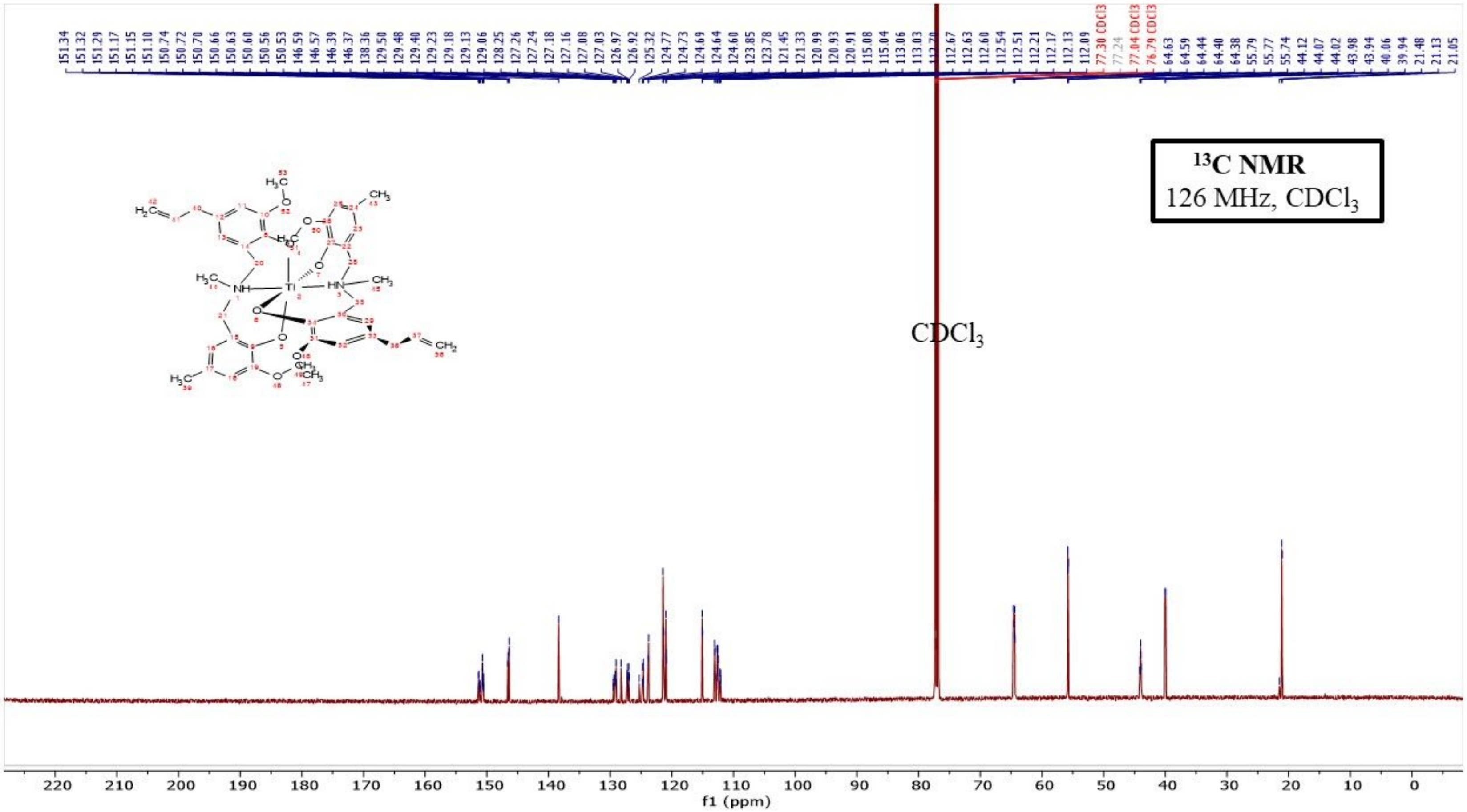

Figure S46. Representative ^{13}C NMR spectrum of final complex *bis*((2,2'-((methylimino-*N*)*bis*(methylene))*bis*(4-allyl-4-methyl-6,6-dimethoxyphenolato-*O*)))titanium(IV) (**3k**).

■ 2. X-ray crystallographic studies

Crystals of compound (**3b**), suitable for X-ray diffraction, were grown by liquid-liquid diffusion of pentane into diethyl ether.

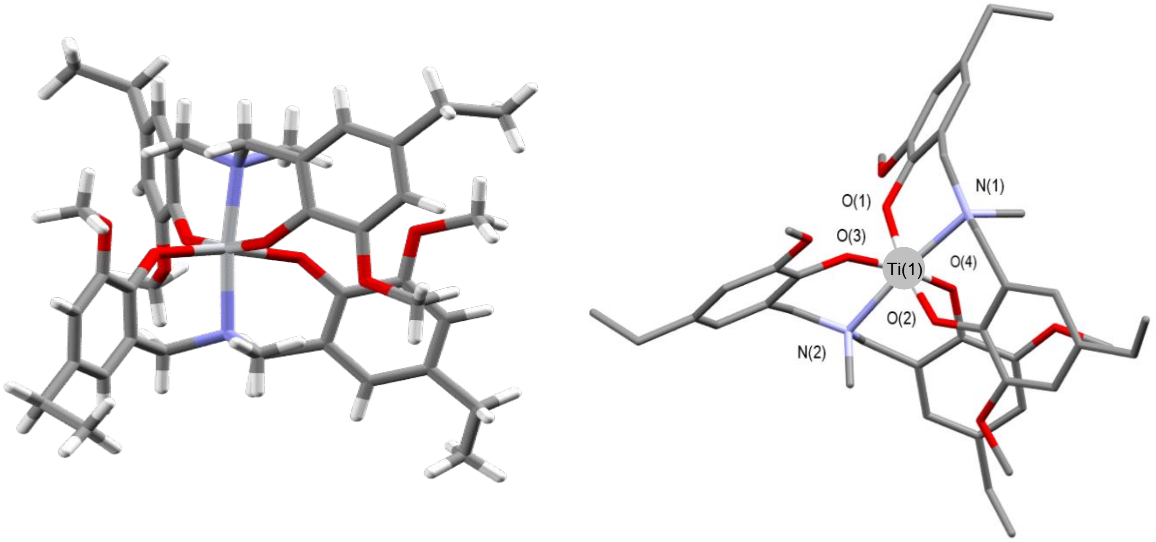

Figure S47. The solid-state structure of compound (**3b**), the right-hand view shows the atom numbering used in Table S1. One of two independent molecules in the unit cell.

Crystal System: Triclinic
Space group: $P2_1/c$
Unit Cell Parameters: a = 10.1867(4) Å, b = 19.4486(9) Å, c = 20.2864(9) Å, β = 88.322(3)°, V = 3982.5(3) $Å^3$, Z = 4, R-Factor = 3.48%.

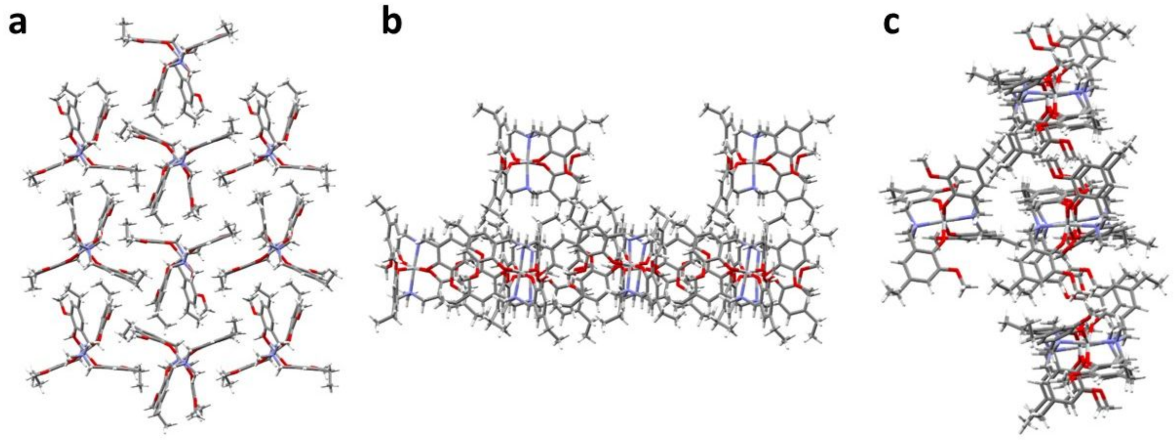

Figure S48. Partial solid-state packing structures of (**3b**). Projections are viewed along the crystallographic (a) *a*-, (b) *b*-, and (c) *c*-axes of the unit cell.

Crystals of compound (**3c**), suitable for X-ray diffraction, were grown by liquid-liquid diffusion of pentane into diethyl ether.

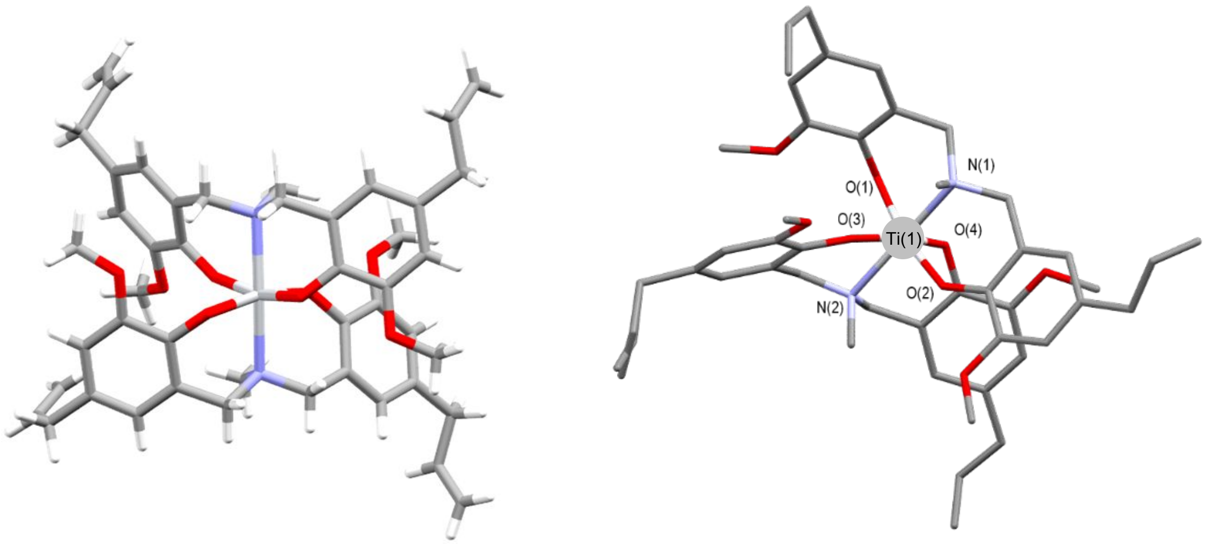

Figure S49. The solid-state structure of compound (**3c**), the right-hand view shows the atom numbering used in Table S1.

Crystal System: Monoclinic
Space group: $P2_1/c$
Unit Cell Parameters: *a* = 17.7695(3) Å, *b* = 10.8588(2) Å, *c* = 25.0228(4) Å, *β* = 90°, *V* = 5699.5 Å^3, *Z* = 4 R-Factor = 3.79%.

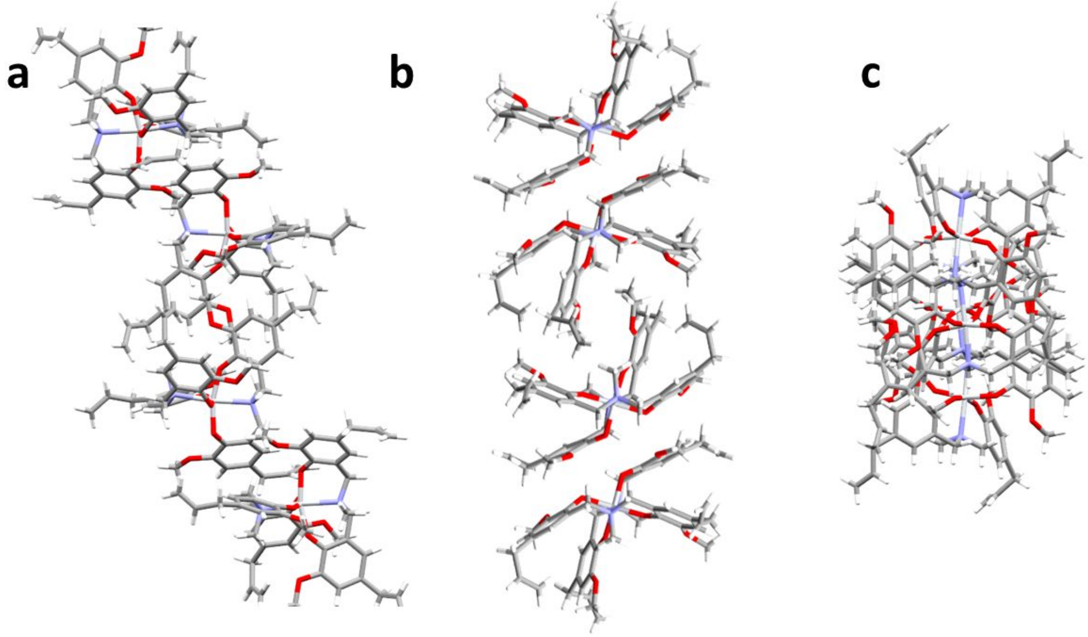

Figure S50. Partial solid-state packing structures of (**3c**). Projections are viewed along the crystallographic (a) *a*-, (b) *b*-, and (c) *c*-axes of the unit cell.

Crystals of compound (**3i**), suitable for X-ray diffraction, were grown by liquid-liquid diffusion of pentane into diethyl ether.

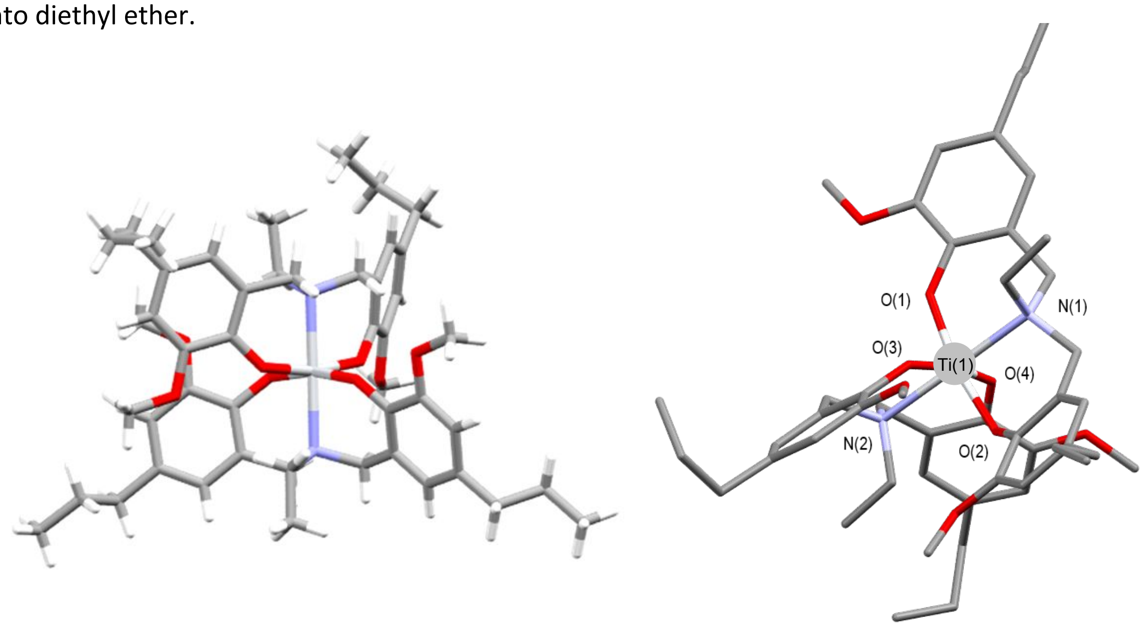

Figure S51*.* The solid-state structure of compound (**3i**), the right-hand view shows the atom numbering used in Table S1.

Crystal System: Monoclinic
Space group: $P2_1/c$
Unit Cell Parameters: a = 13.2724(2) Å, b = 10.03840(10) Å, c = 31.5999(3) Å, β = 101.6410(10)°, V = 4123.57 Å^3, Z = 4 R-Factor = 3.04%.

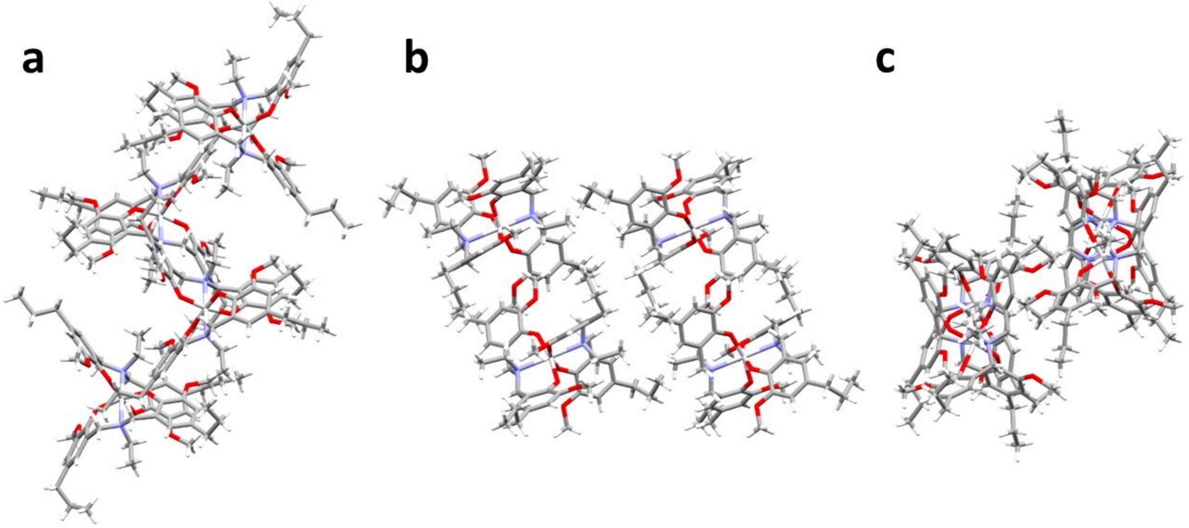

Figure S52. Partial solid-state packing structures of (**3i**). Projections are viewed along the crystallographic (a) *a*-, (b) *b*-, and (c) *c*-axes of the unit cell.

Crystals of (**3j**), suitable for X-ray diffraction, were grown by liquid-liquid diffusion of pentane into CH_2Cl_2.

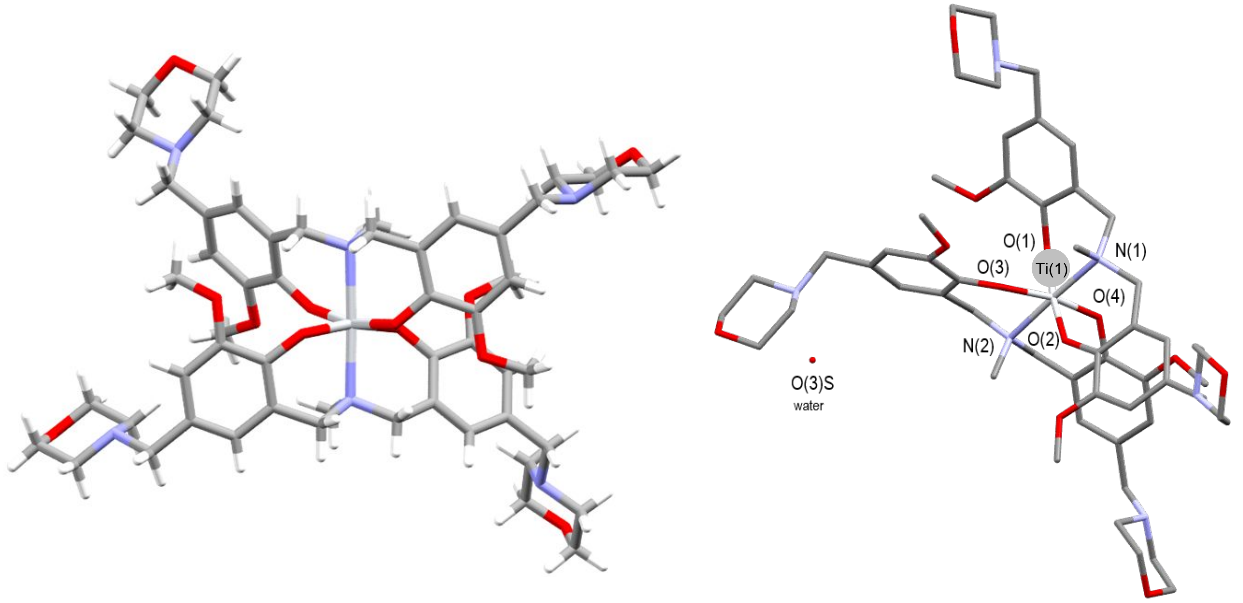

Figure S53. The solid-state structure of compound (**3j**), the right-hand view shows the atom numbering used in Table S1. Crystallized from CH_2Cl_2/pentane the unit cell of (**3j**) contains both a dichloromethane and water molecule. The latter is proximal to one of the morpholine rings $N_{morpholine}$....O(3)S ~2.88 Å.

Crystal System: Orthorhombic
Space group: $Pna2_1$
Unit Cell Parameters: a = 13.1875(2) Å, b = 14.0667(2) Å, c = 30.7243(6) Å, β = 108.860(2)°, V = 4569.06 $Å^3$, Z = 4 R-Factor = 4.73%.

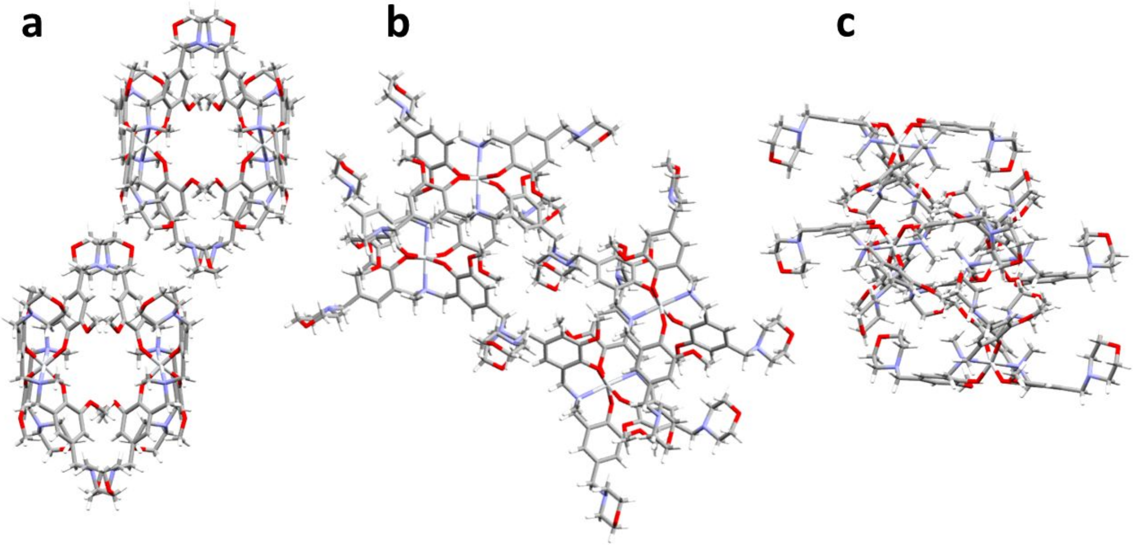

■ 2.1 Hirshfeld Surface Analysis

Figure S54. Partial solid-state packing structures of (**3j**). Projections are viewed along the crystallographic (a) *a*-, (b) *b*-, and (c) *c*-axes of the unit cell.

Hirshfeld surfaces[3] were employed to provide insights into the intermolecular interactions between crystal molecules in the crystal lattice. We calculated Hirshfeld surfaces of: (**3b**) (Figure S55), (**3c**) (Figure S56) (**3d**) (Figure S57), and (**3j**) (Figure S58), in Crystal Explorer17[4] using an isovalue of 0.5 and mapping

the normalized contact distance, d_{norm}. The surfaces highlight in red any regions in which the molecular surfaces meet at distances shorter than the sum of the Van der Waals radii, while white and blue illustrate regions where they meet at distances that are the sum of the van der Waals radii or longer, respectively.

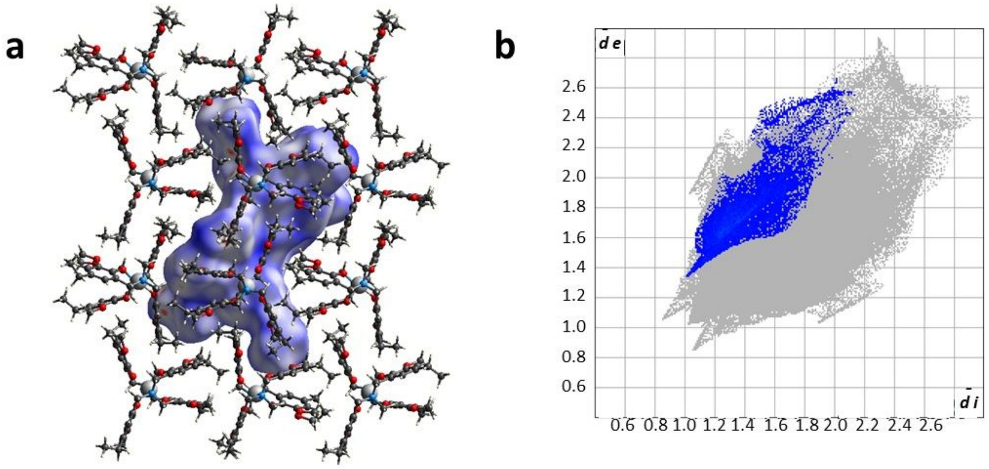

Figure S55. Hirshfeld surfaces of (**3b**). a) Surface Plot. b) Fingerprint highlights of closest contact between the Internal surface plot (d_i) (Hydrogen) and external fragments (d_e) (Oxygen).

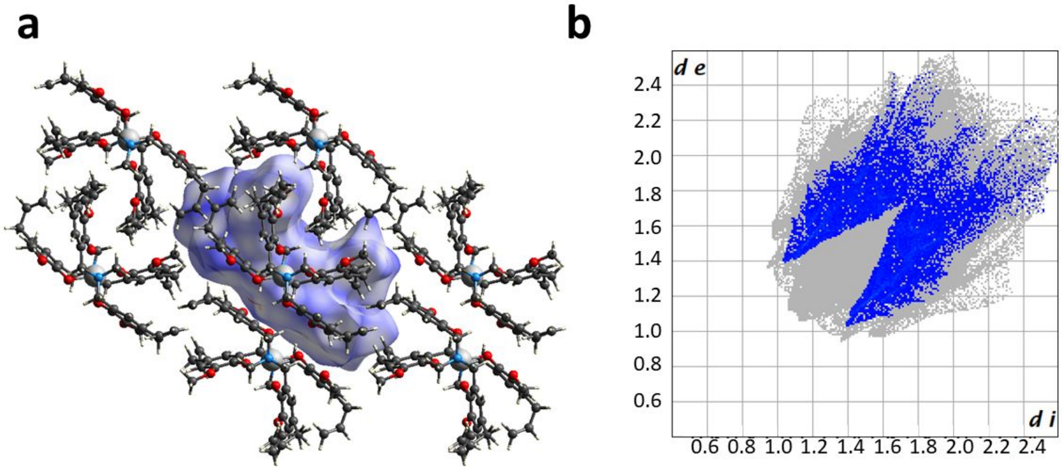

Figure S56. Hirshfeld surfaces of (**3c**). a) Surface Plot. b) Fingerprint highlights of closest contact between the Internal surface plot (d_i) (Hydrogen) and external fragments (d_e) (Oxygen).

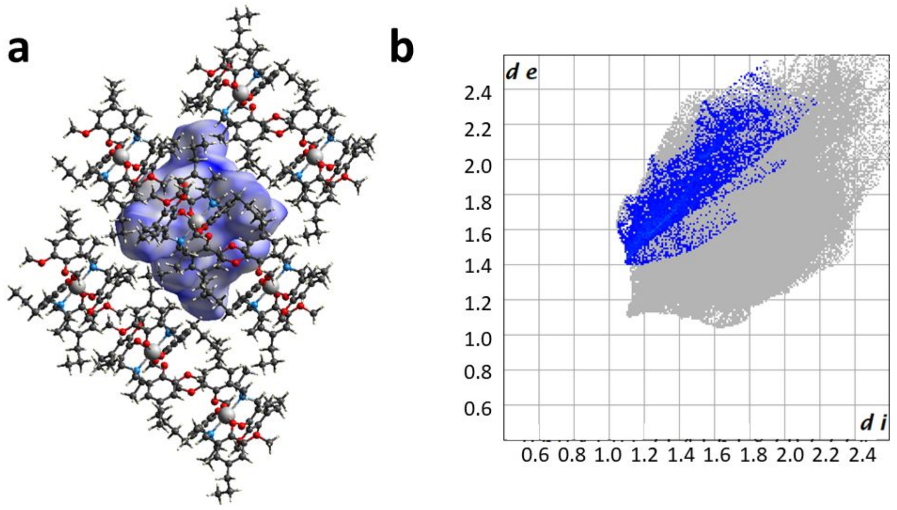

Figure S57. Hirshfeld surfaces of (**3i**). a) Surface Plot. b) Fingerprint highlights of closest contact between the Internal surface plot (d_i) (Hydrogen) and external fragments (d_e) (Oxygen).

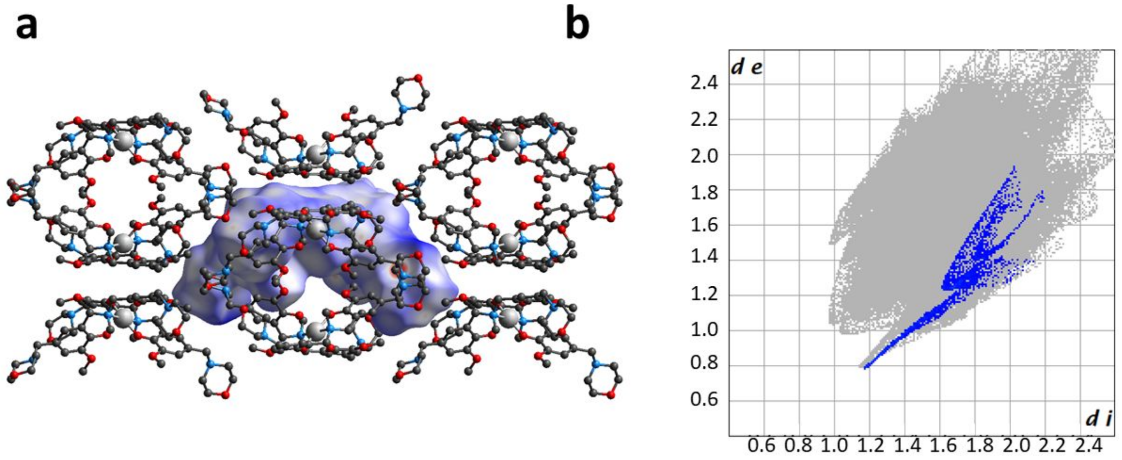

Figure S58. Hirshfeld surfaces of (**3j**). a) Surface Plot. b) Fingerprint highlights of closest contact between the Internal surface plot (d_i) (Nitrogen) and external fragments (d_e) (Hydrogen).

Table S1. Selected comparison of bond length and angle data for titanium complexes **3a-c**, **i** and **j**

Complex	**3a** (ref. 1)[a]	**3b**	**3c**[a]	**3i**	**3j**
R-N	Me	Me	Me	Et	Me
R-(*para*)	Me	Et	$CH_2CH{=}CH_2$	*n*-Pr	CH_2(morpholino)
**Bond Lengths [Å]**					
Ti(1)–O(1)	1.873	1.881	1.903	1.882	1.879
Ti(1)–O(2)	1.900	1.890	1.869	1.872	1.878
Ti(1)–O(3)	1.909	1.883	1.875	1.893	1.893
Ti(1)–O(4)	1.859	1.858	1.904	1.871	1.900
Ti(1)–O_{ave}	1.885	1.878	1.888	1.879	1.888
Ti(1)–N(1)	2.244	2.264	2.267	2.266	2.252
Ti(1)–N(2)	2.267	2.254	2.263	2.269	2.244
Ti(1)–N_{ave}	2.255	2.259	2.265	2.268	2.248
**Bond Angles (°)**					
O(1)–Ti(1)–O(3)	93.1	87.8	91.4	92.0	90.2
O(2)–Ti(1)–O(4)	89.4	93.1	91.0	88.3	91.9
N(1)–Ti(1)–N(2)	174.7	173.2	173.5	179.1	178.0

[a] Average of two independent molecules in the unit cell.

■ 3. Biology methods

■ 3.1 Cell culture

All carcinoma cell lines were obtained from (ATCC) the American Type Tissue Cell Culture Collection and cultured in RPMI 1640 nutrient medium supplemented with 10% (v/v) (FBS) foetal bovine serum, and 1% (v/v) glutamine. All cells were passaged twice weekly to preserve logarithmic growth and were incubated at 37 °C in an atmospheric environment constituting 5% CO_2. The cells utilized in experiments were ≤ 30 passages to decrease genotypic/phenotypic drift. Milligram quantities of cisplatin and the other compounds were weighed using an Ultra Micro Balance (accurate to 0.01 mg). Cisplatin and other compound stock solutions (10 mM) were prepared freshly in DMSO (HPLC grade) directly before use. Dilution (in nutrient medium) procedures were conducted adopting identical procedures each taking <1 h followed by immediate use. Experimental cancer cell lines used in this study were colorectal (HCT-116, HT-29), breast (MCF-7, MDA-MB-468) and pancreatic (Panc-1). Internal repeats were with n = 4. All experiments were replicated ≥3 times.

■ 3.2 Anti-proliferation kinetic rate study (cellular Ti-uptake)

Experimental. Seven identical T25 culture flasks were established containing 1.25 × 10^6 MCF-7 cells. Fresh medium (10 mL) containing agent **3c** (10 µM final concentration) was added to all flasks and the kinetics clock started. Flasks 1-7 were used to assay the number of living and dead cells at 0, 2, 4, 6, 8, 10 and 12 h. An identical approach was used to determine the concentration of **3c** remaining in the solution at the same time points by UV-vis spectroscopy using standard solutions. Control studies indicated: (i) replicates gave identical rates within the statistical errors quoted, (ii) agent **3c** is stable to hydrolysis in the growth

medium alone over the time course of the kinetic experiment, (iii) identical approaches were valid when measuring the growth rate of MCF-7 cells in the absence of agent **3c**.

Analysis. Exponential growth of cells (and decay of agent concentration) was fitted using the regression analysis method of Billo.[5] As the rate of cell doubling and agent depletion both increase over the course of the study data from 0-8 h were taken as representative of the initial slower phase, and data for 4-12 h used to model the onset of the later higher rates (Figure S59). The derived rate constants are reported in Table S2. Based on the loss of agent **3c** from the medium and number of cells at 12 h the total titanium cellular burden is 10 femtomol per cell. Primary data and its fit are reported in the data file 'Kinetics-Cell-Growth-in-presence-of-2' (Excel).

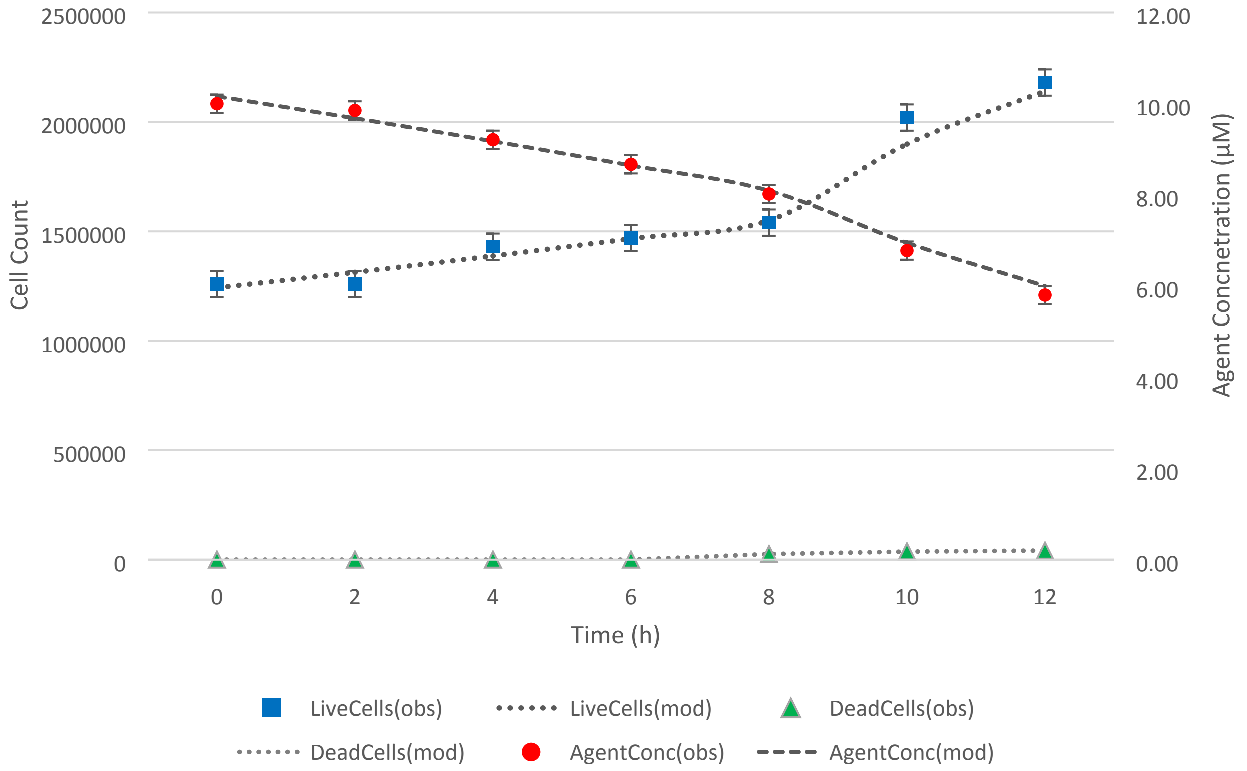

Figure S59. Observed (data points) and modelled behavior (lines) for the disappearance of agent **3c** (●, µM) and growth of living (■) and dead (▲) MCF-7 cell populations over 12 h.

Table S2. Rate constants derived from data of Figure S59. [a]

Process	Initial rate (h^{-1})	Final rate (h^{-1})
Consumption of agent **3c**	0.024(2), R^2 0.98	0.036(3), R^2 0.99
Growth of MCF-7 cells in presence of **3c**	0.028(5), R^2 0.92	0.06(1), R^2 0.91
Death of MCF-7 cells in presence of **3c**	0	0.41(4),[b] R^2 0.99
Growth of MCF-7 cells in absence of **3c**	0.043(4), R^2 0.97[c]	

[a] The figures in parentheses indicate the standard deviation in the proceeding figure on the basis of Billo's SolvStat analysis method, for which the goodness-of-fit is also given.[5]

[b] Data fitted from 6-12 h, no cell death is detected before this time.

[c] Growth of cells (0-12 h) control run identical to approach of Figure S59, but in the absence of **3c**. A constant rate of cell growth, and no cell death, is observed over this period.

3.3 Hydrolysis studies of complexes 3

LCMS Study. Aqueous solutions of complex **3c** were prepared using an identical procedure to that used in biological studies to prepare a 200 µM stock solution. Hydrolysis of **3c** was followed by Liquid chromatography-mass spectrometry (LCMS). Sample analyses were performed using an Agilent 1260 Infinity HPLC with a 6120 quadrupole mass spectrometer with a multimode source. Chromatography conditions: XBridge C18 3.5µm 2.1 x 30 mm column. Mobile phase A: 0.1% Ammonia in water, mobile phase B: acetonitrile. Flow rate 0.8 mL/min in a gradient of 5 – 95 % mobile phase B over 3.5 minutes with UV detection at 210 - 400 nm reported at 254 nm. Column temperature 40 ^{o}C. Exemplary data are presented in Figure S60.

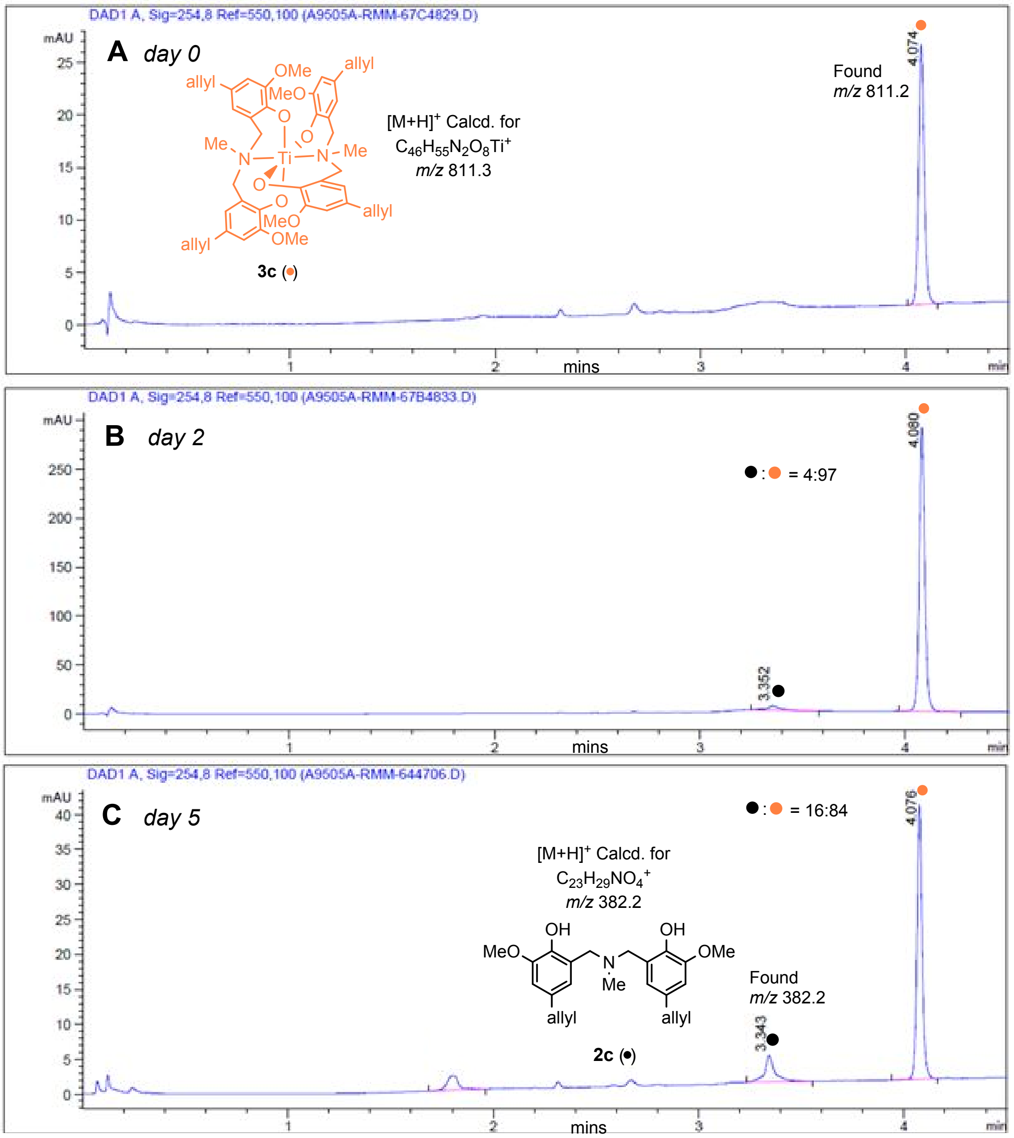

Figure S60. (A) LCMC chromatograph **3c** (200 µM) immediately after dissolution. (B) LCMC chromatograph **3c** (200 µM) two days after dissolution. (C) LCMC chromatograph **3c** (200 µM) two days after dissolution.

NMR Study. DMSO-d$_6$ solutions of **3c** were diluted with D_2O such that a final concentration of 200 µM **3c** in the presence of 9.4 M water (47000 equiv. D_2O) was realized. Equivalent control samples of ligand **2c** in the same solvent mixture were prepared. Complex **3c** was reproducibly observed unchanged over 2-3 days on the basis of its ^{1}H NMR spectrum (Figure S61A). After 1 week, 30±2% of **3c** had been hydrolysed providing free ligand **2c** and a new species (Figure S61C) in exchange with **2c** (Figure SXXA) and **3c** (Figure S61B). After four weeks the new hydrolysis product and free ligand were present in equimolar amounts (Figure S61D). ESI+ MS analysis the NMR sample gave signals attributed to ligand [**2c**+Na]$^+$ *m/z* 406 and those consistent with the hydrolysis product proposed in Figure S61D: [LTi(OH)]$^+$ (L = dianion of **2c**) *m/z* 464).

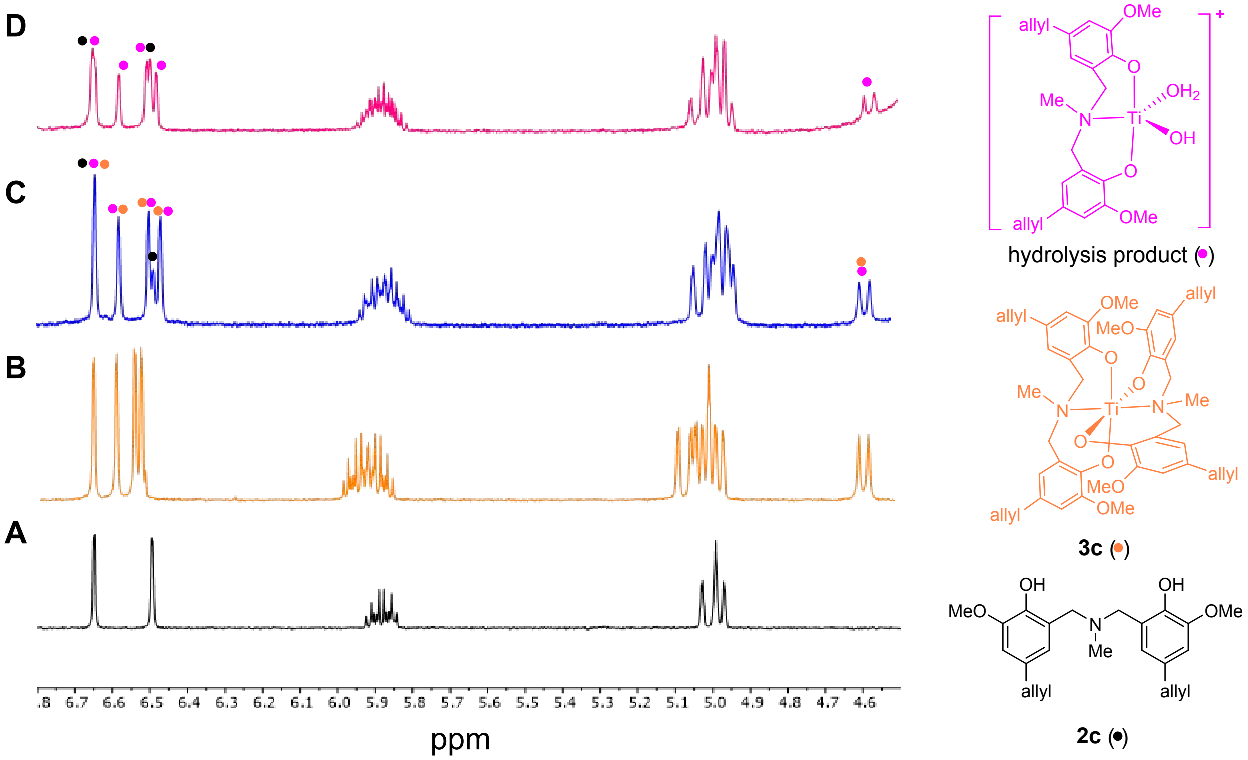

Figure S61. (A) ^{1}H NMR spectra in the aryl/alkene region of **2c** (200 µM in 4:1 DMSO-D$_6$/D_2O; corresponding to 9.4 M water and 4.7 × 10^4 equivalents of D_2O). (B) ^{1}H NMR spectra in the aryl/alkene region of **3c** (200 µM in 4:1 DMSO-D$_6$/D_2O), i.e. identical conditions to (A) and monitored over days 1-3. (C) ^{1}H NMR spectrum of **3c** one week after dissolution. (D) ^{1}H NMR spectrum of **3c** four weeks after dissolution.

■ 3.4 Literature studies allowing estimates of cellular Ti-uptake

The data in Table S3 indicate all literature studies were an estimate of the titanium burden per cancer cell can be made and any assumptions made. Our own data shows a titanium burden increasing smoothly from 0 to 10 femtomol per cell over 12 h. Structures of the literature compounds cited are given in Figure S62.

Table S3. Titanium anti-cancer agents previously identified within cells.

Cell line	TiL_n	Conc. (mM)	Exposure (h)	Anal. method	Ti-burden (femtomol per cell)	Ti imaged in	Ref.
HT-29	**I**	3.2	3	ICPMS	0.2±0.1[a]	-	6
HT-29	**I**	3.2	48	ICPMS	0.75±0.05[a]	-	6
A2780	**I**	1.6	3	ICPMS	0.6±0.15[a]	-	6
A2780	**I**	1.6	48	ICPMS	3.0±0.8[a]	-	6
MDA-468	**II**	30	4	ICPMS	3.3±0.3	-	7
HT-29	**III**	54	30	UV-vis difference	0.9±0.1[b]	-	8
HeLe	**IV**	2	<0.1	Fluorescence	n/a	perinuclear region	9
AGS	**V**	50	4	Fluorescence	n/a	perinuclear region	10
B16F10	**VI**	1 (20)	4	Fluorescence	n/a	perinuclear region (cell wall and localised spots in cytoplasm)	11

[a] A value of 200 pg total protein per cell (average of values in references [12] and [13]) was used to derive these values.

[b] Close packing and a HT-29 diameter of 11 µm (Harvard BioNumbers Database) was used to derive this value.

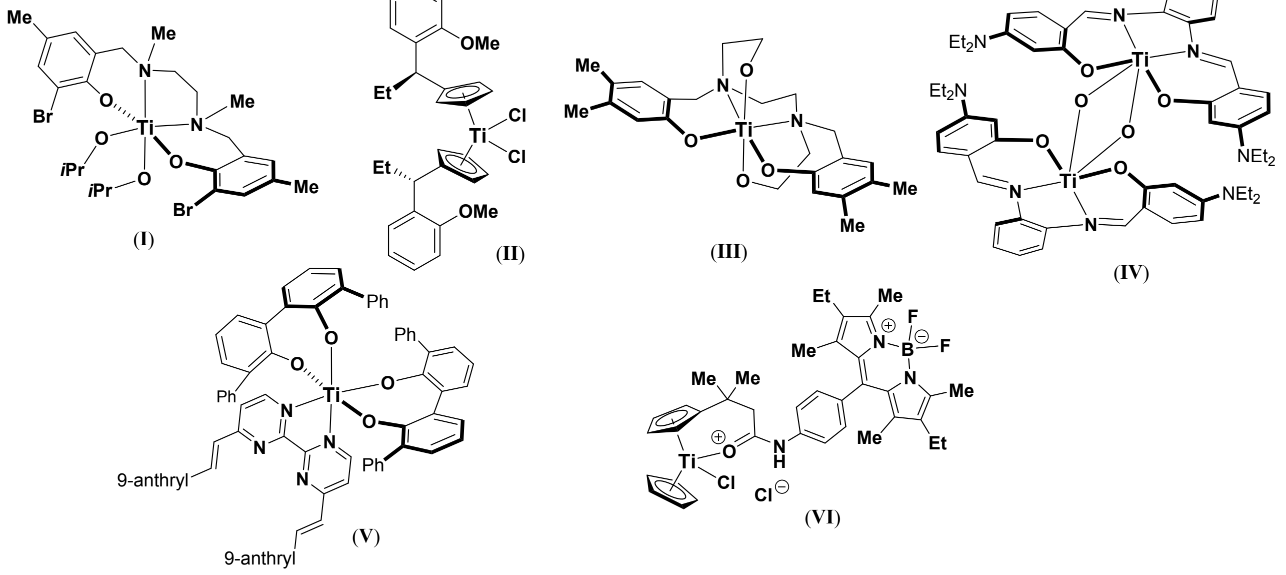

Figure S62. Literature titanium-based cancer agents and probes.

■ 3.5 MTT assay

This technique was utilised to determine the viability of all cell lines employed in this study using the method presented in 1983 by Mosmann.[14] Cells were seeded in 96 well plates containing 180 µL per well at a density of 3000 cells per well and allowed to incubate for 24 h at 37 °C / 5% CO_2. Stock solutions of cisplatin and other screened compounds (10 mM in DMSO) were freshly prepared. Samples of **3b-j** were analytically pure (CHN) and samples were weighed to accuracy of 0.01 mg. From each stock solution serial dilutions of each compound (cisplatin and test agents) were carried out using nutrient medium and 20 µL were introduced into sample wells to achieve 10x final compounds concentrations of 0.05 µM, 0.1 µM, 0.5 µM, 1 µM, 5 µM, 10 µM, 20 µM and 50 µM respectively. MTT assays were conducted at the time of test agent addition (control) and following 72 h exposure of cells to test agents as follows.

To each well was added 50 µL of MTT solution (5 mg/mL PBS). Incubation for 2.5 h at 37 ^{o}C allowed metabolic conversion of MTT to insoluble purple formazan by viable cells, after which time non-formazan supernatants were removed and 150 µL of DMSO was introduced to each well to solubilise formazan crystals. The colour intensity was determined by measuring the wavelength (OD) at 550 nm on a 2104 EnVision® plate reader which is proportional to the number of viable (metabolically active) cells. Data were analysed using the instrument's Wallac EnVision software. Resultant GI_{50} values (concentration required to attain 50% growth inhibition) were calculated using GraphPad Prism software, and are presented in Table S4, Figures S63-S64. Representative growth inhibition by test agents, vs. untreated controls are shown in Movies S1-S4.

Table S4. Activity of complexes **3** (GI_{50} values in µM by MTT assay) against MCF-7, HCT-116, HT-29, Panc-1 and MDA-MB-468 cancer cell lines and non-tumorigenic MRC-5 fibroblast cells. Data were generated from ≥3 independent trials; n = 8 per experimental condition per trial. Selectivity indices (SI) were estimated by dividing GI_{50} in MRC-5 fibroblasts by GI_{50} value cancer cell line.

		GI_{50} values (µM) mean ±SD						Selectivity Indices (SI)				
**(41)**	**CLogP**	**MCF-7**	**HCT-116**	**HT-29**	**PANC-1**	**MDA-MB-468**	**MRC-5**	**MCF-7**	**HCT-116**	**HT-29**	**PANC-1**	**MDA-MB-468**
**3b**	4.0644	1.3±0.2	0.5±0.1	17.5±0.4	1.9±0.2	2.4±0.1	9.3±0.3	7.2	18.6	0.5	4.9	3.9
**3c**	4.1544	2.4±0.2	8.6±0.3	7.5±0.2	4.3±0.1	2.5±0.3	8.2±0.2	3.4	1.0	1.1	1.9	3.3
**3d**	5.1224	17.6±0.3	7.4±0.3	10.2±0.2	16.7±0.3	13.1±0.3	17.7±0.4	1.0	2.4	1.7	1.1	1.4
**3e**	1.9286	26.1±0.1	48.4±0.4	38.2±0.2	20.2±0.6	30.0±0.4	52.1±0.3	2.0	1.1	1.4	2.6	1.7
**3f**	2.7377	2.9±0.3	5.7±0.4	9.1±0.6	1.2±0.4	3.8±0.3	13.5±0.4	4.7	2.4	1.5	11.3	3.6
**3g**	4.5934	7.8±0.4	13.2±0.1	6.8±0.4	14.8±0.2	7.7±0.5	18.5±0.3	2.4	1.4	2.7	1.3	2.4
**3h**	4.6834	7.9±0.4	18.4±0.5	25.0±0.6	11.9±0.5	7.4±0.7	16.3±0.6	2.1	0.9	0.7	1.4	2.2
**3i**	5.6514	6.4±0.2	15.4±0.3	29.1±0.2	11.4±0.1	17.7±0.3	25.3±0.5	4.0	1.6	0.9	2.2	1.4
**3j**	1.5024	27.4±0.6	55.3±0.8	43.1±0.5	32.1±0.4	38.2±0.2	48.5±0.6	1.8	0.9	1.1	1.5	1.3
**3k**	3.5804	3.3±0.5	2.8±0.3	6.2±0.2	7.8±0.6	2.7±0.4	14.6±0.7	4.4	5.2	2.4	1.9	5.4
**Cis**	-2.19	7.6±0.2	8.2±0.4	16.0±0.4	13.1±0.5	4.9±0.3	7.9±0.6	1.0	1.0	0.5	0.6	1.6

Cis = Indicates that Cisplatin was used as a positive control.

A

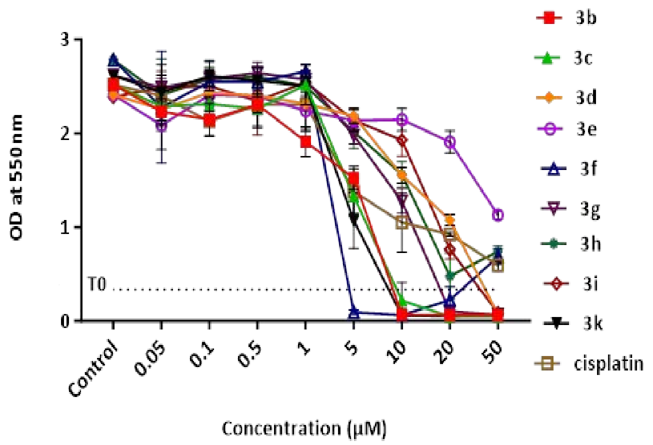
OD at 550nm
Concentration (μM)
Control
0.05
0.1
0.5
1
5
10
20
50
T0
3b
3c
3d
3e
3f
3g
3h
3i
3k
cisplatin

B

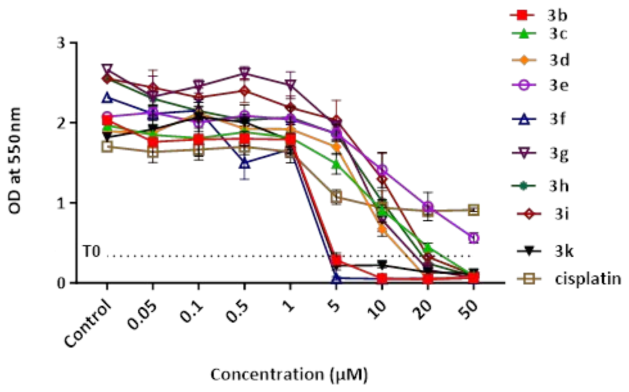
OD at 550nm
Concentration (μM)
Control
0.05
0.1
0.5
1
5
10
20
50
T0
3b
3c
3d
3e
3f
3g
3h
3i
3k
cisplatin

C

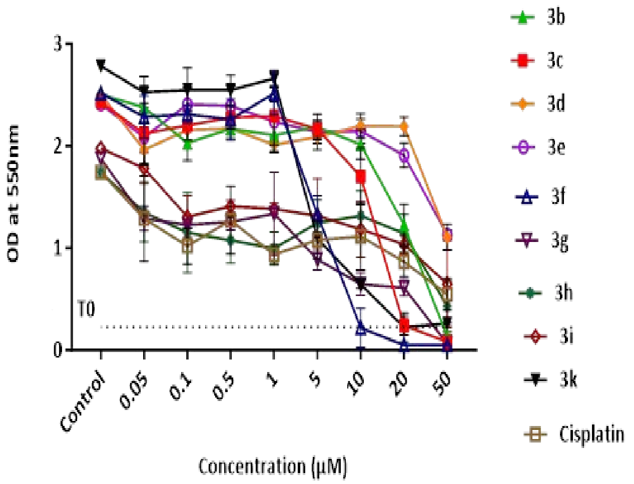
OD at 550nm
Concentration (μM)
Control
0.05
0.1
0.5
1
5
10
20
50
T0
3b
3c
3d
3e
3f
3g
3h
3i
3k
Cisplatin

D

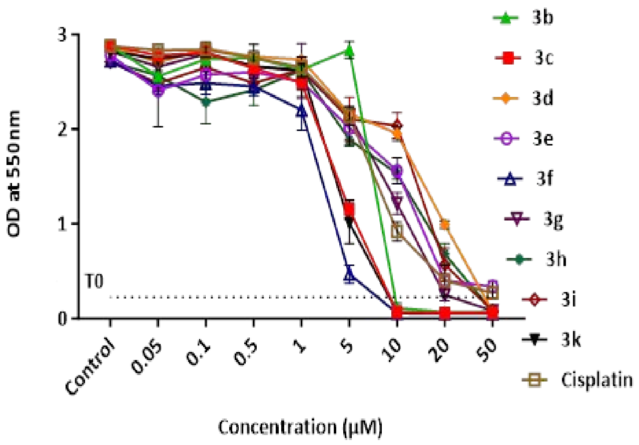
OD at 550nm
Concentration (μM)
Control
0.05
0.1
0.5
1
5
10
20
50
T0
3b
3c
3d
3e
3f
3g
3h
3i
3k
Cisplatin

E

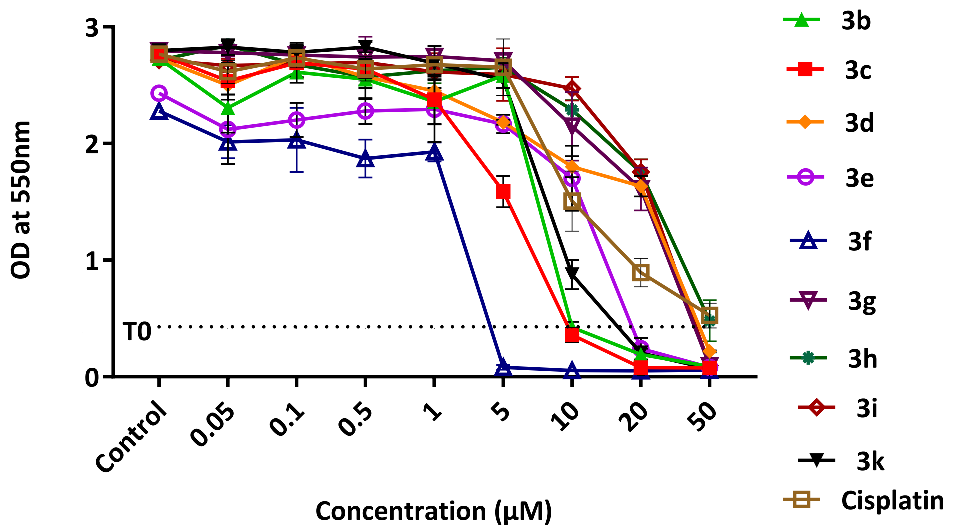

Figure S63. Representative MTT graphs display the dose-dependent growth inhibitory properties of **3b–k** against HCT-116 (**A**), HT-29 (**B**), MCF-7 (**C**), MDA-MB-468 (**D**) and Panc-1 (**E**) cell lines. Cells were seeded in 96-well plates at a density of 3 × 10^3 cells/well. After allowing 24 h to adhere, cells were treated with the specified compound and incubated for 72 h. Data points depict mean ±S.D. n = 8; MTT assays were repeated >3 times.

■ 3.6 Visualization of cellular growth and inhibition

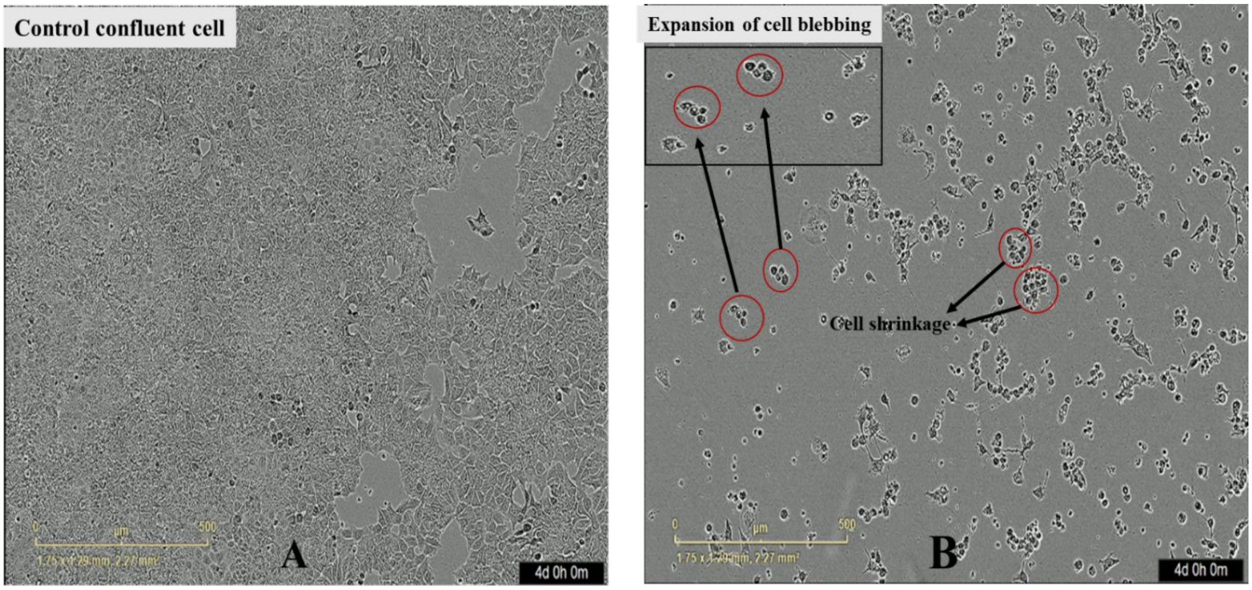

Figure S64. Images of HCT-116 cells: (**A**) control after 72 h from the treatment time; (**B**) following 72 h exposure of cell to 5 μM of **3c**. Images taken from the Incucyte study (see below).

Movies S1-S4. Videos showing comparison between (**A**) HCT-116 treated, (**B**) HCT-116 control, (**C**) MCF-7 treated and (**D**) MCF-7 control. Treated samples were after exposure to **3c** (5 μM) monitored over 72 h. Control movies show normal cell growth in the absence of **3c**. Videos were recorded on a Sartorius Incucyt SX5 device.

■ 3.6 Cell counting assay

The MTT assay is widely applied to measure cell proliferation and viability. However, due to its mechanism which is dependent on chemical (formazan) for detection, false positive results can potentially be obtained for compounds which may have an intrinsic ability to reduce MTT regardless of any cellular mechanism.[15] For that reason, cell counts were also performed to substantiate MTT assay results. Cells were seeded at a density of 2×10^4 in 6 well plates and incubated overnight (16 h) before treatment with compound **3b-k** at 1 and 5 μM. Following 72 h exposure to test agent, cells were harvested and counted using a haemocytometer. Decreasing numbers of viable cells reflected growth inhibition and toxicity caused by test agent and corroborated MTT assay results. Associated primary data are shown in Figure S65.

A

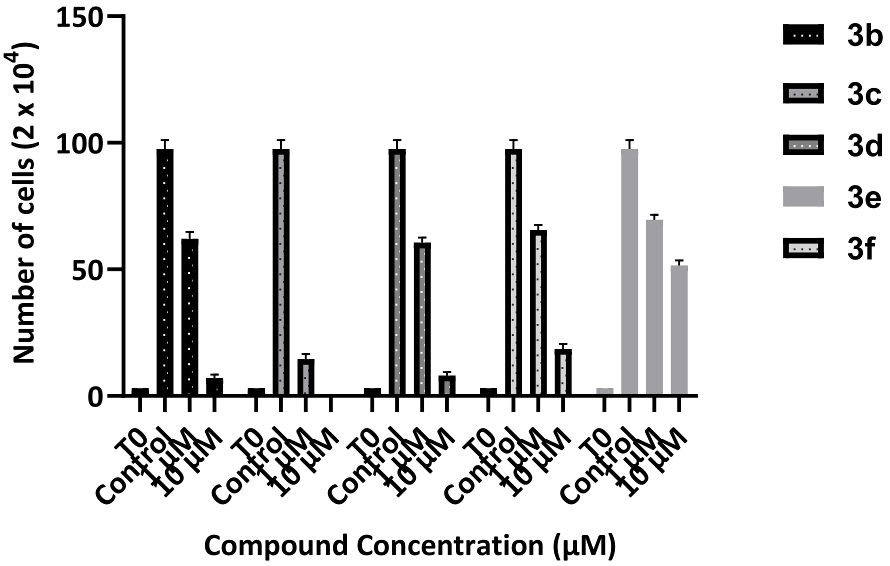

150
100
50
0
Number of cells (2 x 10^4)
T0
Control
1 µM
10 µM
Compound Concentration (µM)
3b
3c
3d
3e
3f

B

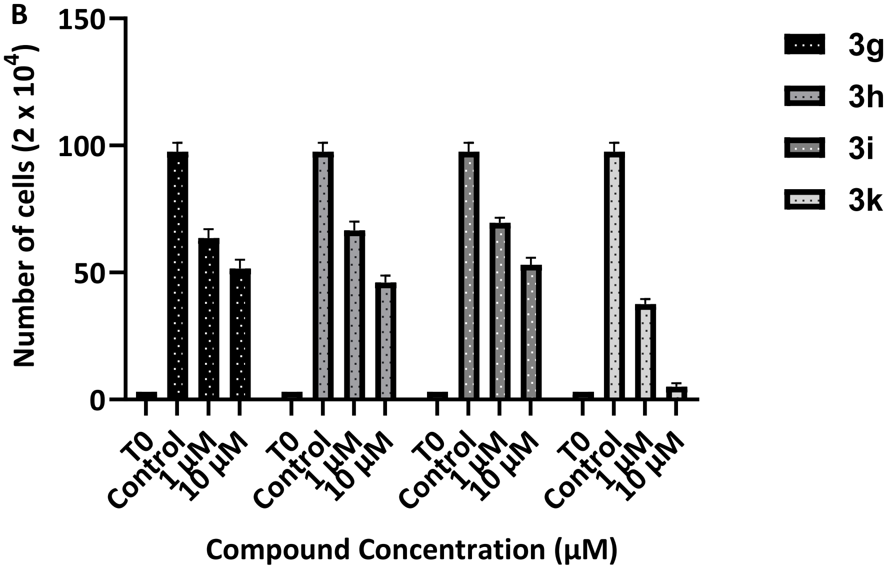

150
100
50
0
Number of cells (2 x 10^4)
T0
Control
1 µM
10 µM
Compound Concentration (µM)
3g
3h
3i
3k

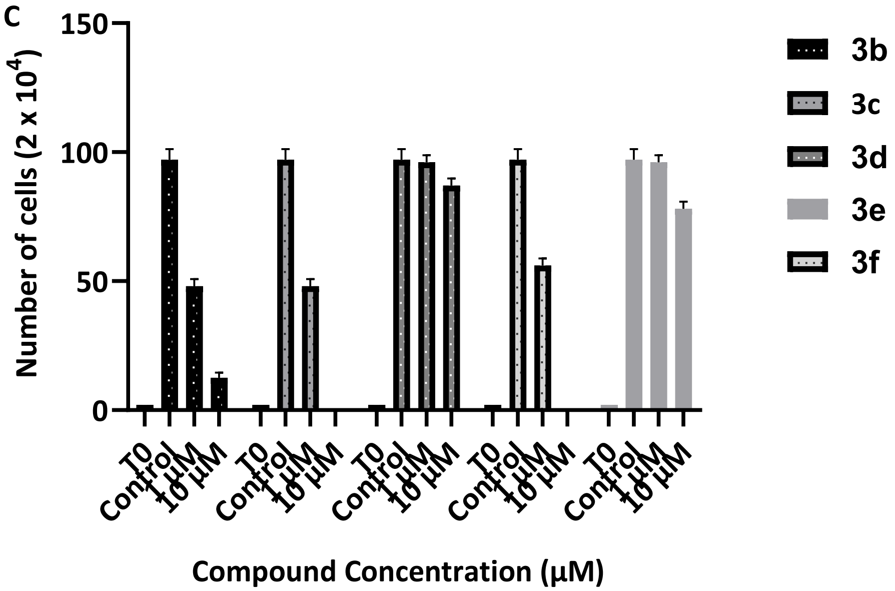

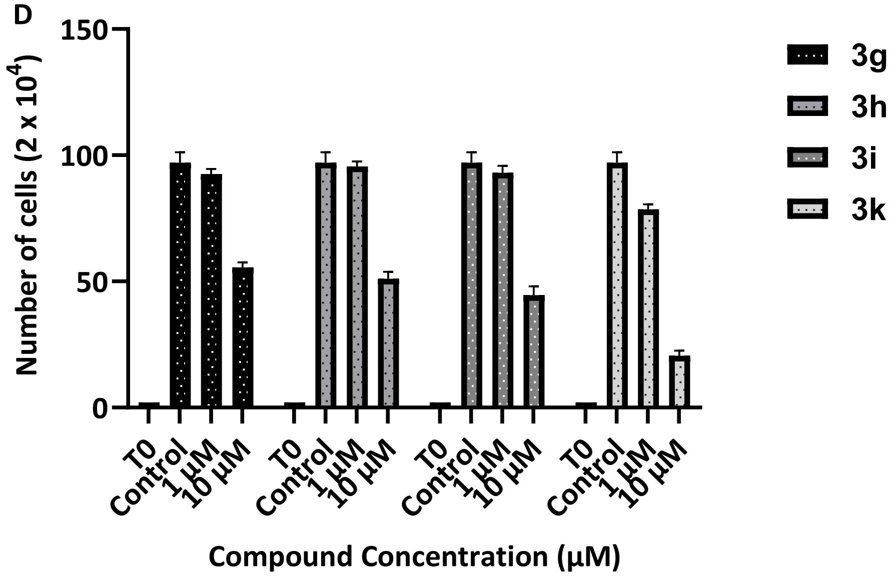

Figure S65. Effects of complex **3b-k** on HCT-116 (**A** and **B**) and MCF-7 (**C** and **D**) cell numbers. Cells (2 × 10^4 cells/well) were seeded in 6-well plates and incubated overnight before treatment at 1 and 10 µM (72 h). Cells were harvested and counted by hemocytometer.

■ 3.8 Clonogenic assay

Cells were counted using a haemocytometer, 400 cells for HCT-116 and 500 cells for MCF-7 were seeded per well in 6-well plates with 2 mL medium. Cells were allowed to attach for 24 h. The cells were then treated with test agent at 5 and 10 μM. Cells in the control wells were treated with the vehicle (medium) alone. Following 24 h exposure titanium compounds, the medium was aspirated along with the compound. Wells were washed with 1 mL of cold PBS and 2 mL of fresh medium was added to each well. Plates were placed in the incubator at 37 °C and inspected daily until cells in control wells formed colonies of ≥ 50 cells. The colonies were washed with PBS before fixation with 100% methanol (0.5 mL) for 15 min and then stained with 0.7 mL of 0.05% methylene blue (1:1 water/methanol) for an additional 10 minutes. The colonies were counted and survival fractions recorded graphically using GraphPad prism, associated primary data are shown in Figures S66-S67.

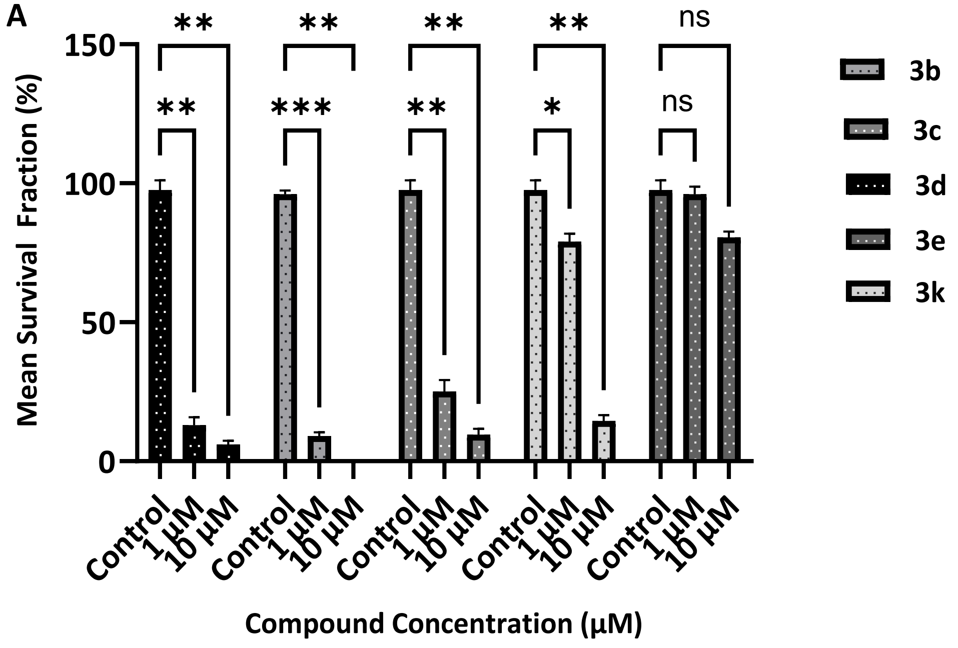

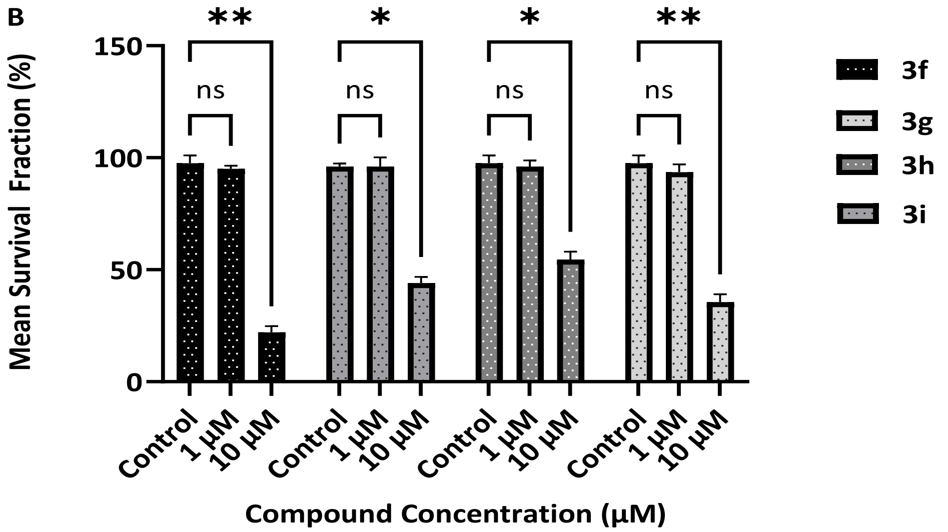
B
Mean Survival Fraction (%)
150
100
50
0
**
*
*
**
ns
ns
ns
ns
Control
1 μM
10 μM
Compound Concentration (μM)
3f
3g
3h
3i

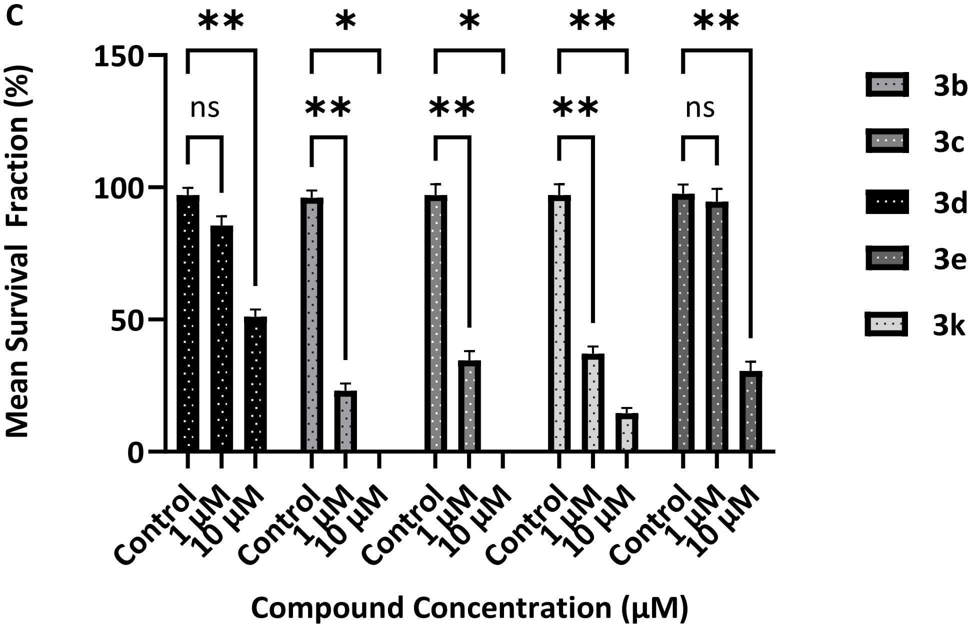
C
Mean Survival Fraction (%)
150
100
50
0
**
*
*
**
**
ns
**
**
**
ns
Control
1 μM
10 μM
Compound Concentration (μM)
3b
3c
3d
3e
3k

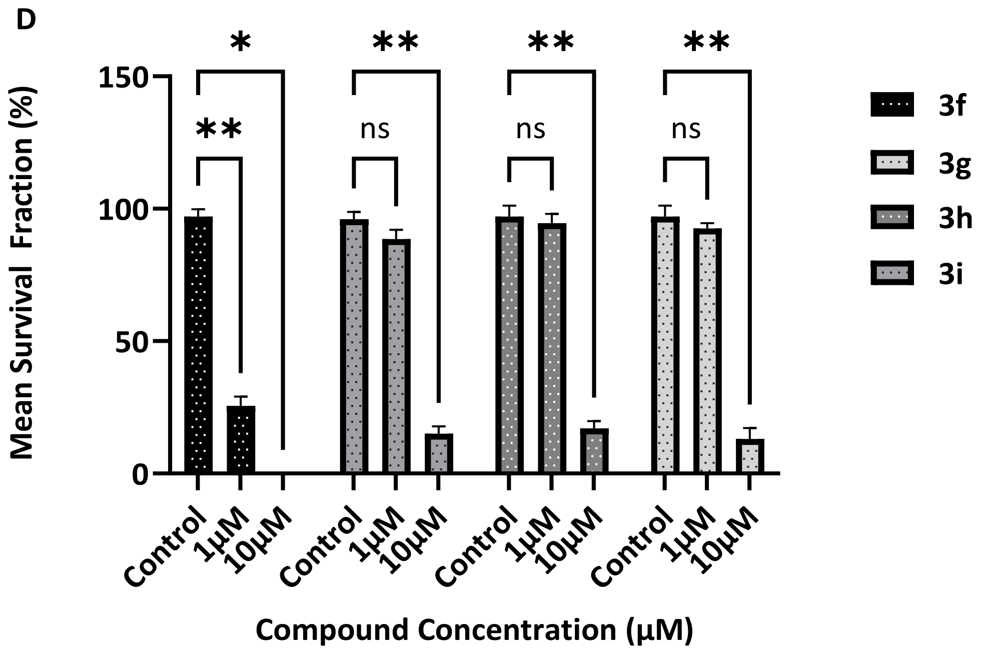

Figure S66. Effect of **3b**-**k** on HCT-116 (**A** and **B**), MCF-7 (**C** and **D**) colony formation. Graphs **A**-**D** show the mean survival fraction of % control represented of mean ±SD of 3 independent trials (n = 2 per trial). Complexes **3d** and **3c** exhibited significant reduction in colony formation with ($p < 0.0001$).

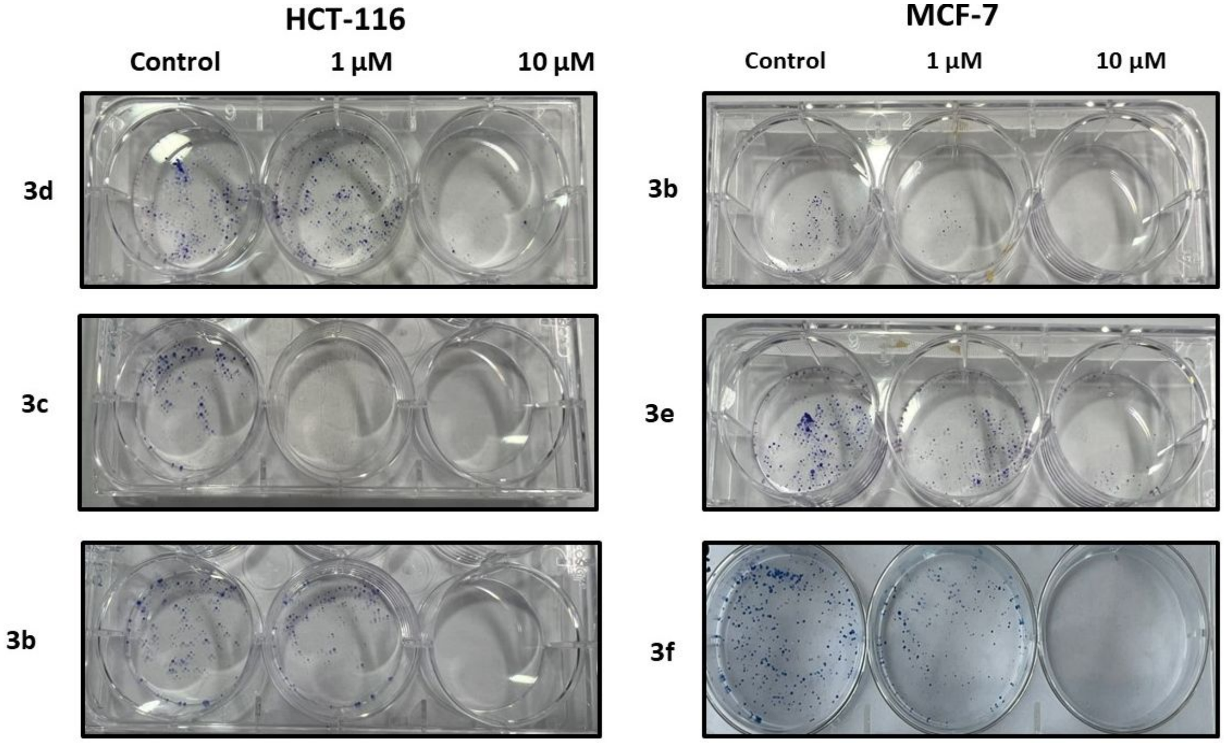

Figure S67. Representative effects of complexes **3b**, **3e** and **3f** for MCF-7 and compounds **3b-d** on HCT-116 clonogenic survival at (1 and 10 µM) respectively.

■ 3.9 Cell cycle assay

The method of Nicoletti et al. (1991)[16] was adapted to analyse cell cycle distributions. Cells were seeded in 10 cm diameter petri dishes in 10 mL of complete medium at a density of 7 × 10^5 (control and 24 h treatment) and 4 × 10^5 (48 and 72 h treatments) cells per dish. The cells were incubated at 37 °C for 24 h to allow time for their adherence to the base of the petri dishes. The cells were then treated with desired concentrations of the test compounds (5 and 10 µM). After the required period of exposure, both the medium and any floating cells were transferred to a labelled 15 mL falcon tube. Afterward, remaining adherent cells were harvested by addition of 0.5 mL 1× trypsin EDTA, and once detached these were pooled together with the previous medium and the total falcon tube contents centrifuged in a Beckman Coulter Allegro 33 centrifuge at 1200 rpm for 5 min at 4 °C. Supernatant was discarded and the pellets were broken down by gently flicking the tube. Thereafter, 1 mL of cooled (4 ^{o}C) PBS was added, the tubes vortexed and the centrifugation repeated. The supernatant was again discarded, the cell pellet disrupted and the cells were re-suspended in 0.7 mL cold (4 ^{o}C) hypotonic fluorochrome solution (50 µg/ml propidium iodide (PI) in 0.1% sodium citrate and 0.1% mg/mL RNA plus 0.1% Triton X-100). The cell suspension was transferred to a labelled fluorescence activated cell sorter (FACS) tube and stored overnight at 4 °C in the dark. Cells were vortexed to attain a single cell suspension for analysis; 20000 events were recorded for each sample using FC500 Beckman Coulter flow cytometer. The results obtained were analysed using WEASEL software, and are presented in Figures S68-S69.

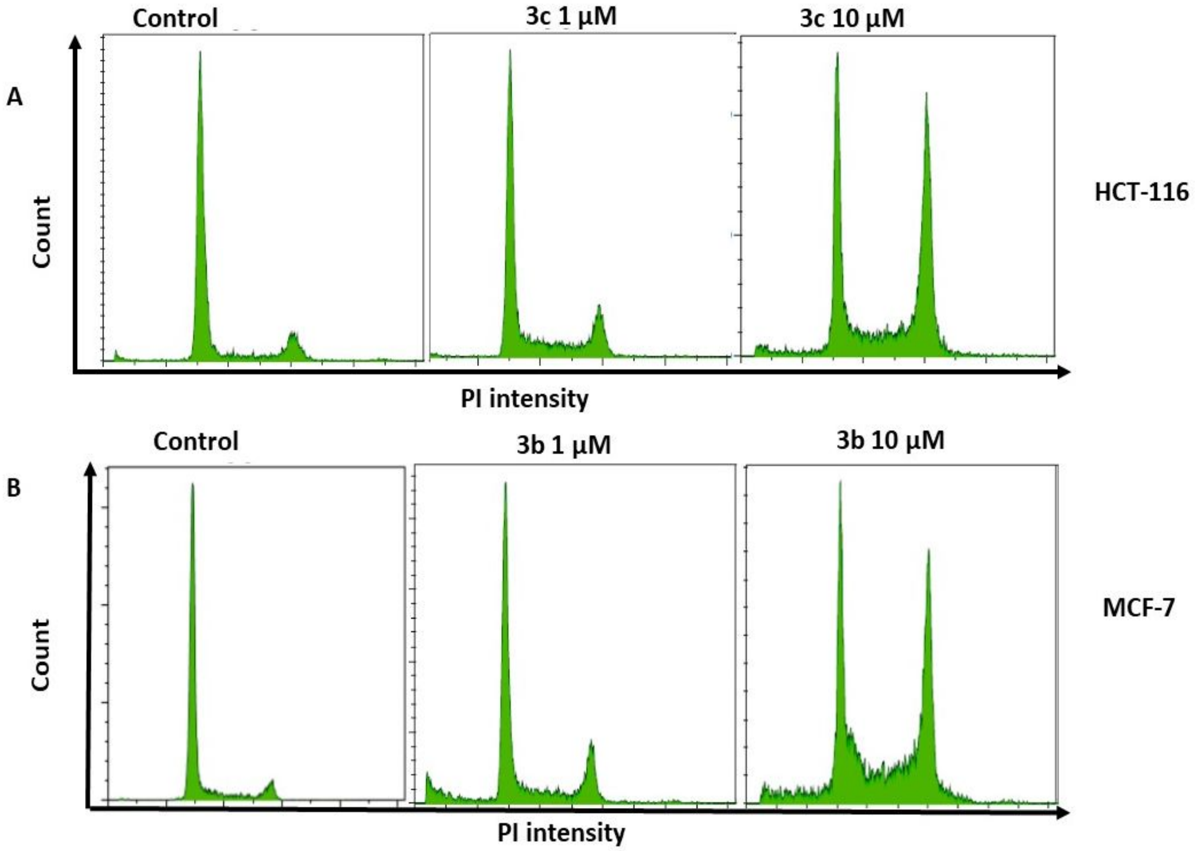

Figure S68. Representative cell cycle histograms of three repeat of (**A**) HCT-116 cells treated with **3c** and (**B**) MCF-7 cells treated with **3b** at 1 and 10 µM concentrations for 72 h. Cells were seeded at a density of 4 x 10^5 (n = 3, for ≥3 trials). The histogram shows the accumulation of events at G2/M phase of the cell cycle caused by **3b** and **3c**; 20000 events were recorded for each sample.

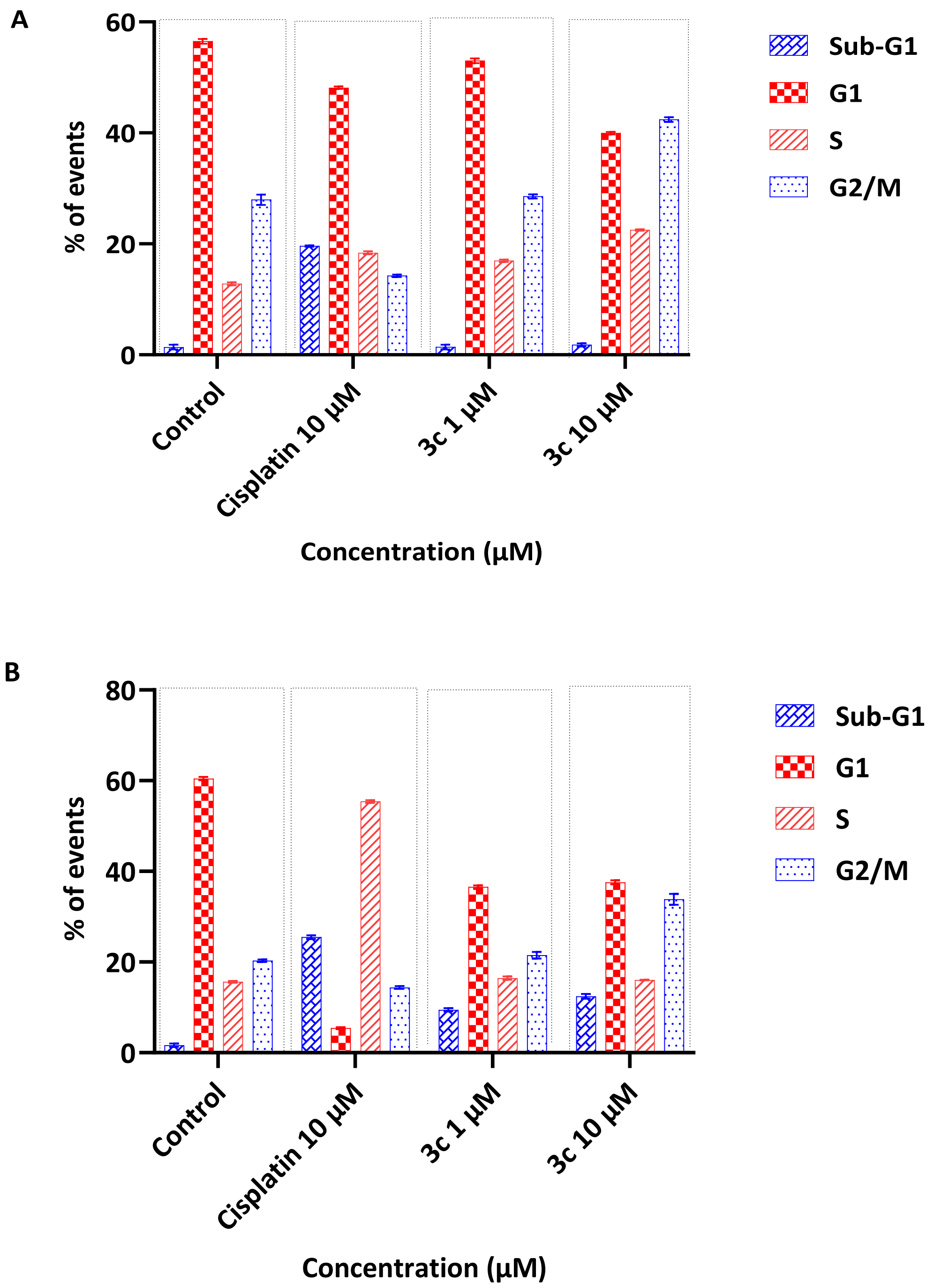

Figure S69. Effect of **3c** on HCT-116 (**A**) and MCF-7 (**B**) cell cycle distribution following 72 h exposure to test agents **3b** the results from these studies are presented in the main paper (Figure 3B). Complex**3c** evoked significant arrest in the G2/M phase, experiments were repeated ≥3 times; 20,000 events were recorded for each sample.

■ 3.10 Annexin V assay

See text in main paper.

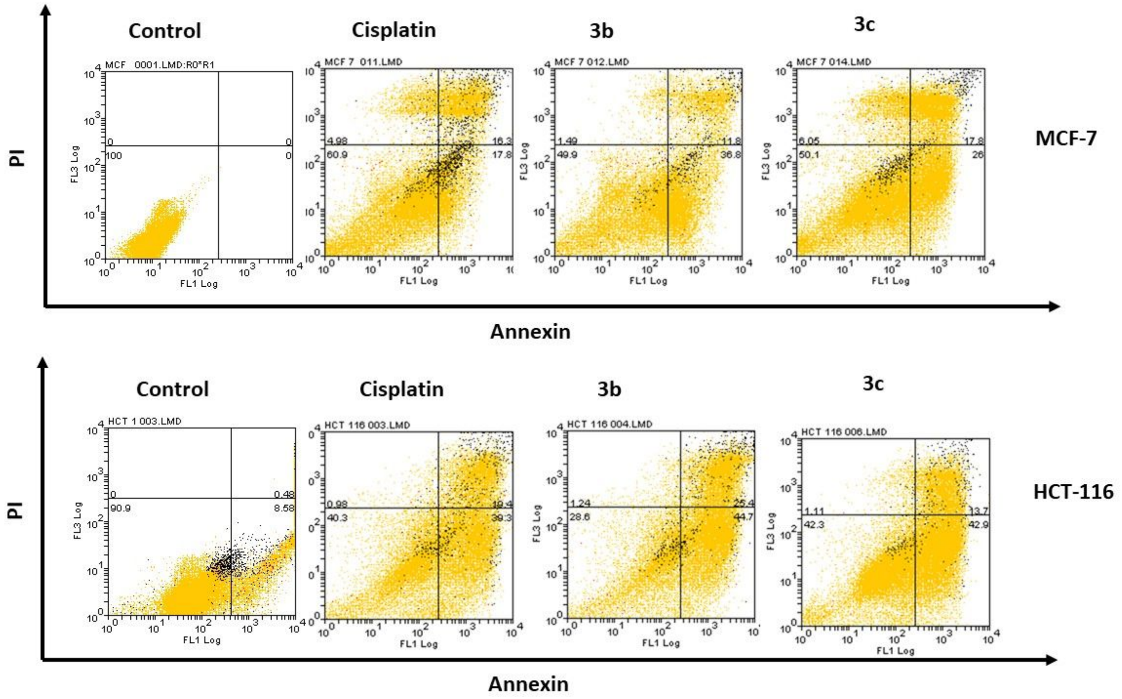

Figure S70. Representative apoptosis quadrant plots illustrating apoptotic effects of **3b**, **3c** and cisplatin on MCF-7 and HCT-116. Both cell lines were treated with 10 µM samples of each agent for 72 h. Cells were initially seeded at a density of 4 × 10^5 (n = 2, for 3 trials). The quadrant plots were attained directly from the flow cytometry data; 20000 events were recorded for each sample.

■ 3.11 Determination of γ-H2AX foci perturbation

See main paper text.

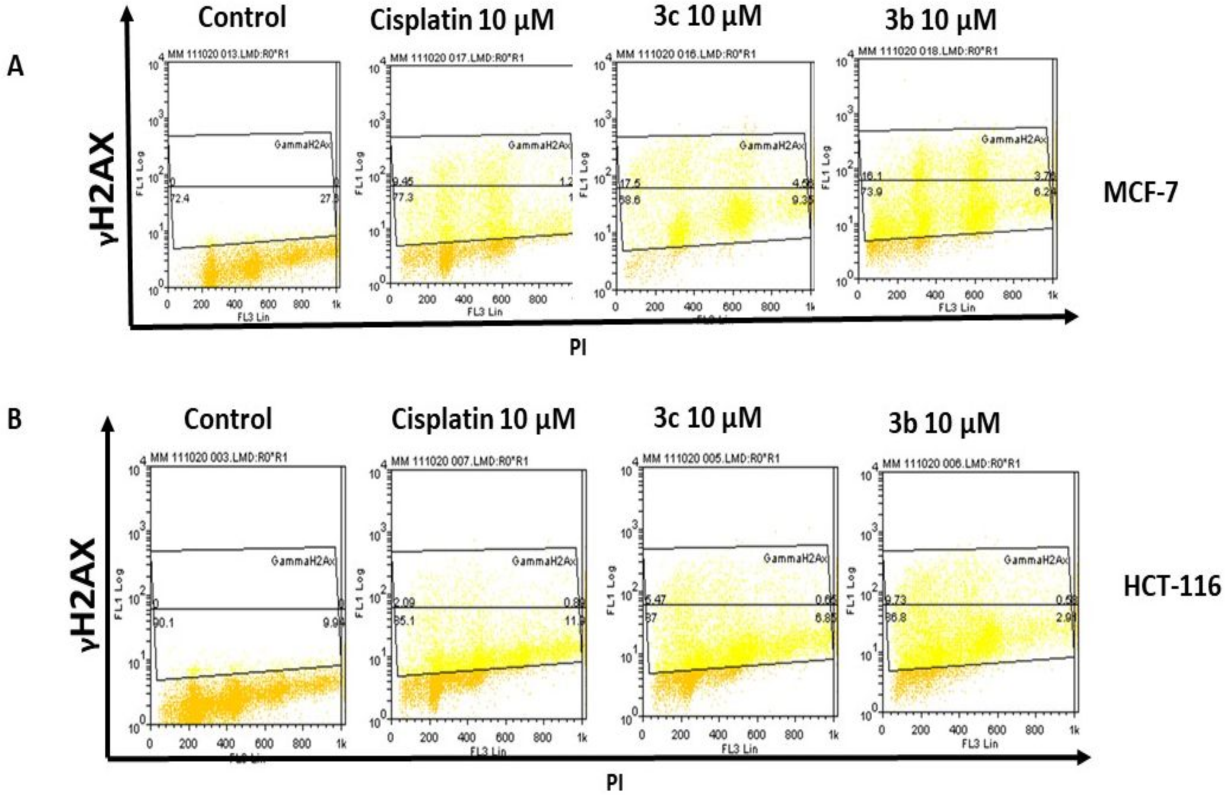

Figure S71. Determination of DNA double strand breaks by flow cytometric analyses of γH2AX tested cells following 72 h treatment of cells with **3b**, **3c** or cisplatin (all at 10 μM): MCF-7 (**A**) and HCT-116 (**B**). Data represents independent trials (n = 2; 20,000 events analysed per sample).

■ 3.12 Caspase-3/7 activity assay

Caspase-Glo(R) 3/7 reagent, # G8091 (Promega) (https://www.promega.co.uk) was used to determine caspase activity. Cells (3 × 10^3 per well) were seeded in 96 well opaque white (or black) cell culture plates and incubated overnight at 37 °C. Compounds **3b** and **3c** (5 and 10 μM, 72 h exposure) were trialed. The caspase-Glo(R) 3/7 reagent was added to each well with gentle mixing (300-500 rpm) for at least 30 seconds. The treated plates were incubated for 30 min at room temperature. Well fluorescence was measured using an EnVision multilabel plate reader (PerkinElmer) at wavelengths between 499 nm and521 nm. The results from these studies are presented in the main paper (Figure 4B).

■ 3.13 Confocal microscopy

HCT-116 cells or MCF-7 cells (3 × 10^5) were seeded in coverslips in glass petri dishes in 2 mL of RPMI supplemented with 10% FBS. Cells were incubated at 37 °C for 24 h, to allow attachment, prior to **3b** and **3c** exposure (5 μM, 24 h). Cells were fixed in formaldehyde (3.7% in PBS; 10-15 min) then permeabilized by PBT (PBS + 0.1% Triton X-100; 2-3 min). Blocking agent (PBT + 1% BSA; 1 h) was used to prevent binding non-specific protein binding. Cells were incubated with 1:200 diluted 1° Ab (monoclonal anti α-tubulin Ab, VWR International Ltd.; 2 h), washed with PBT before incubation in the dark with 1:400 diluted 2° Ab for

1 h. Cells were incubated with DNA binding dye (DRAQ5) for 5 min in the dark. A Zeiss LSM510Meta confocal microscope was used to capture the images. Images were analyzed using Zen software, representative examples are presented in Figure S72.

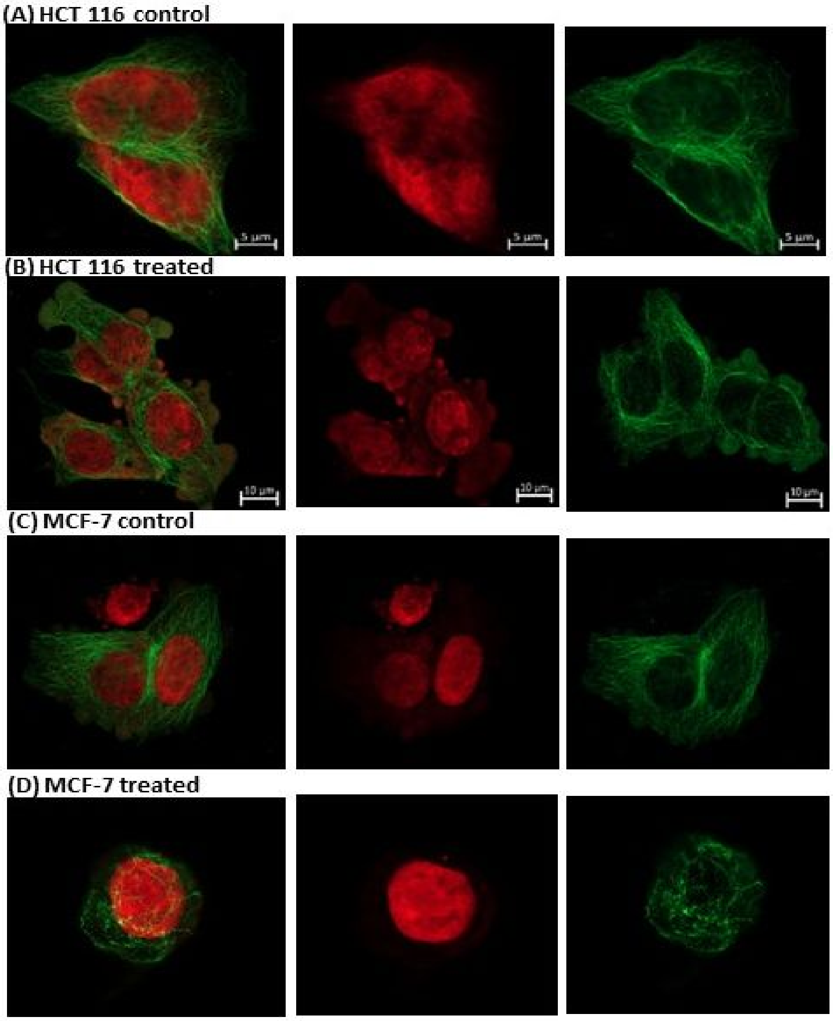

Figure S72. Confocal microscopy studies. (**A**) HCT-116 control cellular images. (**B**) HCT-116 cells treated with **3c** (5 µM, 24 h) membrane blebbing evdent indicative of apoptosis. (**C**) MCF-7 control cellular images. (**D**) MCF-7 cells treated with **3b** (5 µM, 24 h) cell shrinkage, chromatin condensation indicative of apoptosis. Nuclear regions are shown in red (DRAQ5), cell membrane regions in green (secondary antibody). The left most figures show a composite image.

■ 3.14 Relative protein phosphorylation

Protein phosphorylation levels of 17 relevant kinases were detected simultaneously using a human/mouse phospho-mitogen-activated protein kinase (MAPK) antibody array (126AAH-MAPK-1-8) from Ray-bio (https://www.raybiotech.com) according to the manufacturer's instructions. Briefly, HCT-116 and MCF-7 cells were seeded in Corning tissue-culture treated culture dishes (100 mm × 20 mm, 1 × 10^6 cells per culture dish). The cells were allowed to attach overnight and treated with 1 and 5 µM Ti complex **3b** for 24 h. The cells were rinsed with PBS followed by solubilization in lysis buffer whilst gently rocking at 4 °C for 30 min. Cell lysates were then microcentrifuged at 14000× G for 5 min and then the

supernatant was transferred into a clean Eppendorf tube. Quantitation of sample protein concentration was carried out using QuickStart Bradford Assay (Bio-Rad, Hemel Hempstead). Aliquots of the lysates were then stored frozen at –80 °C. The antibody array membrane was blocked using 2 mL of blocking buffer and incubated for 30 minutes at RT. Diluted lysate sample (1 mL) was added into each well and incubated overnight at 4 °C. The lysate was removed and washed with buffer (I) 3 × 2 mL and incubated for 5 mins at RT. The kit's wash buffer (I) was removed, and wash buffer (II) added (2 mL). After washing 2× the antibody array membrane and incubating for 5 mins at RT, the detection antibody cocktail (1 mL) was added into each well and the antibody array membrane incubated overnight at 4 °C. The detection antibody cocktail was then removed from each well and each well was washed twice (5 mins each) using wash buffers (I) and (II) at RT. HRP-Anti-Rabbit IgG 1:1000 dilution (2 mL) was added into each well and incubated overnight at 4 °C. HRP-Anti-Rabbit was removed, and wells were washed twice (5 mins each) using wash buffers (I) and (II) at RT. The antibody array membranes were transferred onto the chromatographic sheet removing any excess wash buffer by blotting the membrane edges with absorbent paper. Detection buffer C (250 µL) and detection buffer D (250 µL) were added to each membrane (one for control and one for treated cells) and membranes incubated for 2 mins at RT. Phosphorylated proteins were revealed using streptavidin-HRP/Chemi Reagent Mix and autoradiography films (Amersham Hyperfilm ECL, GE Healthcare Life Sciences, Buckinghamshire, UK). Results from this study are presented in Figure S73.

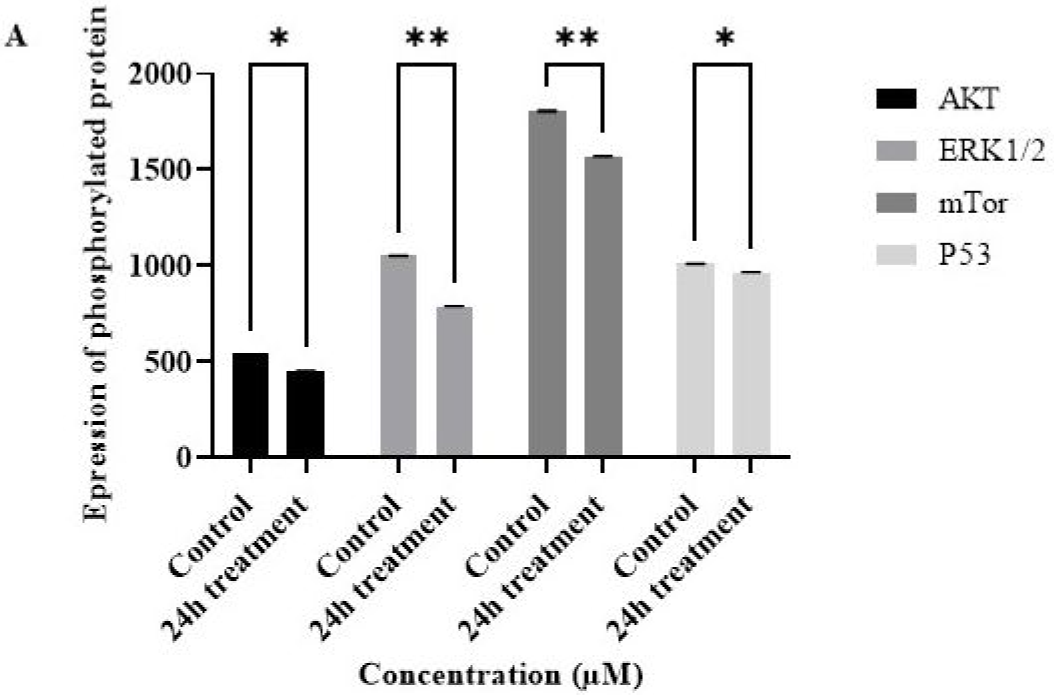
A
Epression of phosphorylated protein
2000
1500
1000
500
0
*
**
**
*
AKT
ERK1/2
mTor
P53
Control
24h treatment
Control
24h treatment
Control
24h treatment
Control
24h treatment
Concentration (μM)

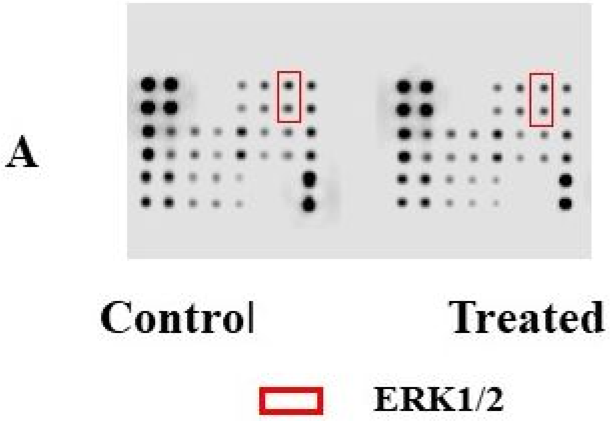
A
Control
Treated
ERK1/2

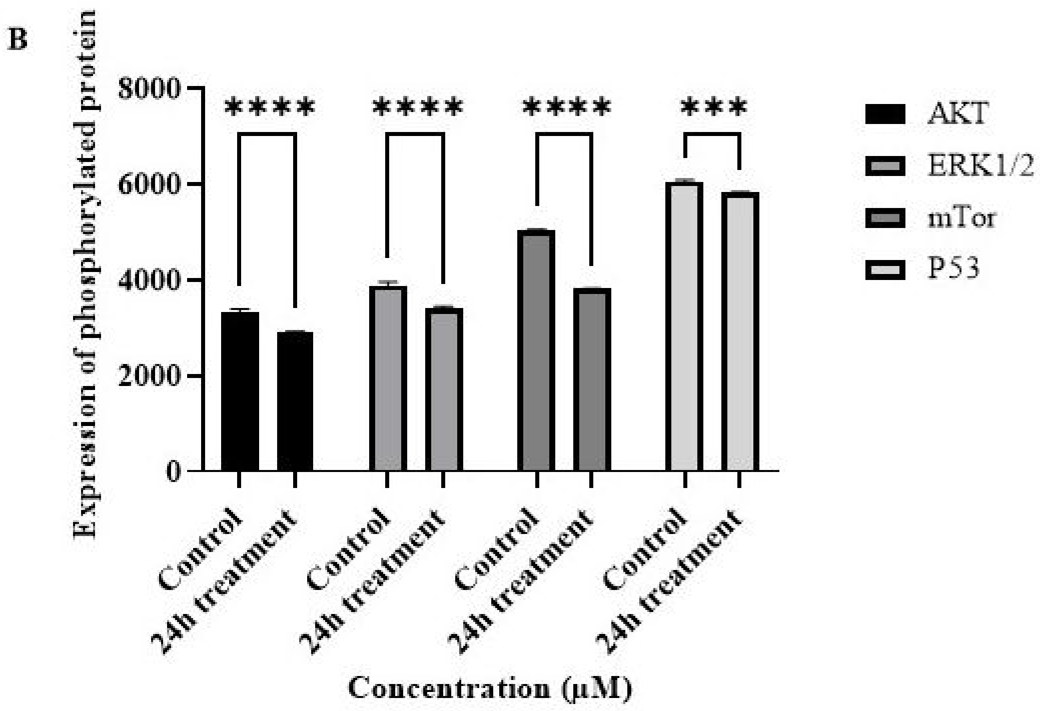

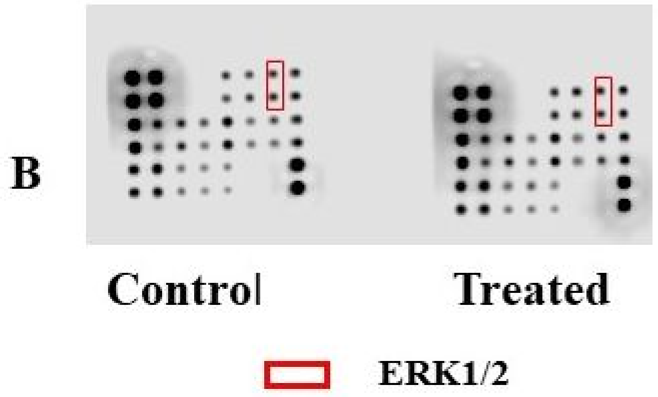

Figure S73. Relative phosphorylation of proteins of the MAPK pathway induced by 24 h treatment with 5 μM **3b** complex using MAPK protein screening kit HCT-116 (**A**) and MCF-7 (**B**). A total of 50 μg protein was loaded, n = 3.

■ **3.15 Western blot**

MCF-7 and HCT 116 cells were seeded in dishes (100 × 20 mm) at a density of 2 × 10^6 per dish. These were allowed 24 h to attach, and then exposed to test agent (**3b** or **3c**, 5 and 10 µM) for 24 h. Following the required exposure, the cells were lysed in NP-40 lysis buffer, supplemented with Phos- STOP Phosphatase Inhibitor Cocktail (Roche Applied Science) and COMPLETE-mini–Protease Inhibitor Cocktail (Roche Applied Science). Cell debris was removed by centrifugation at 1400 rpm for 5 min. Protein concentrations were determined using Quick-Start Bradford Assay (Bio-Rad, Hemel Hempstead, UK). Cell lysate proteins (50 mg) were separated by SDS-polyacrylamide gel electrophoresis and then transferred to a nitrocellulose membrane. Membranes were probed with the following antibodies: Whole PARP, cleaved PARP, Mcl-1, Bcl-2, ERK1/2(Thr202/Tyr204), total-ERK1/2 and GAPDH (all purchased from Cell Signaling Technologies). Results from these studies are reported in Figures S74-S75.

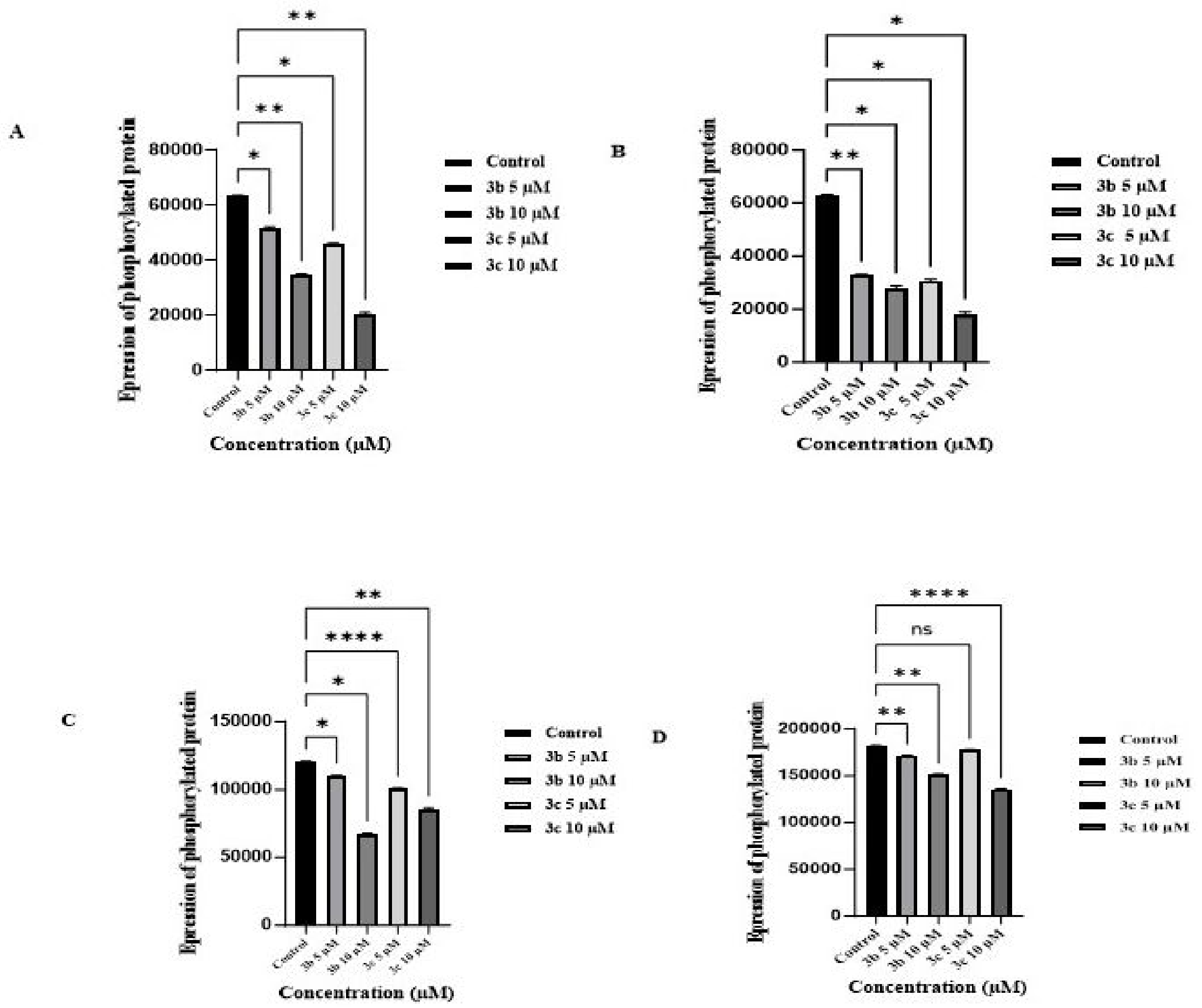

E

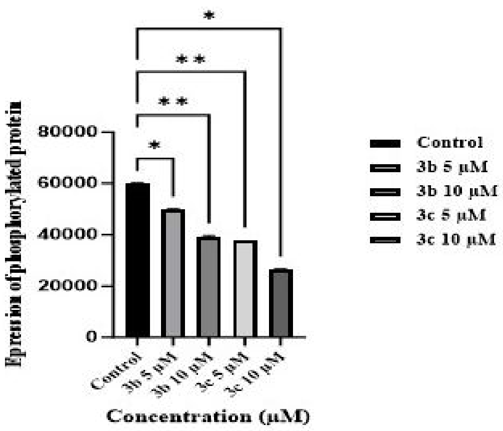

*
* *
* *
*
80000
60000
40000
20000
0
Epression of phosphorylated protein
Control
3b 5 μM
3b 10 μM
3c 5 μM
3c 10 μM
Concentration (μM)

F

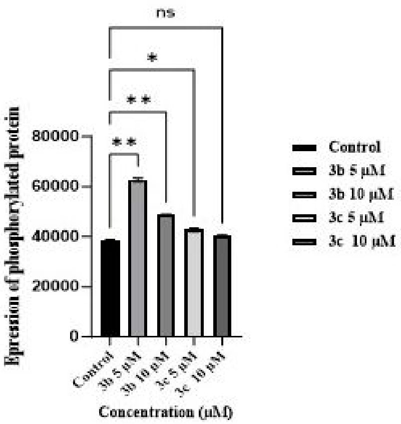

ns
*
* *
* *
80000
60000
40000
20000
0
Epression of phosphorylated protein
Control
3b 5 μM
3b 10 μM
3c 5 μM
3c 10 μM
Concentration (μM)

G

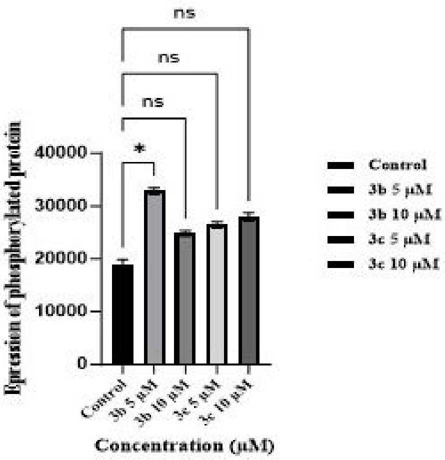

ns
ns
ns
*
40000
30000
20000
10000
0
Epression of phosphorylated protein
Control
3b 5 μM
3b 10 μM
3c 5 μM
3c 10 μM
Concentration (μM)

H

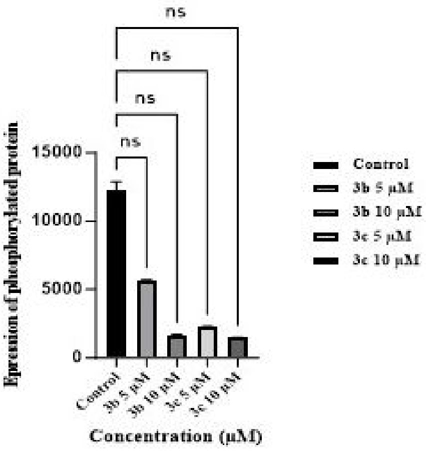

ns
ns
ns
ns
15000
10000
5000
0
Epression of phosphorylated protein
Control
3b 5 μM
3b 10 μM
3c 5 μM
3c 10 μM
Concentration (μM)

I

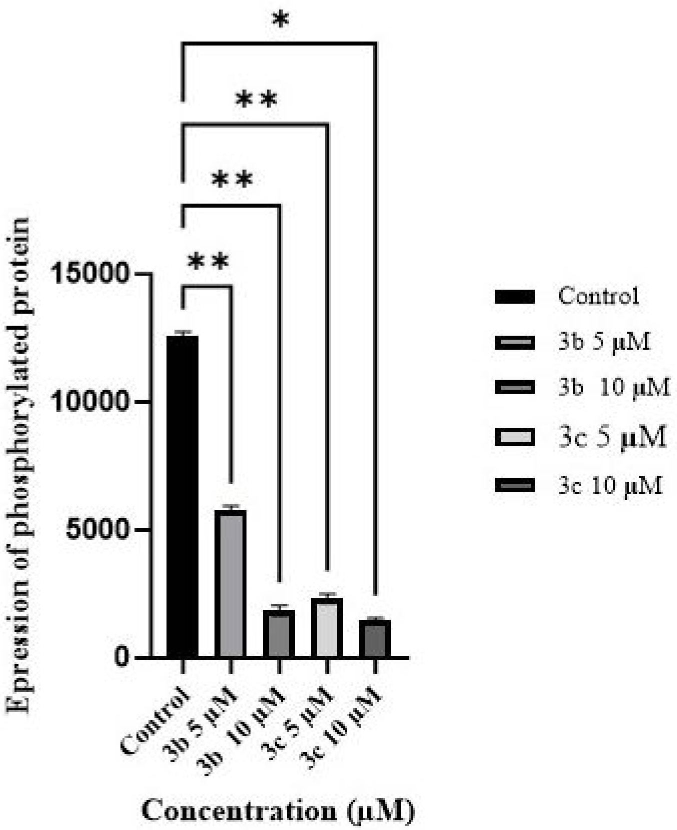

Figure S74. Collated densitometric measurement of cellular protein expression levels of (**A**) HCT-116 ERK1/4 (**B**) MCF-7 ERK1/4 (**C**) HCT-116 Mcl-1 (**D**) MCF-7 Mcl-1 (**E**) MCF-7 Bcl-1 (**F**) HCT-116 PARP cleavage (**G**) MCF-7 PARP cleavage 1 (**H**) HCT-116 Whole PARP observed following treatment with **3b** and **3c** at 5 μM and 10 μM (24 h treatment in each case) accompanied by a dose-dependent decrease and increase.

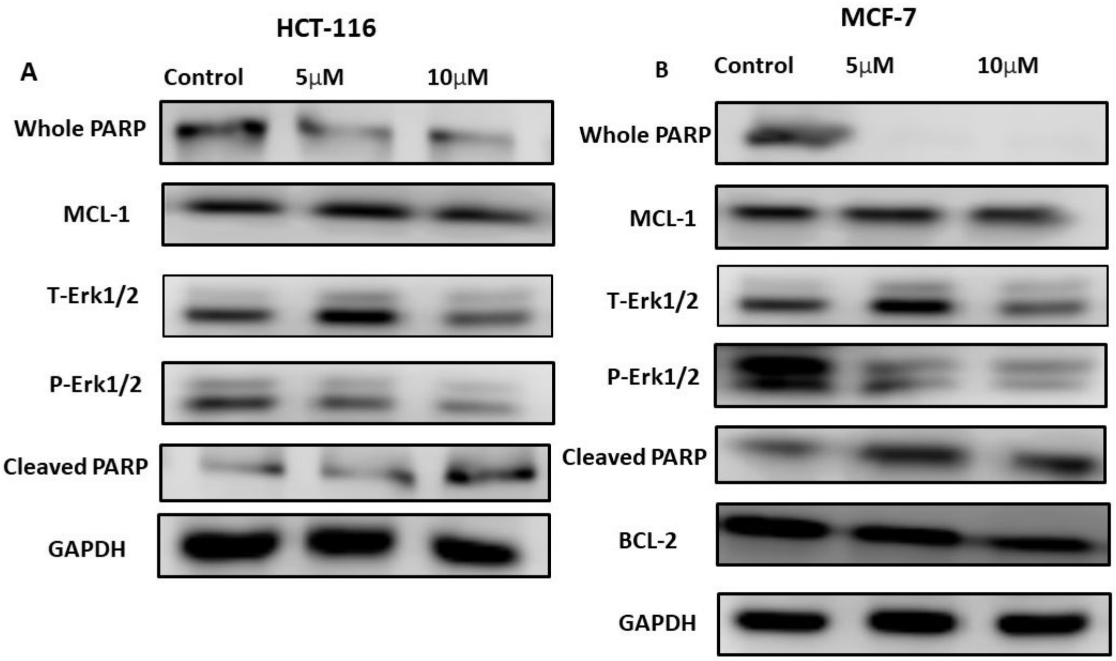

Figure S75. Representative of protein expression in HCT-116 and MCF-7 lysates following 24 h exposure of cells to **3c**. Lysates containing 50 µg protein were loaded into each well and proteins separated by SDS-PAGE. Western blots were performed using antibodies to detect whole and cleaved PARP, Mcl-1, Bcl-2, P-Erk1/2, T-Erk1/2 and a housekeeping gene GAPDH. Mean ±SD ≥3 independent trials.

■ 3.16 Proteomics

MCF-7 cells were seeded in dishes (100 × 20 mm) at a density of 2 × 10^6 per dish, allowed 24 h to attach, and exposed to test agent (**3c**, 1 or 10 µM, 24 h). Control samples of untreated MCF-7 cells were prepared similarly. Following required exposure time, cells were lysed in NP-40 lysis buffer supplemented with Phos- STOP Phosphatase Inhibitor Cocktail (Roche Applied Science) and COMPLETE-mini–Protease Inhibitor Cocktail (Roche Applied Science). Cell debris was removed by centrifugation at 14000 rpm for 5 min. Protein concentrations were determined using Quick-Start Bradford Assay (Bio-Rad, Hemel Hempstead, UK). Cell lysate proteins (50 µg) were digested via the S-trap protocol (Protifi) and peptides (4 µg) were analysed by DIA (SWATH) LC-MSMS mass spectrometry as previously described.[16] Data for the treated (triplicate) samples were compared to the controls using the R package Limma via StatsPro.[17] Greatest effects in protein concentration perturbations were seen for 10 µM treatments with **3c**. Details of the 3105 quantified proteins are reported in the file ‘Proteomics’ (Excel) as log2 fold changes vs. control.

■ 3.17 Detection of reactive oxygen species

The Promega ROS-Glo™ H_2O_2 (https://www.promega.co.uk) luminescent assay was conducted according to manufacturer`s protocol. Cells (5 × 10^3) were seeded in 96-well white opaque plates in 80 µL medium, overnight incubation before treatment with test agent (**3b** or **3c** at 5 or 10 µM) or cisplatin as positive control for 24 h. A hydrogen peroxide substrate solution, which reacts directly with H_2O_2 (or its equivalent), to create the ‘luciferin precursor’ was added and the solution incubated for 6 h. The ROS-Glo™ detection solution was introduced (100 µL) and the plates incubated for 20 min at room

temperature. The ROS solution (containing luciferase) converts any luciferin precursor present to luciferin producing light, allowing its detection. Relative luminescence was measured using an EnVision multilable plate reader (PerkinElmer). Results from this study are presented in Figure S71.

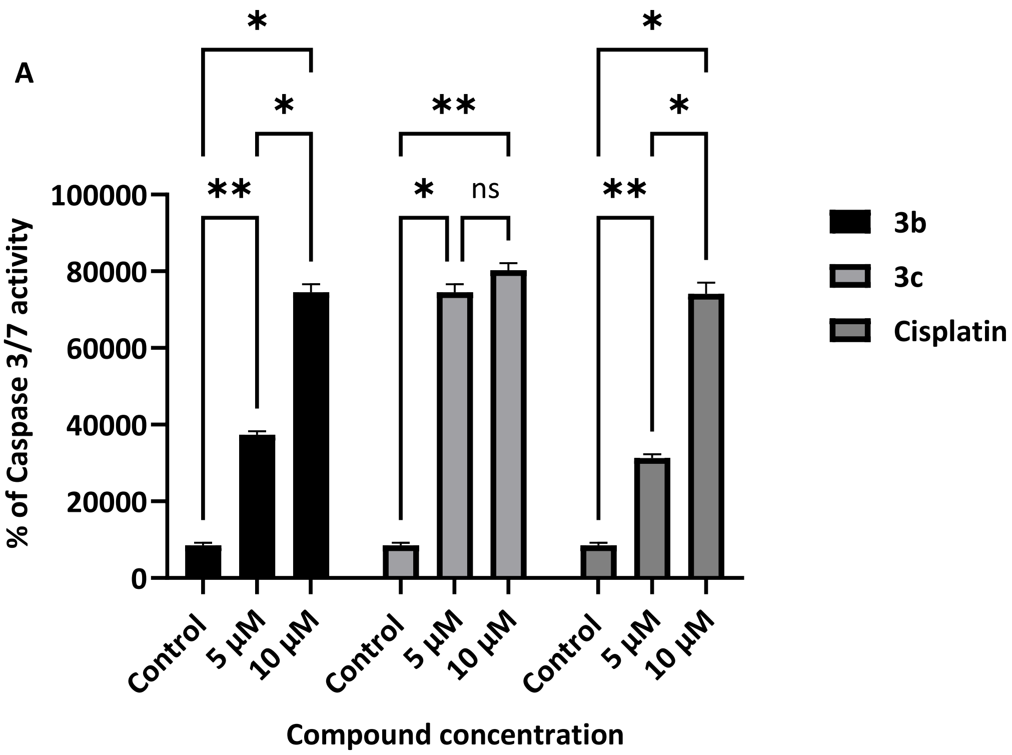

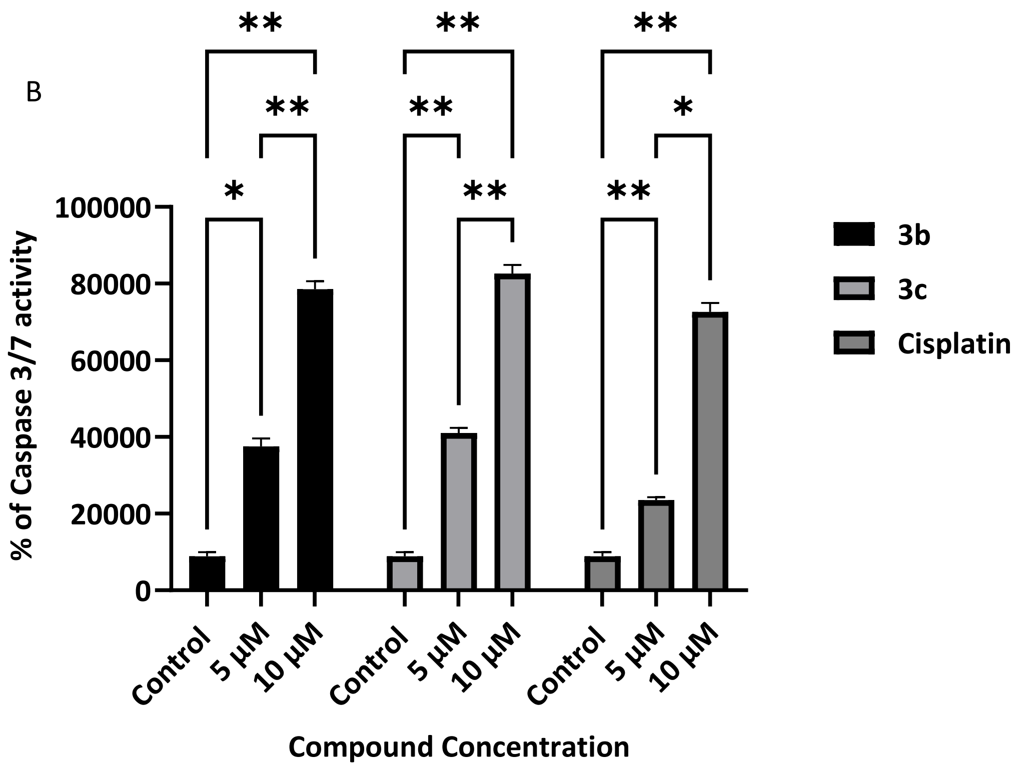

Figure S76. Test agents **3c** (**A**) and **3b** (**B**) (at 24 h) both significantly increased ROS production in HCT-116 and MCF-7 cells to levels comparable to cisplatin. Mean ± SEM ≥3 independent trials (n = 2 per trial). (p < **** and ***) for **A** and **B**.

■ 3.17 References

(1) Abid, M.; Nouch, R.; Bradshaw, T. D.; Lewis, W.; Woodward, S. Tripodal O-N-O Bis-Phenolato Amine Titanium(IV) Complexes Show High in Vitro Anti-Cancer Activity. *Eur. J. Inorg. Chem.* **2019**, *22*, 2774–2780. https://doi.org/10.1002/ejic.201900510

(2) Spackman, M. A.; Jayatilaka, D. Hirshfeld Surface Analysis. *Cryst. Eng. Comm.* **2009**, *11*, 19–32. https://doi.org/10.1039/b818330a

(3) Mackenzie, C. F.; Spackman, P. R.; Jayatilaka, D.; Spackman, M. A. Crystal Explorer Model Energies and Energy Frameworks: Extension to Metal Coordination Compounds, Organic Salts, Solvates and Open-Shell Systems. *IUCrJ* **2017**, *4*, 575–587. https://doi.org/10.1107/S205225251700848X

(4) Billo, E. J. Excel for Chemists: A Comprehensive Guide, 2nd Ed., **2001**, Wiley-VCH, New York.

(5) Miller, M.; Braitbard, O.; Hochman, J.; Tshuva, E. Y. Insights into Molecular Mechanism of Action of Salan Titanium (IV) Complex with in Vitro and in Vivo Anticancer Activity. *J. Inorg. Biochem.* **2016**, *163*, 250–257. https://doi.org/10.1016/j.jinorgbio.2016.04.007

(6) Cini, M.; Williams, H.; Fay, M. W.; Searle, M. S.; Woodward, S.; Bradshaw, T. D. Enantiopure Titanocene Complexes-Direct Evidence for Paraptosis in Cancer Cells. *Metallomics* **2016**, *8*, 286–297. https://doi.org/10.1039/C5MT00297D

(7) Meker, S.; Braitbard, O.; Hall, M. D.; Hochman, J.; Tshuva, E. Y. Specific Design of Titanium(IV) Phenolato Chelates Yields Stable and Accessible, Effective and Selective Anticancer Agents. *Chem. Eur. J.* **2016**, *22*, 9986–9995. https://doi.org/10.1002/chem.201601389
(8) Tzubery, A.; Melamed-Book, N.; Tshuva, E. Y. Fluorescent Antitumor Titanium(IV) Salen Complexes for Cell Imaging. *Dalton Trans.* **2018**, *47*, 3669–3673. https://doi.org/10.1039/c7dt04828a
(9) Khalil, G.; Orvain, C.; Fang, L.; Barloy, L.; Chaumont, A.; Gaiddon, C.; Henry, M.; Kyritsakas, N.; Mobian, P. Monomeric Ti (Iv)-Based Complexes Incorporating Luminescent Nitrogen Ligands: Synthesis, Structural Characterization, Emission Spectroscopy and Cytotoxic Activities. *Dalton Trans.* **2016**, *45*, 19072–19085. https://doi.org/10.1039/C6DT03477B
(10) Florès, O.; Trommenschlager, A.; Amor, S.; Marques, F.; Silva, F.; Gano, L.; Denat, F.; Campello, M. P. C.; Goze, C.; Bodio, E. In Vitro and in Vivo Trackable Titanocene-Based Complexes Using Optical Imaging or SPECT. *Dalton Trans.* **2017**, *46*, 14548–14555. https://doi.org/10.1039/c7dt01981e
(11) Volpe, P.; Eremenko-Volpe, T. Quantitative Studies on Cell Proteins in Suspension Cultures. *Eur. J. Biochem.* **1970**, *12*, 195–200. https://doi.org/10.1111/j.1432-1033.1970.tb00837.x
(12) Wiśniewski, J. R.; Hein, M. Y.; Cox, J.; Mann, M. A “Proteomic Ruler” for Protein Copy Number and Concentration Estimation without Spike-in Standards. *Mol. Cell. Proteomics* **2014**, *13*, 3497–3506. https://doi.org/10.1074/mcp.M113.037309
(13) Mosmann, T. Rapid Colorimetric Assay for Cellular Growth and Survival: Application to Proliferation and Cytotoxicity Assays. *J. Immunol. Meth.* **1983**, *65*, 55–63. https://doi.org/10.1016/0022-1759(83)90303-4
(14) Bruggisser R.; Von D. K.; Jundt. G.; Schaffner. W.; Tullberg. R. H. Interference of Plant Extracts, Phytoestrogens and Antioxidants with the MTT Thetrazolium Assay. *Planta Med.* **2002**, *68,* 5, 445–448. https://doi.org/10.1055/s-2002-32073
(15) Riccardi, C.; Nicoletti. I.; Migliorati. G.; Pigliacci. M. C.; Grignani. F. A Rapid and Simple Method for Measuring Thymocyte Apoptosis by Propidium Iodide Staining and Flow Cytometry. *J. Immunol. Meth.* **1991**, *139*, 271–279. https://doi.org/10.1016/0022-1759(91)90198-o
(16) Tong, D. L.; Boocock, D. J.; Coveney, C.; Saif, J.; Gomez, S. G.; Querol, S.; Rees, R.; Ball, G. R. A Simpler Method of Preprocessing MALDI-TOF MS Data for Differential Biomarker Analysis: Stem Cell and Melanoma Cancer Studies. *Clin. Proteomics* **2011**, *8,* 1–8. https://doi.org/10.1186/1559-0275-8-14.
(17) Yang, Y.; Cheng, J.; Wang, S.; Yang, H. StatsPro: Systematic integration and evaluation of statistical approaches for detecting differential expression in label-free quantitative proteomics. *J. Proteomics* **2022**, *250*, 104386. https://doi.org/10.1016/j.jprot.2021.104386
