# Supplementary material for: Probing the Mechanism of Action of Bis(phenolato) Amine (ONO Donor Set) Titanium(IV) Anticancer Agents
Source: J Med Chem. 2024 Feb 8;67(4):2732–44. doi: 10.1021/acs.jmedchem.3c01874 (PMC10895680; doi:10.1021/acs.jmedchem.3c01874)
Supplement: Supplementary file 8 — jm3c01874_si_008.pdf [file jm3c01874_si_008.pdf]

https://uniofnottm.sharepoint.com/sites/WoodwardResearchGroup/_layouts/15/download.aspx?SourceUrl=%2Fsites%2FWoodwardResearchGroup%2FShared%20Documents%2FGeneral%2FFor%20Tracey%2FCIF%20%283b%29%2Ecif

https://uniofnottm.sharepoint.com/:u:/r/sites/WoodwardResearchGroup/Shared%20Documents/General/For%20Tracey/CIF%20(3b).cif?csf=1&web=1&e=cWl39U

https://uniofnottm.sharepoint.com/:u:/r/sites/WoodwardResearchGroup/Shared%20Documents/General/For%20Tracey/CIF%20(3c).cif?csf=1&web=1&e=DpDEDQ

https://uniofnottm.sharepoint.com/:u:/r/sites/WoodwardResearchGroup/Shared%20Documents/General/For%20Tracey/CIF%20(3i).cif?csf=1&web=1&e=hLvLeF

https://uniofnottm.sharepoint.com/:u:/r/sites/WoodwardResearchGroup/Shared%20Documents/General/For%20Tracey/CIF%20(3k).cif?csf=1&web=1&e=sJmUO1
